# Novel 3-Dehydroteasterone Derivatives with 23,24-Dinorcholanic Side Chain and Benzoate Groups at C-22: Synthesis and Activity Evaluation by Rice Lamina Inclination Test and Bean Second-Internode Bioassay

**DOI:** 10.3390/ijms26178710

**Published:** 2025-09-06

**Authors:** Ernesto Valdés, Katy Díaz, María Núñez, Andrés F. Olea, José F. Quilez del Moral, Rodrigo Carvajal, Mauricio A. Cuellar, Luis Espinoza-Catalán

**Affiliations:** 1 Departamento de Química, Universidad Técnica Federico Santa María, Avenida España 1680, Valparaíso 2340000, Chile; ernesto.valdes@usm.cl (E.V.); katy.diaz@usm.cl (K.D.); maria.nunezg@usm.cl (M.N.); 2 Grupo QBAB, Instituto de Ciencias Aplicadas, Facultad de Ingeniería, Universidad Autónoma de Chile, Av. del Valle Sur 534, Santiago 8580640, Chile; 3 Departamento de Química Orgánica, Instituto de Biotecnología, Universidad de Granada, 18071 Granada, Spain; jfquilez@ugr.es; 4 Facultad de Farmacia, Escuela de Química y Farmacia, Universidad de Valparaíso, Av. Gran Bretaña 1093, Valparaíso 2340000, Chile; rodrigo.carvajal@usm.cl (R.C.); mauricio.cuellar@uv.cl (M.A.C.); 5 Centro de Investigación, Desarrollo e Innovación de Productos Bioactivos (CINBIO), Universidad de Valparaíso, Valparaíso 2340000, Chile

**Keywords:** 3-dehydroteasterone, brassinosteroid analogs, synthesis, plant growth

## Abstract

Herein, a new series of 3-DT analogs with benzoylated groups at C-23 are synthesized and characterized. The benzoylated groups carry the same substituents in the *ortho*- or *para*-positions. Thus, the effect of structure on activity, measured using the rice lamina inclination test (RLIT) and the bean second-internode assay (BSI), is evaluated. The RLIT results indicate that a benzoylate function at C-22 induces a strong increase in activity that depends on the position and nature of the substituent in the phenyl ring. For example, an analog with an -OAc group in the *ortho*-position is the most active derivative, and its activity is like that of brassinolide. A relative index is calculated using brassinolide as a positive control to compare the RLIT results with those reported previously. This analysis allows for the conclusion that benzoylated derivatives with a hydroxyl group at C-3 are much more active than the corresponding analogs with a carbonyl group in this position, and one extra alcohol group in the alkyl chain decreases RLIT activity. Finally, the results obtained with the BSI are clearly different to those obtained in the RLIT bioassay. Therefore, the application of any activity–structure relationship will always be dependent on the bioassay used to determine activity.

## 1. Introduction

**Figure 1:**
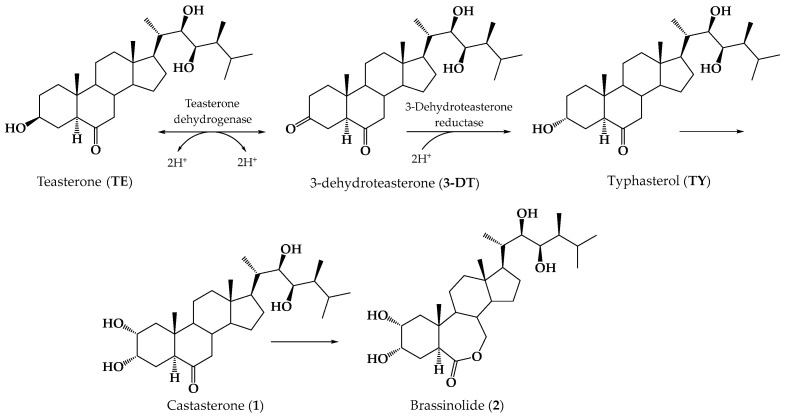
Abbreviated biosynthetic pathway of brassinolide (**2**) from teasterone (TE), 3-dehydroteasterone (3-DT), typhasterol (TY), and castasterone (**1**).

It is well established that the biosynthesis of brassinolide occurs by two parallel routes, connected at different steps, and both lead to castasterone, which is finally converted to brassinolide. In one of these pathways (Figure 1), epimerization of the C-3 hydroxyl group of teasterone (TE) leads to thyphasterol (TY) through 3-dehydroteasterone (3-DT), which is a very short-lived biosynthetic intermediate [1,2,3,4]. Typhasterol is subsequently hydroxylated at C-2 to give castasterone (**1**). Finally, an oxygen atom is inserted between C-6 and C-7 to form brassinolide (**2**) [1,2,3,4].

The biological activities of brassinolide (**2**), castasterone (**1**), and its precursors have been examined using the rice lamina inclination test (RLIT) and wheat leaf unrolling assay. In both bioassays, brassinolide (**2**) and castasterone (**1**) exhibit much higher activities than TE, 3-DT, and TY, whose activities are almost the same [3]. These results suggest that chemical modifications of precursors that produce **1** and **2**, two of the most active natural brassinosteroids, could provide a synthetic means of obtaining active compounds.

**Figure 2:**
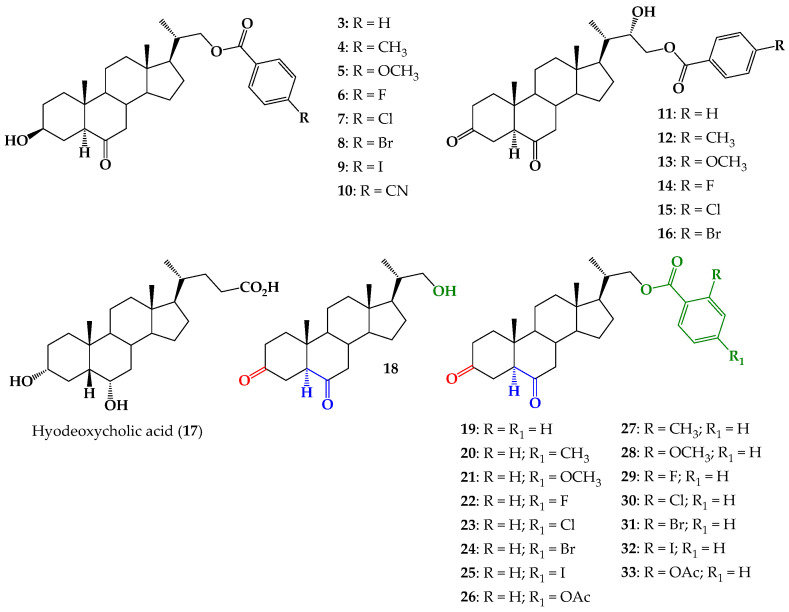
Structure of 3-hydroxy-6-oxo-23,24-dinorcholans with benzoate function at C-22 (compounds **3**–**10**, TE analogs), 3,6-dioxo-24-norcholans with benzoate function at C-23 (compounds **11**–**16**, 3-DT analogs), hyodeoxycholic acid (**17**), 3,6-dioxo-22-hydroxy-23,24-dinorcholan (synthetical precursor, **18**), and new 3,6-dioxo-23,24-dinorcholans with benzoate function at C-22 (compounds **19**–**33**, 3-DT analogs).

Following this line, a series of TE and 3-DT derivatives with modifications in the alkyl chain, compounds **3**–**10** and **11**–**16** in Figure 2, respectively, have been synthesized, and their bioactivity has been assessed using different bioassays [5].

More specifically, the synthesis of 3-hydroxy-6-oxo-23,24-dinorcholans with a benzoate function at C-22 (compounds **3**–**10**, TE analogs) has recently been reported [6]. Assessments of the bioactivity of these analogs by using the RLIT indicate that, at the lowest tested concentration (1 × 10^−8^ M), TE analogs in which the aromatic ring is substituted in the *para*-position with methoxy (**5**), I atom (**9**), and CN (**10**) are more active than brassinolide (50–72%) and 2–3 times more active than analogs in which the substituent group is F, Cl, or Br atoms [6]. Similarly, a series of 24-Nor-22(*S*)-hydroxy side chains with a *p*-substituted benzoate function at C-23 (compounds **11**–**16**, 3-DT analogs) has also been synthesized and evaluated using the RLIT [7]. The obtained results show that all these 3-DT analogs exhibit much lower activity than brassinolide. In addition, compound **16**, i.e., an analog with *p*-Br in the aromatic ring, is the most active compound tested at 1 × 10^−8^ M [7].

Thus, by comparing the RLIT activities of these two series of compounds, it can be concluded that TE analogs are more active than 3-DT analogs. The main structural differences between these two series are in the side alkyl chain. For TE analogs, **3**–**10**, the benzoylated groups are attached to C-22, whereas in 3-DT analogs, **11**–**16,** these groups are attached to C-23 and leave a hydroxyl group at C-22.

Thus, considering the different effects on biological activity observed between TE analogs and 3-DT analogs, herein, the synthesis of a new series of 3-DT analogs with benzoylated groups at C-22 is described. In this way, we intend to elucidate the importance of the shortest alkyl chain with no hydroxyl group at C-22 on the biological activity of 3-DT analogs. Compounds with different substituents in the *para*- (**19**–**26**) and *ortho*-positions (**27**–**33**) of the aromatic ring are obtained, as shown in Figure 2. All obtained compounds are characterized by IR, HRMS, 1D, and 2D NMR spectroscopic techniques, and their bioactivity is evaluated by RLIT and BSI bioassays [6,7].

The results obtained herein can be used to determine and compare the effect on the bioactivity of substituents in the “*ortho*”- and “*para*”-positions of the aromatic ring, as it has already been described for other aryl or phenyl analogs of the brassinosteroid type [8,9]. Finally, a new structural requirement in the side chain for this type of BR analog is established.

## 2. Results and Discussion

### 2.1. Chemical Synthesis

**Scheme 1:**
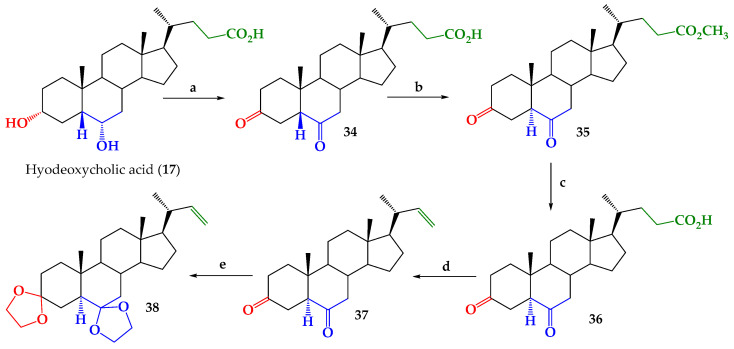
Synthesis of compounds **34**–**38**. Conditions **a**. Jones (CrO_3_/H_2_SO_4_/(CH_3_)_2_CO), r.t. 4h, 98.0%. **b**. HCl 2.5% *v*/*v*, CH_3_OH, reflux, 2h, 95.8%. **c**. K_2_CO_3_ 15.0% *w*/*v*, CH_3_OH, reflux, 1 h, HCl 2.5% *v*/*v*, 98.4%. **d**. Pb(OAc)_4_/Cu(OAc)_2_/C_5_H_5_N/C_6_H_6_, reflux, 4h, 36.8%. **e**. HOCH_2_CH_2_OH/TsOH/C_6_H_6_, reflux, 3h, 91.3%.

Commercially available hyodeoxycholic acid (**17**) (Figure 2 and Scheme 1) (AK Scientific Inc.) was used as a starting material for the preparation of new 3-DT analogs **19**–**33**. The first part of the synthetic route is obtaining alkene **38** (Scheme 1). Following a described procedure, deoxycholic acid (**17**) is completely oxidized with Jones reagent to produce 3,6-dioxo acid **34** with 98.0% yield [7]. IR and NMR spectroscopic data of **34** are consistent with those previously reported [7]. Subsequently, isomerization of C-5 from 5β to 5α on compounds **34** to **35** is performed under acidic conditions (HCl 2.5%, CH_3_OH, reflux) according to a reported procedure, with 95.8% yield [7,10]. The structure of compound **35** is confirmed by ^1^H and ^13^C and 2D NMR spectroscopy (Figures S1–S5, Supplementary Material). Thus, in the ^1^H NMR spectrum, the signal observed at δ = 3.65 ppm (s, 3H) is assigned to the CH_3_O group at C-24, while the signal at δ = 2.62–2.53 (m, 2H) is assigned to H-5 and H-2. In the ^13^C NMR spectrum, the signal observed at δ = 174.57 and δ = 51.47 ppm confirm the presence of a methyl ester group (-CO_2_CH_3_). Isomerization from 5β (compound **34**) to 5α (compound **35**) is shown by the change in field chemical shift of C-5 signal, from δ = 59.69 to 57.43 ppm. The saponification reaction of **35** with K_2_CO_3_/CH_3_OH (15.0% *w*/*v*) reflux, followed by acidification (HCl 2.5% *v*/*v*), gives acid **36** with 98.4% yield. The presence of the carboxylic function is confirmed by the signal appearing at δ = 179.88 ppm in the ^13^C NMR spectrum (Figures S6–S10, Supplementary Material). Oxidative decarboxylation of the side chain in compound **36**, with Pb(OAc)_4_/Cu(OAc)_2_/C_5_H_5_N/C_6_H_6_ under reflux, and following a reported procedure [11], leads to olefin **37** with 36.8% yield. The formation of a terminal double bond at the C-22 position is confirmed by ^1^H-NMR and ^13^C-NMR spectra, and by ^13^C DEPT-135, 2D HSQC and 2D HMBC NMR experiments (Figures S11–S15, Supplementary Material). All NMR spectroscopic data registered for compound **37** are consistent with that reported in [8]. The protection of keto groups of compound **37** with an ethylene glycol/TsOH/C_6_H_6_ system gives the bisdioxolane derivative **38**. The presence of bisdioxolane groups in compound **38** is confirmed by signals observed at δ = 3.97–3.87 ppm (m, 7H) and δ = 3.76–3.72 ppm (m, 1H) in ^1^H NMR spectrum, and signals observed at δ = 65.39, 64.26, 64.16, and 64.10 ppm in the ^13^C NMR spectrum, which are assigned to four carbinol carbons of bisdioxolane groups (Figures S16–S20, Supplementary Material).

The second part of the synthetic route, shown in Scheme 2, starts with Lemieux–Johnson oxidation of **38** with OsO_4_/NaIO_4_/THF/H_2_O to obtain aldehyde **39** with 57.1% yield [12]. ^1^H and ^13^C NMR spectroscopic data obtained for **39** (Figures S21–S25, Supplementary Material) are consistent with those previously reported [13]. Reduction of aldehyde **39** with NaBH_4_/THF/CH_3_OH at 40 °C [14], followed by acid hydrolysis (HCl 5% *v*/*v* [6]), produces primary alcohol **18** with 80.8% yield. Primary alcohol at C-22 and both ketone functions at C-3 and C-6 are identified by ^1^H and ^13^C NMR spectroscopies. Thus, in the ^1^H NMR spectrum, the signals appearing at δ = 3.63 ppm (1H, dd, *J* = 10.6 and 3.3 Hz) and 3.38 ppm (1H, dd, *J* = 10.6 and 6.7 Hz) are assigned to carbinolic and diasterotopic hydrogen atoms H-22a and H-22b. In contrast, in the ^13^C NMR spectrum, signals at δ = 211.25, 209.02 and 67.76 ppm appear, which are assigned to carbon atoms of carbonyl groups C-3 and C-6, and carbinolic carbon atom C-22, respectively. Additionally, the full molecular structure is assigned by ^13^C DEPT-135, 2D HSQC and 2D HMBC NMR experiments (Figures S26–S30, Supplementary Material). Finally, benzoylation of the primary alcohol at C-22 with the corresponding benzoyl chlorides, following a reported protocol [7,11,15], gives new 3,6-dioxo BRs analogs **19**–**33** (3-DT derivatives) with yields ranging from 18.4% to 76.9% (Scheme 2). Compounds **19**–**33** are fully characterized by the IR, 1D, 2D NMR (Figures S31–S105, Supplementary Material) and HRMS (Figures S106–S121, Supplementary Material) spectroscopic techniques.

### 2.2. Biological Activity

#### 2.2.1. Bioactivity in the Rice Lamina Inclination Test (RLIT)

The biological activities of precursor **18** and new **19**–**33** derivatives have been evaluated by using RLIT, a highly sensitive and specific assay for BRs [6,7]. Leaf–sheath bending angles are measured in growing rice plants in the absence and presence of different concentrations of exogenously applied BR analogs. The effect of BR analogs on bending angle is calculated as described in the Material and Methods, and RLIT activities at different concentrations of 3-DT derivatives are presented in Table 1.

By comparing the RLIT activities of 3-DT derivatives and **18**, it can be concluded that a benzoate function at the C-22 position induces a strong increase in activity, which depends on the position and nature of the phenyl substituent. Additionally, experimental data show that RLIT activities are concentration-dependent, i.e., brassinolide activity increases with increasing concentration. This behavior is shown only by derivatives **21** and **29**. Therefore, the analysis of structure effect on activity must be performed separately for each tested concentration.

At the lowest tested concentration, the data indicate that the highest activity corresponds to analog **33**, which has an acetyl group substitution in the *ortho*-position of the aromatic ring, and its activity is slightly higher than that obtained for brassinolide. Interestingly, the analog with this substituent in the *para*-position, **26**, shows slightly lower activity. A similar effect is observed for derivatives with methoxy and a Br atom as substituents (compare the activities of **21** with **28**, and **24** with **31**, respectively). The opposite effect, i.e., lowest activities for the *ortho*-substitution, are obtained for methyl (**20** and **27**), F (**22** and **29**), and Cl (**23** and **30**) atoms. Finally, for the I atom, the substitution position has no effect on RLIT activity (**25** and **32**). Thus, at the lowest tested concentration, the results obtained for the activity of 3-DT derivatives indicate that a substituent attached to the *ortho*- or *para*-positions produces different effects on activities.

The effect of increasing activity with increasing concentration has been observed only for brassinolide (**2**), **21**, and **29**. Therefore, at concentrations higher than 1 × 10^−8^ M, 3-DT analogs become less active than brassinolide (**2**). By comparing 3-DT activities at 1 × 10^−7^ M, it is seen that the most active analogs are **20** and **25**, substituted in *para*-position with a methyl group and an I atom, respectively, and **26** and **33**, which are substituted with an acetyl group in the *para*- and *ortho*-positions, respectively. The same comparison at 1 × 10^−6^ M show that **21** and **29**, which are substituted with a methoxy group in the *para*-position and a F atom in the *ortho*-position, respectively, are the most active derivatives.

It is worth mentioning that the effect of substituent position is also dependent on concentration. In other words, the ratio of activities obtained for derivatives with substituents in the *ortho*- or *para*-positions changes with concentration as well. Thus, the chemical structure of 3-DT derivatives is somehow related to their RLIT activity. It has been shown that the RLIT process occurs thanks to a very complex mechanism, and therefore, it is not possible to attribute this behavior to a special step [16].

A main aim of this work is to obtain a more general relationship between the measured RLIT activity and the chemical structure of benzoylated BRs. To allow for a comparison of activity data obtained under varying experimental conditions, it is essential to include the positive control, brassinolide, in the calculation of RLIT activity. This goal is accomplished by defining a relative activity (RA), calculated by dividing the activity value of each tested compound by that of brassinolide under the same conditions (see the Experimental Section). Thus, the relative activity values obtained herein at 1 × 10^−8^ M are listed in Table 2 along with those calculated from data previously reported for two series of compounds, namely, TE and 3-DT (C22-OH) derivatives.

A comparison of the results obtained herein (**19**–**25**) with those described in reference [6] (**3**–**9**) indicate that derivatives with a -OH group in C-3 are much more active than derivatives with a carbonyl group in this position. This effect is observed even for precursors of benzoylated derivatives, and the only exceptions are compounds **22** and **6** that exhibit the same activity. The key role played by the hydroxyl group in this position has been already established [17,18]. On the other hand, by comparing results obtained for compounds **11**–**15** with **18**–**23**, it becomes clear that an extra alcohol group in the alkyl chain decreases RLIT activity. Thus, these results represent a contribution to the understanding of how the structure of BR benzoylated analogs determine RLIT activity, but so far, it is not possible to establish a consistent structure–activity relationship.

Another important aspect to highlight is that data for analogs substituted in the *ortho*-positions are scarce, and benzoylated analogs with substitution with acetate group (**33**) exhibit interesting activity on the RLIT bioassay.

#### 2.2.2. Bioactivity in Bean Second-Internode Bioassay (BSI)

All 3-DT derivatives, **19**–**33**, and its synthetic precursor **18**, have also been tested using the BSI bioassay. The values of the bean second-internode elongation have been obtained for BR analogs applied at 1 × 10^−8^ M concentration. This concentration was chosen because it has been shown that the optimal response for brassinolide in the range 1 × 10^−5^–1 × 10^−10^ is obtained at this concentration [19]. The results are shown in Table 3 and Figure S123, Supplementary Materials.

The data listed in Table 3 indicate that, in the BSI bioassay and at the concentration of 1 × 10^−8^ M, analogs **22** and **27**–**30** show biological activity comparable to that of brassinolide, whereas analog **21** (with OCH_3_ group substituent in the “*para*” position of the aromatic ring), exhibits biological activity that is twofold that exhibited by brassinolide (**2**). This is the only case in which a derivative with a substituent in the *para*-position is more active than the corresponding *ortho*-isomer. In all other cases, **27**–**30** and **32**, the ortho-derivatives are significantly more active in the BSI bioassay.

These results are clearly different from those obtained in the RLIT bioassay, and therefore, it can be concluded that the activity of BR derivatives cannot be determined by just one bioassay. So, their application and activity–structure relationship will always be dependent on the bioassay used to determine activity.

## 3. Materials and Methods

### 3.1. General Chemicals and Methods

All reagents were purchased from commercial suppliers and used without further purification. Melting points were measured on a SMP3 apparatus (Stuart-Scientific, now Merck KGaA, Darmstadt, Germany) and left uncorrected. ^1^H-, ^13^C-, ^13^C DEPT-135, gs 2D HSQC, and gs 2D HMBC NMR spectra were recorded in CDCl_3_ solutions and were referenced to the residual peaks of CHCl_3_ at δ = 7.26 ppm and δ = 77.00 ppm for ^1^H and ^13^C, respectively, on an Avance Neo 400 Digital NMR spectrometer (Bruker, Rheinstetten, Germany) operating at 400.1 MHz for ^1^H and 100.6 MHz for ^13^C. Chemical shifts are reported in δ ppm, and coupling constants (*J*) are given in Hz. Multiplicities are reported as follows: singlet (s), doublet (d), broad doublet (bd), doublet of doublets (dd), doublet of triplets (dt), triplet (t), broad triplet (bt), quartet (q), doublet of quartet (dq), doublet of double doublets (ddd), triplet of triplets (tt), and multiplet (m). IR spectra were recorded as KBr disks in a FT-IR 6700 spectrometer (Nicolet, Thermo Scientific, San Jose, CA, USA), and frequencies are reported in cm^−1^. High-resolution mass spectra (HRMS-ESI) were recorded in a Bruker Daltonik. The analysis for the reaction products was performed with the following relevant parameters: dry temperature, 180 °C; nebulizer, 0.4 Bar; dry gas, 4 L/min; and spray voltage, 4.5 kV in positive mode. Accurate mass measurements were performed at a resolving power: 140,000 FWHM in the range *m*/*z* 50–1300. For analytical TLC, silica gel 60 in a 0.25 mm layer was used, and TLC spots were detected by heating after spraying with 25% H_2_SO_4_ in H_2_O. Chromatographic separations were carried out by conventional column on silica gel 60 (230–400 mesh) using hexane–EtOAc mixtures of increasing polarity. All organic extracts were dried over anhydrous magnesium sulfate and evaporated under reduced pressure, below 40 °C.

### 3.2. Synthesis

#### 3.2.1. 3,6-Dioxo-5β-cholan-24-oic acid (**34**)

Compound **34** is prepared from hyodeoxycholic acid (**17**) according to a described protocol, with 98% yield. All characterization data are consistent with those reported [7].

#### 3.2.2. Methyl 3,6-Dioxo-5α-cholan-24-oate (**35**)

Compound **34** (2.00 g, 5.15 mmol) is dissolved in 200 mL of 2.5% *v*/*v* HCl-MeOH and refluxed for 2 h. The end of reaction is verified by TLC. The solvent is evaporated under reduced pressure, and the residue is dissolved in EtOAc (60 mL). The solution is washed with a saturated solution of NaHCO_3_ (3 × 50 mL) and water (3 × 40), and the organic layer is dried over MgSO_4_. Finally, the solvent is evaporated by using a rotary evaporator. Compound **35** (1.99 g, 95.8% yield) is obtained as a colorless solid; m.p. = 105.2–106.9 °C (CH_2_Cl_2_). IR_νmax_ (KBr, cm^−1^): 2947 and 2867 (C-H); 1736 and 1712 (C=O); 1433 (CH2-); 1384 (CH3-); 1239 and 1166 (C-O). ^1^H NMR (400.1 MHz, CDCl_3_) δ (ppm): 3.65 (3H, s, OCH_3_); 2.62–2.53 (2H, m, H-5 and H-2); 2.21 (1H, ddd, *J* = 18.6, 9.8 and 9.4 Hz, H-22a); 2.09 (1H, dd, *J* = 6.4 and 2.4 Hz, H-1α); 1.99 (1H, dd, *J* = 12.8 and 12.5 Hz, H-7α); 0.942 (3H, s, H-19); 0.916 (3H, d, *J* = 6.6 Hz, H-21); 0.678 (3H, s, H-18). ^13^C NMR (100.6 MHz, CDCl_3_) δ (ppm): 211.23 (C-3); 209.01 (C-6); 174.57 (C-24); 57.43 (C-5); 56.49 (C-14); 55.71 (C-17); 53.34 (C-9); 51.47 (OCH_3_); 46.51 (C-7); 42.98 (C-13); 41.17 (C-10); 39.27 (C-12); 38.02 (C-1); 37.93 (C-20); 37.32 (C-4); 36.93 (C-2); 35.22 (C-8); 30.98 (C-22); 30.82 (C-23); 27.85 (C-16); 23.90 (C-15); 21.59 (C-11), 18.17 (C-21); 12.50 (C-19); 11.97 (C-18) (Figures S1–S5, Supplementary Material).

#### 3.2.3. 3,6-Dioxo-5α-cholan-24-oic Acid (**36**)

Compound **35** (2.00 g, 4.97 mmol) dissolved in CH_3_OH (120 mL) and in presence of 15% *w*/*v* K_2_CO_3_ is refluxed for 1h. The end of the reaction is verified by TLC. The solvent is evaporated under reduced pressure, and the residue is dissolved in water (50 mL) and neutralized with HCl 2.5% *v*/*v* until pH = 3. EtOAc (60 mL) is added to the solution and washed with water (3 × 50), and the organic layer is dried over MgSO_4_. Finally, the solvent is evaporated in a rotary evaporator. Compound **36** (1.90 g, 98.4% yield) is obtained as a colorless solid; m.p. = 196.3–198.3 °C (CH_2_Cl_2_). IR_νmax_ (KBr, cm^−1^): 3500–2600 (OH); 2945 and 2868 (C-H); 1710 (C=O); 1416 (CH_2_-); 1384 (CH_3_-); 1240 and 1165 (C-O). ^1^H NMR (400.1 MHz, CDCl_3_) δ (ppm): 2.61–2.53 (2H, m, H-5 and H-2), 2.09 (1H, dd, *J* = 6.4 and 2.4 Hz, H-1α); 2.00 (1H, dd, *J* = 12.8 and 12.3 Hz, H-7α); 0.942 (3H, s, H-19), 0.928 (3H, d, *J* = 7.1 Hz, H-21), 0.681 (3H, s, H-18). ^13^C NMR (100.6 MHz, CDCl_3_) δ (ppm): 211.47 (C-3); 209.12 (C-6); 179.88 (C-24); 57.43 (C-5); 56.48 (C-14); 55.69 (C-17); 53.33 (C-9); 46.50 (C-7); 43.00 (C-13); 41.18 (C-10); 39.27 (C-12); 38.01 (C-1); 37.93 (C-20); 37.30 (C-4); 36.92 (C-12); 35.17 (C-2); 30.91 (C-22); 30.57 (C-23); 27.84 (C-16); 23.90 (C-15); 21.59 (C-11), 18.15 (C-21); 12.51 (C-19); 11.98 (C-18) (Figures S6–S10, Supplementary Material).

#### 3.2.4. 24-Nor-5α-chol-22-ene-3,6-dione (**37**)

To a solution of compound **36** (2.00 g, 5.15 mmol) in dry benzene (60 mL), Cu(OAc)_2_·H_2_O_2_ (200 mg, 1.00 mmol) and pyridine (0.8 mL) are added. The reaction mixture is refluxed, and Pb(OAc)_4_ (5.6 g, 12.6 mmol) is added in four portions every 1 h. Then, the mixture is filtered, and the solvent is evaporated under reduced pressure. The crude is diluted with EtOAc (50 mL) and washed with water (3 × 20 mL), and the organic layer is dried over MgSO_4_. Then, the solvent is evaporated in vacuo, and the crude is redissolved in CH_2_Cl_2_ (7 mL) and chromatographed on silica gel with hexane–EtOAc mixtures of increasing polarity (19.8:0.2 → 15.8:4.2). Compound **37** (0.65 g, 36.8%) is obtained as a colorless solid; m.p. = 148.9–149.7 °C (AcOEt/hex.). IR_νmax_ (KBr, cm^−1^): 3073 (CH=CH_2_); 2946, 2870 and 2866 (C-H); 1712 (C=O); 1638 (C=C); 1460 (CH_2_-); 1389 (CH_3_-); 1248 and 1216 (C-O); 906 (CH=CH_2_). ^1^H NMR (400.1 MHz, CDCl_3_) δ (ppm): 5.65 (1H, ddd, *J* = 17.2, 10.2 and 8.5 Hz, H-22); 4.91 (1H, dd, *J* = 17.2 and 1.8 Hz, H*_trans_*-23); 4.83 (1H, dd, *J* = 10.2 and 1.8 Hz, H*_cis_*-23); 2.62–2.54 (2H, m, H-5 and H-2); 2.00 (1H, t, *J* = 13.0 Hz, H-7α); 1.86 (1H, ddd, *J* = 14.6, 10.3 and 4.2 Hz, H-8); 1.43 (1H, ddd, *J* = 16.7, 12.7 and 3.7 Hz, H-11β); 1.10 (1H, ddd, *J* = 18.0, 11.7 and 5.9 Hz, H-15); 1.03 (3H, d, *J* = 6.6 Hz, H-21); 0.953 (3H, s, H-19); 0.712 (3H, s, H-18). ^13^C NMR (100.6 MHz, CDCl_3_), δ (ppm): 211.23 (C-3); 209.03 (C-6); 144.75 (C-22); 111.92 (C-23); 57.47 (C-5); 56.58 (C-14); 55.34 (C-9); 53.44 (C-17); 46.55 (C-7); 42.95 (C-13); 41.22 (C-10); 41.06 (C-20); 39.22 (C-12); 38.06 (C-1); 37.96 (C-8); 37.35 (C-2); 36.96 (C-4); 28.15 (C-16); 23.95 (C-15); 21.62 (C-11); 20.03 (C-21); 12.54 (C-19); 12.17 (C-18) (Figures S11–S15, Supplementary Material).

#### 3.2.5. 3,6-Didioxolan-24-nor-5α-cholan-22-ene (**38**)

To a solution of compound **37** (1.00 g, 2.92 mmol) in C_6_H_6_ (80 mL), ethylene glycol (7.2 mL, 129.9 mmol) and TsOH (140 mg, 0.81 mmol) are added. The solution is refluxed for 3 h, using a Dean-Stark apparatus. Later, a saturated solution of NaHCO_3_ (80 mL) is added to quench the reaction and dissolved with EtOAc (60 mL). The organic layer is washed with water (2 × 25 mL) and dried over MgSO_4_. Finally, the solvent is evaporated in vacuo. Compound **38** (1.15 g, 91.3% yield) is obtained as a colorless solid; m.p. = 108.3–109.4 °C (EtOAc). IR_νmax_ (KBr, cm^−1^): 2961, 2930, 2905, 2875 and 2862 (C-H); 1639 (C=C); 1459 (CH_2_-); 1355 (CH_3_-); 1256, 1229, 1198, 1180, 1106, 1090 and 1042 (C-O); 906 (CH=CH_2_). ^1^H NMR (400.1 MHz, CDCl_3_) δ (ppm): 5.64 (1H, ddd, *J* = 17.2, 10.2 and 8.5 Hz, H-22); 4.88 (1H, dd, *J* = 17.1 and 1.7 Hz, H*_trans_*-23); 4.80 (1H, dd, *J* = 10.1 and 1.7 Hz, H*_cis_*-23); 3.96–3.83 (7H, m, dioxolane); 3.77–3.71 (1H, m, dioxolane); 2.10–2.01 (1H, m, H-20); 1.95 (1H, dt, *J* = 12.5 and 3.2 Hz, H-12α); 1.08–0.979 (3H, m, H-15, H-7 and H-5); 1.01 (3H, d, *J* = 6.6 Hz, H-21); 0.937 (3H, s, H-19); 0.793 (1H, dd, *J* = 12.1 and 4.1 Hz, H-17); 0.688 (3H, s, H-18). ^13^C NMR (100.6 MHz, CDCl_3_), δ (ppm): 145.24 (C-22); 111.52 (C-23); 109.70 (C-3 and C-6); 65.41; 64.28; 64.17 and 64.12 (dioxolane); 56.00 (C-5); 55.40 (C-14); 53.32 (C-9); 49.56 (C-17); 42.58 (C-13); 41.18 (C-20 and C-7); 39.83 (C-12); 37.23 (C-1); 36.83 (C-10); 33.38 (C-8); 31.02 (C-2); 29.23 (C-4); 28.33 (C-16); 24.16 (C-15); 21.00 (C-11); 20.08 (C-21); 13.52 (C-19); 12.22 (C-18) (Figures S16–S20, Supplementary Material).

#### 3.2.6. 3,6-Didioxolan-23,24-dinor-5α-cholan-22-al (**39**)

To a solution of compound **38** (600mg, 1.39 mmol) in THF (30 mL), H_2_O (18 mL) and NaIO_4_ (900 mg, 4.21 mmol) are added at room temperature. Later, 10% *v*/*v* OsO_4_-H_2_O (0.7 mL) is added under inert gas atmosphere (N_2_). After 6 h, a solution of 5% *w*/*v* Na_2_SO_3_-H_2_O (5 mL) is added and stirred for 15 min (TLC). THF is evaporated under reduced pressure, and the resulting mixture is diluted with EtOAc (50 mL), washed with water (2 × 10 mL), and dried over MgSO_4_. Finally, the solvent is evaporated under reduced pressure, and the crude is chromatographed on silica gel with hexane–EtOAc mixtures of increasing polarity (19.8:0.2 → 10.2:9.8). Compound **39** (344 mg, 57.1%) was obtained as a colorless solid; m.p. = 148.9–149.7 °C (AcOEt/hex.). IR_νmax_ (KBr, cm^−1^): 2946 and 2913 (C-H); 2871 and 2707 (CHO); 1713 (C=O); 1460 (CH_2_-); 1390 (CH_3_-); 1261, 1241 and 1167 (C-O). ^1^H NMR (400.1 MHz, CDCl_3_) δ (ppm): 9.54 (1H, d, *J* = 3.3 Hz, H-22); 3.96–3.86 (7H, m, dioxolane); 3.76–3.71 (1H, m, dioxolane); 2.38–2.29 (1H, m, H-20); 1.91 (1H, dt, *J* = 12.5 and 3.3 Hz, H-12α); 1.87–1.79 (1H, m, H-16); 1.17–0.997 (3H, m, H-15, H-7 and H-5); 1.10 (3H, d, *J* = 7.0 Hz, H-21); 0.934 (3H, s, H-19); 0.797 (1H, dd, *J* = 11.3 and 4.0 Hz, H-17); 0.706 (3H, s, H-18) [18]. ^13^C NMR (100.6 MHz, CDCl_3_), δ (ppm): 205.08 (C-22); 109.71 and 109.54 (C-3 and C-6); 65.38; 64.24; 64.16 and 64.07 (dioxolane); 55.22 (C-5); 53.25 (C-17); 50.95 (C-14); 49.51 (C-9); 49.43 (C-20); 43.19 (C-13); 41.14 (C-7); 39.44 (C-12); 37.19 (C-1); 36.81 (C-10); 33.34 (C-8); 30.98 (C-2); 29.19 (C-4); 26.94 (C-16); 24.50 (C-15); 20.93 (C-11); 13.49 (C-19); 13.38 (C-21); 12.39 (C-18) [18] (Figures S21–S25, Supplementary Material).

#### 3.2.7. 22-Hydroxy-23,24-dinor-5α-cholan-3,6-dione (**18**)

Compound **39** (690 mg, 1.60 mmol) is dissolved in THF (15 mL) and MeOH (30 mL) with slow stirring. Then, NaBH_4_ (40 mg, 1.06 mmol) is added in two portions (each 1 h). The reaction reduction is verified by TLC (2 h). Later, acetone (5 mL) and acid water (pH = 3) (15 mL) are added, and the solvents are evaporated under reduced pressure. The resulting residue is diluted with EtOAc (30 mL), washed with water (2 × 10 mL), and dried over MgSO_4_. Subsequently, the compound is dissolved in acetone (60 mL) and hydrochloric acid (37%) (1 mL), and H_2_O (10 mL) is added at 50 °C. The end of the reaction is verified by TLC (3 h). Then, acetone is evaporated under reduced pressure. The resulting compound is diluted with CH_2_Cl_2_ (10 mL), washed with water (2 × 15 mL), and dried over MgSO_4_. Finally, the solvent is evaporated in vacuo. Compound **18** (445 mg, 80.5%) is obtained as a colorless solid; m.p. = 172.4–174.1 °C (CH_2_Cl_2_). IR_νmax_ (KBr, cm^−1^): 3571–3200 (OH); 2972, 2940, 2905 and 2861 (CH-); 1714 (C=O); 1460 (CH_2_-); 1385 (CH_3_-); 1270 and 1239 (C-O). ^1^H NMR (400.1 MHz, CDCl_3_) δ (ppm): 3.63 (1H, dd, *J* = 10.6 and 3.2 Hz, H-22a); 3.38 (1H, dd, *J* = 10.6 and 6.6 Hz, H-22b); 2.63–2.54 (2H, m, H-5 and H-2); 2.00 (1H, dd, *J* = 13.2 and 12.7 Hz, H-7α); 1.45 (1H, dd, *J* = 12.7 and 3.7 Hz, H-11b); 1.14 (1H, ddd, *J* = 17.7, 11.9 and 6.0 Hz, H-15); 1.05 (3H, d, *J* = 6.6 Hz, H-21); 0.952 (3H, s, H-19); 0.706 (3H, s, H-18). ^13^C NMR (100.6 MHz, CDCl_3_) δ (ppm): 211.25 (C-3); 209.02 (C-6); 67.76 (C-22); 57.46 (C-5); 56.30 (C-14); 53.38 (C-9); 52.29 (C-17); 46.55 (C-7); 43.04 (C-13); 41.20 (C-10); 39.17 (C-12); 38.56 (C-20); 38.04 (C-1); 37.99 (C-8); 37.34 (C-2); 36.95 (C-4); 27.49 (C-16); 24.06 (C-15); 21.61 (C-11); 16.69 (C-21); 12.53 (C-19); 12.06 (C-18) (Figures S26–S30, Supplementary Material). HRMS-ESI (positive mode): *m*/*z* calculated for C_22_H_34_O_3_: 347.2581 [M + H]^+^; found 347.2590 [M + H]^+^ (Figure S106, Supplementary Material).

#### 3.2.8. 3,6-Dioxo-23,24-dinor-5α-cholan-(4-substituted or 2-substituted)-benzoate-22-yl (**19**–**33**)

General procedure: Compound **18** is dissolved in CH_2_Cl_2_ (20 mL) with slow stirring, and after that, pyridine (py, 0.3 mL), 4-dimetilaminopyiridine (DMAP) (15 mg), and *p*-PhCOCl or *o*-PhCOCl are added at room temperature. The end of the reaction is verified by TLC (3 h). Then, 5 mL of acid water (pH = 4) is added and stirred for 30 min. The crude is extracted with CH_2_Cl_2_ (2 × 20 mL) and washed with acid water (3 × 25 mL) until pH = 4. The organic layer is dried over MgSO_4_ and filtered, and the solvent is evaporated under reduced pressure. The crude is redissolved in CH_2_Cl_2_ (5–10 mL) and chromatographed on silica gel with hexane–EtOAc mixtures of increasing polarity. Finally, the selected fractions are chromatographed on Sephadex LH-20 with hexane:CH_2_Cl_2_:MeOH (2:1:1).

##### 3,6-Dioxo-23,24-dinor-5α-cholan-benzoate-22-yl (**19**)

Compound **19** is obtained by using the general procedure described in 3.2.8 and under the following conditions: **18** (90 mg, 0.26 mmol), CH_2_Cl_2_ (20 mL), py (0.3 mL), DMAP (15 mg) and PhCOCl (0.3 mL, 2.58 mmol, d = 1.21 g/mL). Compound **19** (52 mg, 44.4% yield) is obtained as a colorless solid; m.p. = 208.0–209.1 °C (EtOAc/hex.). IR_νmax_ (KBr, cm^−1^): 2945, 2900 and 2868 (CH-); 1716 (C=O); 1449 (CH_2_-); 1393 (CH_3_-); 1277 and 1108 (C-O); 718 (CH=CH). ^1^H NMR (400.1 MHz, CDCl_3_) δ (ppm): 8.04 (2H, d, *J* = 8.3 Hz, H-2′ and H-6′); 7.57 (1H, t, *J* = 7.4 Hz, H-4′); 7.45 (2H, d, *J* = 7.4 Hz, H-3′ and H-5′); 4.32 (1H, dd, *J* = 10.8 and 3.4 Hz, H-22a); 4.07 (1H, dd, *J* = 10.8 and 6.9 Hz, H-22b); 2.63–2.57 (2H, m, H-5 and H-2); 2.02 (1H, dd, *J* = 13.0 and 12.6 Hz, H-7α); 1.16 (1H, ddd, *J* = 17.8, 11.9 and 5.9 Hz, H-15); 1.13 (3H, d, *J* = 6.6 Hz, H-21); 0.964 (3H, s, H-19); 0.751 (3H, s, H-18). ^13^C NMR (100.6 MHz, CDCl_3_) δ (ppm): 211.20 (C-3); 208.91 (C-6); 166.70 (Ar-CO_2_); 132.86 (C-4′); 130.46 (C-1′); 129.49 (C-2′ and C-6′); 128.36 (C-3′ and C-5′); 69.77 (C-22); 57.48 (C-5); 56.26 (C-14); 53.39 (C-9); 52.82 (C-17); 46.52 (C-7); 43.17 (C-13); 41.19 (C-10); 39.20 (C-12); 38.07 (C-1); 37.98 (C-8); 37.39 (C-2); 36.96 (C-4); 35.94 (C-20); 27.54 (C-16); 24.07 (C-15); 21.64 (C-11); 17.31 (C-21); 12.55 (C-19); 12.08 (C-18) (Figures S31–S35, Supplementary Material). HRMS-ESI (positive mode): *m*/*z* calculated for C_29_H_38_O_4_: 451.2843 [M + H]^+^; found 451.2847 [M + H]^+^ (Figure S107, Supplementary Material).

##### 3,6-Dioxo-23,24-dinor-5α-cholan-(4-methyl)-benzoate-22-yl (**20**)

Compound **20** is obtained by using the general procedure described in 3.2.8 and under the following conditions: **18** (100 mg, 0.29 mmol), CH_2_Cl_2_ (20 mL), py (0.2 mL), DMAP (15 mg) and *p*-CH_3_-PhCOCl (0.7 mL, 5.30 mmol, d = 1.17 g/mL). Compound **20** (67 mg, 50.0% yield) is obtained as a colorless solid; m.p. = 199.3–200.9 °C (EtOAc/hex.). IR_νmax_ (KBr, cm^−1^): 2947 and 2868 (CH-); 1714 (C=O); 1610 (C=C); 1274 and 1178 (C-O); 755 (CH=CH). ^1^H NMR (400.1 MHz, CDCl_3_) δ (ppm): 7.92 (2H, d, *J* = 8.2 Hz, H-2′ and H-6′); 7.24 (2H, d, *J* = 8.2 Hz, H-3′ and H-5′); 4.29 (1H, dd, *J* = 10.7 and 3.4 Hz, H-22a); 4.04 (1H, dd, *J* = 10.7 and 7.1 Hz, H-22b); 2.61–2.57 (2H, m, H-5 and H-2); 2.41 (3H, s, Ar-CH_3_); 2.01 (1H, t, *J* = 12.7 Hz, H-7α); 1.12 (3H, d, *J* = 6.6 Hz, H-21); 0.962 (3H, s, H-19); 0.746 (3H, s, H-18). ^13^C NMR (100.6 MHz, CDCl_3_) δ (ppm): 211.18 (C-3); 208.91 (C-6); 166.75 (Ar-CO_2_); 143.50 (C-4′); 129.50 (C-3′ and C-5′); 129.05 (C-2′ and C-6′); 127.71 (C-1′); 69.55 (C-22); 57.46 (C-5); 56.24 (C-14); 53.36 (C-9); 52.82 (C-17); 46.50 (C-7); 43.15 (C-13); 41.17 (C-10); 39.18 (C-12); 38.05 (C-1); 37.95 (C-8); 37.33 (C-2); 36.94 (C-4); 35.93 (C-20); 27.52 (C-16); 24.06 (C-15); 21.63 (Ar-CH_3_); 21.61 (C-11); 17.29 (C-21); 12.53 (C-19); 12.06 (C-18) (Figures S36–S40, Supplementary Material). HRMS-ESI (positive mode): *m*/*z* calculated for C_30_H_40_O_4_: 465.2999 [M + H]^+^; found 465.3012 [M + H]^+^ (Figure S108, Supplementary Material).

##### 3,6-Dioxo-23,24-dinor-5α-cholan-(4-methoxy)-benzoate-22-yl (**21**)

Compound **21** is obtained by using the general procedure described in 3.2.8 and under the following conditions: **18** (149 mg, 0.43 mmol), CH_2_Cl_2_ (25 mL), py (0.3 mL), DMAP (15 mg) and *p*-OCH_3_-PhCOCl (970 mg, 5.67 mmol). Compound **21** (38 mg, 18.4% yield) is obtained as a colorless solid; m.p. = 206.2–207.6 °C (EtOAc/hex.). IR_νmax_ (KBr, cm^−1^): 2947, 2894 and 2862 (CH-); 1709 (C=O); 1604 and 1511 (C=C); 1259 and 1164 (C-O); 852 (CH=CH). ^1^H NMR (400.1 MHz, CDCl_3_) δ (ppm): 7.98 (2H, d, *J* = 8.9 Hz, H-2′ and H-6′); 6.92 (2H, d, *J* = 8.9 Hz, H-3′ and H-5′); 4.27 (1H, dd, *J* = 10.7 and 3.4 Hz, H-22a); 4.02 (1H, dd, *J* = 10.7 and 7.1 Hz, H-22b); 3.85 (3H, s, Ar-OCH_3_); 2.60–2.56 (2H, m, H-5 and H-2); 2.00 (1H, t, *J* = 12.7 Hz, H-7α); 1.11 (3H, d, *J* = 6.6 Hz, H-21); 0.952 (3H, s, H-19); 0.737 (3H, s, H-18). ^13^C NMR (100.6 MHz, CDCl_3_) δ (ppm): 211.24 (C-3); 208.95 (C-6); 166.45 (Ar-CO_2_); 163.26 (C-4′); 131.47 (C-2′ and C-6′); 122.85 (C-1′); 113.57 (C-3′ and C-5′); 69.44 (C-22); 57.43 (C-5); 56.23 (C-14); 55.39 (Ar-OCH_3_); 53.35 (C-9); 52.83 (C-17); 46.48 (C-7); 43.13 (C-13); 41.16 (C-10); 39.17 (C-12); 38.02 (C-1); 37.94 (C-8); 37.31 (C-2); 36.92 (C-4); 35.92 (C-20); 27.50 (C-16); 24.04 (C-15); 21.60 (C-11); 17.29 (C-21); 12.51 (C-19); 12.04 (C-18) (Figures S41–S45, Supplementary Material). HRMS-ESI (positive mode): *m*/*z* calculated for C_30_H_40_O_5_: 481.2949 [M + H]^+^; found 481.2951 [M + H]^+^ (Figure S109, Supplementary Material).

##### 3,6-Dioxo-23,24-dinor-5α-cholan-(4-fluoro)-benzoate-22-yl (**22**)

Compound **22** is obtained by using the general procedure described in 3.2.8 and under the following conditions: **18** (100 mg, 0.29 mmol), CH_2_Cl_2_ (25 mL), py (0.3 mL), DMAP (15 mg) and *p*-F-PhCOCl (0.3 mL, 2.54 mmol, d = 1.34 g/mL). Compound **22** (80 mg, 59.2% yield) is obtained as a colorless solid; m.p. = 197.3–198.5 °C (CH_2_Cl_2_). IR_νmax_ (KBr, cm^−1^): 2965, 2944 and 2865 (CH-); 1716 (C=O); 1604 (C=C); 1281 and 1155 (C-O); 763 (CH=CH). ^1^H NMR (400.1 MHz, CDCl_3_) δ (ppm): 8.04 (2H, dd, *J* = 8.7 and 5.5 Hz, H-2′ and H-6′); 7.11 (2H, t, *J* = 8.7 Hz, H-3′ and H-5′); 4.30 (1H, dd, *J* = 10.7 and 3.4 Hz, H-22a); 4.04 (1H, dd, *J* = 10.7 and 7.1 Hz, H-22b); 2.61–2.56 (2H, m, H-5 and H-2); 2.01 (1H, t, *J* = 12.7 Hz, H-7α); 1.11 (3H, d, *J* = 6.6 Hz, H-21); 0.955 (3H, s, H-19); 0.740 (3H, s, H-18). ^13^C NMR (100.6 MHz, CDCl_3_) δ (ppm): 211.16 (C-3); 208.87 (C-6); 165.70 (Ar-CO_2_); 165.67 (d, ^1^*J*_C-F_ = 253.6 Hz, C-4′); 131.98 (d, ^3^*J*_C-F_ = 10.1 Hz, C-2′ and C-6′); 126.66 (d, ^4^*J*_C-F_ = 3.0 Hz, C-1′); 115.47 (d, ^2^*J*_C-F_ = 22.1 Hz, C-3′ and C-5′); 69.87 (C-22); 57.44 (C-5); 56.22 (C-14); 53.34 (C-9); 52.79 (C-17); 46.47 (C-7); 43.15 (C-13); 41.15 (C-10); 39.17 (C-12); 38.03 (C-1); 37.93 (C-8); 37.31 (C-2); 36.92 (C-4); 35.89 (C-20); 27.51 (C-16); 24.04 (C-15); 21.60 (C-11); 17.27 (C-21); 12.51 (C-19); 12.05 (C-18) (Figures S46–S50, Supplementary Material). HRMS-ESI (positive mode): *m*/*z* calculated for C_29_H_37_FO_4_: 469.2749 [M + H]^+^; found 469.2755 [M + H]^+^ (Figure S110, Supplementary Material).

##### 3,6-Dioxo-23,24-dinor-5α-cholan-(4-chloro)-benzoate-22-yl (**23**)

Compound **23** is obtained by using the general procedure described in 3.2.8 and under the following conditions: **18** (92 mg, 0.27 mmol), CH_2_Cl_2_ (20 mL), py (0.2 mL), DMAP (15 mg) and *p*-Cl-PhCOCl (0.6 mL, 4.70 mmol, d = 1.37 g/mL). Compound **23** (37 mg, 28.7% yield) is obtained as a colorless solid; m.p. = 210.1–211.5 °C (CH_2_Cl_2_). IR_νmax_ (KBr, cm^−1^): 2946 (CH-); 1715 (C=O); 1592 (C=C); 1274 and 1116 (C-O); 762 (CH=CH). ^1^H NMR (400.1 MHz, CDCl_3_) δ (ppm): 7.97 (2H, d, *J* = 8.6 Hz, H-2′ and H-6′); 7.42 (2H, d, *J* = 8.6 Hz, H-3′ and H-5′); 4.31 (1H, dd, *J* = 10.7 and 3.5 Hz, H-22a); 4.05 (1H, dd, *J* = 10.8 and 7.2 Hz, H-22b); 2.61–2.58 (2H, m, H-5 and H-2); 2.01 (1H, t, *J* = 12.7 Hz, H-7α); 1.12 (3H, d, *J* = 6.6 Hz, H-21); 0.964 (3H, s, H-19); 0.748 (3H, s, H-18). ^13^C NMR (100.6 MHz, CDCl_3_) δ (ppm): 211.19 (C-3); 208.90 (C-6); 165.83 (Ar-CO_2_); 139.31 (C-4′); 130.88 (C-2′ and C-6′); 128.87 (C-1′); 128.72 (C-3′ and C-5′); 70.01 (C-22); 57.48 (C-5); 56.25 (C-14); 53.37 (C-9); 52.81 (C-17); 46.50 (C-7); 43.18 (C-13); 41.18 (C-10); 39.19 (C-12); 38.06 (C-1); 37.96 (C-8); 37.34 (C-2); 36.95 (C-4); 35.91 (C-20); 27.54 (C-16); 24.06 (C-15); 21.62 (C-11); 17.28 (C-21); 12.54 (C-19); 12.07 (C-18) (Figures S51–S55, Supplementary Material). HRMS-ESI (positive mode): *m*/*z* calculated for C_29_H_37_ClO_4_: 485.2453 [M + H]^+^; found 485.2440 [M + H]^+^ (Figure S111, Supplementary Material).

##### 3,6-Dioxo-23,24-dinor-5α-cholan-(4-bromo)-benzoate-22-yl (**24**)

Compound **24** is obtained by using the general procedure described in 3.2.8 and under the following conditions: **18** (100 mg, 0.29 mmol), CH_2_Cl_2_ (25 mL), py (0.3 mL), DMAP (15 mg) and *p*-Br-PhCOCl (380 mg, 1.73 mmol). Compound **24** (90 mg, 58.9% yield) is obtained as a colorless solid; m.p. = 224.1–225.8 °C (CH_2_Cl_2_). IR_νmax_ (KBr, cm^−1^): 2951, 2899 and 2866 (CH-); 1714 (C=O); 1588 (C=C); 1271 and 1175 (C-O); 761 (CH=CH). ^1^H NMR (400.1 MHz, CDCl_3_) δ (ppm): 7.89 (2H, d, *J* = 8.6 Hz, H-2′ and H-6′); 7.58 (2H, d, *J* = 8.6 Hz, H-3′ and H-5′); 4.31 (1H, dd, *J* = 10.8 and 3.5 Hz, H-22a); 4.05 (1H, dd, *J* = 10.8 and 7.2 Hz, H-22b); 2.61–2.57 (2H, m, H-5 and H-2); 2.01 (1H, t, *J* = 12.7 Hz, H-7α); 1.11 (3H, d, *J* = 6.6 Hz, H-21); 0.963 (3H, s, H-19); 0.747 (3H, s, H-18). ^13^C NMR (100.6 MHz, CDCl_3_) δ (ppm): 211.17 (C-3); 208.88 (C-6); 165.95 (Ar-CO_2_); 131.71 (C-2′ and C-6′); 131.03 (C-3′ and C-5′); 129.34 (C-1′); 127.97 (C-4′); 70.03 (C-22); 57.47 (C-5); 56.25 (C-14); 53.37 (C-9); 52.81 (C-17); 46.50 (C-7); 43.19 (C-13); 41.18 (C-10); 39.19 (C-12); 38.06 (C-1); 37.96 (C-8); 37.34 (C-2); 36.95 (C-4); 35.90 (C-20); 27.54 (C-16); 24.06 (C-15); 21.62 (C-11); 17.28 (C-21); 12.54 (C-19); 12.08 (C-18) (Figures S56–S60, Supplementary Material). HRMS-ESI (positive mode): *m*/*z* calculated for C_29_H_37_BrO_4_: 531.1932 [M + H]^+^; found 531.1940 [M + H]^+^ (Figure S112, Supplementary Material).

##### 3,6-Dioxo-23,24-dinor-5α-cholan-(4-iodo)-benzoate-22-yl (**25**)

Compound **25** is obtained by using the general procedure described in 3.2.8 and under the following conditions: **18** (100 mg, 0.29 mmol), CH_2_Cl2 (25 mL), py (0.3 mL), DMAP (15 mg) and *p*-I-PhCOCl (370 mg, 1.39 mmol). Compound **25** (45 mg, 27.0% yield) is obtained as a colorless solid; m.p. = 245.6–247.6 °C (CH_2_Cl_2_). IR_νmax_ (KBr, cm^−1^): 2949, 2899 and 2865 (CH-); 1712 (C=O); 1583 (C=C); 1283, 1266 and 1180 (C-O); 759 (CH=CH). ^1^H NMR (400.1 MHz, CDCl_3_) δ (ppm): 7.80 (2H, d, *J* = 8.4 Hz, H-3′ and H-5′); 7.73 (2H, d, *J* = 8.4 Hz, H-2′ and H-6′); 4.30 (1H, dd, *J* = 10.8 and 3.3 Hz, H-22a); 4.05 (1H, dd, *J* = 10.8 and 7.2 Hz, H-22b); 2.62–2.56 (2H, m, H-5 and H-2); 2.41 (3H, s, Ar-CH_3_); 2.01 (1H, t, *J* = 12.7 Hz, H-7α); 1.11 (3H, d, *J* = 6.6 Hz, H-21); 0.960 (3H, s, H-19); 0.743 (3H, s, H-18). ^13^C NMR (100.6 MHz, CDCl_3_) δ (ppm): 211.14 (C-3); 208.86 (C-6); 166.17 (Ar-CO_2_); 137.72 (C-3′ and C-5′); 130.94 (C-2′ and C-6′); 129.90 (C-1′); 100.61 (C-4′); 70.01 (C-22); 57.46 (C-5); 56.24 (C-14); 53.36 (C-9); 52.80 (C-17); 46.49 (C-7); 43.17 (C-13); 41.17 (C-10); 39.19 (C-12); 38.05 (C-1); 37.94 (C-8); 37.33 (C-2); 36.94 (C-4); 35.89 (C-20); 27.53 (C-16); 24.05 (C-15); 21.61 (C-11); 17.27 (C-21); 12.53 (C-19); 12.07 (C-18) (Figures S61–S65, Supplementary Material). HRMS-ESI (positive mode): *m*/*z* calculated for C_29_H_37_IO_4_: 577.1809 [M + H]^+^; found 577.1800 [M + H]^+^ (Figure S113, Supplementary Material).

##### 3,6-Dioxo-23,24-dinor-5α-cholan-(4-acetoxy)-benzoate-22-yl (**26**)

Compound **26** is obtained by using the general procedure described in 3.2.8 and under the following conditions: **18** (110 mg, 0.32 mmol), CH_2_Cl_2_ (25mL), py (0.5 mL), DMAP (23 mg) and *p*-CH_3_COO-PhCOCl (300 mg, 1.52 mmol). Compound **26** (52 mg, 63.8% yield) is obtained as a colorless solid; m.p. = 179.5–180.8 °C (CH_2_Cl_2_). IR_νmax_ (KBr, cm^−1^): 2966, 2944 and 2865 (CH-); 1763 and 1715 (C=O); 1603 (C=C); 1276, 1192, 1159 and 1113 (C-O); 763 (CH=CH). ^1^H NMR (400.1 MHz, CDCl_3_) δ (ppm): 8.06 (2H, d, *J* = 8.8 Hz, H-2′ and H-6′); 7.17 (2H, d, *J* = 8.8 Hz, H-3′ and H-5′); 4.30 (1H, dd, *J* = 10.7 and 3.4 Hz, H-22a); 4.07 (1H, dd, *J* = 10.7 and 7.0 Hz, H-22b); 2.61–2.57 (2H, m, H-5 and H-2); 2.32 (3H, s, COCH3); 2.01 (1H, t, J = 12.8 Hz, H-7α); 1.12 (3H, d, *J* = 6.6 Hz, H-21); 0.960 (3H, s, H-19); 0.744 (3H, s, H-18). ^13^C NMR (100.6 MHz, CDCl_3_) δ (ppm): 211.17 (C-3); 208.90 (C-6); 168.90 (COCH_3_); 165.85 (Ar-CO_2_); 154.21 (C-4′); 131.07 (C-2′ and C-6′); 128.02 (C-1′); 121.60 (C-3′ and C-5′); 69.87 (C-22); 57.46 (C-5); 56.24 (C-14); 53.37 (C-9); 52.78 (C-17); 46.50 (C-7); 43.15 (C-13); 41.18 (C-10); 39.18 (C-12); 38.05 (C-1); 37.96 (C-8); 37.33 (C-2); 36.94 (C-4); 35.91 (C-20); 27.52 (C-16); 24.05 (C-15); 21.62 (C-11); 21.13 (COCH_3_); 17.29 (C-21); 12.53 (C-19); 12.07 (C-18) (Figures S66–S70, Supplementary Material). HRMS-ESI (positive mode): *m*/*z* calculated for C_31_H_40_O_6_: 509.2898 [M + H]^+^; found 509.2897 [M + H]^+^ (Figure S114, Supplementary Material).

##### 3,6-Dioxo-23,24-dinor-5α-cholan-(2-methyl)-benzoate-22-yl (**27**)

Compound **27** is obtained by using the general procedure described in 3.2.8 and under the following conditions: **18** (100 mg, 0.29 mmol), CH_2_Cl_2_ (25 mL), py (0.3 mL), DMAP (15 mg) and *o*-CH_3_-PhCOCl (0.5 mL, 3.85 mmol, d = 1.19 g/mL). Compound **27** (54 mg, 40.3% yield) is obtained as a colorless solid; m.p. = 203.2–205.5 °C (CH_2_Cl_2_). IR_νmax_ (KBr, cm^−1^): 2932 and 2854 (CH-); 1714 (C=O); 1264 and 1130 (C-O); 741 (CH=CH). ^1^H NMR (400.1 MHz, CDCl_3_) δ (ppm): 7.91 (1H, d, *J* = 8.4 and 1.5 Hz, H-6′); 7.40 (1H, ddd, *J* = 7.5, 7.5 and 1.3 Hz, H-4′); 7.27–7.23 (2H, m, H-5′ and H-3′); 4.30 (1H, dd, *J* = 10.8 and 3.2 Hz, H-22a); 4.04 (1H, dd, *J* = 10.8 and 7.0 Hz, H-22b); 2.62–2.54 (2H, m, H-5 and H-2); 2.61 (3H, s, CH_3_-Ar); 2.01 (1H, dd, *J* = 12.7 and 12.7 Hz, H-7α); 1.19–1.15 (1H, m, H-15); 1.13 (3H, d, *J* = 6.6 Hz, H-21); 0.962 (3H, s, H-19); 0.747 (3H, s, H-18). ^13^C NMR (100.6 MHz, CDCl_3_) δ (ppm): 211.16 (C-3); 208.89 (C-6); 167.76 (Ar-CO_2_); 140.11 (C-2′); 131.88 (C-4′); 131.70 (C-5′); 130.48 (C-6′); 129.85 (C-1′); 125.68 (C-3′); 69.65 (C-22); 57.47 (C-5); 56.28 (C-14); 53.37 (C-9); 52.76 (C-17); 46.51 (C-7); 43.13 (C-13); 41.18 (C-10); 39.20 (C-12); 38.06 (C-1); 37.97 (C-8); 37.34 (C-2); 36.95 (C-4); 35.87 (C-20); 27.54 (C-16); 24.05 (C-15); 21.85 (CH_3_-Ar); 21.63 (C-11); 17.44 (C-21); 12.53 (C-19); 12.08 (C-18) (Figures S71–S75, Supplementary Material). HRMS-ESI (positive mode): *m*/*z* calculated for C_30_H_40_O_4_: 465.2999 [M + H]^+^; found 465.3000 [M + H]^+^ (Figure S115, Supplementary Material).

##### 3,6-Dioxo-23,24-dinor-5α-cholan-(2-methoxy)-benzoate-22-yl (**28**)

Compound **28** is obtained by using the general procedure described in 3.2.8 and under the following conditions: **18** (100 mg, 0.29 mmol), CH_2_Cl_2_ (25 mL), py (0.3 mL), DMAP (15 mg) and *o*-CH_3_O-PhCOCl (0.4 mL, 2.70 mmol, d = 1.15 g/mL). Compound **28** (88 mg, 63.4% yield) is obtained as a colorless solid; m.p. = 182.4–183.4 °C (CH_2_Cl_2_). IR_νmax_ (KBr, cm^−1^): 2948 and 2866 (CH-); 1712 (C=O); 1598 and 1582 (C=C); 1253 and 1167 (C-O); 757 (CH=CH). ^1^H NMR (400.1 MHz, CDCl_3_) δ (ppm): 7.92 (1H, d, *J* = 7.9 and 1.9 Hz, H-6′); 7.47 (1H, ddd, *J* = 7.5, 7.5 and 1.8 Hz, H-4′); 7.00–6.96 (2H, m, H-5′ and H-3′); 4.30 (1H, dd, *J* = 10.8 and 3.3 Hz, H-22a); 4.02 (1H, dd, *J* = 10.8 and 7.1 Hz, H-22b); 3.90 (3H, s, OCH_3_); 2.62–2.54 (2H, m, H-5 and H-2); 2.01 (1H, dd, *J* = 12.5 and 12.5 Hz, H-7α); 1.20–1.15 (1H, m, H-15); 1.12 (3H, d, *J* = 6.6 Hz, H-21); 0.957 (3H, s, H-19); 0.735 (3H, s, H-18). ^13^C NMR (100.6 MHz, CDCl_3_) δ (ppm): 211.18 (C-3); 208.90 (C-6); 167.39 (Ar-CO_2_); 159.39 (C-2′); 133.41 (C-4′); 131.54 (C-6′); 120.31 (C-1′); 120.06 (C-5′); 111.97 (C-3′); 69.65 (C-22); 57.46 (C-5); 56.30 (C-14); 55.86 (OCH_3_); 53.38 (C-9); 52.71 (C-17); 46.51 (C-7); 43.12 (C-13); 41.17 (C-10); 39.20 (C-12); 38.04 (C-1); 37.96 (C-8); 37.33 (C-2); 36.94 (C-4); 35.86 (C-20); 27.46 (C-16); 24.06 (C-15); 21.62 (C-11); 17.26 (C-21); 12.52 (C-19); 12.07 (C-18) (Figures S76–S80, Supplementary Material). HRMS-ESI (positive mode): *m*/*z* calculated for C_30_H_40_O_5_: 481.2949 [M + H]^+^; found 481.2942 [M + H]^+^ (Figure S116, Supplementary Material).

##### 3,6-Dioxo-23,24-dinor-5α-cholan-(2-fluoro)-benzoate-22-yl (**29**)

Compound **29** is obtained by using the general procedure described in 3.2.8 and under the following conditions: **18** (100 mg, 0.29 mmol), CH_2_Cl_2_ (25 mL), py (0.3 mL), DMAP (15 mg) and *o*-F-PhCOCl (0.2 mL, 1.68 mmol, d = 1.33 g/mL). Compound **29** (64 mg, 47.3% yield) is obtained as a colorless solid; m.p. = 222.3–224.1 °C (CH_2_Cl_2_). IR_νmax_ (KBr, cm^−1^): 2953 and 2867 (CH-); 1718 (C=O); 1612 (C=C); 1259 and 1159 (C-O); 769 (CH=CH). ^1^H NMR (400.1 MHz, CDCl_3_) δ (ppm): 7.94 (1H, dd, *J* = 7.6 and 1.7 Hz, H-4′); 7.55–7.49 (1H, m, H-5′); 7.21 (1H, t, *J* = 7.6 Hz, H-6′); 7.13 (1H, dd, *J* = 10.6, and 8.6 Hz, H-3′); 4.33 (1H, dd, *J* = 10.7 and 3.1 Hz, H-22a); 4.08 (1H, dd, *J* = 10.7 and 6.9 Hz, H-22b); 2.62–2.54 (2H, m, H-5 and H-2); 2.01 (1H, dd, *J* = 12.7 and 12.7 Hz, H-7α); 1.20–1.16 (1H, m, H-15); 1.13 (3H, d, *J* = 6.9 Hz, H-21); 0.960 (3H, s, H-19); 0.742 (3H, s, H-18). ^13^C NMR (100.6 MHz, CDCl_3_) δ (ppm): 211.19 (C-3); 208.91 (C-6); 164.71 (d, ^3^*J*_C-F_ = 3.9 Hz, Ar-CO_2_); 161.93 (d, ^1^*J*_C-F_ = 259.8 Hz, C-2′); 134.36 (d, ^3^*J*_C-F_ = 8.8 Hz, C-4′); 132.09 (C-5′); 123.93 (d, ^3^*J*_C-F_ = 3.8 Hz, C-6′); 118.93 (d, ^2^*J*_C-F_ = 9.9 Hz, C-1′); 116.97 (d, ^2^*J*_C-F_ = 22.8 Hz, C-3′); 70.20 (C-22); 57.47 (C-5); 56.26 (C-14); 53.37 (C-9); 52.56 (C-17); 46.51 (C-7); 43.11 (C-13); 41.18 (C-10); 39.16 (C-12); 38.06 (C-1); 37.97 (C-8); 37.34 (C-2); 36.95 (C-4); 35.81 (C-20); 27.47 (C-16); 24.04 (C-15); 21.62 (C-11); 17.23 (C-21); 12.53 (C-19); 12.06 (C-18) (Figures S81–S85, Supplementary Material). HRMS-ESI (positive mode): *m*/*z* calculated for C_29_H_37_FO_4_: 469.2749 [M + H]^+^; found 469.2745 [M + H]^+^ (Figure S117, Supplementary Material).

##### 3,6-Dioxo-23,24-dinor-5α-cholan-(2-chloro)-benzoate-22-yl (**30**)

Compound **30** is obtained by using the general procedure described in 3.2.8 and under the following conditions: **18** (100 mg, 0.29 mmol), CH_2_Cl_2_ (25 mL), py (0.3 mL), DMAP (15 mg) and *o*-Cl-PhCOCl (0.2 mL, 1.58 mmol, d = 1.38 g/mL). Compound **30** (64 mg, 45.7% yield) is obtained as a colorless solid; m.p. = 204.8–206.2 °C (CH_2_Cl_2_). IR_νmax_ (KBr, cm^−1^): 2945 and 2904 (CH-); 1713 (C=O); 1595 (C=C); 1307 and 1137 (C-O); 763 (CH=CH). ^1^H NMR (400.1 MHz, CDCl_3_) δ (ppm): 7.83 (1H, dd, *J* = 7.7 and 1.6 Hz, H-6′); 7.46 (1H, dd, *J* = 8.2 and 1.3 Hz, H-3′); 7.42 (1H, ddd, *J* = 8.2, 7.0 and 1.6 Hz, H-4′); 7.32 (1H, ddd, *J* = 7.5, 7.3 and 1.6 Hz, H-5′); 4.35 (1H, dd, *J* = 10.8 and 3.3 Hz, H-22a); 4.09 (1H, dd, *J* = 10.8 and 7.0 Hz, H-22b); 2.63–2.54 (2H, m, H-5 and H-2); 2.01 (1H, dd, *J* = 12.8 and 12.8 Hz, H-7α); 1.21–1.16 (1H, m, H-15); 1.14 (3H, d, *J* = 6.6 Hz, H-21); 0.961 (3H, s, H-19); 0.742 (3H, s, H-18). ^13^C NMR (100.6 MHz, CDCl_3_) δ (ppm): 211.18 (C-3); 208.90 (C-6); 166.01 (Ar-CO_2_); 133.58 (C-2′); 132.45 (C-4′); 131.41 (C-6′); 131.08 (C-3′); 130.42 (C-1′); 126.57 (C-5′); 70.54 (C-22); 57.48 (C-5); 56.28 (C-14); 53.37 (C-9); 52.57 (C-17); 46.51 (C-7); 43.13 (C-13); 41.18 (C-10); 39.18 (C-12); 38.06 (C-1); 37.97 (C-8); 37.34 (C-2); 36.95 (C-4); 35.83 (C-20); 27.52 (C-16); 24.05 (C-15); 21.63 (C-11); 17.38 (C-21); 12.54 (C-19); 12.07 (C-18) (Figures S86–S90, Supplementary Material). HRMS-ESI (positive mode): *m*/*z* calculated for C_29_H_37_ClO_4_: 485.2453 [M + H]^+^; found 485.2451 [M + H]^+^ (Figure S118, Supplementary Material).

##### 3,6-Dioxo-23,24-dinor-5α-cholan-(2-bromo)-benzoate-22-yl (**31**)

Compound **31** is obtained by using the general procedure described in 3.2.8 and under the following conditions: **18** (130 mg, 0.38 mmol), CH_2_Cl_2_ (25 mL), py (0.3 mL), DMAP (15 mg) and *o*-Br-PhCOCl (0.4 mL, 3.06 mmol, d = 1.68 g/mL). Compound **31** (104 mg, 52.4% yield) is obtained as a colorless solid; m.p. = 202.5–204.7 °C (CH_2_Cl_2_). IR_νmax_ (KBr, cm^−1^): 2945, 2904 and 2861 (CH-); 1713 (C=O); 1590 (C=C); 1305 and 1134 (C-O); 759 (CH=CH). ^1^H NMR (400.1 MHz, CDCl_3_) δ (ppm): 7.78 (1H, dd, *J* = 7.5 and 2.0 Hz, H-6′); 7.66 (1H, dd, *J* = 7.7 and 1.3 Hz, H-3′); 7.37 (1H, ddd, *J* = 7.7, 7.7 and 1.3 Hz, H-4′); 7.32 (1H, ddd, *J* = 7.5, 7.5 and 2.0 Hz, H-5′); 4.34 (1H, dd, *J* = 10.7 and 3.2 Hz, H-22a); 4.08 (1H, dd, *J* = 10.7 and 6.9 Hz, H-22b); 2.62–2.54 (2H, m, H-5 and H-2); 2.01 (1H, dd, *J* = 13.0 and 13.0 Hz, H-7α); 1.22–1.16 (1H, m, H-15); 1.13 (3H, d, *J* = 6.6 Hz, H-21); 0.957 (3H, s, H-19); 0.738 (3H, s, H-18). ^13^C NMR (100.6 MHz, CDCl_3_) δ (ppm): 211.16 (C-3); 208.88 (C-6); 166.42 (Ar-CO_2_); 134.33 (C-3′); 132.51 (C-1′); 132.44 (C-5′); 131.26 (C-6′); 127.14 (C-4′); 121.51 (C-2′); 70.59 (C-22); 57.45 (C-5); 56.26 (C-14); 53.34 (C-9); 52.56 (C-17); 46.49 (C-7); 43.13 (C-13); 41.16 (C-10); 39.16 (C-12); 38.04 (C-1); 37.95 (C-8); 37.33 (C-2); 36.94 (C-4); 35.81 (C-20); 27.52 (C-16); 24.03 (C-15); 21.61 (C-11); 17.39 (C-21); 12.53 (C-19); 12.06 (C-18) (Figures S91–S95, Supplementary Material). HRMS-ESI (positive mode): *m*/*z* calculated for C_29_H_37_BrO_4_: 531.1932 [M + H]^+^; found 531.1936 [M + H]^+^ (Figure S119, Supplementary Material).

##### 3,6-Dioxo-23,24-dinor-5α-cholan-(2-iodo)-benzoate-22-yl (**32**)

Compound **32** is obtained by using the general procedure described in 3.2.8 and under the following conditions: **18** (100 mg, 0.29 mmol), CH_2_Cl_2_ (25 mL), py (0.3 mL), DMAP (15 mg) and o-*I*-PhCOCl (755 mg, 2.83 mmol). Compound **32** (128 mg, 76.9% yield) is obtained as a colorless solid; m.p. = 202.9–204.1 °C (CH_2_Cl_2_). IR_νmax_ (KBr, cm^−1^): 2943, 2903 and 2861 (CH-); 1713 (C=O); 1584 (C=C); 1300, 1249 and 1130 (C-O); 755 (CH=CH). ^1^H NMR (400.1 MHz, CDCl_3_) δ (ppm): 7.99 (1H, dd, *J* = 8.0 and 0.9 Hz, H-3′); 7.78 (1H, dd, *J* = 7.7 and 1.6 Hz, H-6′); 7.41 (1H, ddd, *J* = 7.7, 7.7 and 1.1 Hz, H-5′); 7.15 (1H, ddd, *J* = 8.0, 7.7 and 1.6 Hz, H-4′); 4.34 (1H, dd, *J* = 10.8 and 3.3 Hz, H-22a); 4.07 (1H, dd, *J* = 10.8 and 7.2 Hz, H-22b); 2.62–2.54 (2H, m, H-5 and H-2); 2.01 (1H, dd, *J* = 12.9 and 12.9 Hz, H-7α); 1.20–1.15 (1H, m, H-15); 1.13 (3H, d, *J* = 6.6 Hz, H-21); 0.955 (3H, s, H-19); 0.737 (3H, s, H-18). ^13^C NMR (100.6 MHz, CDCl_3_) δ (ppm): 211.15 (C-3); 208.88 (C-6); 166.68 (Ar-CO_2_); 141.30 (C-3′); 135.44 (C-1′); 132.52 (C-4′); 130.78 (C-6′); 127.87 (C-5′); 93.96 (C-2′); 70.56 (C-22); 57.44 (C-5); 56.24 (C-14); 53.33 (C-9); 52.61 (C-17); 46.48 (C-7); 43.14 (C-13); 41.16 (C-10); 39.15 (C-12); 38.03 (C-1); 37.94 (C-8); 37.32 (C-2); 36.93 (C-4); 35.81 (C-20); 27.54 (C-16); 24.03 (C-15); 21.60 (C-11); 17.41 (C-21); 12.52 (C-19); 12.07 (C-18) (Figures S96–S100, Supplementary Material). HRMS-ESI (positive mode): *m*/*z* calculated for C_29_H_37_IO_4_: 577.1809 [M + H]^+^; found 577.1815 [M + H]^+^ (Figure S120, Supplementary Material).

##### 3,6-Dioxo-23,24-dinor-5α-cholan-(2-acetoxy)-benzoate-22-yl (**33**)

Compound **33** is obtained by using the general procedure described in 3.2.8 and under the following conditions: **18** (100 mg, 0.29 mmol), CH_2_Cl_2_ (25 mL), py (0.3 mL), DMAP (15 mg) and *o*-CH_3_COO-PhCOCl (260 mg, 1.31 mmol). Compound **33** (82 mg, 55.9% yield) is obtained as a colorless solid; m.p. = 148.2–149.2 °C (CH_2_Cl_2_). IR_νmax_ (KBr, cm^−1^): 2947 and 2867 (CH-); 1770 and 1716 (C=O); 1606 (C=C); 1293, 1259, 1192 and 1078 (C-O); 752 (CH=CH). ^1^H NMR (400.1 MHz, CDCl_3_) δ (ppm): 8.00 (1H, dd, *J* = 7.8 and 1.5 Hz, H-6′); 7.57 (1H, ddd, *J* = 8.0, 7.8 and 1.6 Hz H-4′); 7.32 (1H, ddd, *J* = 7.8, 7.8 and 0.8 Hz, H-5′); 7.11 (1H, ddd, *J* = 8.0, 7.8 and 0.8 Hz, H-3′); 4.27 (1H, dd, *J* = 10.7 and 3.3 Hz, H-22a); 4.00 (1H, dd, *J* = 10.7 and 7.4 Hz, H-22b); 2.63–2.55 (2H, m, H-5 and H-2); 2.01 (1H, dd, *J* = 12.6 and 12.6 Hz, H-7α); 1.17 (1H, ddd, *J* = 12.5, 11.8 and 5.9 Hz, H-15); 1.11 (3H, d, *J* = 6.6 Hz, H-21); 0.962 (3H, s, H-19); 0.740 (3H, s, H-18). ^13^C NMR (100.6 MHz, CDCl_3_) δ (ppm): 211.16 (C-3); 208.88 (C-6); 169.69 (CH_3_CO); 150.79 (C-2′); 133.78 (C-4′); 131.48 (C-6′); 125.97 (C-5′); 123.86 (C-3′); 123.39 (C-1′); 69.88 (C-22); 57.48 (C-5); 56.23 (C-14); 53.37 (C-9); 52.75 (C-17); 46.51 (C-7); 43.19 (C-13); 41.19 (C-10); 39.19 (C-12); 38.07 (C-1); 37.97 (C-8); 37.35 (C-2); 36.96 (C-4); 35.88 (C-20); 27.53 (C-16); 24.07 (C-15); 21.63 (C-11); 21.07 (CH_3_CO); 17.28 (C-21); 12.55 (C-19); 12.08 (C-18) (Figures S101–S105, Supplementary Material). HRMS-ESI (positive mode): *m*/*z* calculated for C_31_H_40_O_6_: 509.2898 [M + H]^+^; found 509.2899 [M + H]^+^ (Figure S121, Supplementary Material).

### 3.3. Bioactivity Measurements

#### 3.3.1. Rice Lamina Inclination Test (RLIT)

The bioactivity of BR analogs (**19**–**33** and precursor **18**) was evaluated by RLIT [20] using a modified version of a previously described procedure [7]. Seeds of a local rice (*Oryza sativa* L) cultivar of the Zafiro variety (provided by INIA-QUILAMAPU, Chillán, Chile) were sterilized, sown, and grown to the second internode of the lamina. Once 8 cm segments were obtained, they were placed in Petri dishes containing sterile distilled water (60 mL). Standard dissolutions of the BR analogs were prepared by dissolving pure compounds in 1 mL of THF and diluting them with water (0.01:50) to reach analog concentration of approximately 1 × 10^−5^ M. Then, different volumes of these dissolutions were added to obtain final concentrations of 1 × 10^−8^, 1 × 10^−7^, and 1 × 10^−6^ M. Brassinolide (**2**) (APExBIO; Houston, TX, USA) was used as a positive control at the same tested concentrations, while an aqueous solution of tetrahydrofuran (0.002%) was used as a negative control. After 72 h of incubation in the dark, the angle of inclination of the slice, between the leaf and the sheath, was measured. Images were taken with a microscope, including camera software (Leica Microsystem EZ4HD, Leica Application Suite X, Wetzlar, Germany). Each treatment consisted of 8 independent replicates. The effect of BR analogs on rice was assessed by comparing bending angles in the absence and presence of BRs. The measured bending angles (ba) are shown in Figure S122, and the differences between BRs and negative control are given in Table S1. For the sake of comparison, BR RLIT activities were calculated by normalizing these differences with respect to the negative control (Equation (1)).

(1) $$\mathrm{Activity}(\mathrm{RLIT})=\frac{\left[\mathrm{ba}(\mathrm{BRs}\;\mathrm{analogs})-\mathrm{ba}(\mathrm{negative}\;\mathrm{control})\right]}{\left[\mathrm{ba}(\mathrm{negative}\;\mathrm{control})\right]}$$

Finally, to obtain the relative activity with respect to the positive control, the values obtained from equation 1 were divided by the corresponding activity of brassinolide.

(2) $$\mathrm{Relative}\;\mathrm{activity}=\frac{\mathrm{Activity}(\mathrm{RLIT})}{\mathrm{Activity}(\mathrm{brassinolide})}$$

The data were subjected to an analysis of variance and an LSD–Fischer mean comparison test (*p* < 0.05) using InfoStat software version 2020 (Córdoba, Argentina).

#### 3.3.2. Bean Second-Internode Bioassay

The bean second-internode test for compounds **18** and **19**–**33** was performed following the procedure described in [21], with some modifications [7]. Bean (*Phaseolus vulgaris* L., cv. Pinto) seeds were sown, and plants were grown under controlled conditions into a plant growth chamber until the second internode was 1–2 mm long. Then, the bract was removed from the base of the second internode, and standard dissolutions of each treatment (10 µL, 1 × 10^−8^ M) and TWEEN^®^ 20 (AMRESCO, Solon, OH, USA) (2 µL) were applied. Control plants were treated with water and TWEEN^®^ 20 only. After 5 days, the difference between the length of the second internode of treated and control plants was measured. Data were subjected to an analysis of variance and an LSD–Fischer mean comparison test (*p* < 0.05) using InfoStat software version 2020 (Córdoba, Argentina).

## 4. Conclusions

A new series of 23,24-dinorcholanic 3,6-dioxo derivative analogs (compounds **19**–**33**) have been synthesized and fully characterized by their physical and spectroscopic properties. An assessment of their biological activity by using RLIT and BSI bioassays has been performed. RLIT data indicate that the incorporation of benzoylate function in the C-22 position induces an interesting increase in the angle opening at all tested concentrations. This activity depends on the position and nature of the phenyl substituent and is also dependent on concentration. At the lowest tested concentration, 1 × 10^−8^ M, analog **33** (*o*-OAc) exhibits the highest activity, i.e., slightly higher than that obtained for brassinolide. Thus, the data suggest that the chemical structure of the benzoate substituent on 3-DT derivatives is a key factor in determining their RLIT activity, but there it is not enough information to establish a clear correlation with activity. However, a comparison of the RLIT results obtained herein with those previously reported for other series of benzoylated analogs allows us to obtain a clearer structure–activity relationship. For example, 3-DT analogs carrying an extra alcohol group in the alkyl chain (C-22) exhibit lower activity than the 3-DT analogs studied herein. On the other hand, benzoylated BR analogs with a -OH group at C-3 are much more active than the respective derivatives with carbonyl group in this position. However, in both series, the activity increases for benzoylated groups substituted with -OCH_3_ and I atom in the *para*-position. This effect has been attributed to the increasing size of these BRs derivatives. Thus, it would be interesting to evaluate the activity of benzoylated TE derivatives with the substituent in the *ortho*-position.

Interestingly, *ortho*-derivatives are significantly more active in the bean second-internode elongation bioassay, except derivative **21** (*p*-OCH_3_), which is even more active than brassinolide and all other analogs. These results are clearly different from those obtained in the RLIT bioassay, and therefore, it can be concluded that the activity of BR derivatives cannot be determined by just one bioassay. So, their application and activity–structure relationship will always be dependent on the bioassay used to determine activity.

Finally, it is important to emphasize that the synthesis of **33**, the most active 3-DT analog, is performed with a total reaction yield of 17% and using hyodeoxycholic acid, a secondary bile acid that is commercially available, as the starting material. This means that the synthesis described herein could be scaled for a potential application of this analog.

## Figures and Tables

**Scheme 2:**
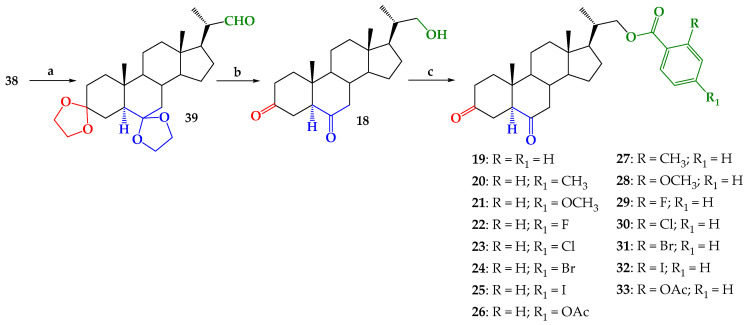
Synthesis of compounds **39**, **18**, and new 3-DT analogs **19**–**33**. Conditions **a**. OsO_4_/NaIO_4_/THF/H_2_O, N_2_, r.t., 6 h, 57.1%. **b**. (1) NaBH_4_/THF/CH_3_OH, r.t., 2h; (2) HCl 5% *v*/*v*, 40 °C, 2h, 80.5%. **c**. 4-R-PhCOCl and 2-R-PhCOCl/CH_2_Cl_2_/C_5_H_5_N/DMAP, r.t., 2h, **19**: 44.4%; **20**: 50.0%; **21**: 18.4%; **22**: 59.2%; **23**: 28.7%; **24**: 58,9%; **25**: 27.0%; **26**: 63.8%; **27**: 40.3%; **28**: 63.4%; **29**: 47.3%; **30**: 45.7%; **31**: 52.4%; **32**: 76.9%; and **33**: 55.9%.

**Table 1:** RLIT activities obtained by measurements of bending angles at different concentrations of 3-DT derivatives.

RLIT Activities ± (Standard Error) ^1^			
Compounds	1 × 10^−8^ M	1 × 10^−7^ M	1 × 10^−6^ M
Brassinolide (**2**)	2.3 ± 1.4 ^d^	3.13 ± 1.7 ^e^	4.17 ± 1.5 ^f^
**18**	-	0.13 ± 3.8 ^a^	0.70 ± 4.2 ^a^
**19** (*p*-H)	1.65 ± 5.2 ^c^	1.22 ± 4.9 ^c^	0.48 ± 4.1 ^b^
**20** (*p*-CH_3_)	1.83 ± 8.2 ^c^	1.83 ± 4.2 ^d^	1.30 ± 4.9 ^d^
**27** (*o*-CH_3_)	0.52 ± 5.8 ^a^	1.13 ± 5.2 ^c^	1.48 ± 4.1 ^d^
**21 (***p*-OCH_3_)	0.83 ± 5.5 ^b^	1.22 ± 4.1 ^c^	2.70 ± 8.8 ^e^
**28** (*o*-OCH_3_)	1.87 ± 5.8 ^c^	0.91 ± 4.1 ^b^	0.26 ± 2.0 ^a^
**22** (*p*-F)	1.78 ± 5.2 ^c^	0.87 ± 2.0 ^b^	0.83 ± 4.5 ^c^
**29** (*o*-F)	0.56 ± 4.5 ^a^	0.83 ± 2.6 ^b^	2.52 ± 3.2 ^e^
**23** (*p*-Cl)	1.04 ± 3.3 ^b^	0.74 ± 2.7 ^b^	1.22 ± 4.1 ^d^
**30** (*o*-Cl)	-	0.56 ± 4.1 ^a^	1.43 ± 4.5 ^d^
**24** (*p*-Br)	-	0.13 ± 1.1 ^a^	0.13 ± 1.0 ^a^
**31** (*o*-Br)	1.78 ± 5.2 ^c^	1.34 ± 2.6 ^d^	0.70 ± 7.5 ^b^
**25** (*p*-I)	1.78 ± 8.2 ^c^	1.74 ± 2.0 ^d^	0.78 ± 2.6 ^c^
**32** (*o*-I)	2.0 ± 2.3 ^c^	1.4 ± 4.9 ^d^	0.96 ± 3.8 ^c^
**26** (*p*-OAc)	1.74 ± 3.8 ^c^	1.83 ± 2.7 ^d^	0.83 ± 5.0 ^c^
**33** (*o*-OAc)	2.74 ± 6.6 ^d^	1.87 ± 4.5 ^e^	1.04 ± 2.6 ^c^

^1^ Values are the mean of leaf–sheath bending angle, normalized to the negative control (23 ± 2.7°). Data are expressed as mean ± standard deviation of two independent experiments, each with at least eight replicates. Superscript letters indicate significant differences between treatments according to the LSD multiple comparison test (*p* < 0.05). (-) indicates lower values than the negative control. Brassinolide was used as a positive control.

**Table 2:** RLIT relative activity (RA) at 1 × 10^−8^ M, using brassinolide (**2**) as a reference compound.

3-DT	Activity	TE	Activity (Ref. [6])	3-DT(C22-OH)	Activity (Ref. [7])
18 *	-	(*)	0.95	(*)	0.32
**19** (*p*-H)	0.72	**3** (*p*-H)	0.95	**11** (*p*-H)	0.24
**20** (*p*-CH_3_)	0.80	**4** (*p*-CH_3_)	1.05	**12** (*p*-CH_3_)	0.24
**21** (*p*-OCH_3_)	0.36	**5** (*p*-OCH_3_)	1.50	**13** (*p*-OCH_3_)	-
**22** (*p*-F)	0.77	**6** (*p*-F)	0.73	**14** (*p*-F)	0.47
**23** (*p*-Cl)	0.45	**7** (*p*-Cl)	0.55	**15** (*p*-Cl)	0.40
**24** (*p*-Br)	-	**8** (*p*-Br)	0.68	**16** (*p*-Br)	0.93
**25** (*p*-I)	0.77	**9** (*p*-I)	1.73		
**26** (*p*-OAc)	0.76				
**33** (*o*-OAc)	1.19				

(-) No activity. (*) Precursor with no benzoylate function.

**Table 3:** Activities in the bean second-internode bioassay of brassinolide (**2**), **18**, and 3-DT derivatives **19**–**33** at 1 × 10^−8^ M.

Compounds	Elongation of the Second Internode, mm ± SD
**Brassinolide** (**2**)	7.67 ± 2.35 ^b^
**18**	2.67 ± 1.0 ^d,e^
**19** (*p*-H)	3.17 ± 0.89 ^d,e^
**20** (*p*-CH_3_)	1.67 ± 1.0 ^e,f^
**27** (*o*-CH_3_)	6.83 ± 3.0 ^b^
**21** (*p*-OCH_3_)	13.67 ± 2.90 ^a^
**28** (*o*-OCH_3_)	6.00 ± 2.0 ^b,c^
**22** (*p*-F)	6.17 ± 1.79 ^b,c^
**29** (*o*-F)	7.00 ± 2.48 ^b^
**23** (*p*-Cl)	3.17 ± 0.89 ^e,f^
**30** (*o*-Cl)	7.17 ± 2.8 ^b^
**24** (*p*-Br)	0.00 ± 0
**31** (*o*-Br)	2.17 ± 0.89 ^d,e,f^
**25** (*p*-I)	2.50 ± 1.51 ^d,e^
**32** (*o*-I)	5.83 ± 2.8 ^b,c^
**26** (*p*-OAc)	1.83 ± 1.0 ^e,f^
**33** (*o*-OAc)	4.33 ± 2.48 ^c,d^
**Negative Control**	1.00 ± 0.0 ^f^

Letters indicate the level of significance according to the LSD–Fischer test; *p* < 0.05.

## Data Availability

Data are contained within the article and Supplementary Materials.

## Supplementary Materials

The following supporting information can be downloaded at https://www.mdpi.com/article/10.3390/ijms26178710/s1.
