# Supplementary material for: Novel 3-Dehydroteasterone Derivatives with 23,24-Dinorcholanic Side Chain and Benzoate Groups at C-22: Synthesis and Activity Evaluation by Rice Lamina Inclination Test and Bean Second-Internode Bioassay
Source: Int J Mol Sci. 2025 Sep 6;26(17):8710. doi: 10.3390/ijms26178710 (PMC12429595; doi:10.3390/ijms26178710)
Supplement: Supplementary file 1 [file ijms-26-08710-s001.zip › ijms-3810249-supplementary.pdf]

Article

Novel 3-dehydroteasterone derivatives with 23,24-dinorcholanic side chain and benzoate groups at C-22. Synthesis and activity evaluation by Rice Lamina Inclination Test and Bean Second Internode Bioassay.

Ernesto Valdés [1], Katy Díaz [1], María Núñez [1], Andrés F. Olea* [2], José F. Quilez del Moral [3], Rodrigo Carvajal [4], Mauricio A. Cuellar [4,5] and Luis Espinoza-Catalán* [1]

[1] Departamento de Química, Universidad Técnica Federico Santa María, Avenida España 1680, Valparaíso 2340000, Chile; ernesto.valdes@usm.cl (E.V.); katy.diaz@usm.cl (K.D.); maria.nunezg@usm.cl (M.N.); luis.espinozac@usm.cl. (L.E.C.)
[2] Instituto de Ciencias Químicas Aplicadas, Facultad de Ingeniería, Universidad Autónoma de Chile, Av. del Valle Sur 534, Santiago, Chile and CP 8580640; andres.olea@uautonoma.cl (A.F.O)
[3] Departamento de Química Orgánica, Instituto de Biotecnología, Universidad de Granada, 18071, Granada, Spain; jfquilez@ugr.es (J. F. Q. M.)
[4] Facultad de Farmacia, Escuela de Química y Farmacia, Universidad de Valparaíso, Av. Gran Bretaña 1093, Valparaíso 2340000, Chile. rodrigo.carvajal@usm.cl.
[5] Centro de Investigación, Desarrollo e Innovación de Productos Bioactivos (CINBIO), Universidad de Valparaíso, Valparaíso 2340000, Chile mauricio.cuellar@uv.cl (M.A.C.).

* Correspondence: luis.espiozac@usm.cl; Tel.: +56-32-2654425 (L.E.C.); andres.olea@uautonoma.cl; (A.F.O.)

Index **pag.**

S1. ^{1}H NMR spectrum compound **35**……………………………7

S2. ^{13}C NMR spectrum compound **35**……………………………8

S3. ^{13}C DEPT-135 NMR spectrum compound **35**……………………………9

S4. 2D HSQC spectrum compound **35**……………………………10

S5. 2D HMBC spectrum compound **35**……………………………11

S6. ^{1}H NMR spectrum compound **36**……………………………12

S7. ^{13}C NMR spectrum compound **36**……………………………13

S8. ^{13}C DEPT-135 NMR spectrum compound **36**……………………………14

S9. 2D HSQC spectrum compound **36**……………………………15

S10. 2D HMBC spectrum compound **36**........16
S11. ^{1}H NMR spectrum compound **37**........17
S12. ^{13}C NMR spectrum compound **37**........18
S13. ^{13}C DEPT-135 NMR spectrum compound **37**........19
S14. 2D HSQC spectrum compound **37**........20
S15. 2D HMBC spectrum compound **37**........21
S16. ^{1}H NMR spectrum compound **38**........22
S17. ^{13}C NMR compound **38**........23
S18. ^{13}C DEPT-135 NMR compound **38**........24
S19. 2D HSQC compound **38**........25
S20. 2D HMBC compound **38**........26
S21. ^{1}H NMR spectrum compound **39**........27
S22. ^{13}C NMR spectrum compound **39**........28
S23. ^{13}C DEPT-135 NMR spectrum compound **39**........29
S24. 2D HSQC spectrum compound **39**........30
S25. 2D HMBC spectrum compound **39**........31
S26. ^{1}H NMR spectrum compound **18**........32
S27. ^{13}C NMR spectrum compound **18**........33
S28. ^{13}C DEPT-135 NMR spectrum compound **18**........34
S29. 2D HSQC spectrum compound **18**........35
S30. 2D HMBC spectrum compound **18**........36
S31. ^{1}H NMR spectrum compound **19**........37
S32. ^{13}C NMR spectrum compound **19**........38
S33. ^{13}C DEPT-135 NMR spectrum compound **19**........39
S34. 2D HSQC spectrum compound **19**........40
S35. 2D HMBC spectrum compound **19**........41
S36. ^{1}H NMR spectrum compound **20**........42
S37. ^{13}C NMR spectrum compound **20**........43

S38. ^{13}C DEPT-135 NMR spectrum compound **20**……44
S39. 2D HSQC spectrum compound **20**……45
S40. 2D HMBC spectrum compound **20**……46
S41. ^{1}H NMR spectrum compound **21**……47
S42. ^{13}C NMR spectrum compound **21**……48
S43. ^{13}C DEPT-135 NMR spectrum compound **21**……49
S44. 2D HSQC spectrum compound **21**……50
S45. 2D HMBC spectrum compound **21**……51
S46. ^{1}H NMR spectrum compound **22**……52
S47. ^{13}C NMR spectrum compound **22**……53
S48. ^{13}C DEPT-135 NMR spectrum compound **22**……54
S49. 2D HSQC spectrum compound **22**……55
S50. 2D HMBC spectrum compound **22**……56
S51. ^{1}H NMR spectrum compound **23**……57
S52. ^{13}C NMR spectrum compound **23**……58
S53. ^{13}C DEPT-135 NMR spectrum compound **23**……59
S54. 2D HSQC spectrum compound **23**……60
S55. 2D HMBC spectrum compound **23**……61
S56. ^{1}H NMR spectrum compound **24**……62
S57. ^{13}C NMR spectrum compound **24**……63
S58. ^{13}C DEPT-135 NMR spectrum compound **24**……64
S59. 2D HSQC spectrum compound **24**……65
S60. 2D HMBC spectrum compound **24**……66
S61. ^{1}H NMR spectrum compound **25**……67
S62. ^{13}C NMR spectrum compound **25**……68
S63. ^{13}C DEPT-135 NMR spectrum compound **25**……69
S64. 2D HSQC spectrum compound **25**……70
S65. 2D HMBC spectrum compound **25**……71

S66. ^{1}H NMR spectrum compound **26**……………………………………72
S67. ^{13}C NMR spectrum compound **26**……………………………………73
S68. ^{13}C DEPT-135 NMR spectrum compound **26**……………………………………74
S69. 2D HSQC spectrum compound **26**……………………………………75
S70. 2D HMBC spectrum compound **26**……………………………………76
S71. ^{1}H NMR spectrum compound **27**……………………………………77
S72. ^{13}C NMR spectrum compound **27**……………………………………78
S73. ^{13}C DEPT-135 NMR spectrum compound **27**……………………………………79
S74. 2D HSQC spectrum compound **27**……………………………………80
S75. 2D HMBC spectrum compound **27**……………………………………81
S76. ^{1}H NMR spectrum compound **28**……………………………………82
S77. ^{13}C NMR spectrum compound **28**……………………………………83
S78. ^{13}C DEPT-135 NMR spectrum compound **28**……………………………………84
S79. 2D HSQC spectrum compound **28**……………………………………85
S80. 2D HMBC spectrum compound **28**……………………………………86
S81. ^{1}H NMR spectrum compound **29**……………………………………87
S82. ^{13}C NMR spectrum compound **29**……………………………………88
S83. ^{13}C DEPT-135 NMR spectrum compound **29**……………………………………89
S84. 2D HSQC spectrum compound **29**……………………………………90
S85. 2D HMBC spectrum compound **29**……………………………………91
S86. ^{1}H NMR spectrum compound **30**……………………………………92
S87. ^{13}C NMR spectrum compound **30**……………………………………93
S88. ^{13}C DEPT-135 NMR spectrum compound **30**……………………………………94
S89. 2D HSQC spectrum compound **30**……………………………………95
S90. 2D HMBC spectrum compound **30**……………………………………96
S91. ^{1}H NMR spectrum compound **31**……………………………………97
S92. ^{13}C NMR spectrum compound **31**……………………………………98
S93. ^{13}C DEPT-135 NMR spectrum compound **31**……………………………………99

S94. 2D HSQC spectrum compound **31**……………………………………………………………..100

S95. 2D HMBC spectrum compound **31**……………………………………………………………..101

S96. ^{1}H NMR spectrum compound **32**……………………………………………………………102

S97. ^{13}C NMR spectrum compound **32**…………………………………………………………...103

S98. ^{13}C DEPT-135 NMR spectrum compound **32**……………………………………………….104

S99. 2D HSQC spectrum compound **32**………………………………………………………………105

S100. 2D HMBC spectrum compound **32**……………………………………………………………106

S101. ^{1}H NMR spectrum compound **33**…………………………………………………………107

S102. ^{13}C NMR spectrum compound **33**…………………………………………………………...108

S103. ^{13}C DEPT-135 NMR spectrum compound **33**……………………………………………109

S104. 2D HSQC spectrum compound **33**……………………………………………………………110

S105. 2D HMBC spectrum compound **33**……………………………………………………………111

S106. HRSM-ESI spectra of compound **18**……………………………………………………………..112

S107. HRSM-ESI spectra of compound **19**……………………………………………………………112

S108. HRSM-ESI spectra of compound **20**……………………………………………………………113

S109. HRSM-ESI spectra of compound **21**……………………………………………………………113

S110. HRSM-ESI spectra of compound **22**……………………………………………………………114

S111. HRSM-ESI spectra of compound **23**……………………………………………………………114

S112. HRSM-ESI spectra of compound **24**……………………………………………………………115

S113. HRSM-ESI spectra of compound **25**……………………………………………………………115

S114. HRSM-ESI spectra of compound **26**……………………………………………………………116

S115. HRSM-ESI spectra of compound **27**……………………………………………………………116

S116. HRSM-ESI spectra of compound **28**……………………………………………………………117

S117. HRSM-ESI spectra of compound **29**……………………………………………………………117

S118. HRSM-ESI spectra of compound **30**……………………………………………………………118

S119. HRSM-ESI spectra of compound **31**……………………………………………………………118

S120. HRSM-ESI spectra of compound **32**……………………………………………………………119

S121. HRSM-ESI spectra of compound **33**……………………………………………………………119

S122. Effect of BRs Analogs on the Rice Lamina Inclination of compounds **2**, **18** and **19-33**………….……….……120

Table S1. Effect of different concentrations of brassinolide (**2**) and **3-DT** derivatives on lamina inclination of rice seedlings……………………………………………………………………………………………….…124

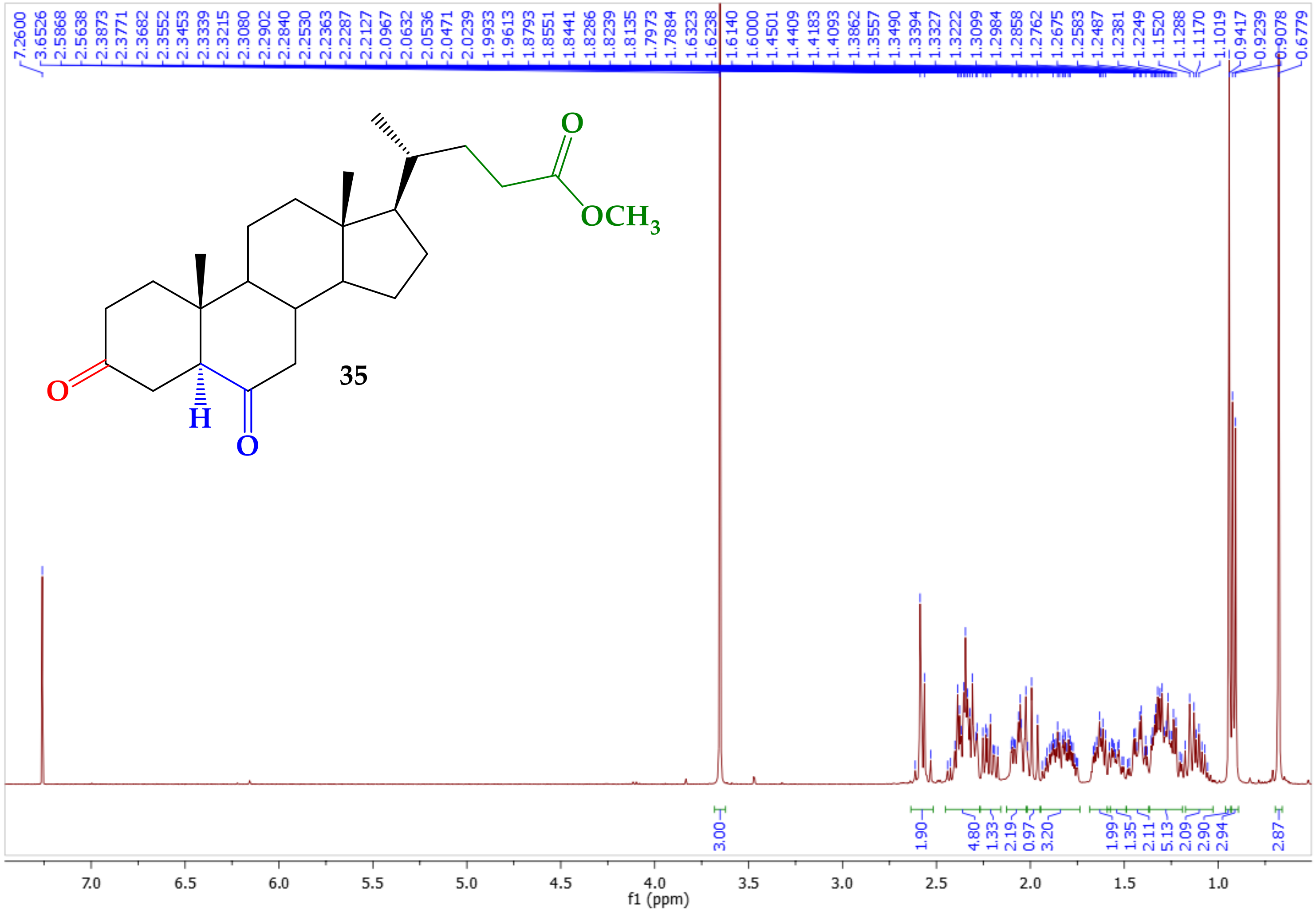

S1. ^{1}H NMR spectrum of *Methyl 3,6-dioxo-5α-cholan-24-oate* (**35**).

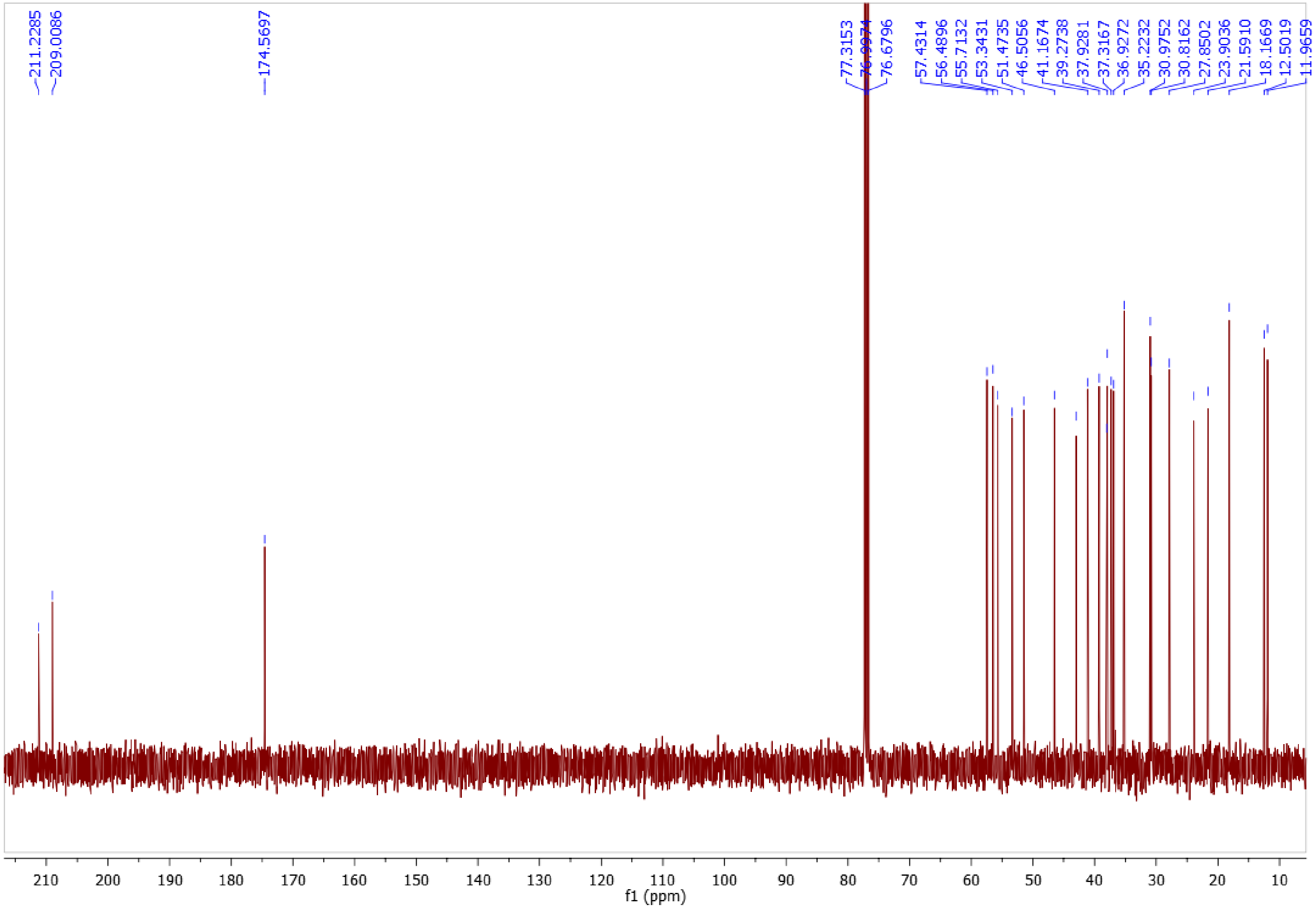

S2. ^{13}C NMR spectrum of *Methyl 3,6-dioxo-5α-cholan-24-oate* (**35**).

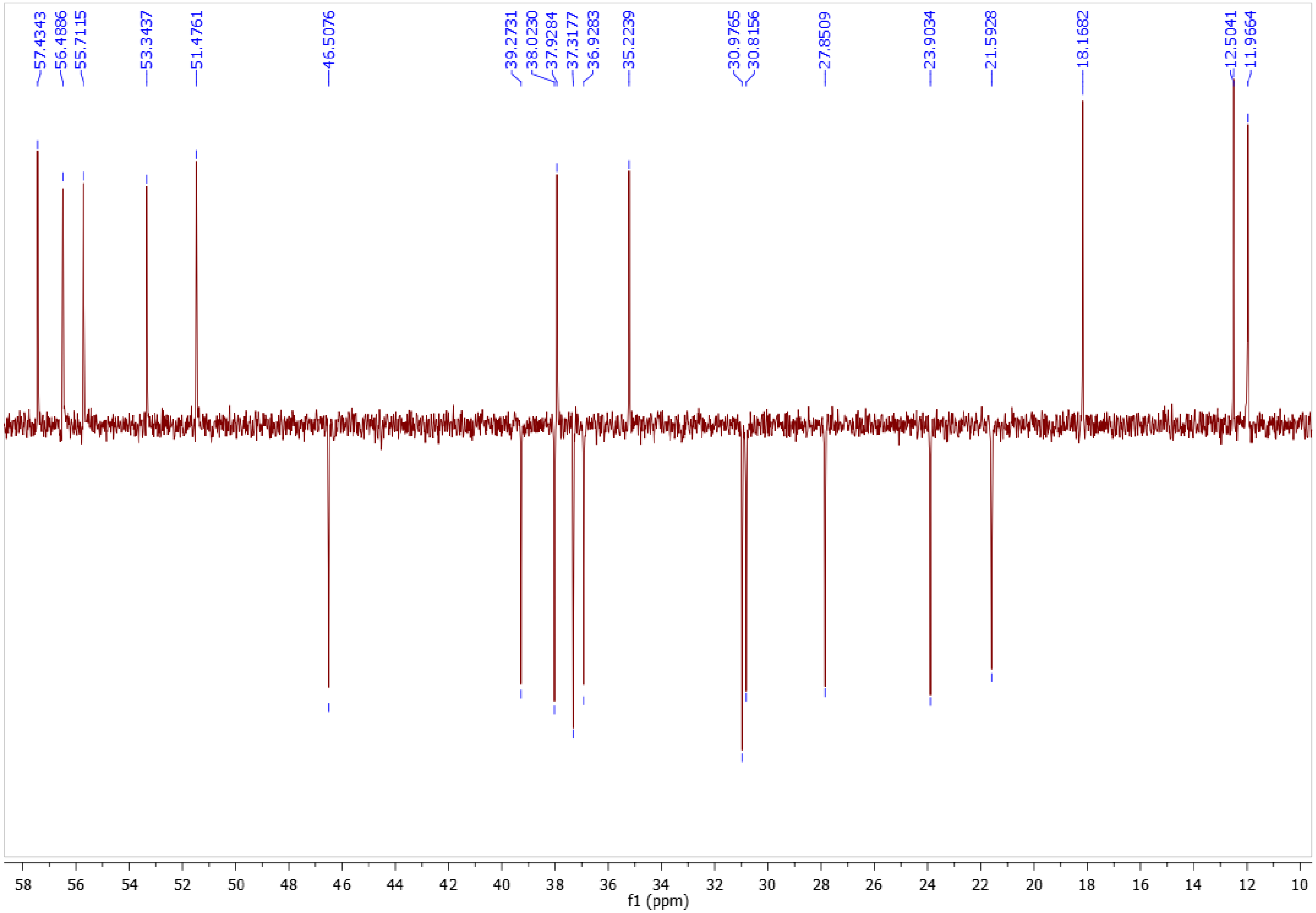

S3. ^{13}C NMR DEPT-135 spectrum of *Methyl 3,6-dioxo-5α-cholan-24-oate* (**35**).

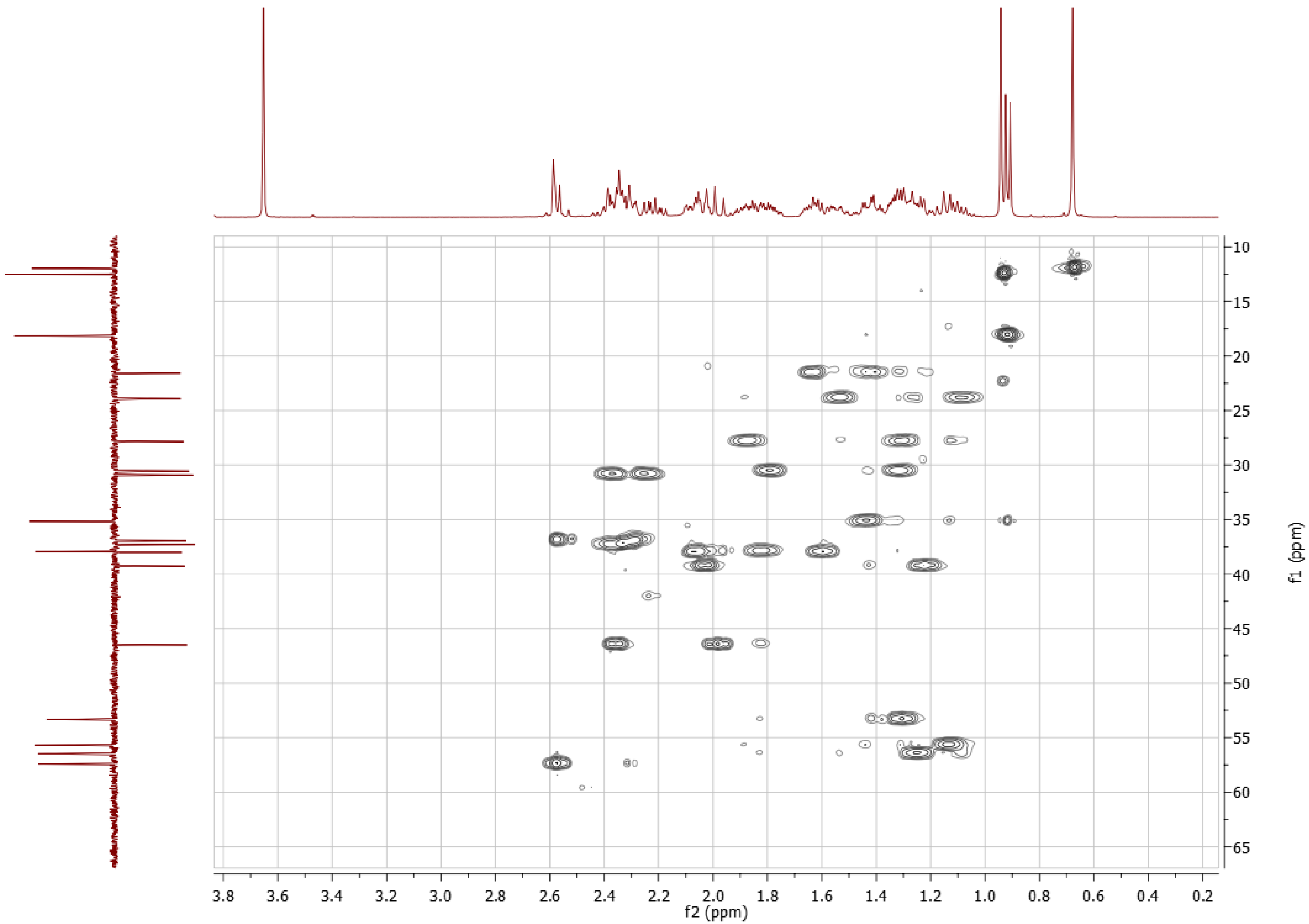

S4. 2D HSQC spectrum of *Methyl 3,6-dioxo-5α-cholan-24-oate* (**35**).

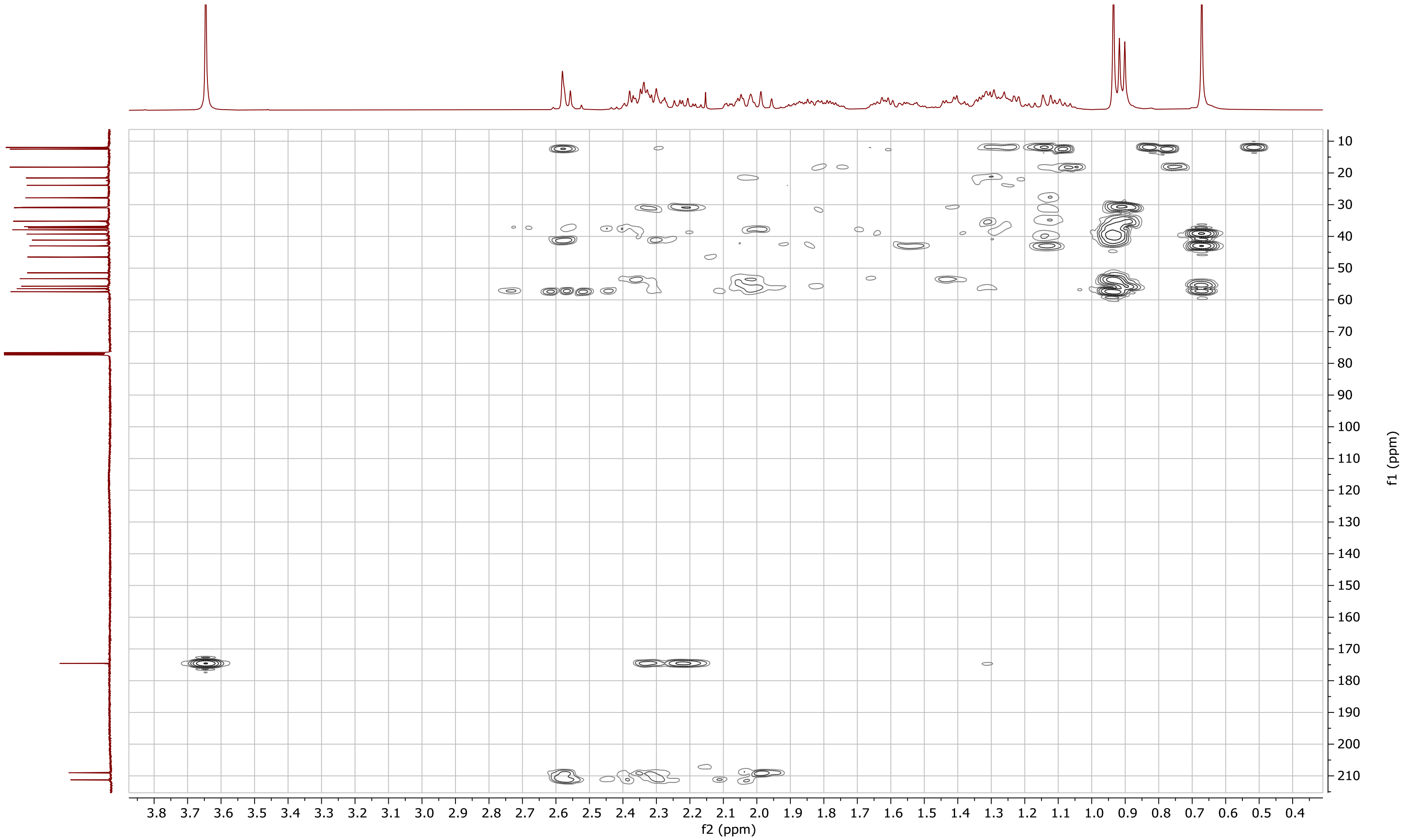

S5. 2D HMBC spectrum of *Methyl 3,6-dioxo-5α-cholan-24-oate* (**35**).

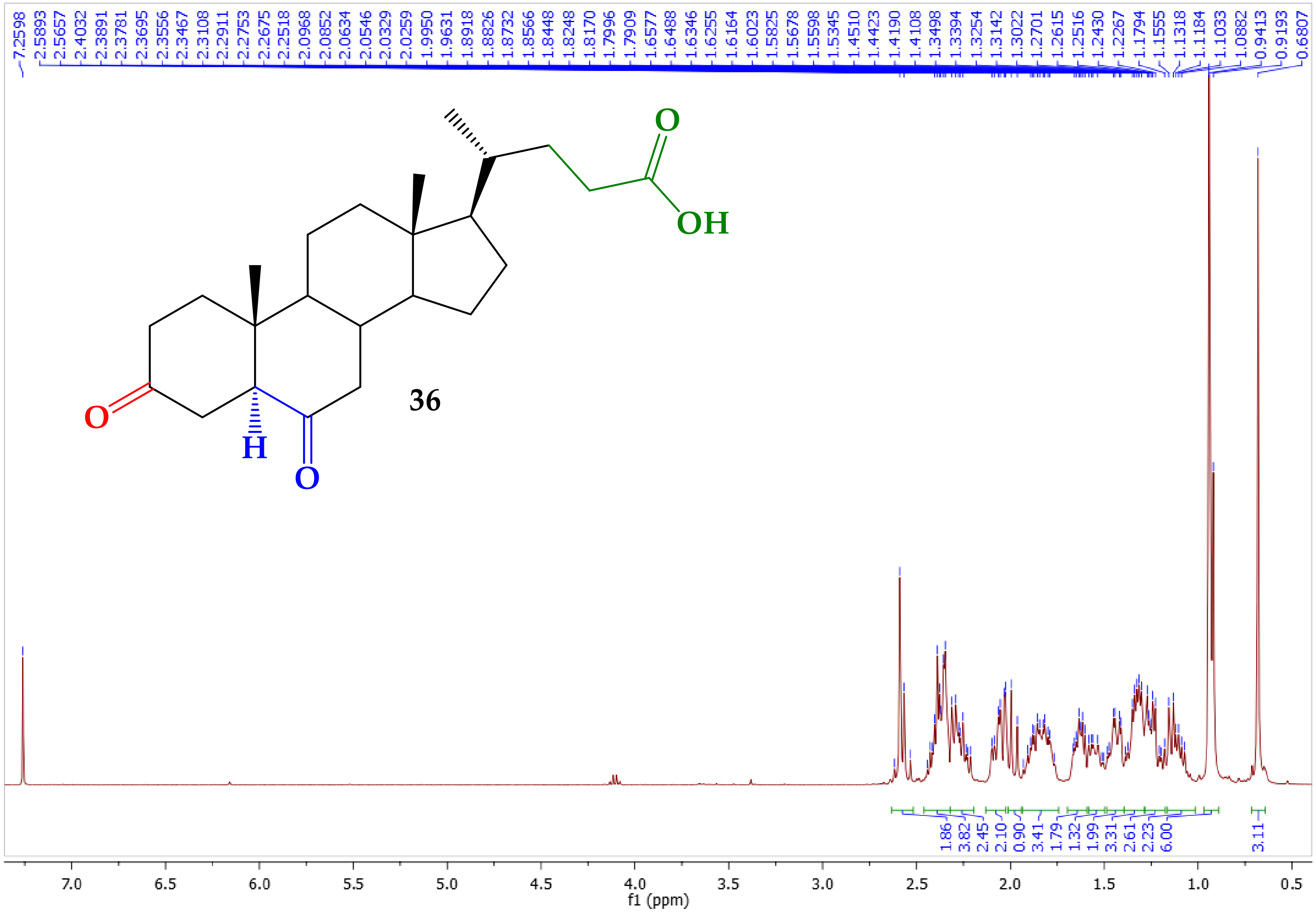

S6. ¹H NMR spectrum of *3,6-dioxo-5α-cholan-24-oic acid (**36**).*

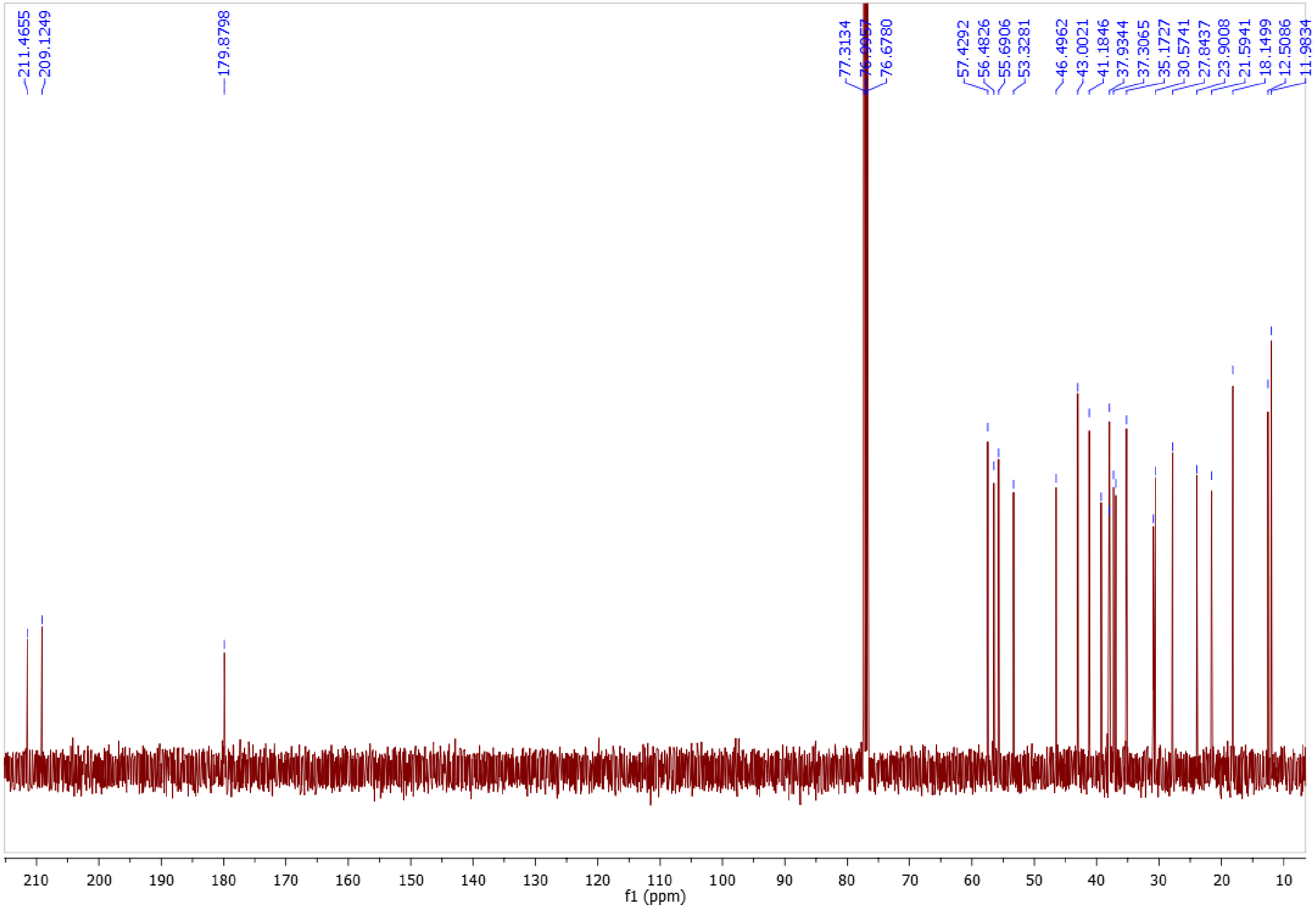

S7. ^{13}C NMR spectrum of *3,6-dioxo-5α-cholan-24-oic acid* (***36***).

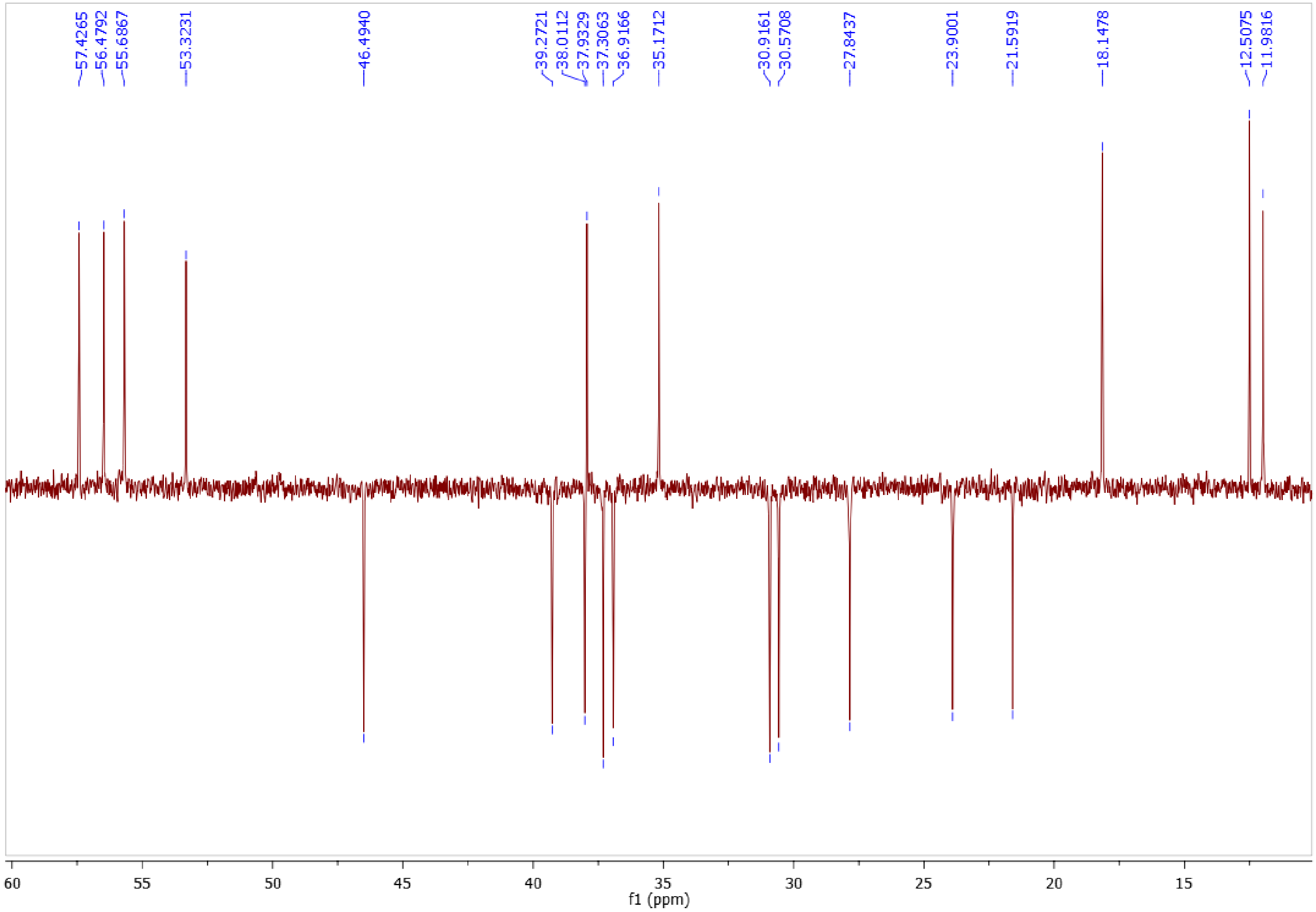

S8. ^{13}C NMR DEPT-135 spectrum of *3,6-dioxo-5α-cholan-24-oic acid* (***36***).

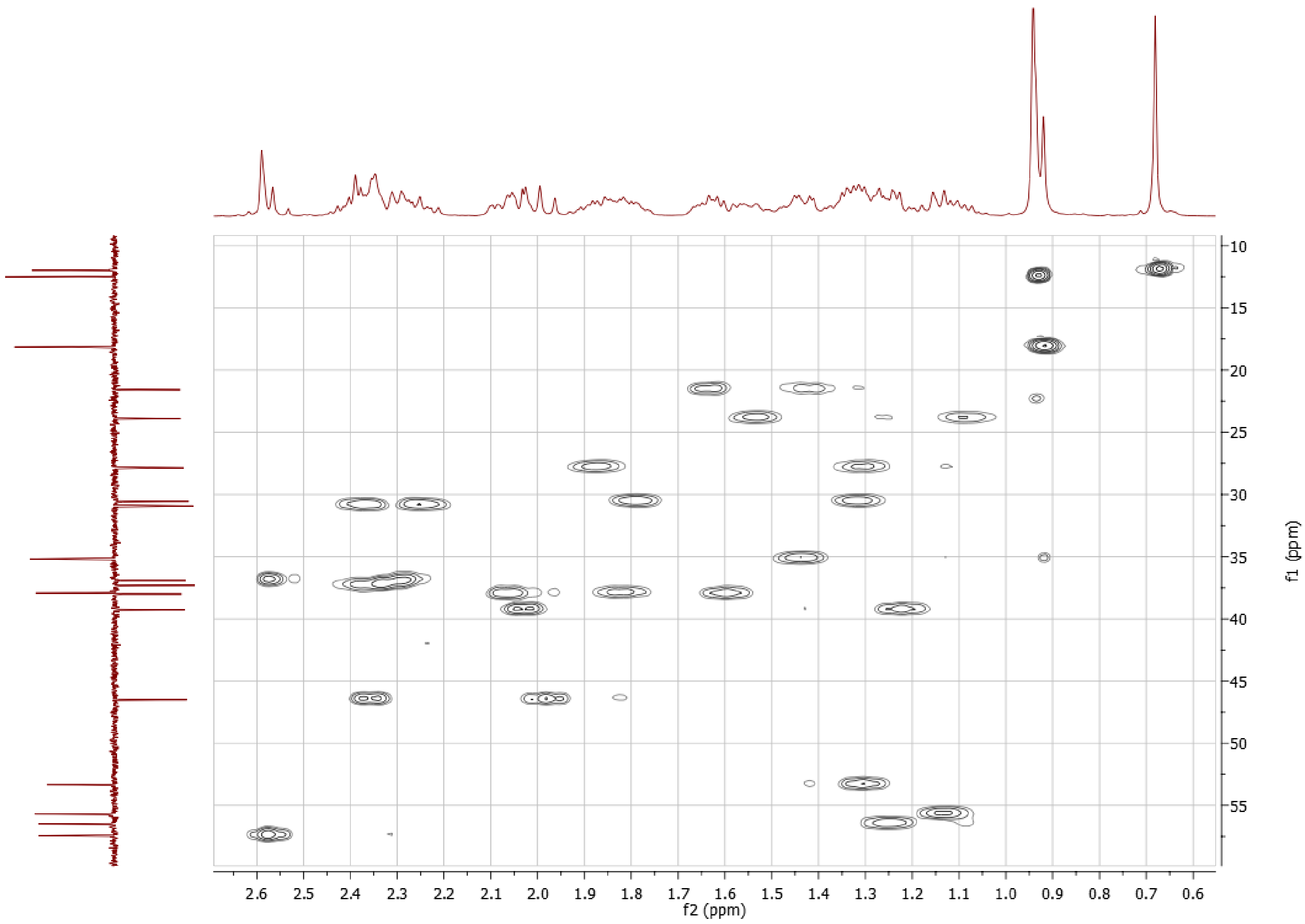

S9. 2D HSQC spectrum of *3,6-dioxo-5α-cholan-24-oic acid* (***36***).

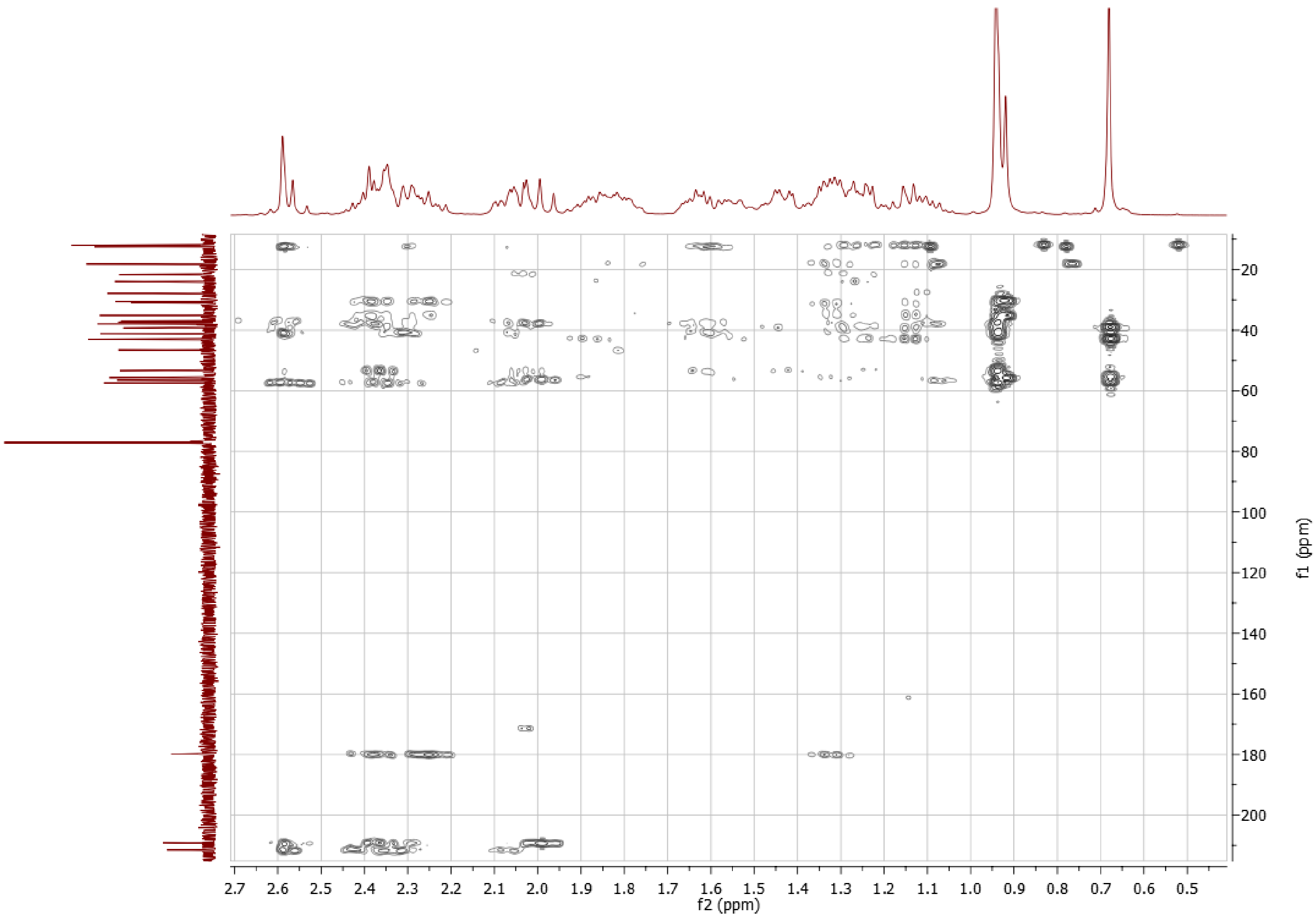

S10. 2D HMBC spectrum of *3,6-dioxo-5α-cholan-24-oic acid* (***36***).

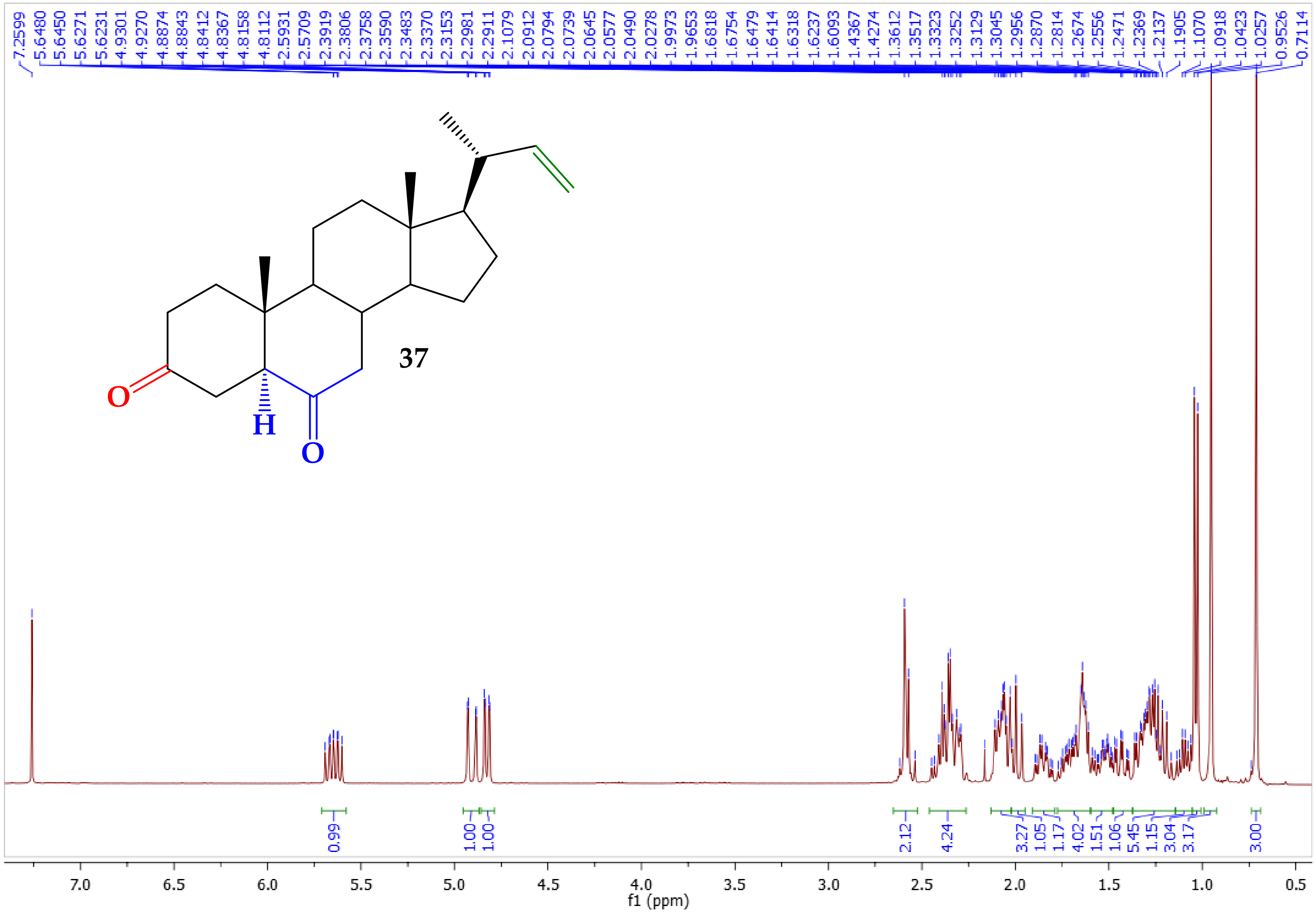

S11. ^{1}H NMR spectrum of *24-nor-5α-chol-22-ene-3,6-dione* (**37**).

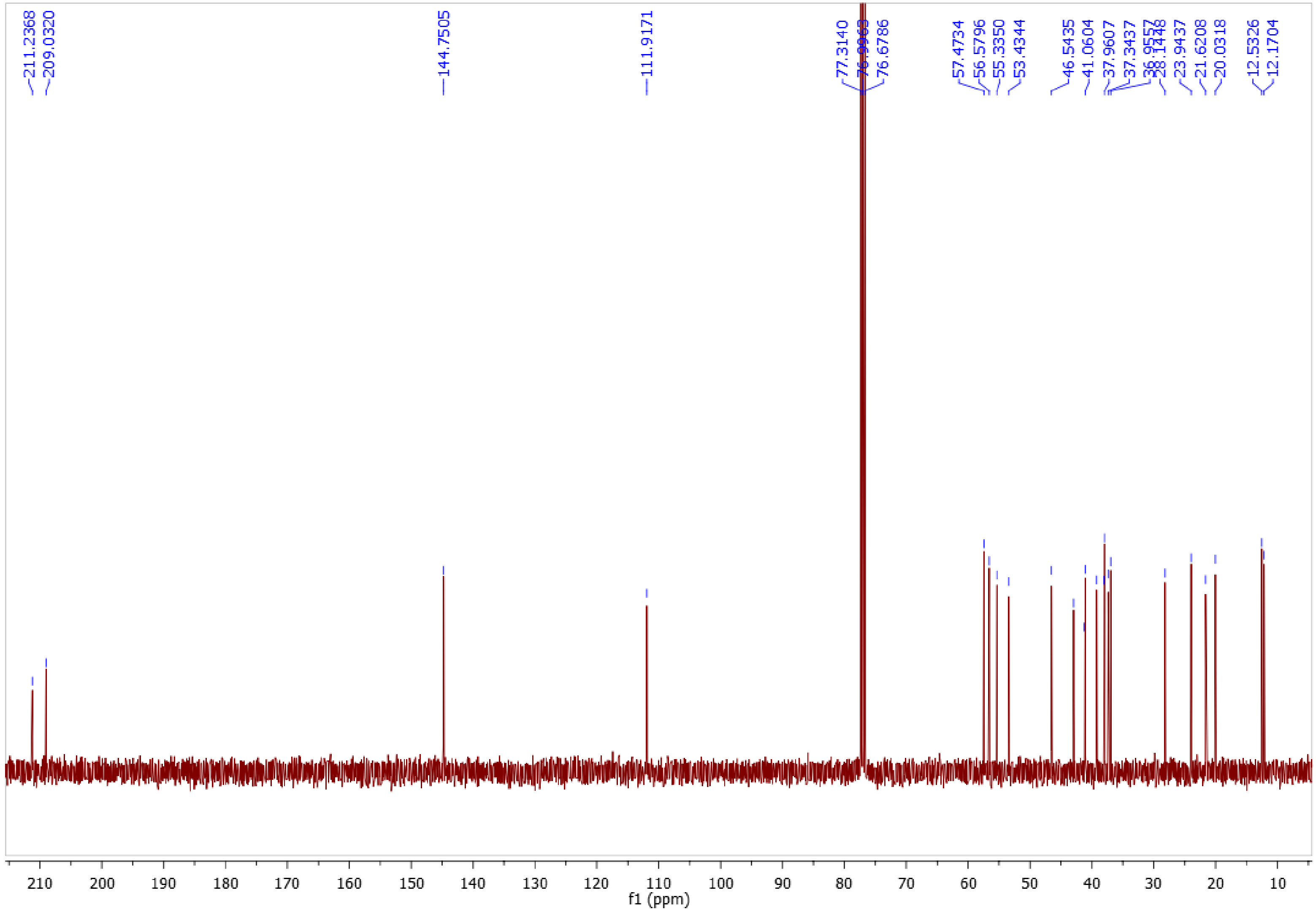

S12. ^{13}C NMR spectrum of *24-nor-5α-chol-22-ene-3,6-dione* (**37**).

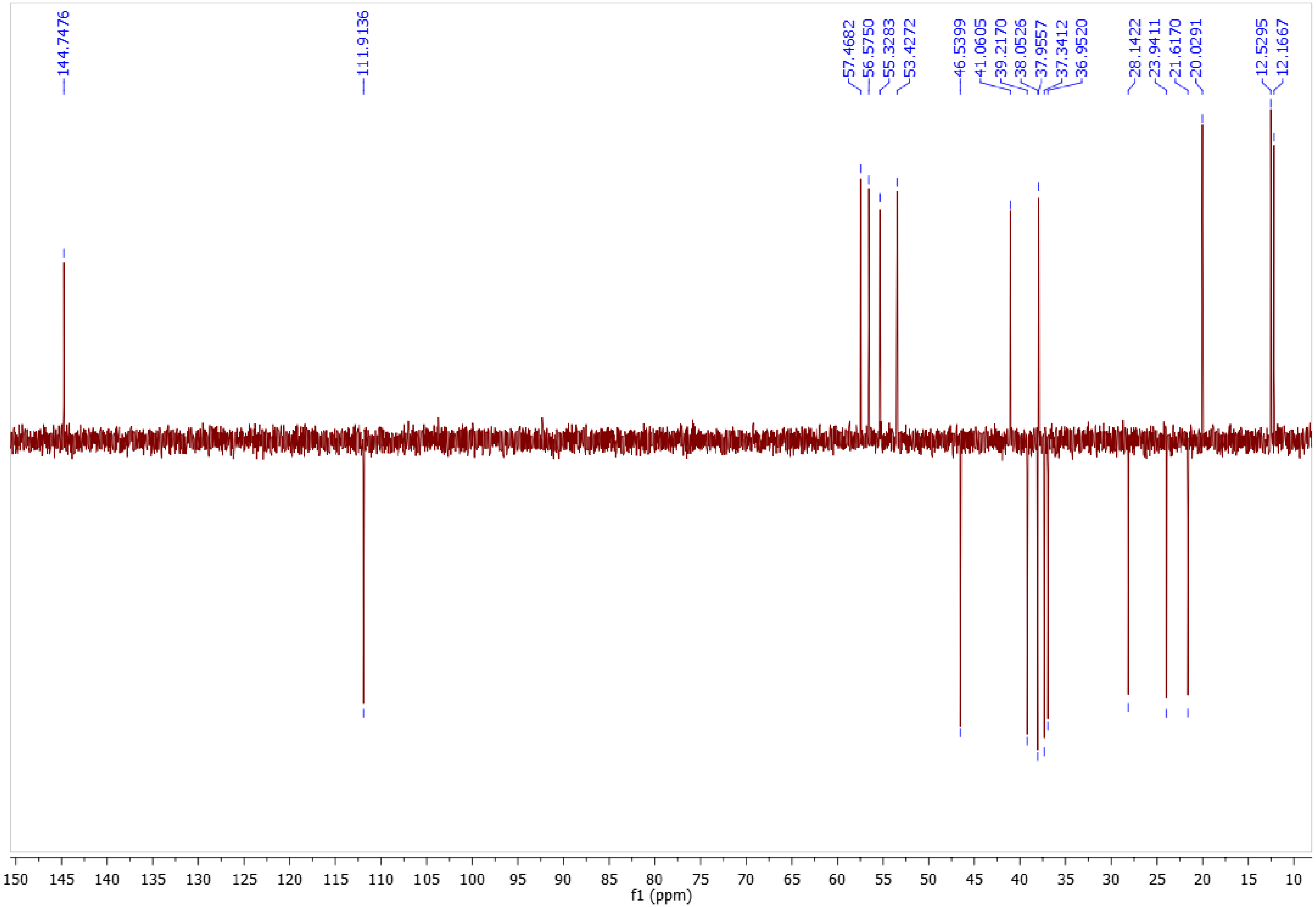

S13. ^{13}C DEPT-135 NMR spectrum of *24-nor-5α-chol-22-ene-3,6-dione* (**37**).

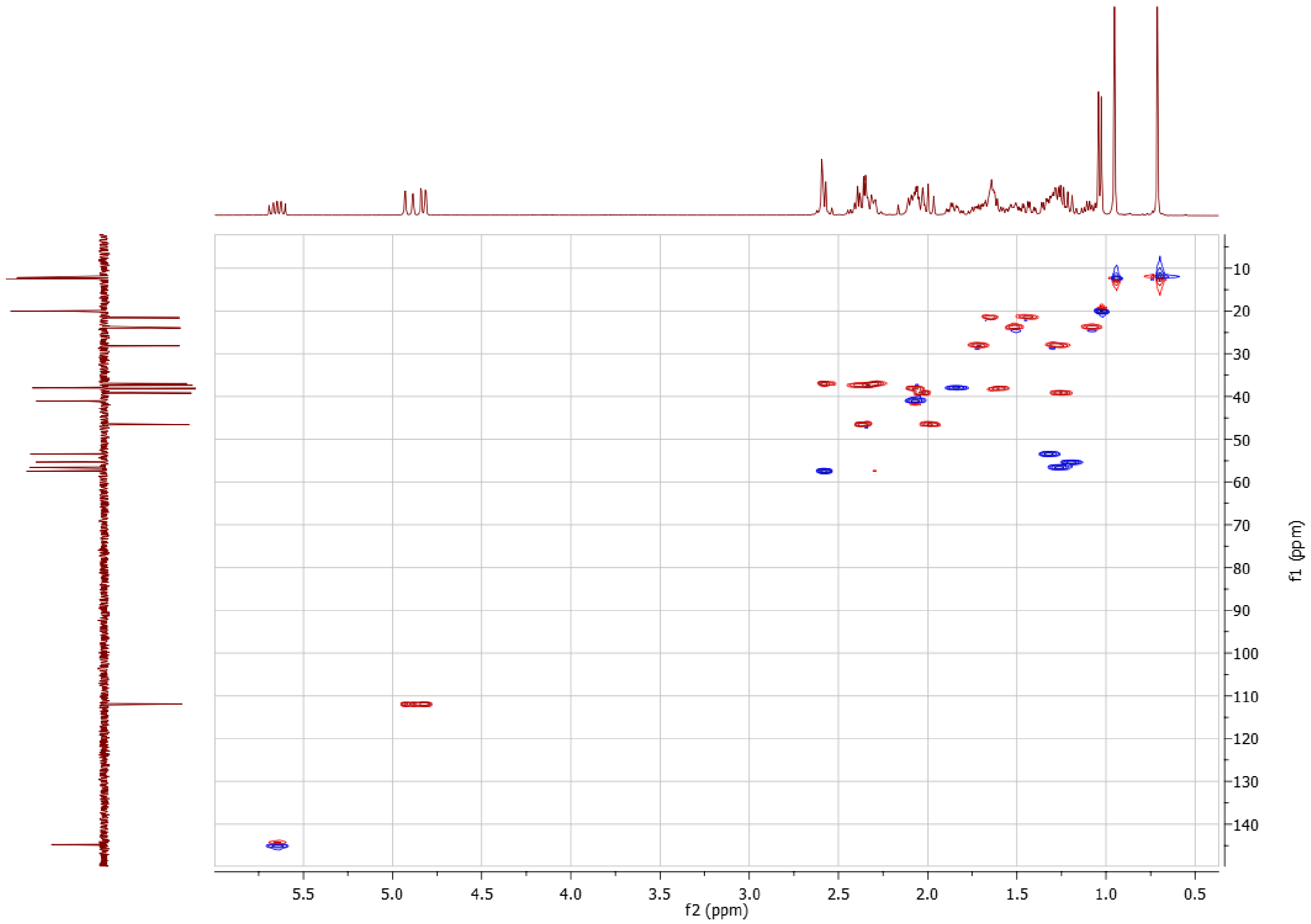

S14. 2D HSQC NMR spectrum of *24-nor-5α-chol-22-ene-3,6-dione* (**37**).

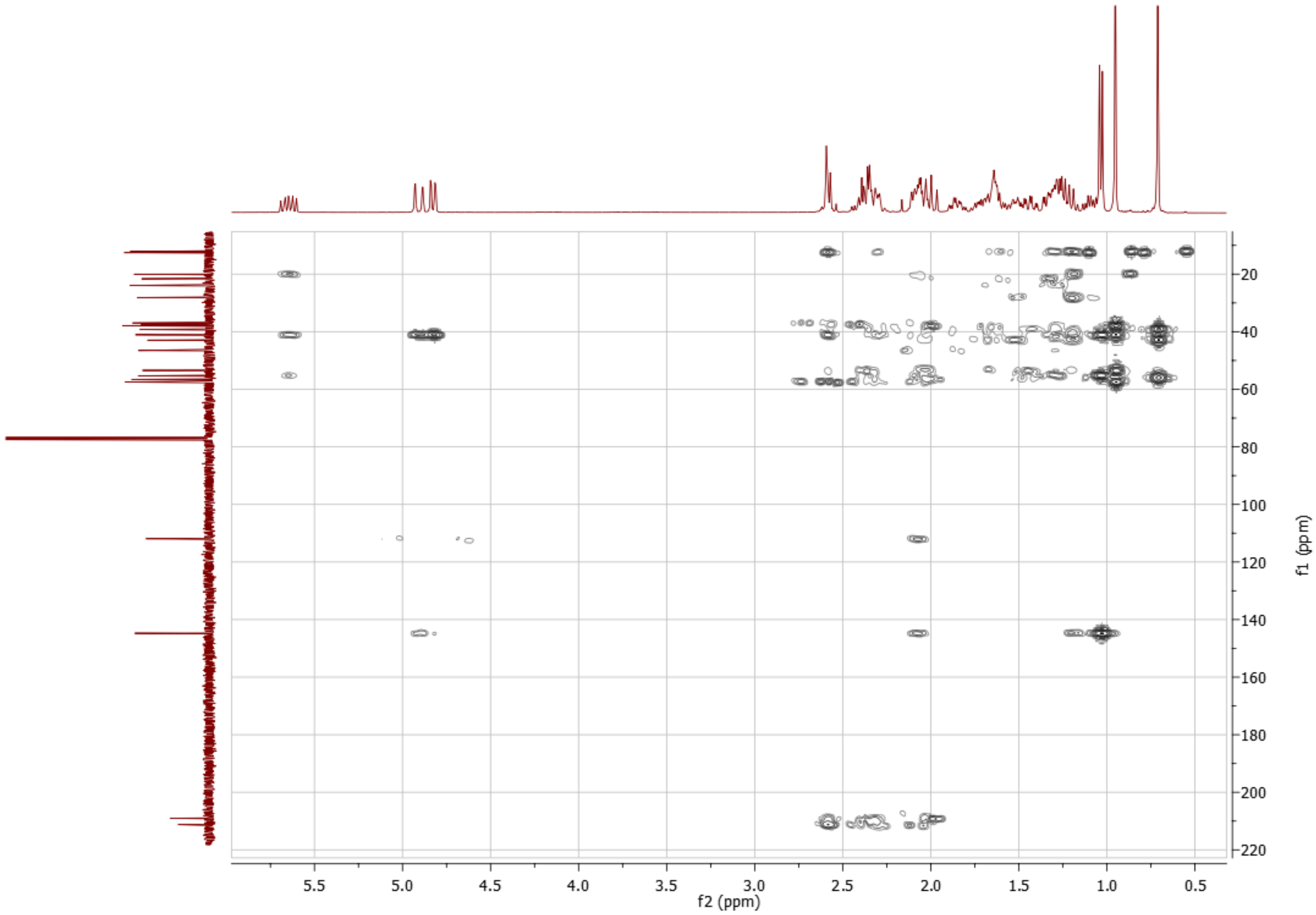

S15. 2D HMBC NMR spectrum of *24-nor-5α-chol-22-ene-3,6-dione* (**37**).

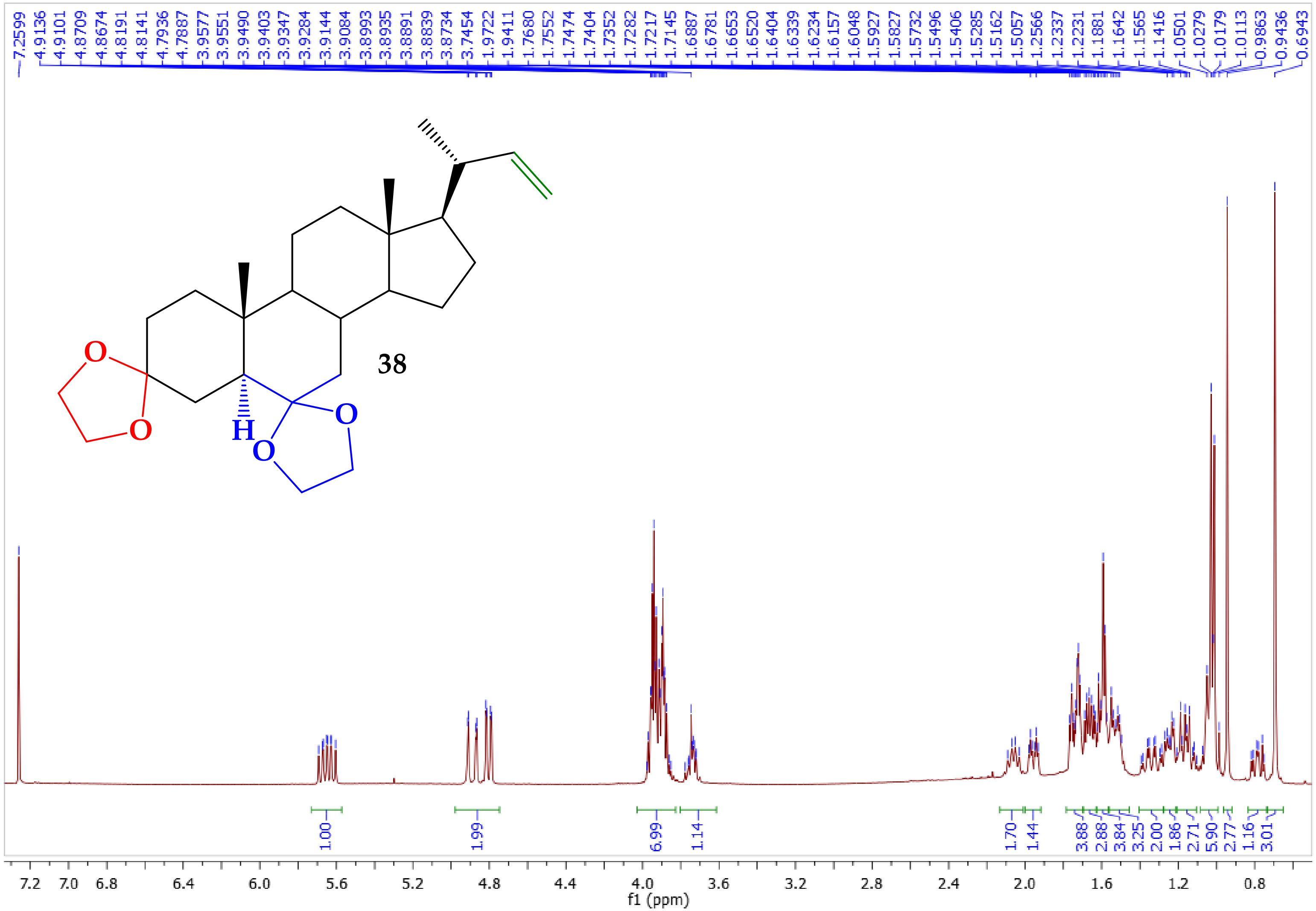

S16. ^{1}H NMR spectrum of *3,6-didioxolan-24-nor-5α-cholan-22-ene* (**38**).

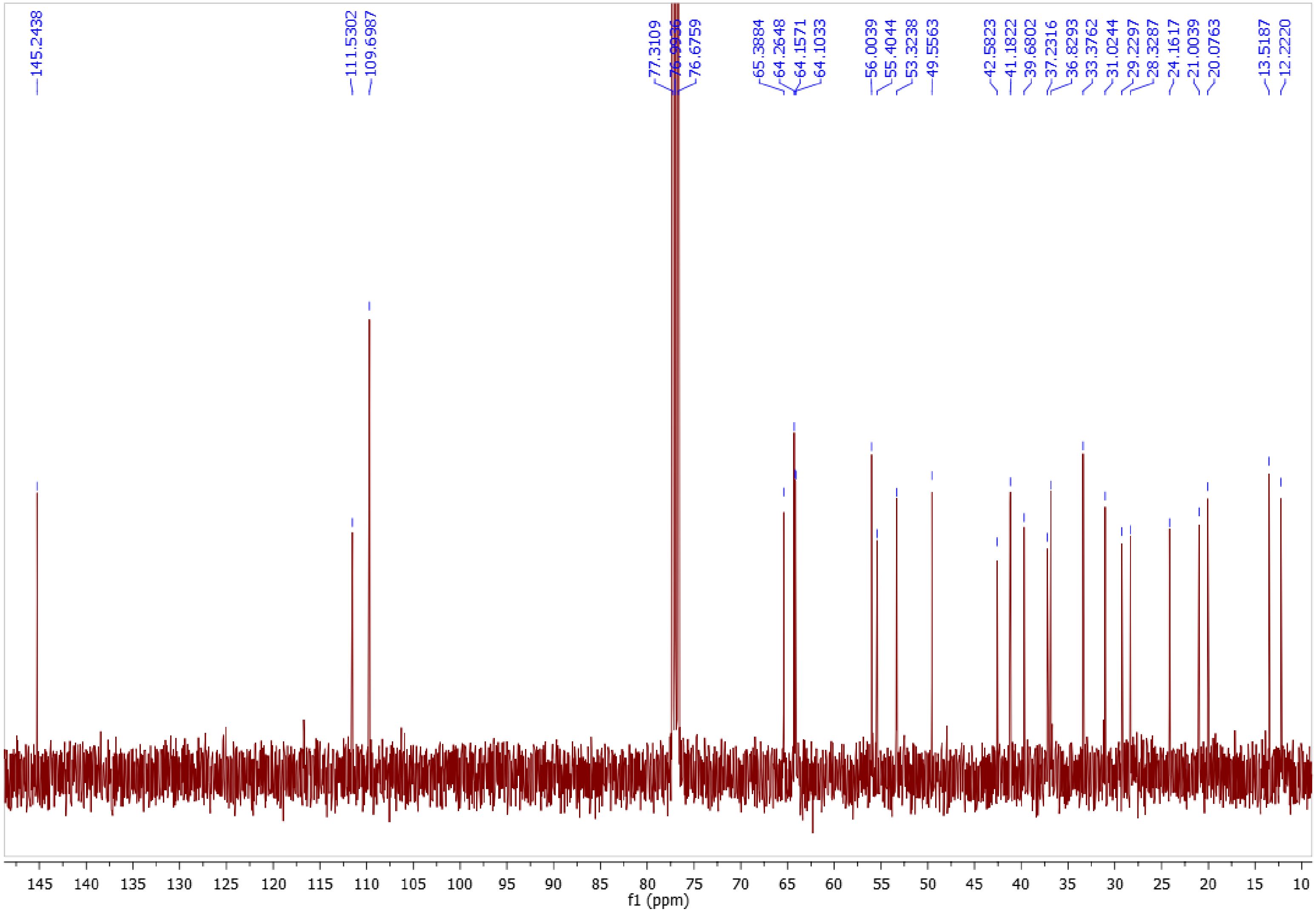

S17. ^{13}C NMR spectrum of *3,6-didioxolan-24-nor-5α-cholan-22-ene* (***38***).

.

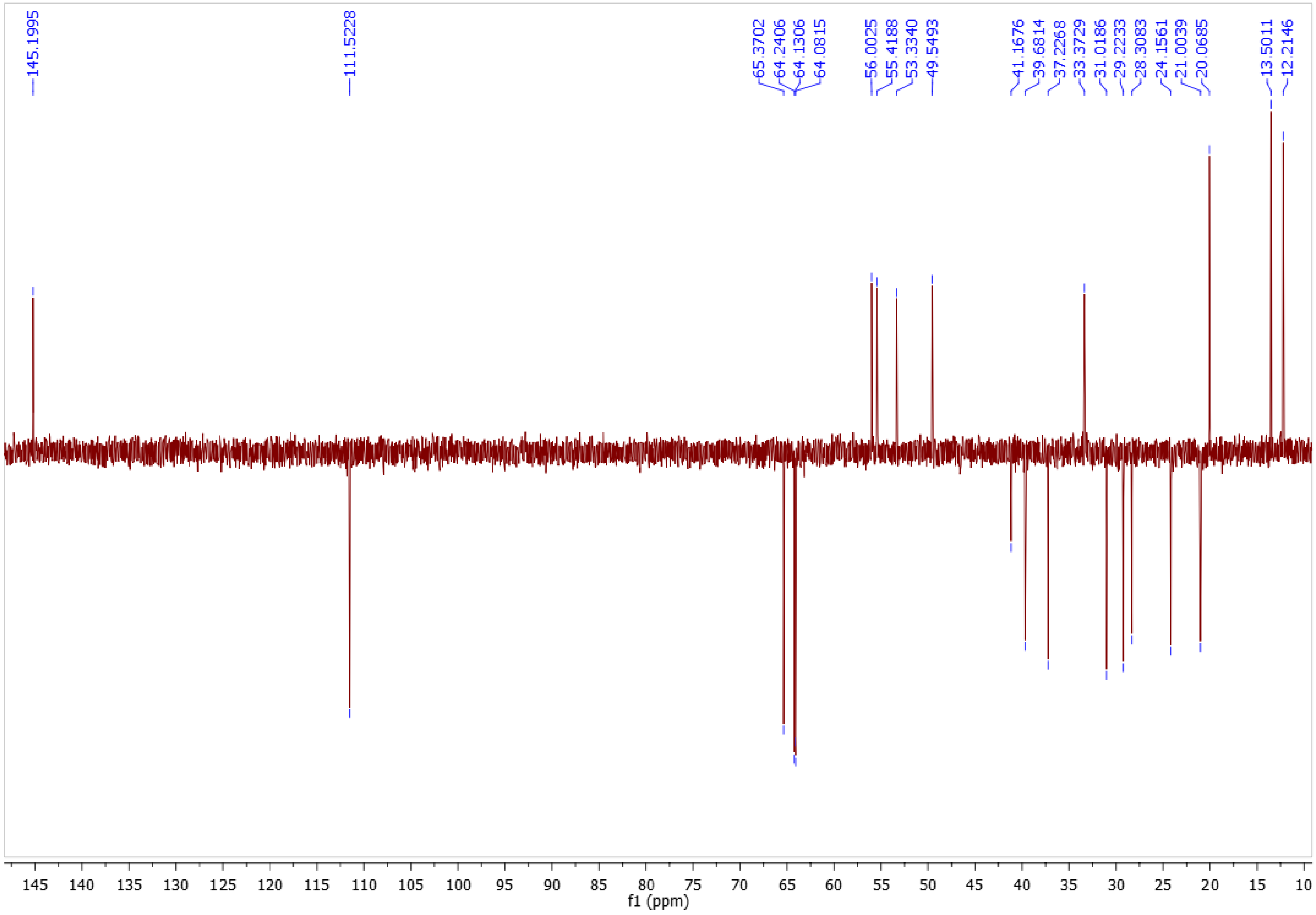

S18. ^{13}C DEPT-135 NMR spectrum of *3,6-didioxolan-24-nor-5α-cholan-22-ene* (**38**).

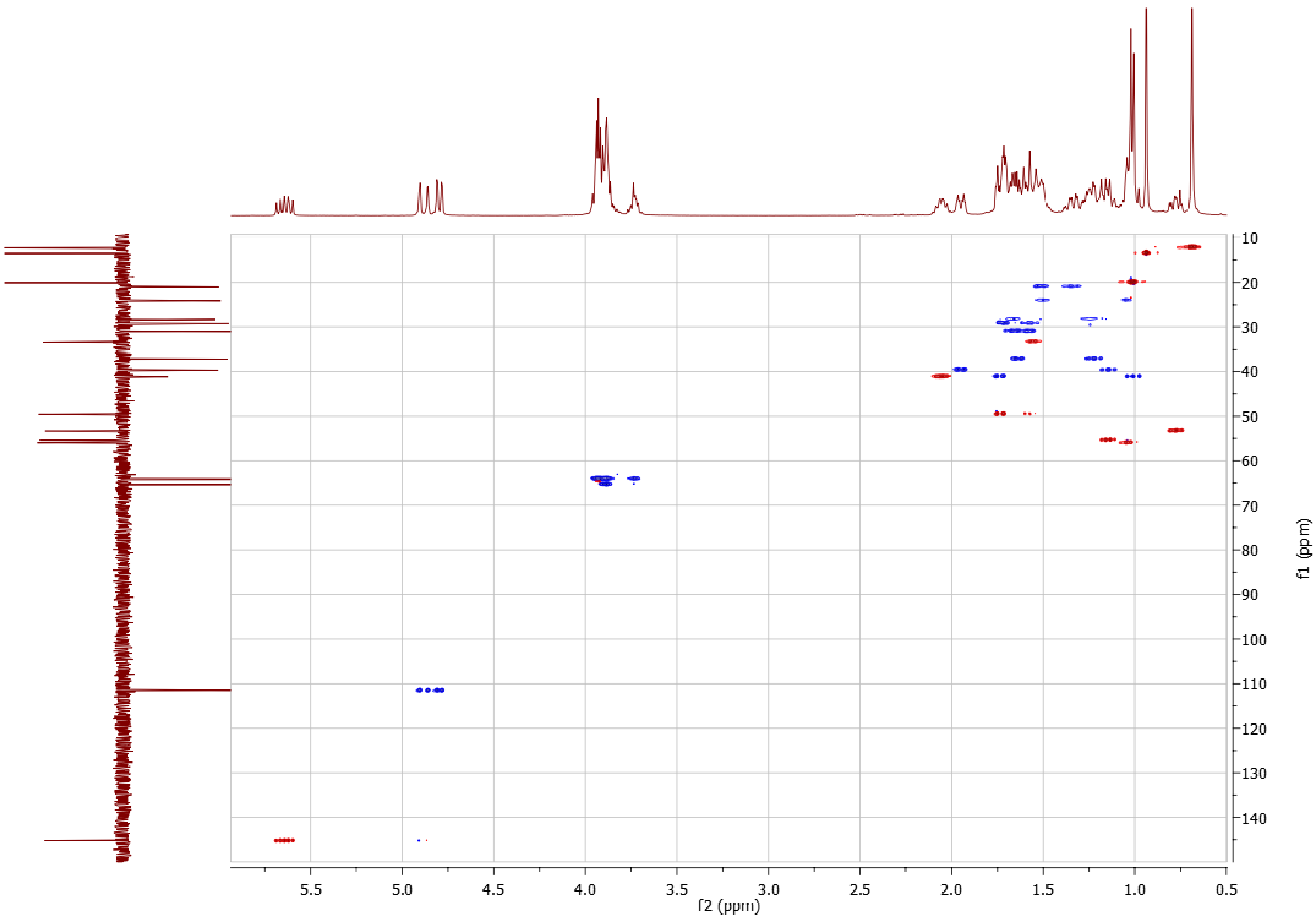

S19. 2D HSQC NMR spectrum of *3,6-didioxolan-24-nor-5α-cholan-22-ene (**38**)*.

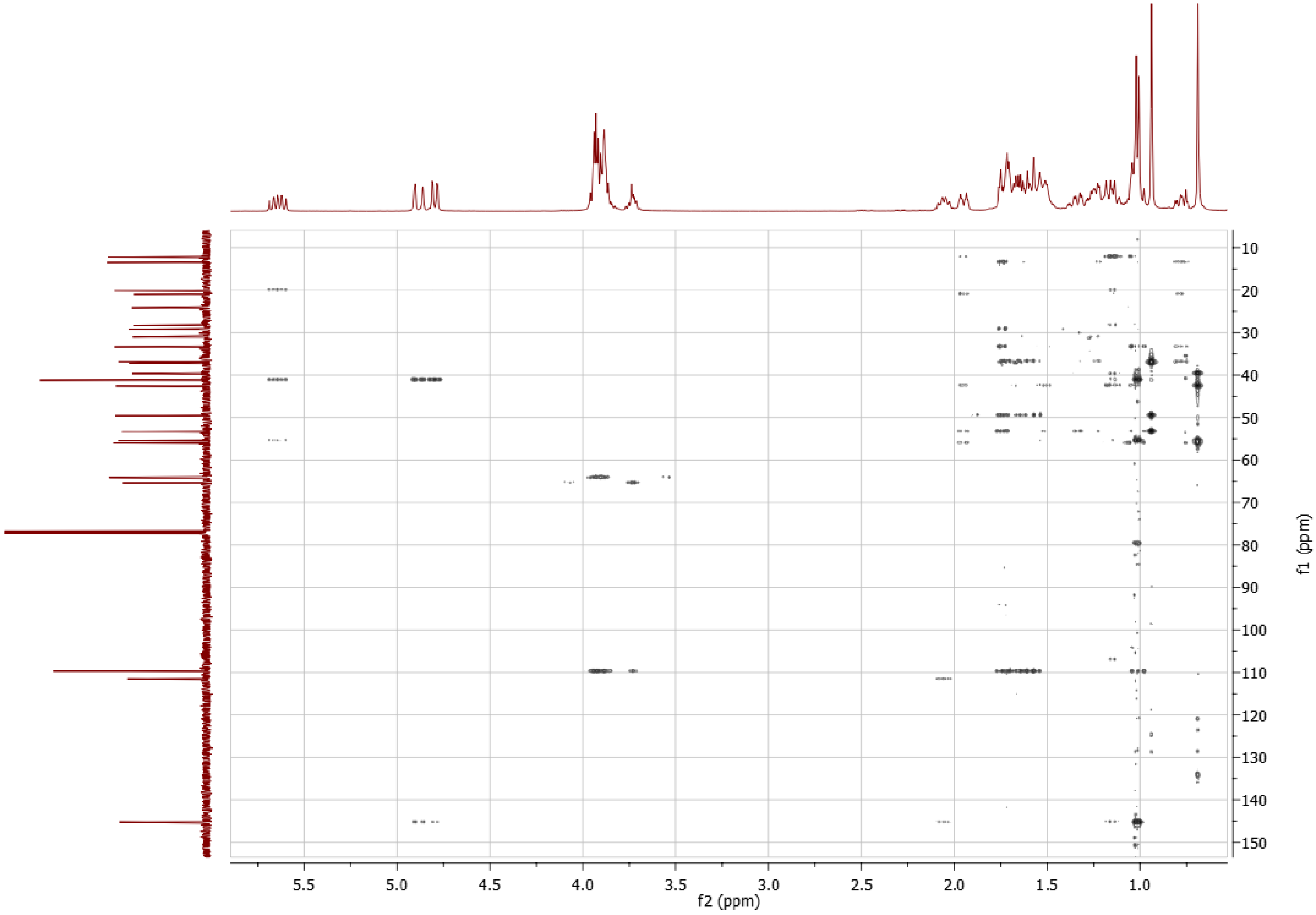

S20. 2D HMBC NMR spectrum of *3,6-didioxolan-24-nor-5α-cholan-22-ene (**38**).*

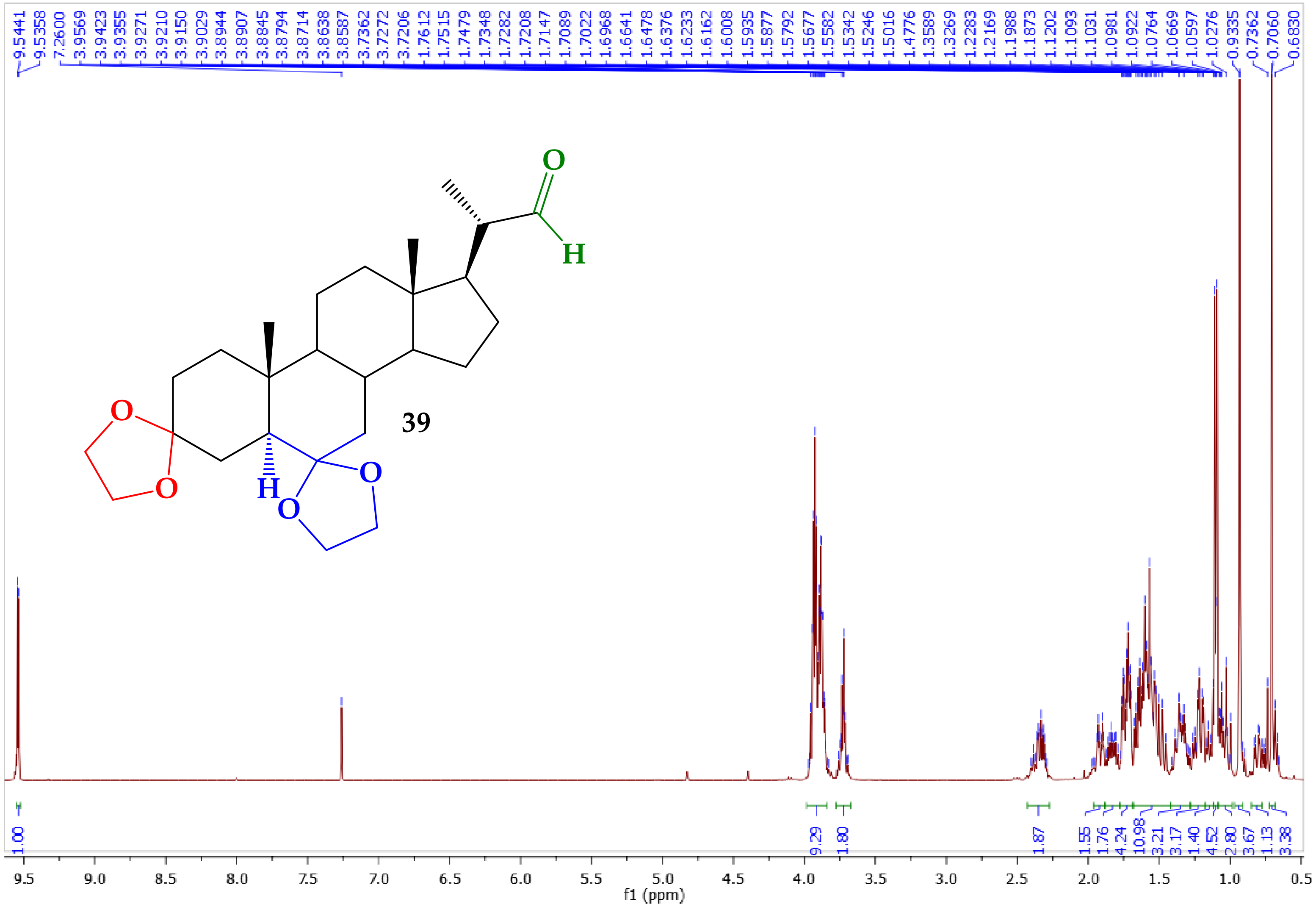

S21. ^{1}H NMR spectrum of *3,6-didioxolan-23,24-dinor-5α-cholan-22-al* (***39***).

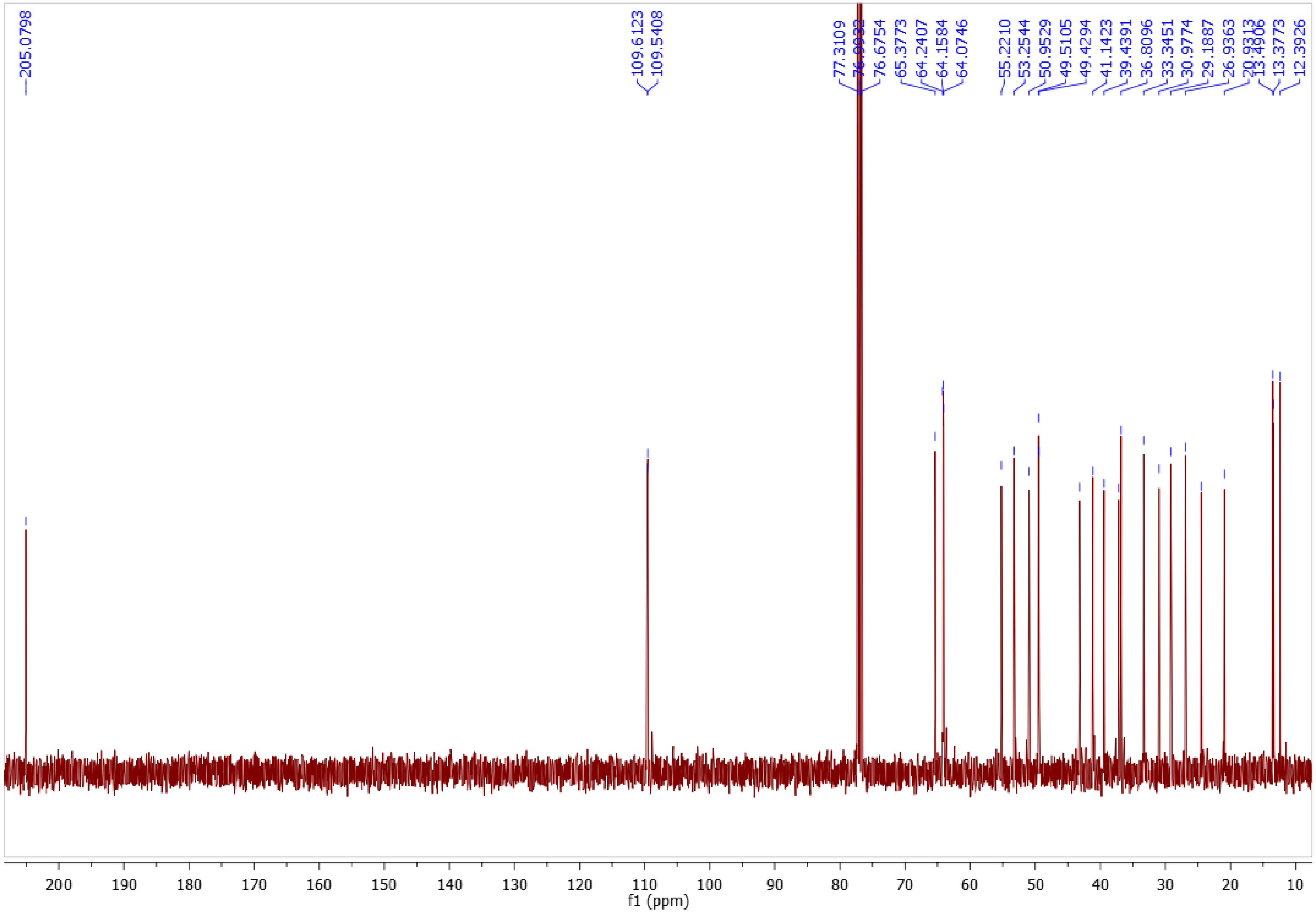

S22. ^{13}C NMR spectrum of *3,6-didioxolan-23,24-dinor-5α-cholan-22-al* (***39***).

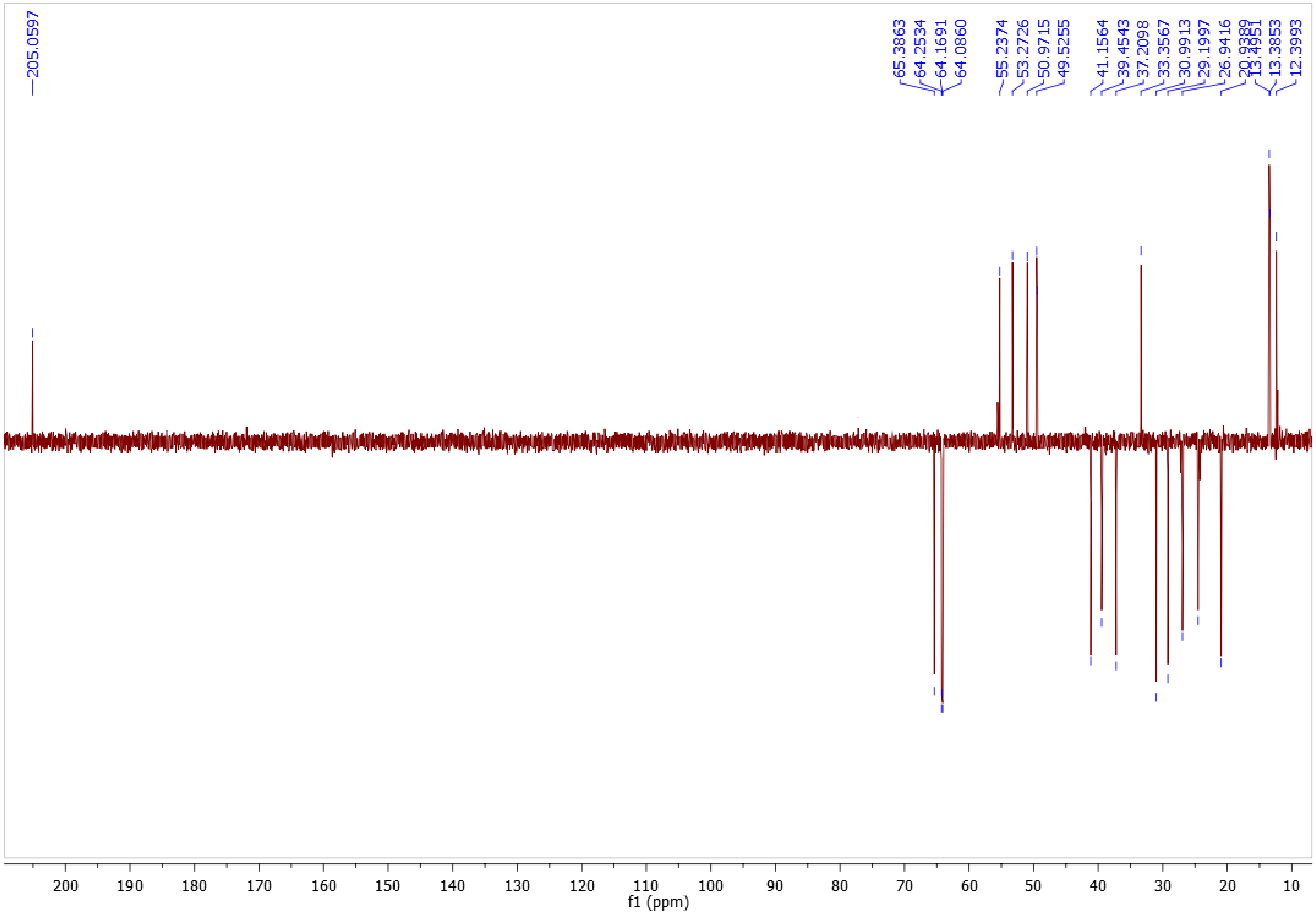

S23. ^{13}C DEPT-135 NMR spectrum of *3,6-didioxolan-23,24-dinor-5α-cholan-22-al* (**39**).

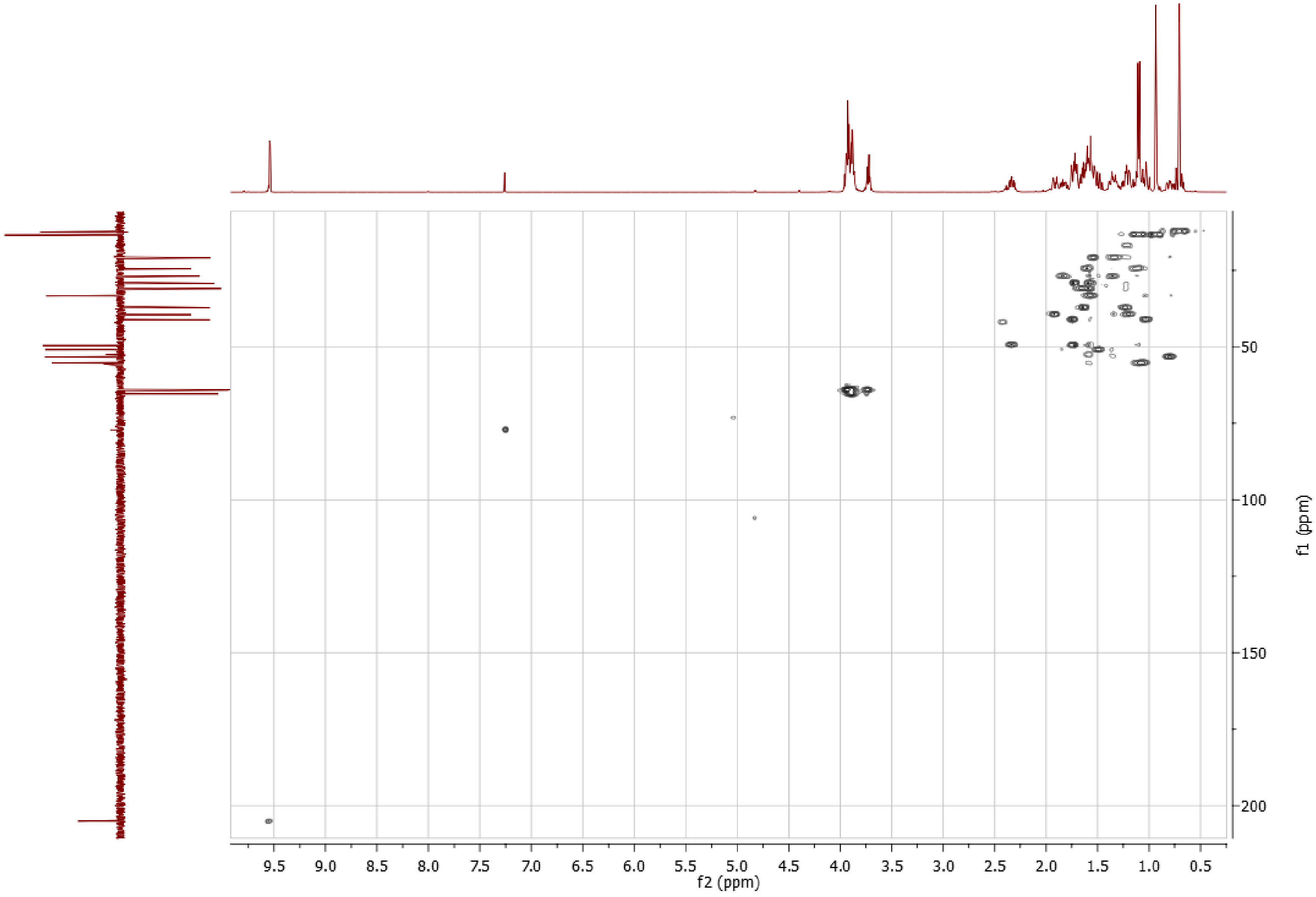

S24. 2D HSQC NMR spectrum of *3,6-didioxolan-23,24-dinor-5α-cholan-22-al* (***39***).

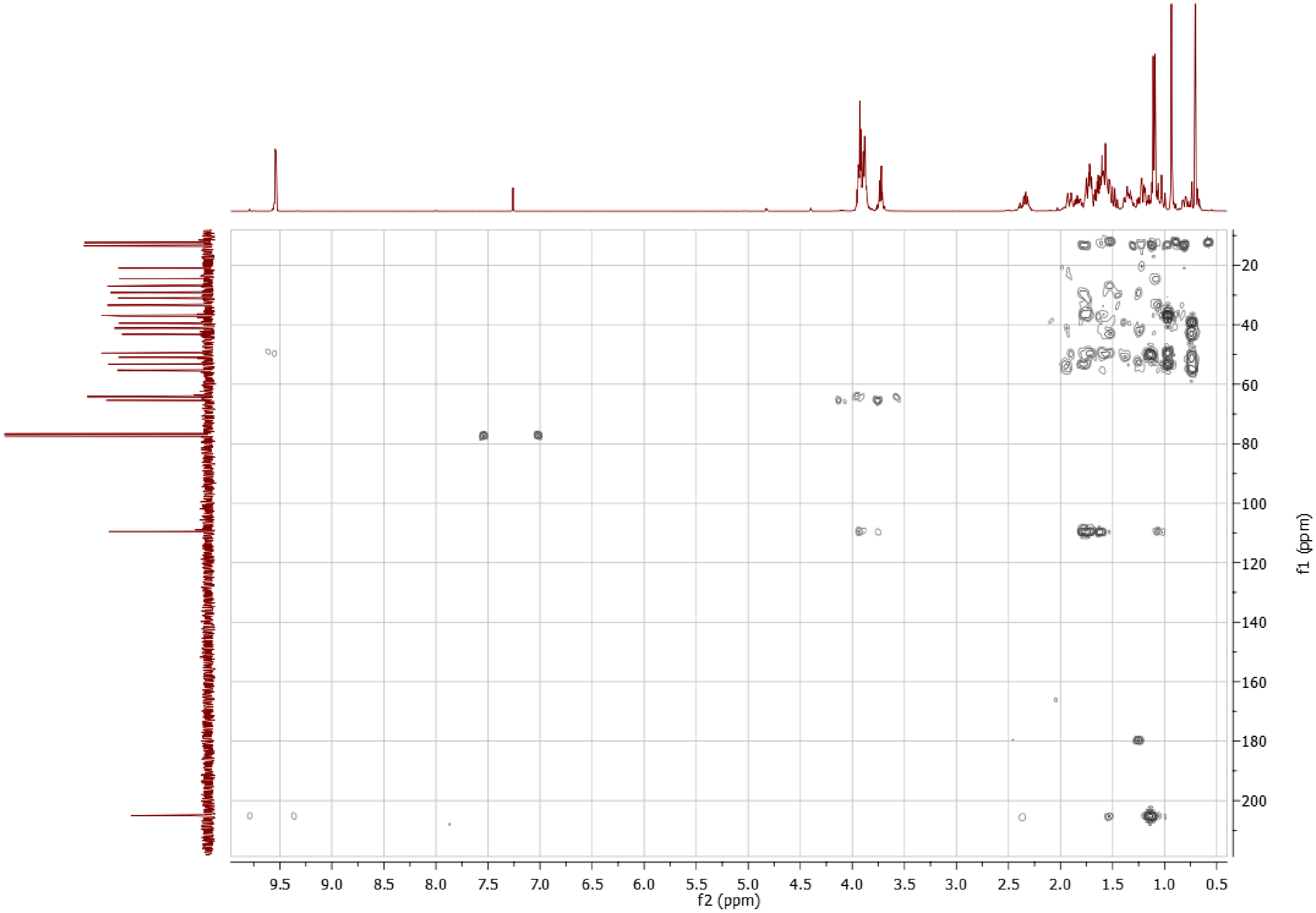

S25. 2D HMBC NMR spectrum of *3,6-didioxolan-23,24-dinor-5α-cholan-22-al* (***39***).

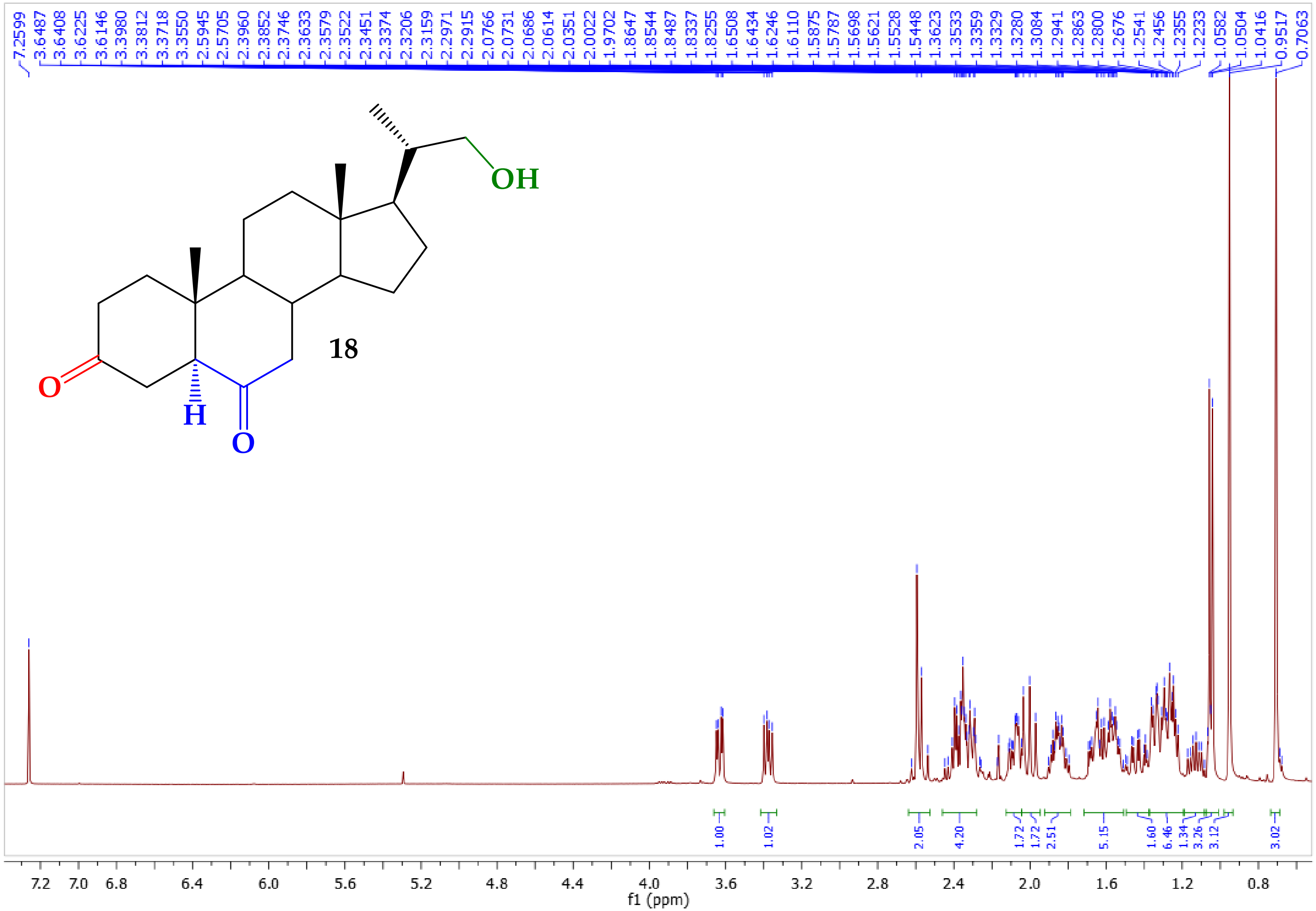

S26. ^{1}H NMR spectrum of *22-hydroxy-23,24-dinor-5α-cholan-3,6-dione* (***18***).

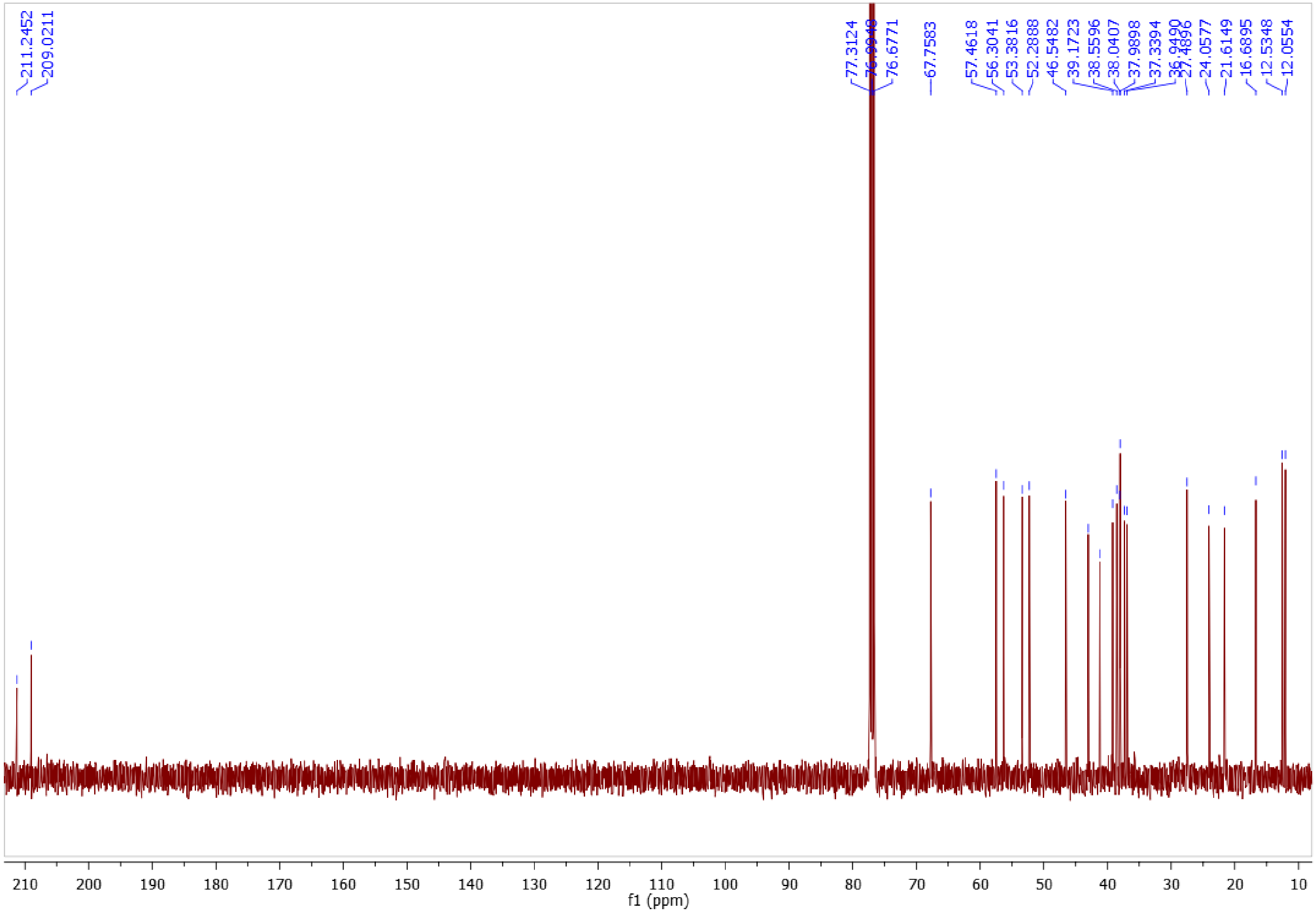

S27. ^{13}C NMR spectrum of *22-hydroxy-23,24-dinor-5α-cholan-3,6-dione* (***18***).

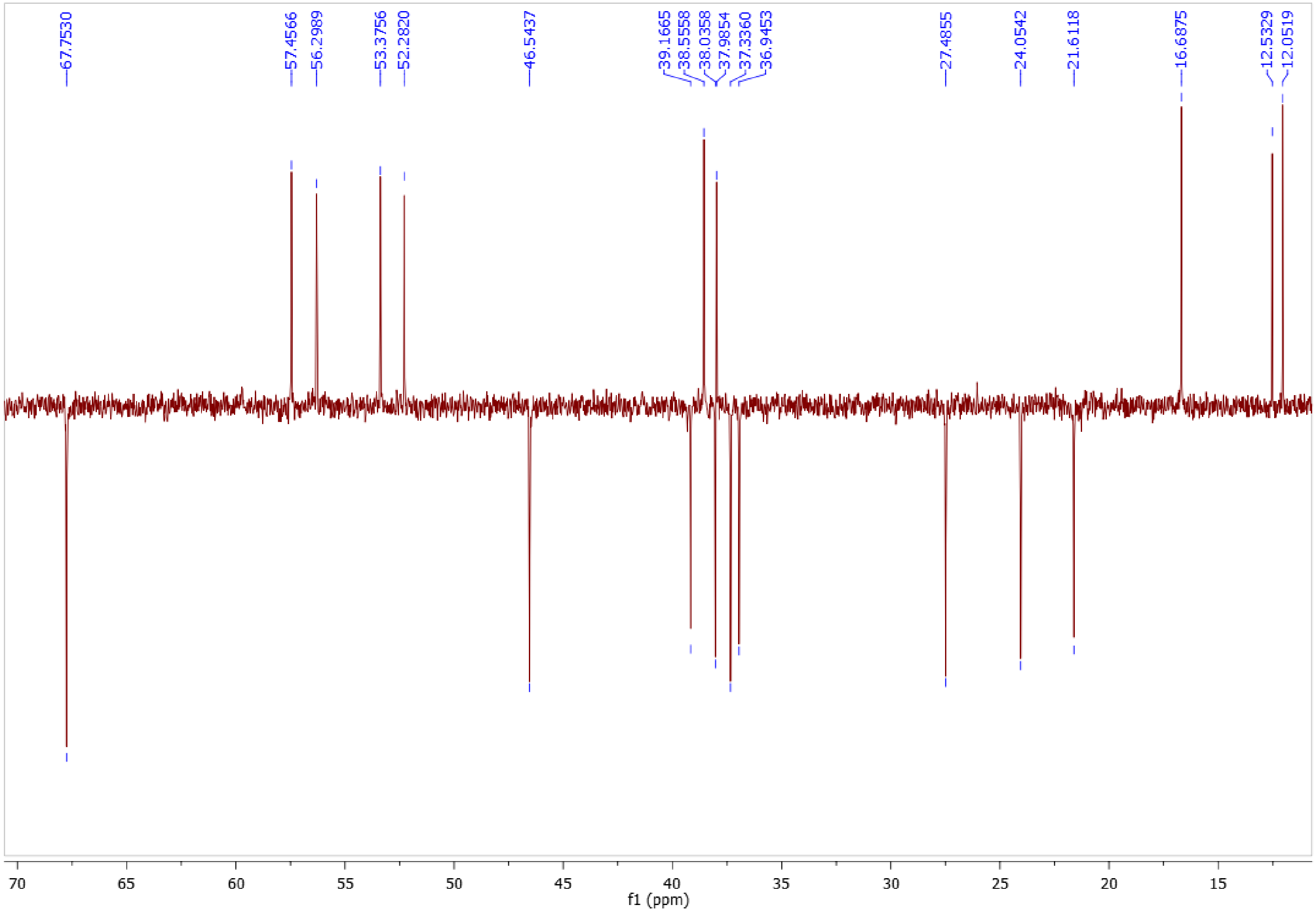

S28. ^{13}C NMR DEPT-135 spectrum of *22-hydroxy-23,24-dinor-5α-cholan-3,6-dione* (***18***).

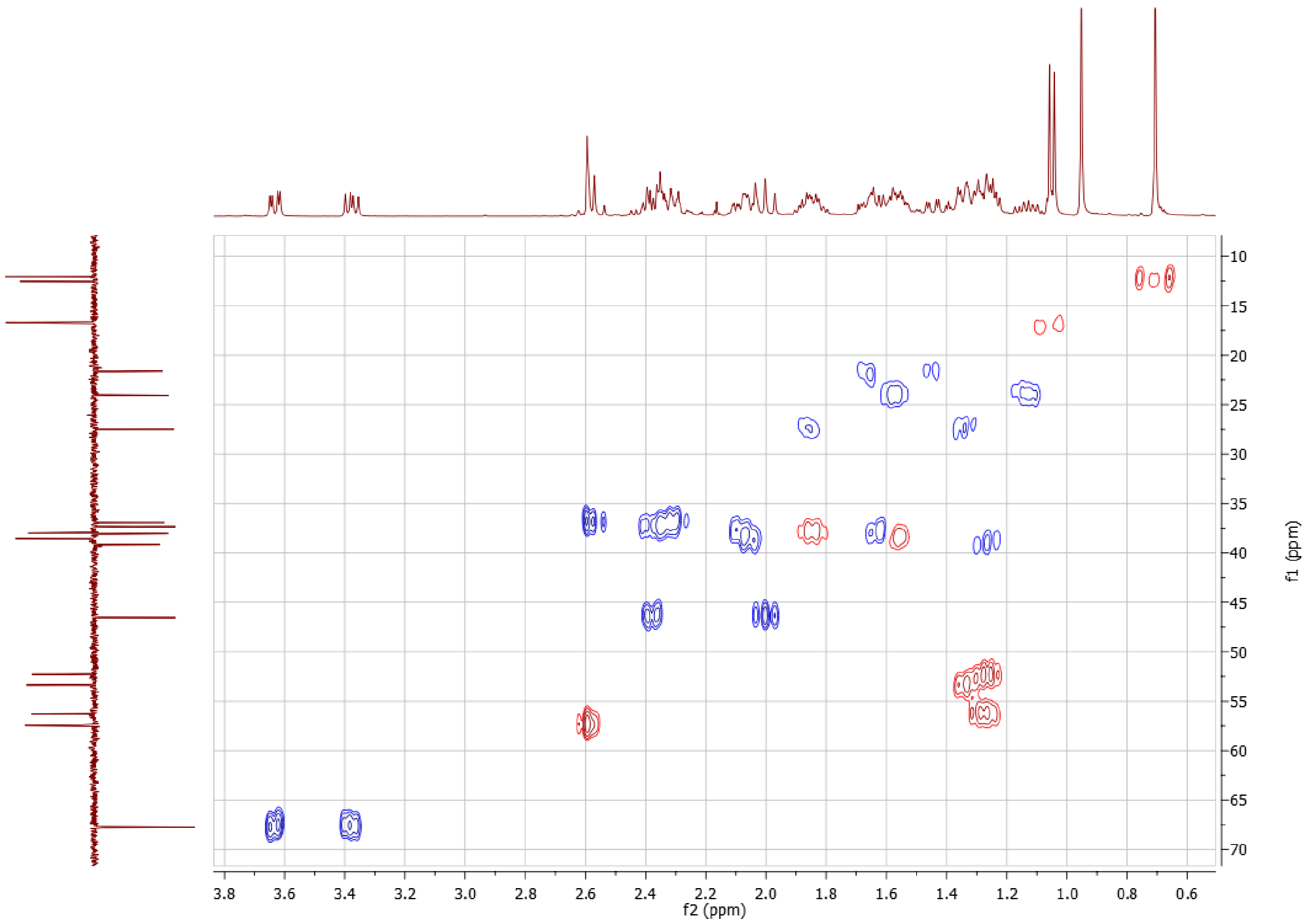

S29. 2D HSQC spectrum of *22-hydroxy-23,24-dinor-5α-cholan-3,6-dione* (***18***).

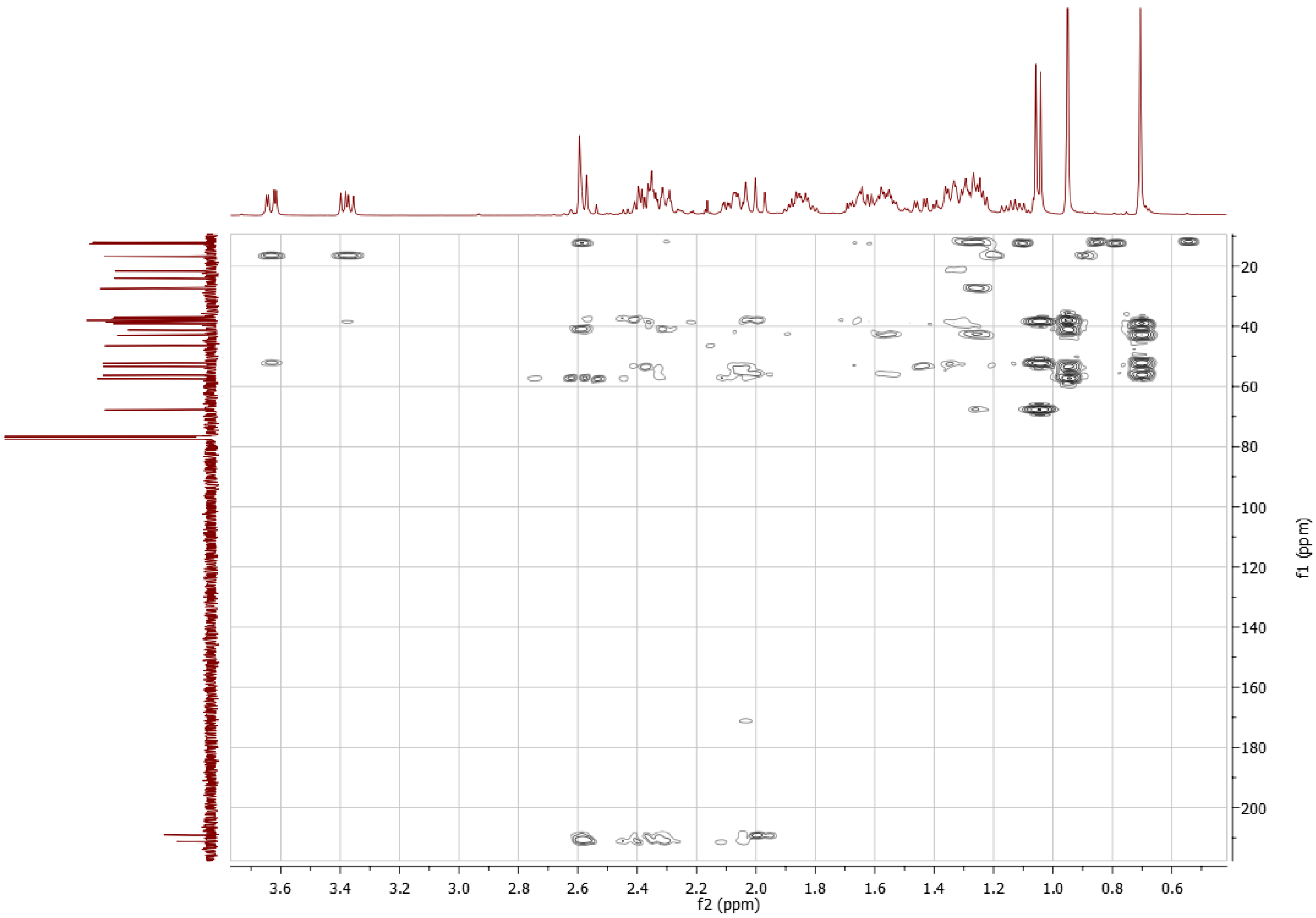

S30. 2D HMBC spectrum of *22-hydroxy-23,24-dinor-5α-cholan-3,6-dione* (***18***).

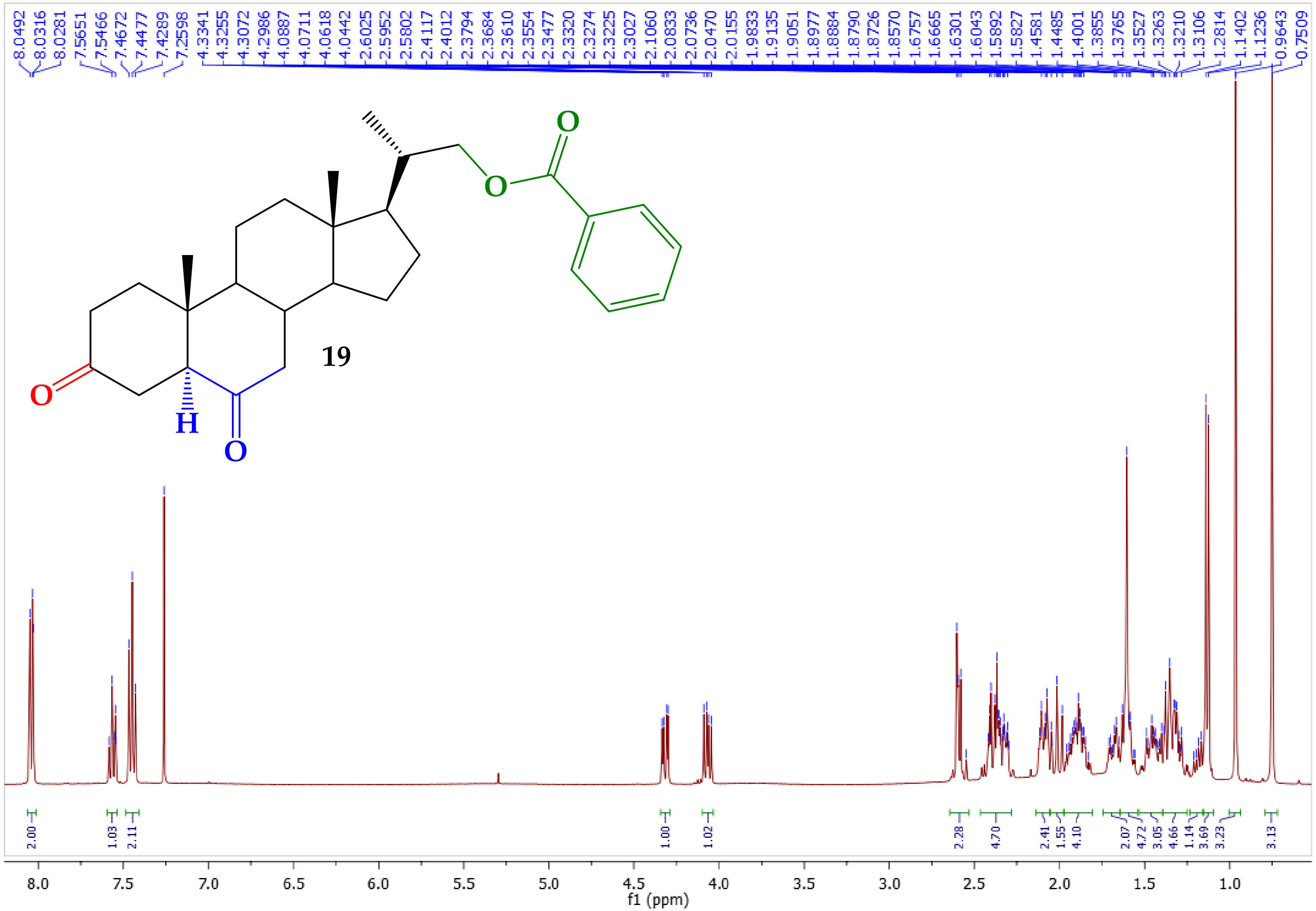

S31. ^{1}H NMR spectrum of *3,6-dioxo-23,24-dinor-5α-cholan-benzoate-22-yl (**19**)*.

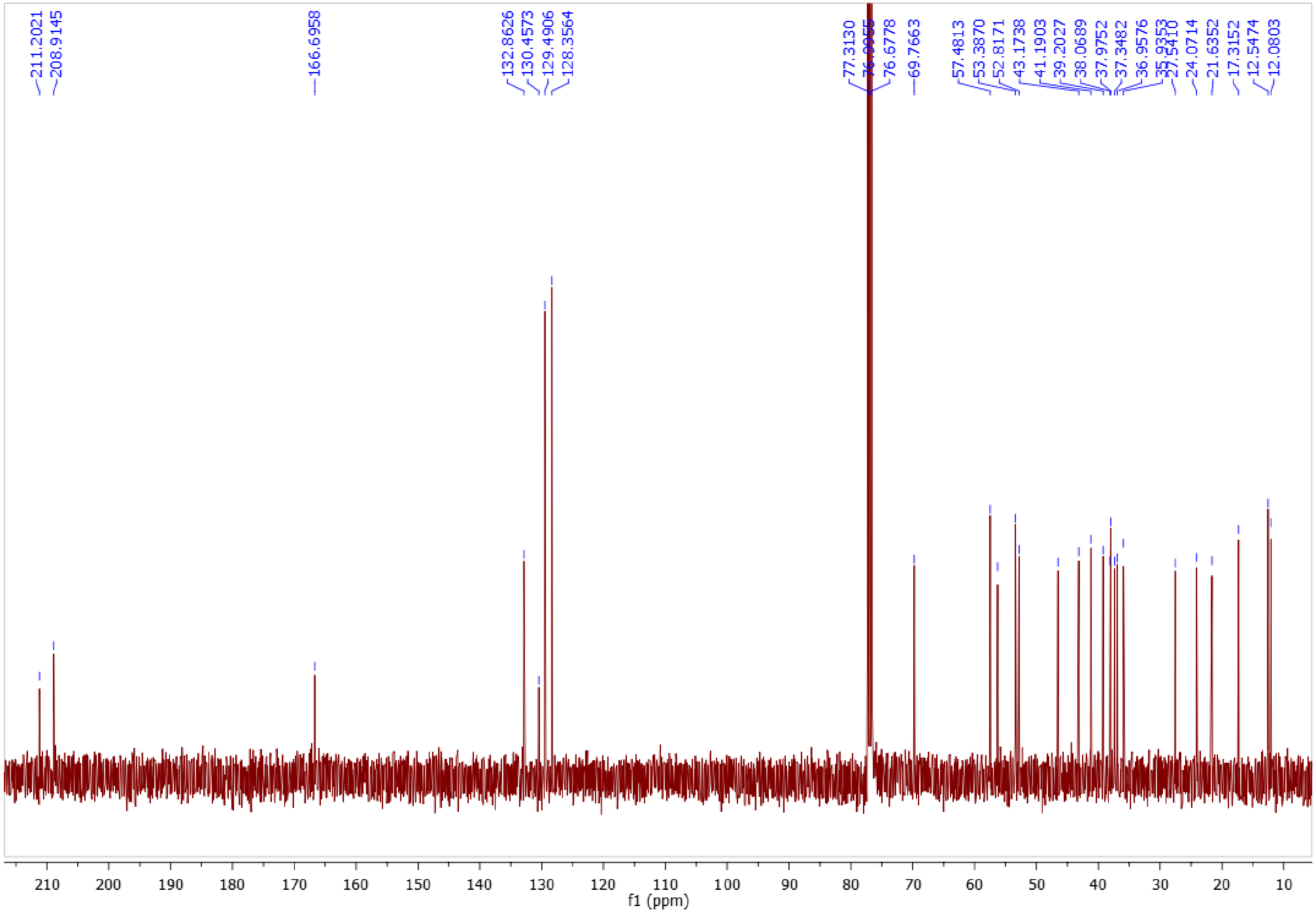

S32. ^{13}C NMR spectrum of *3,6-dioxo-23,24-dinor-5α-cholan-benzoate-22-yl* (***19***).

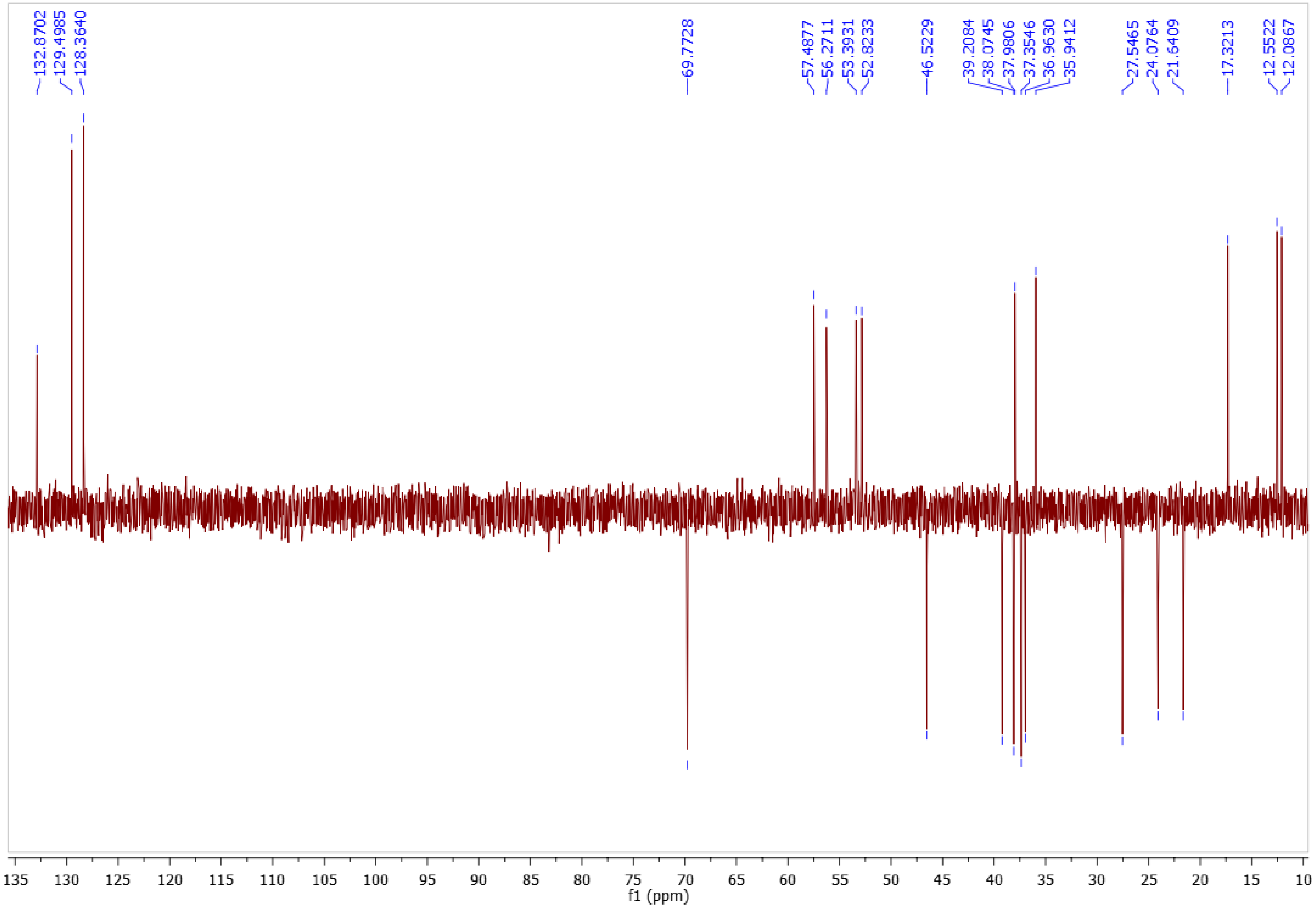

S33. ^{13}C DEPT-135 NMR spectrum of *3,6-dioxo-23,24-dinor-5α-cholan-benzoate-22-yl* (***19***).

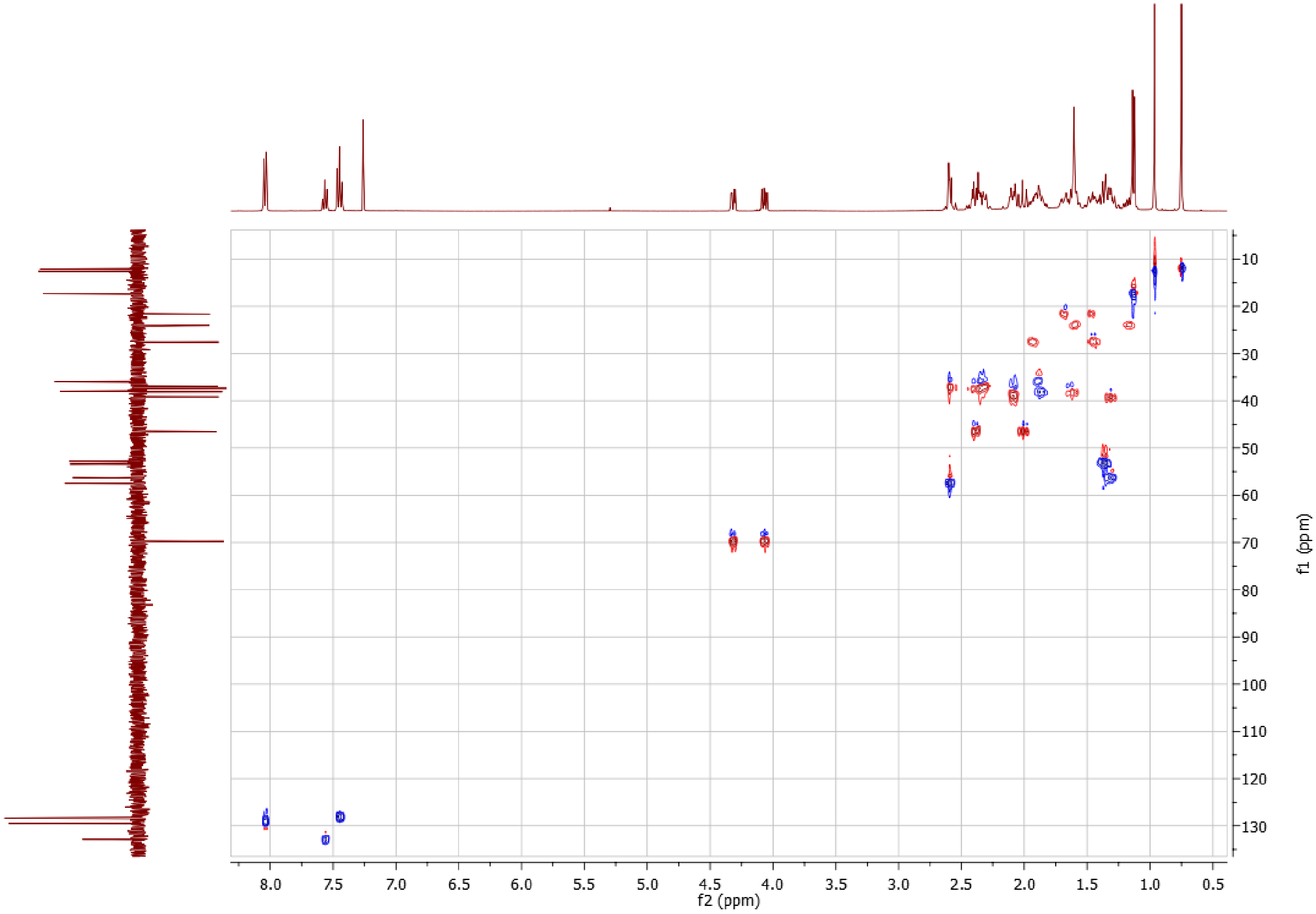

S34. 2D HSQC NMR spectrum of *3,6-dioxo-23,24-dinor-5α-cholan-benzoate-22-yl (**19**)*.

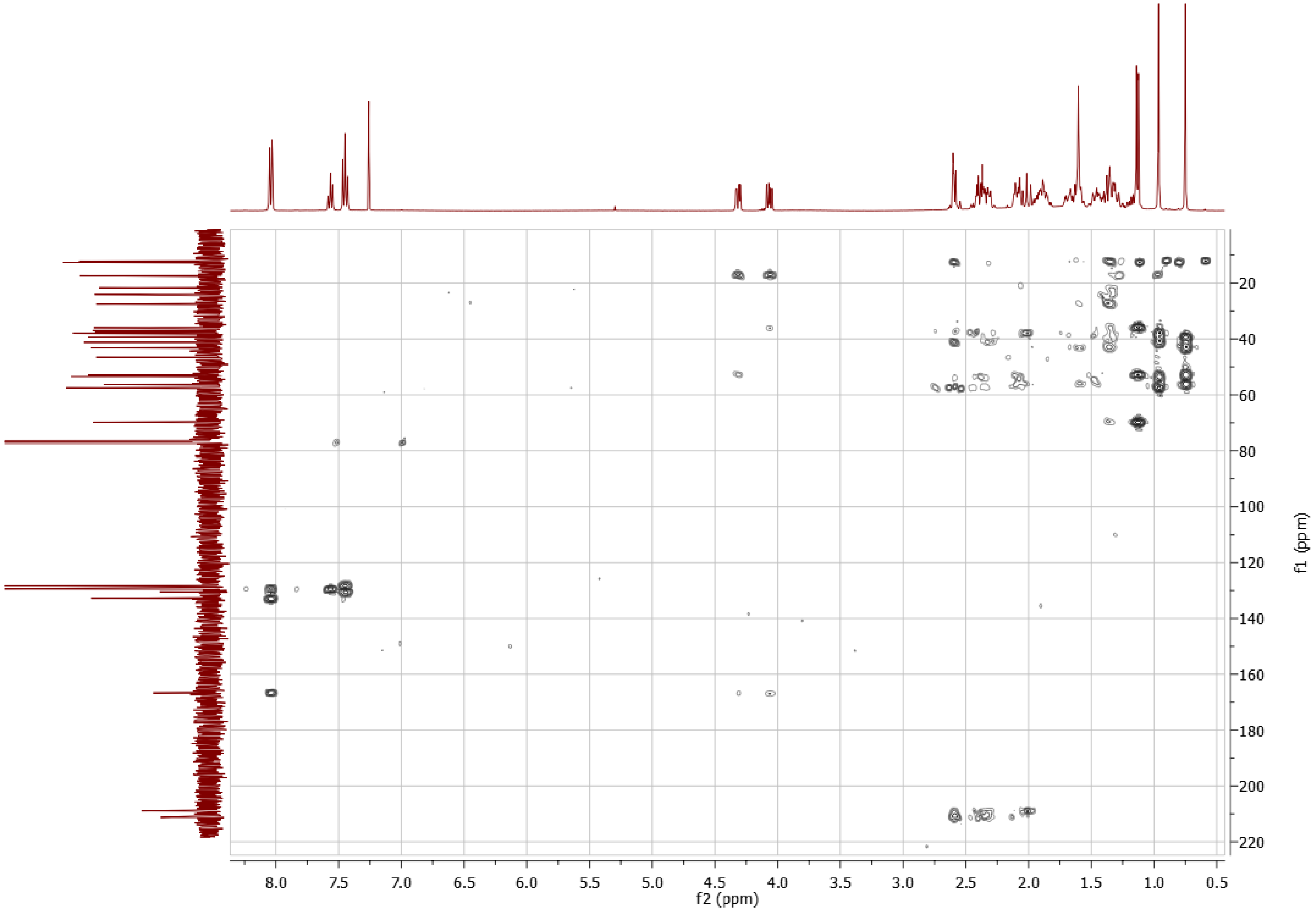

S35. 2D HMBC NMR spectrum of *3,6-dioxo-23,24-dinor-5α-cholan-benzoate-22-yl (**19**)*.

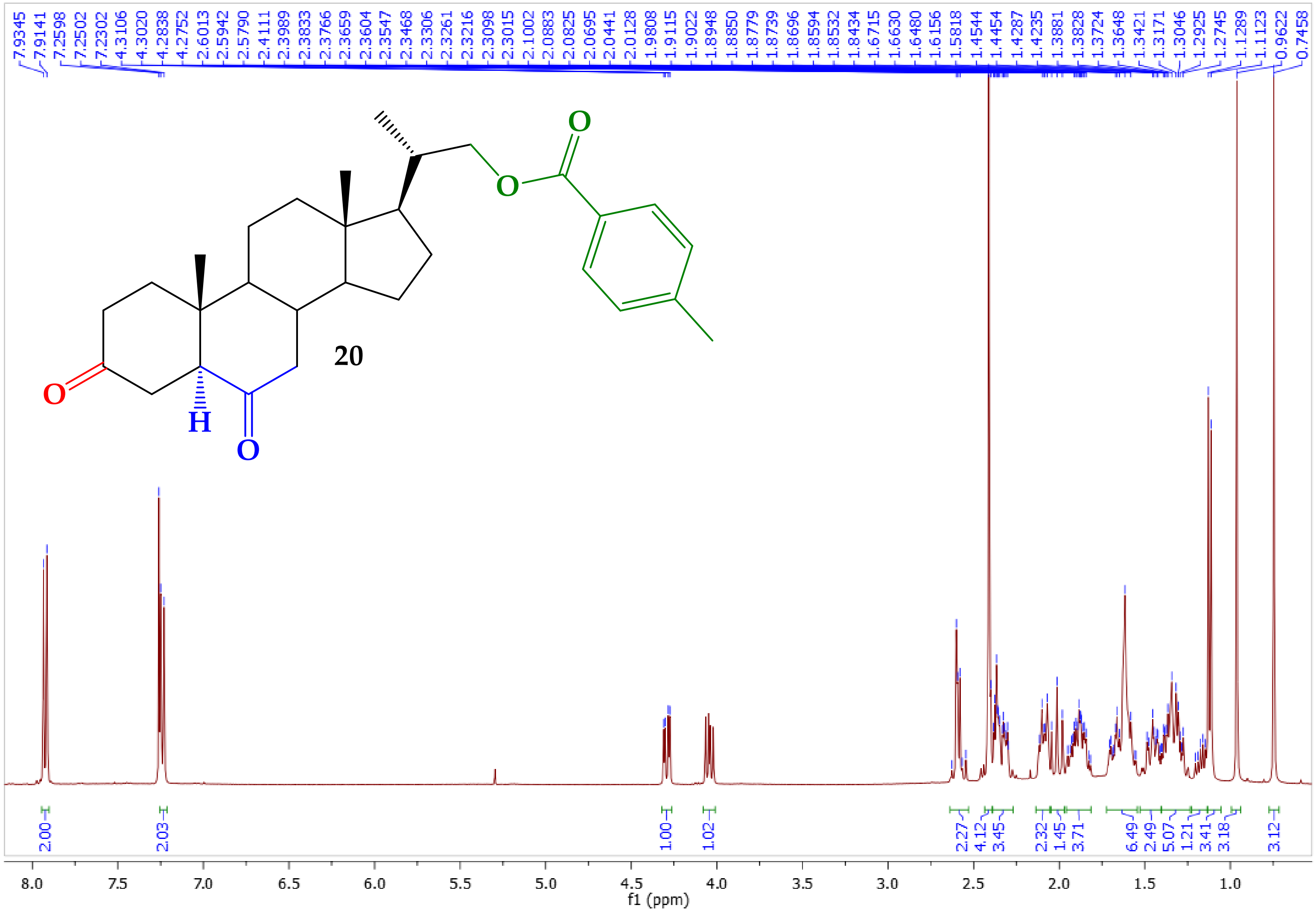

S36. ^{1}H NMR spectrum of *3,6-dioxo-23,24-dinor-5α-cholan-(4-methyl)-benzoate-22-yl* (**20**).

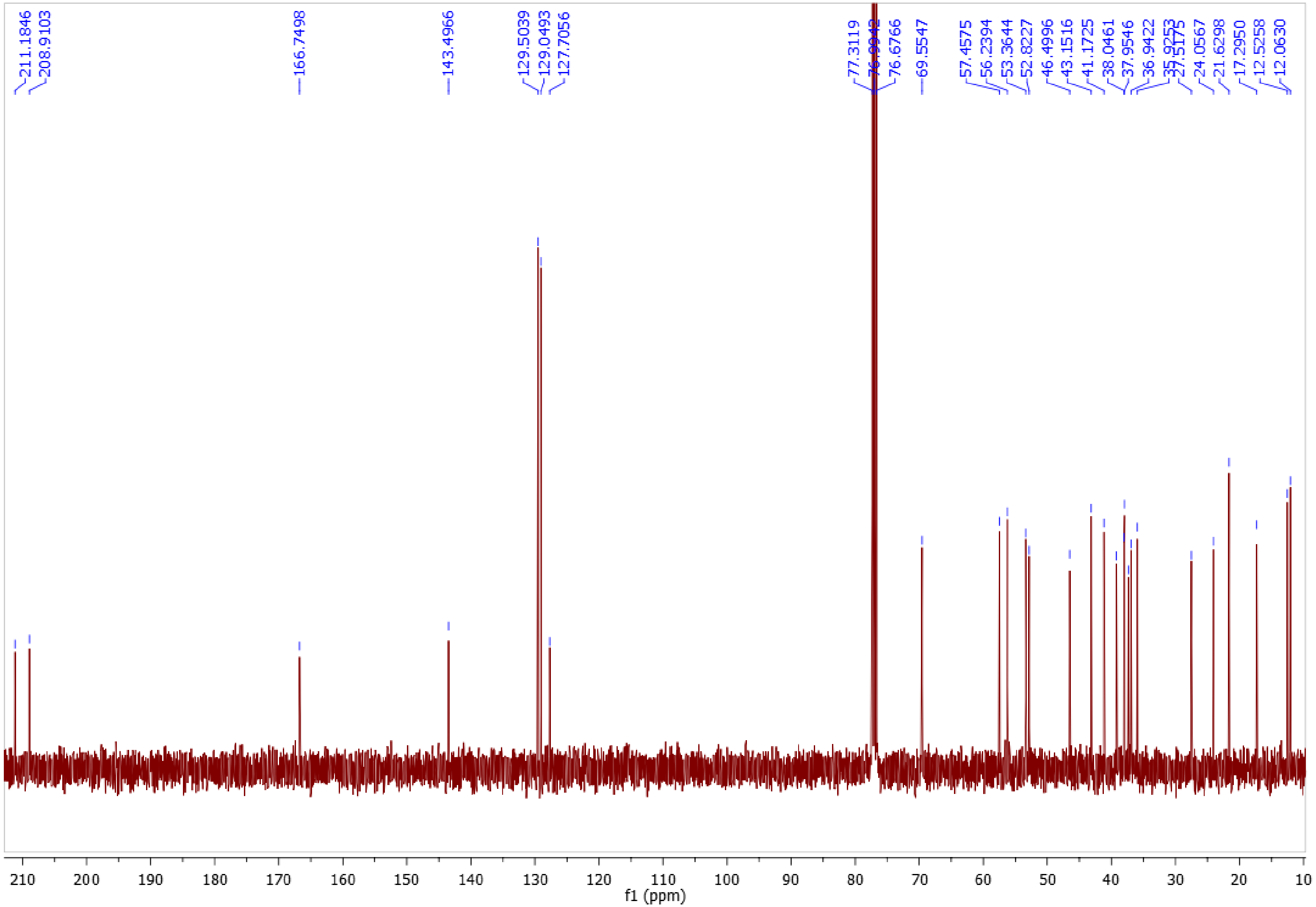

S37. ^{13}C NMR spectrum of *3,6-dioxo-23,24-dinor-5α-cholan-(4-methyl)-benzoate-22-yl* (**20**).

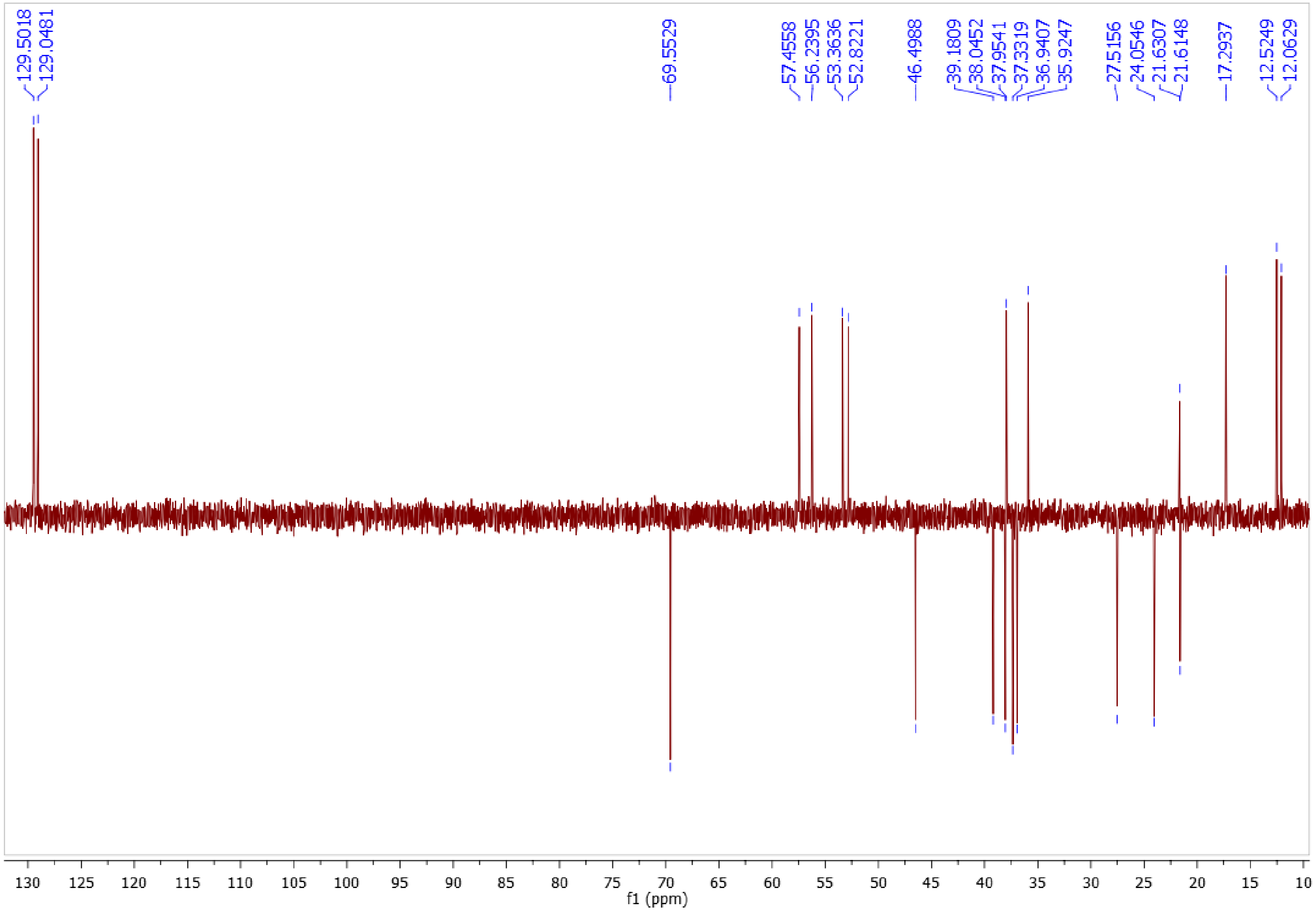

S38. ^{13}C DEPT-135 NMR spectrum of *3,6-dioxo-23,24-dinor-5α-cholan-(4-methyl)-benzoate-22-yl* (**20**).

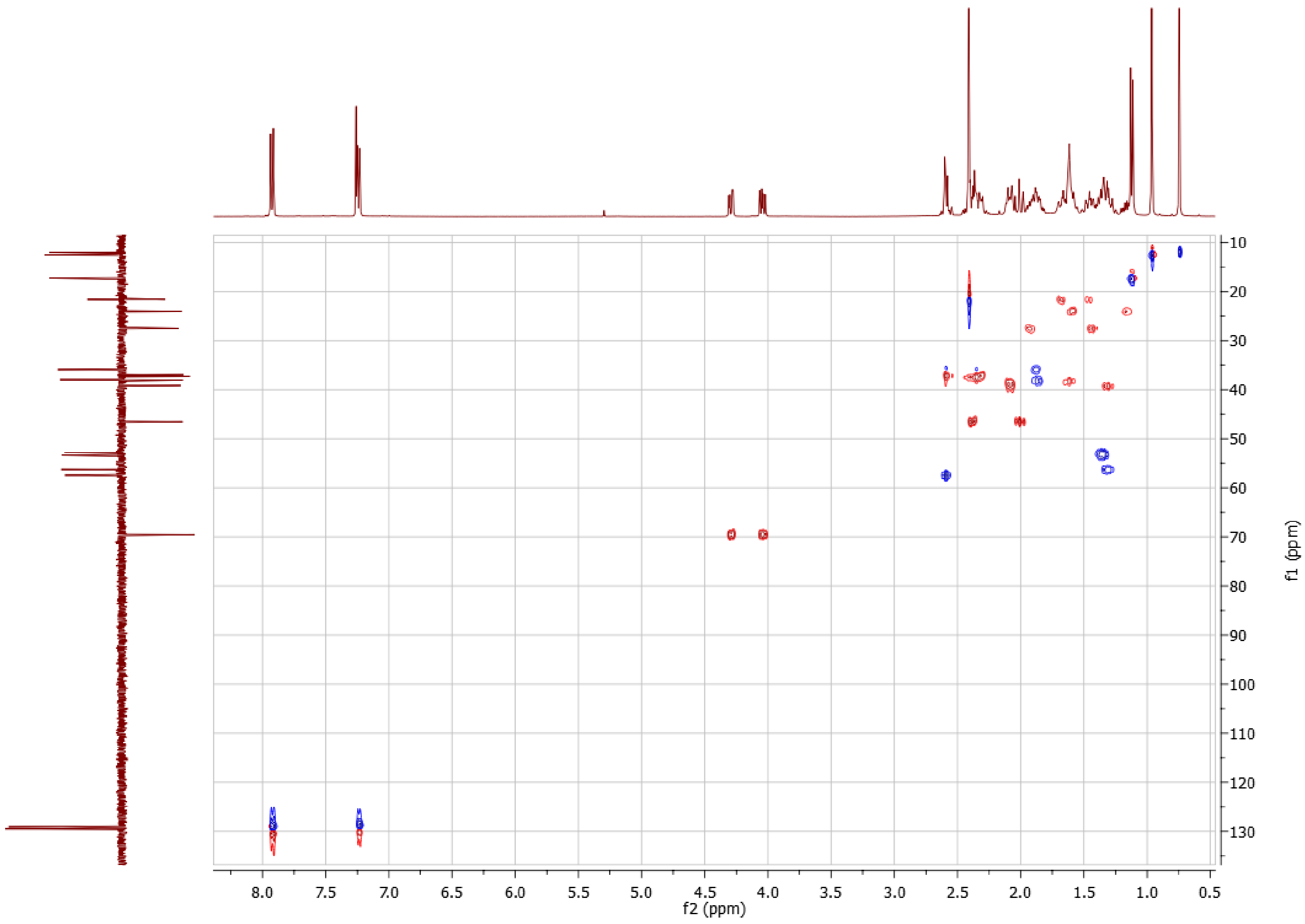

S39. 2D HSQC NMR spectrum of *3,6-dioxo-23,24-dinor-5α-cholan-(4-methyl)-benzoate-22-yl* (**20**).

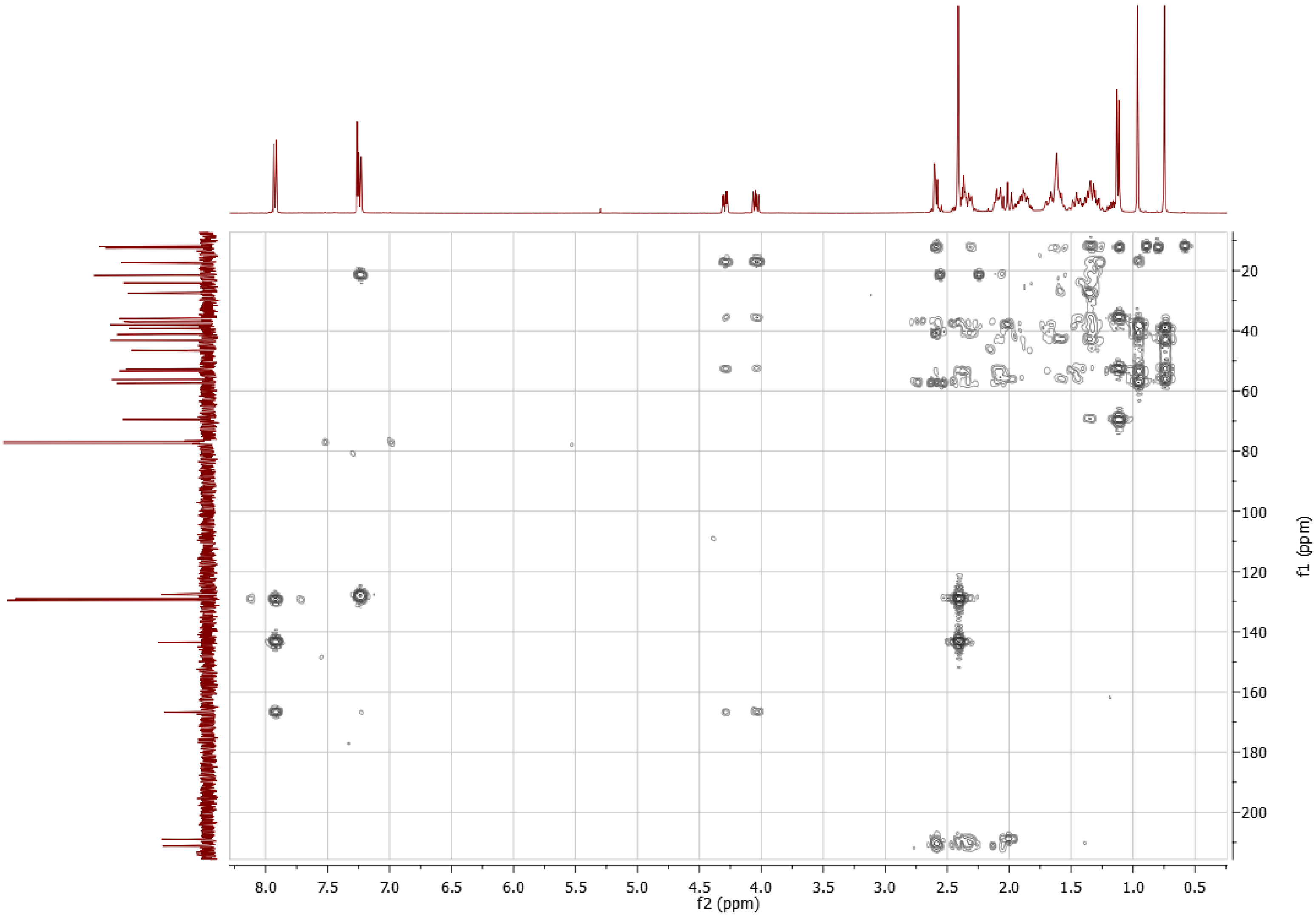

S40. 2D HMBC NMR spectrum of *3,6-dioxo-23,24-dinor-5α-cholan-(4-methyl)-benzoate-22-yl* (**20**).

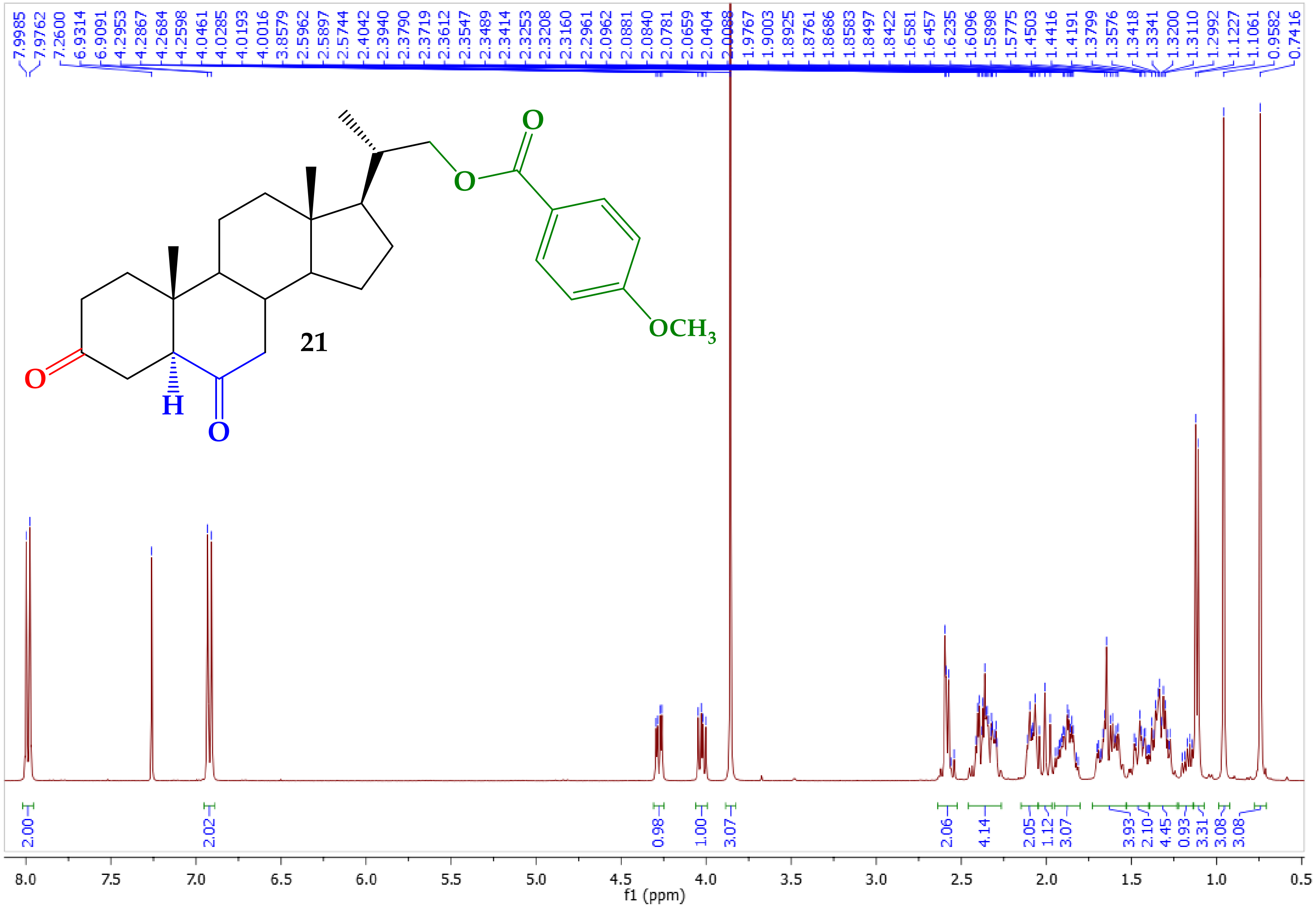

S41. ^{1}H NMR spectrum of *3,6-dioxo-23,24-dinor-5α-cholan-(4-methoxy)-benzoate-22-yl* (**21**).

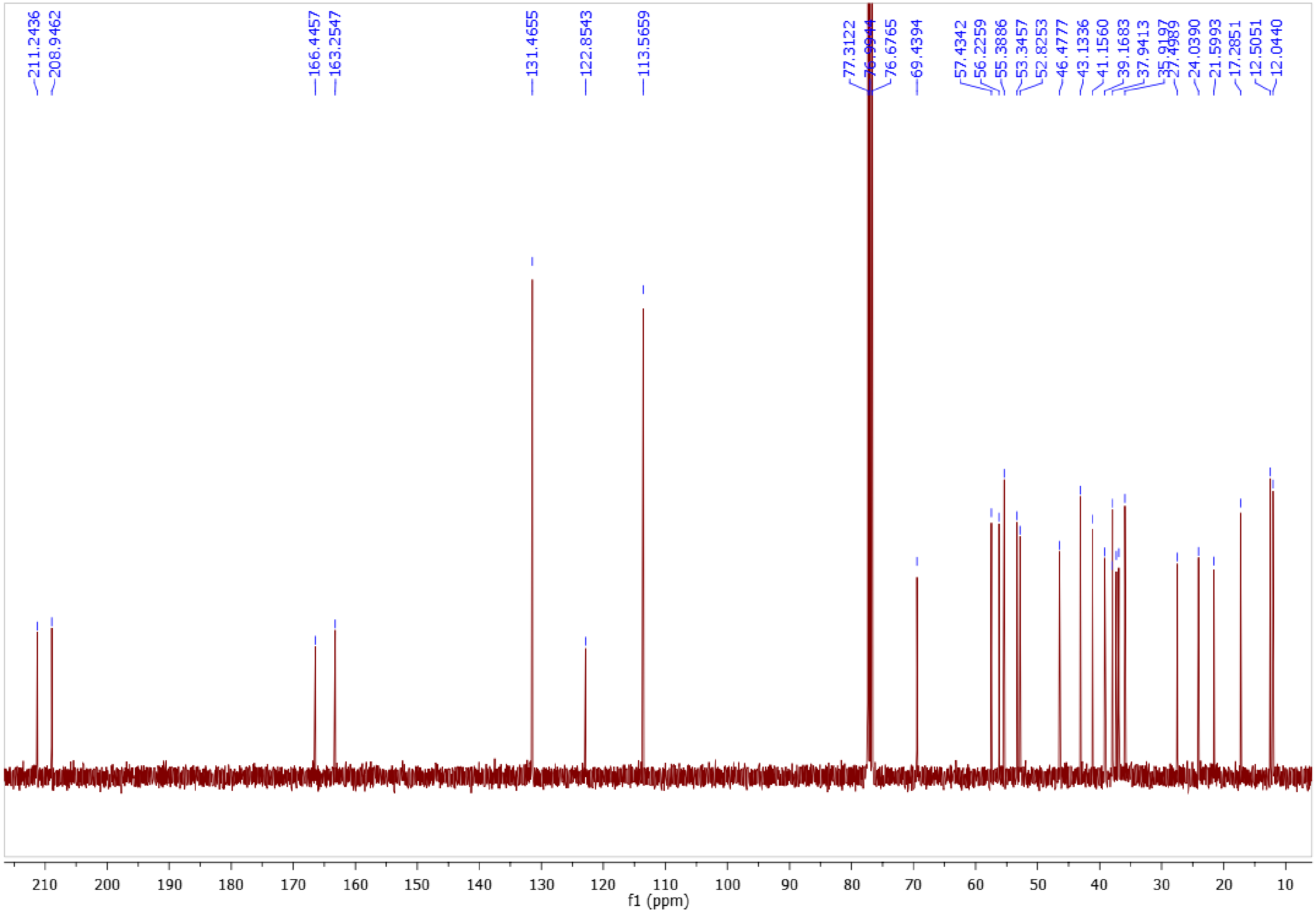

S42. ^{13}C NMR spectrum of *3,6-dioxo-23,24-dinor-5α-cholan-(4-methoxy)-benzoate-22-yl* (***21***).

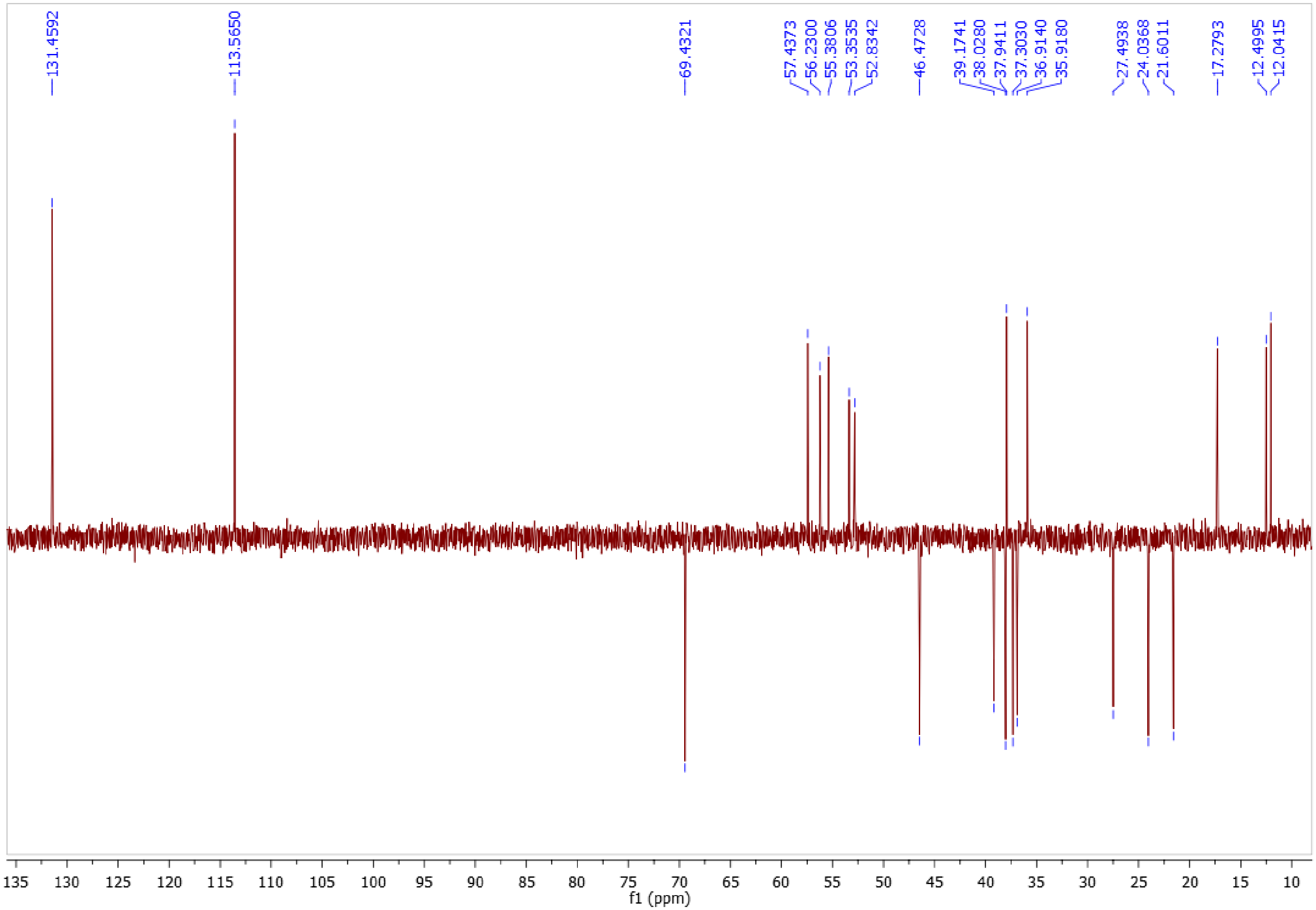

S43. ^{13}C DEPT-135 NMR spectrum of *3,6-dioxo-23,24-dinor-5α-cholan-(4-methoxy)-benzoate-22-yl* (**21**).

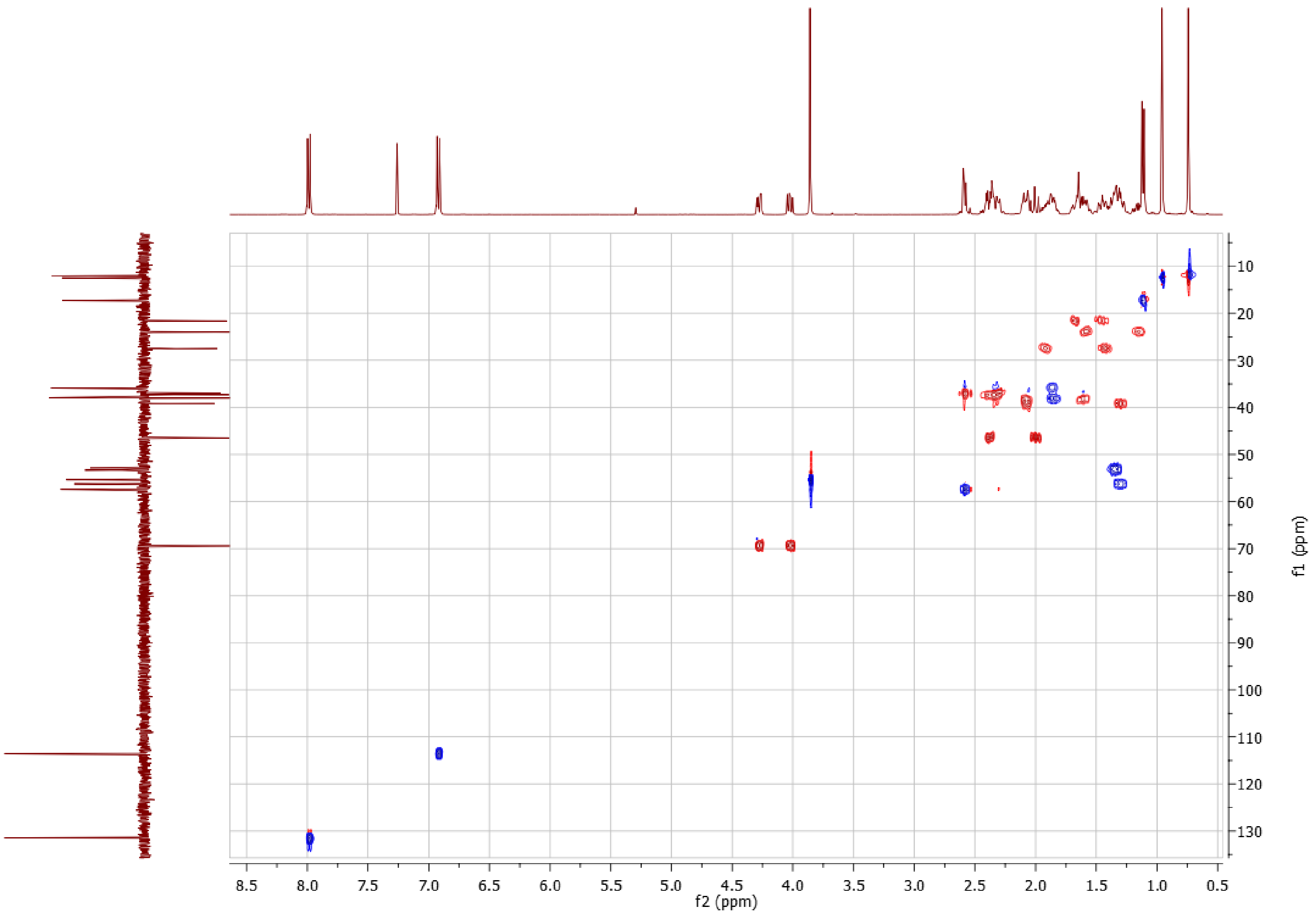

S44. 2D HSQC NMR spectrum of *3,6-dioxo-23,24-dinor-5α-cholan-(4-methoxy)-benzoate-22-yl* (***21***).

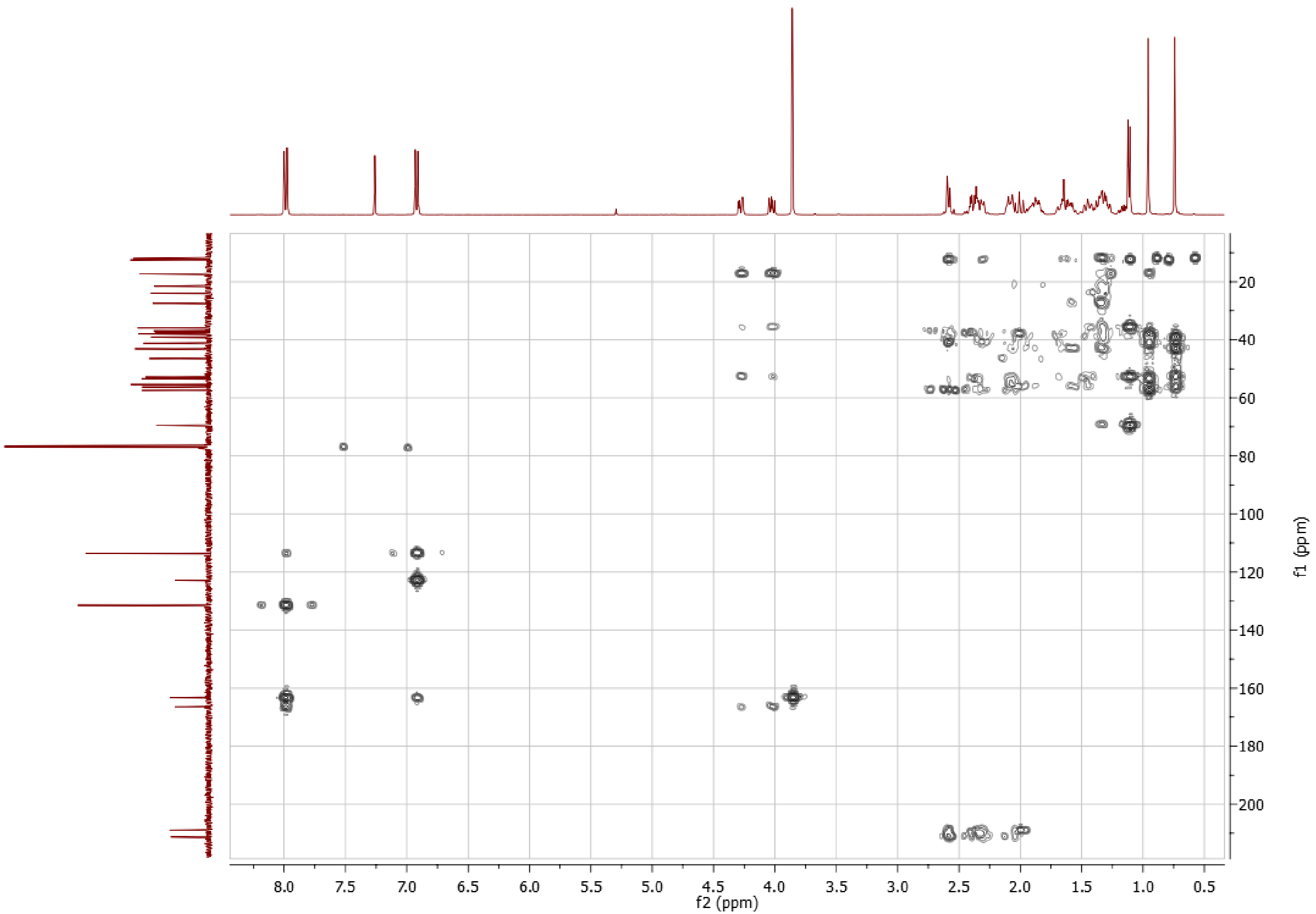

S45. 2D HMBC NMR spectrum of *3,6-dioxo-23,24-dinor-5α-cholan-(4-methoxy)-benzoate-22-yl* (**21**).

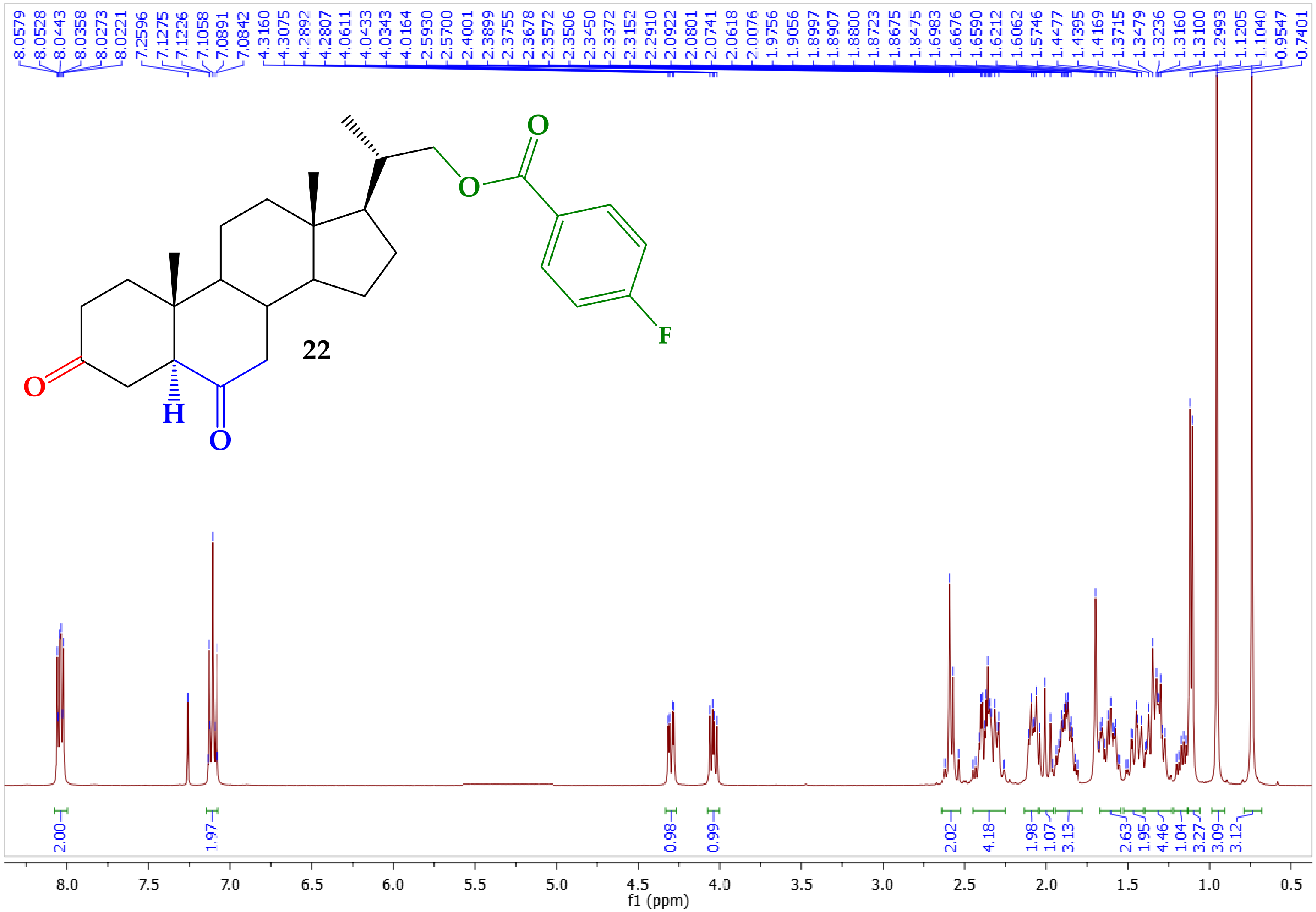

S46. ^{1}H NMR spectrum of *3,6-dioxo-23,24-dinor-5α-cholan-(4-fluoro)-benzoate-22-yl* (**22**).

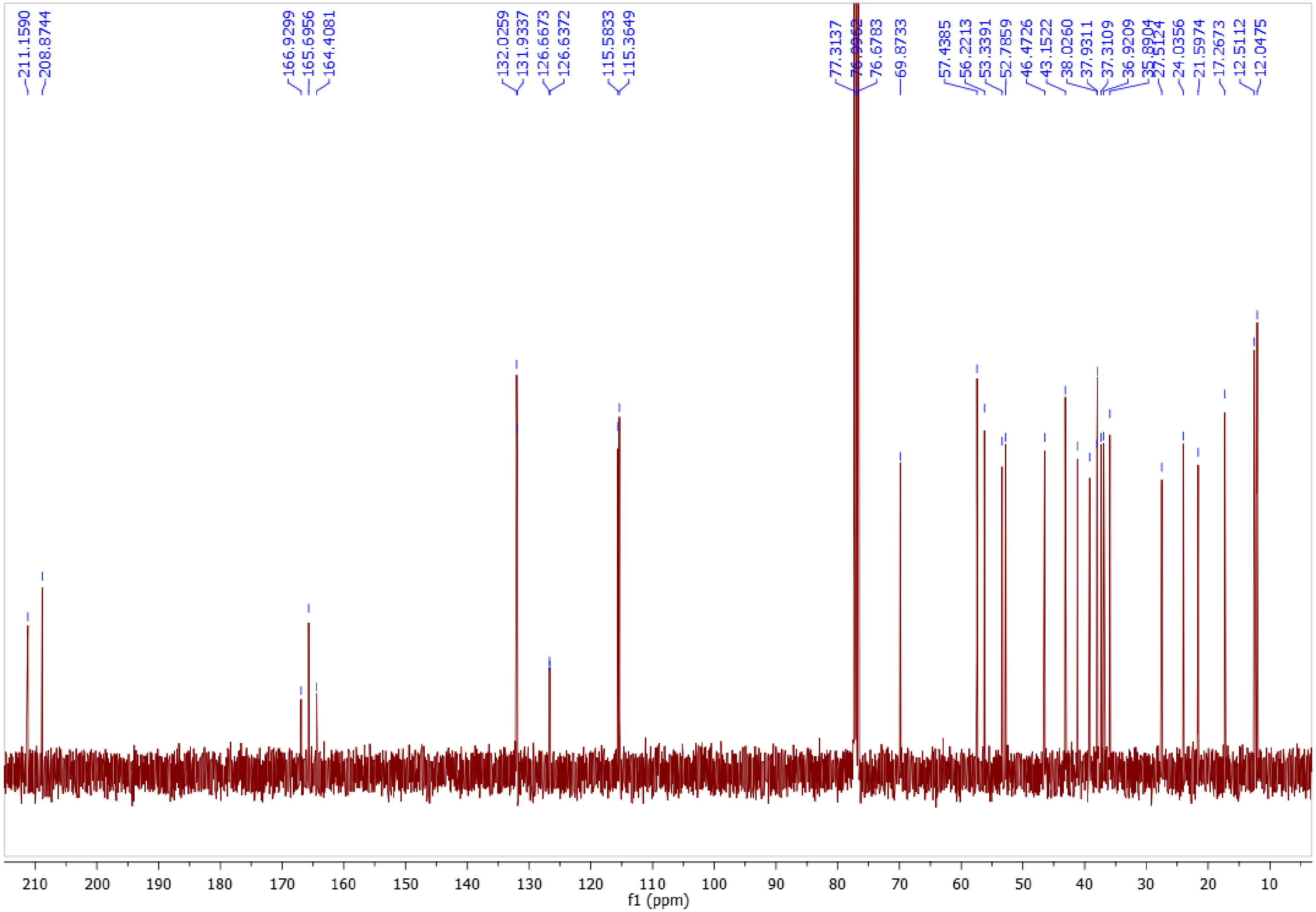

S47. ^{13}C NMR spectrum of *3,6-dioxo-23,24-dinor-5α-cholan-(4-fluoro)-benzoate-22-yl* (**22**).

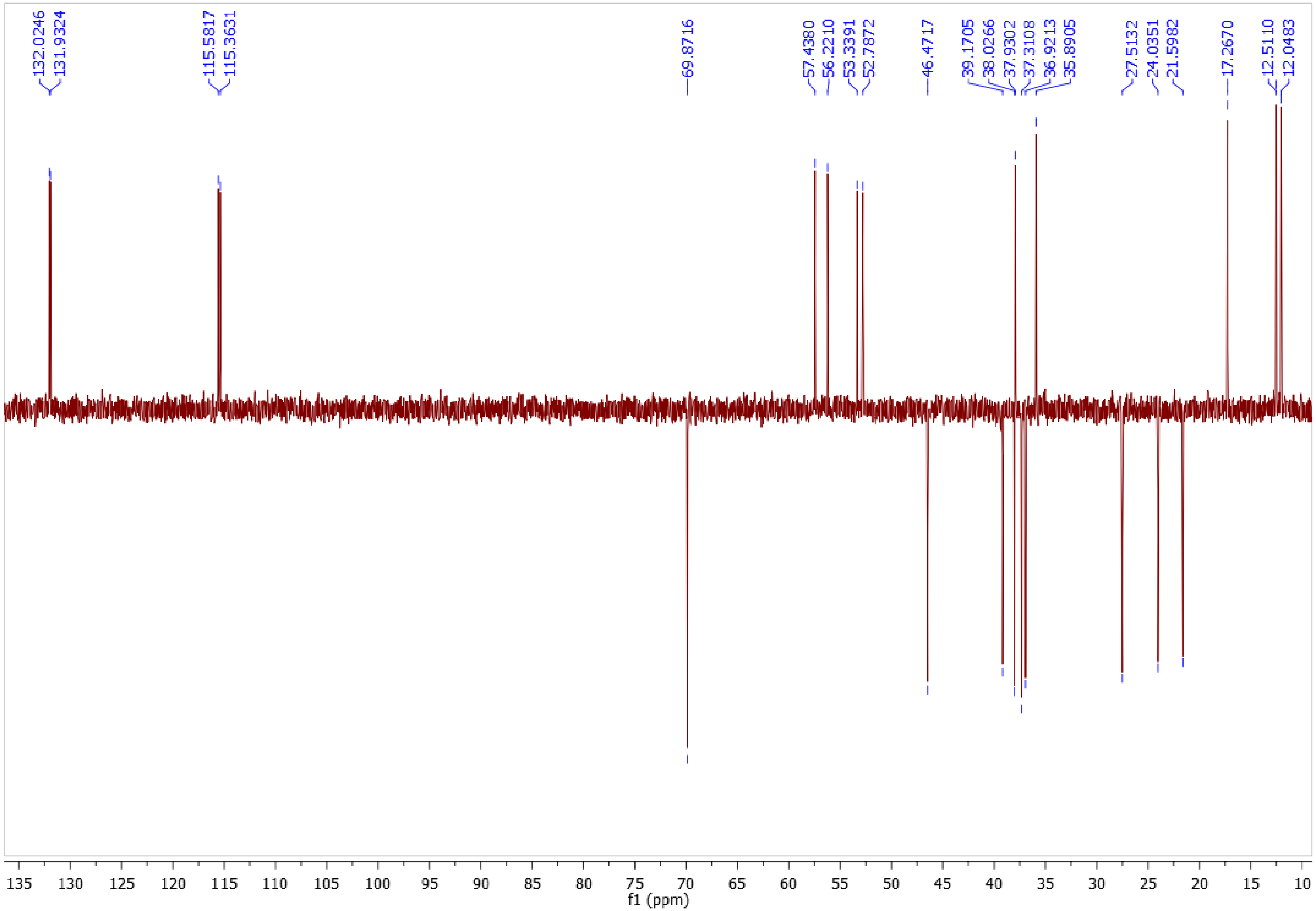

S48. ^{13}C DEPT-135 NMR spectrum of *3,6-dioxo-23,24-dinor-5α-cholan-(4-fluoro)-benzoate-22-yl* (**22**).

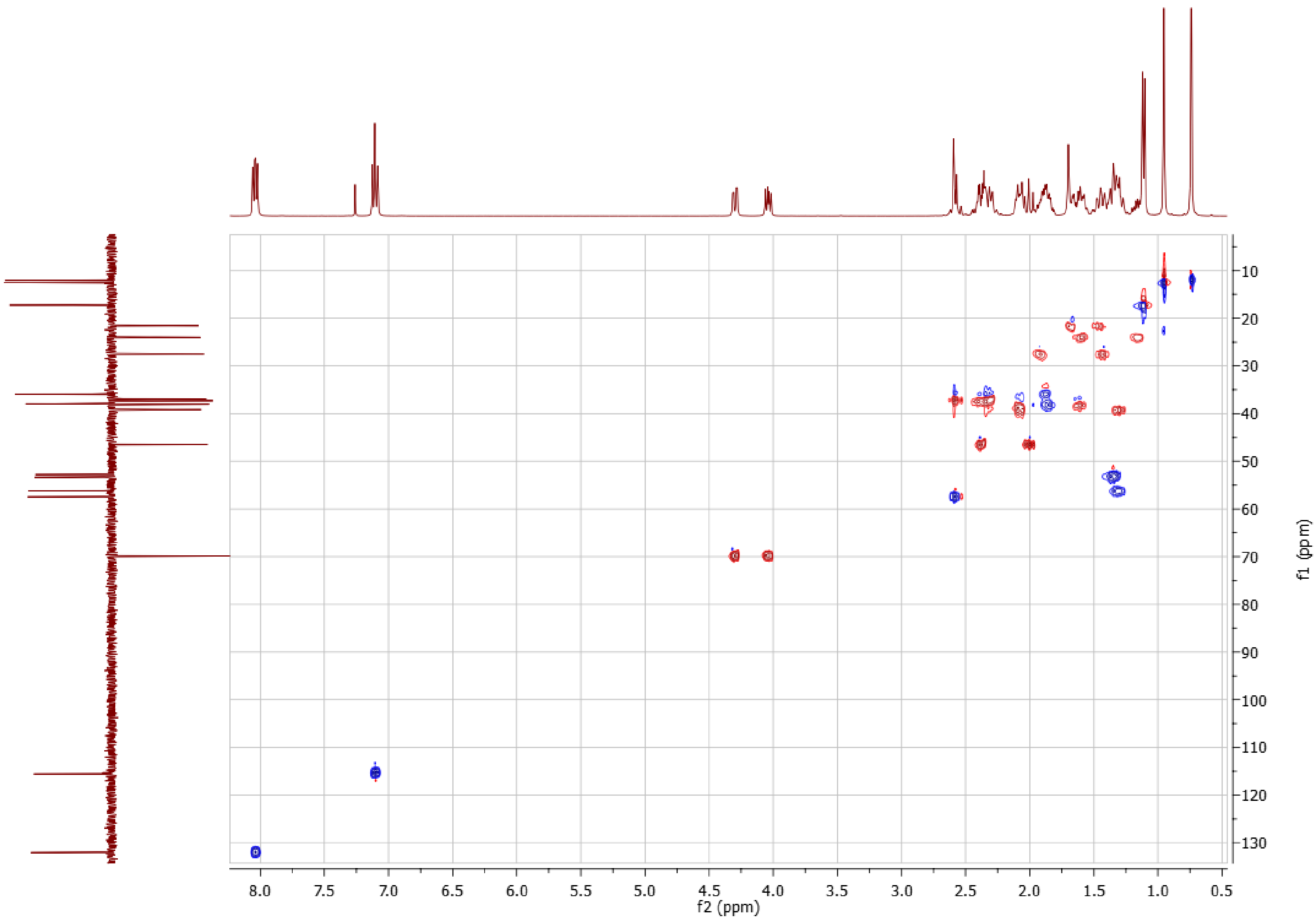

S49. 2D HSQC NMR spectrum of *3,6-dioxo-23,24-dinor-5α-cholan-(4-fluoro)-benzoate-22-yl* (**22**).

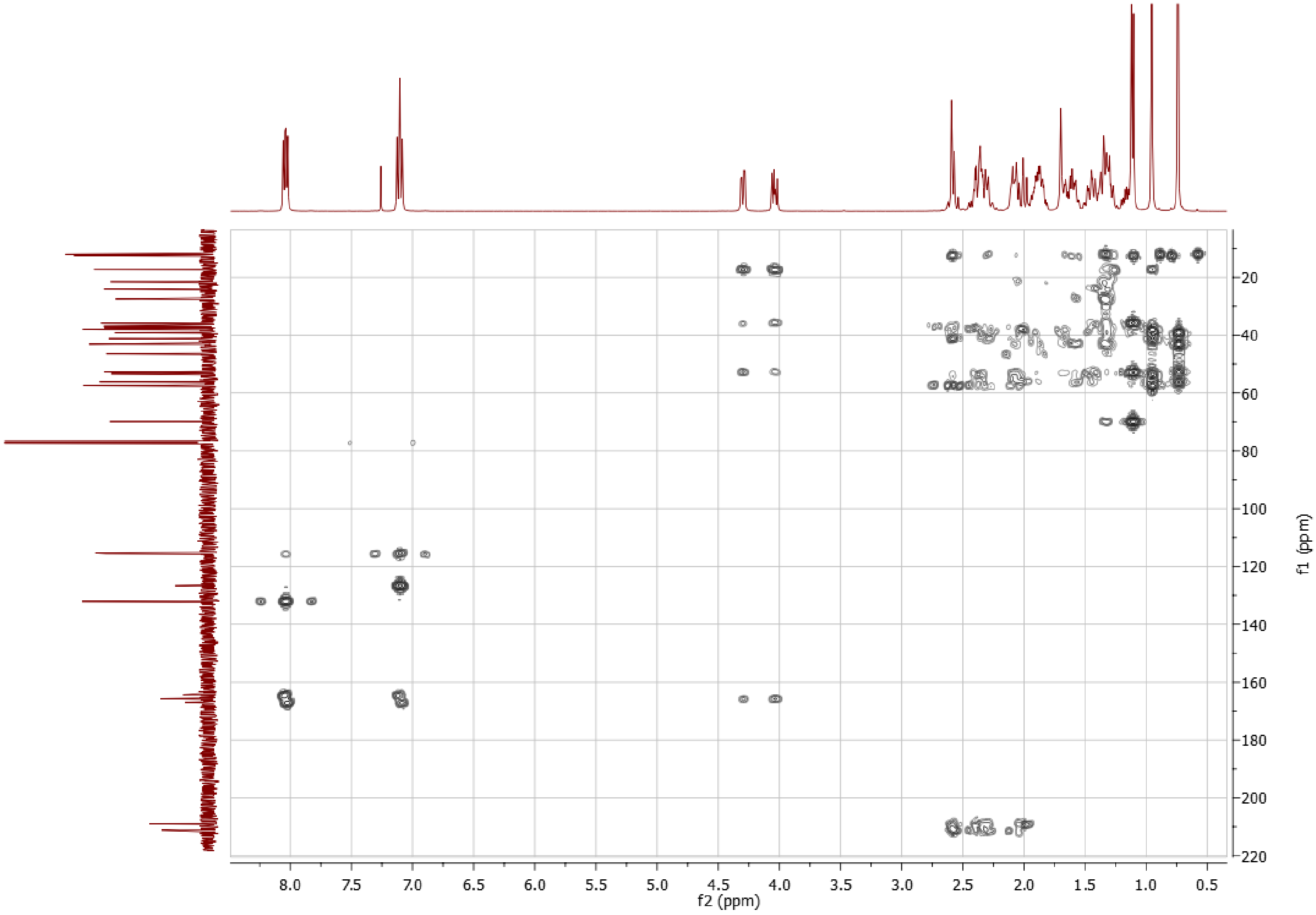

S50. 2D HMBC NMR spectrum of *3,6-dioxo-23,24-dinor-5α-cholan-(4-fluoro)-benzoate-22-yl* (**22**).

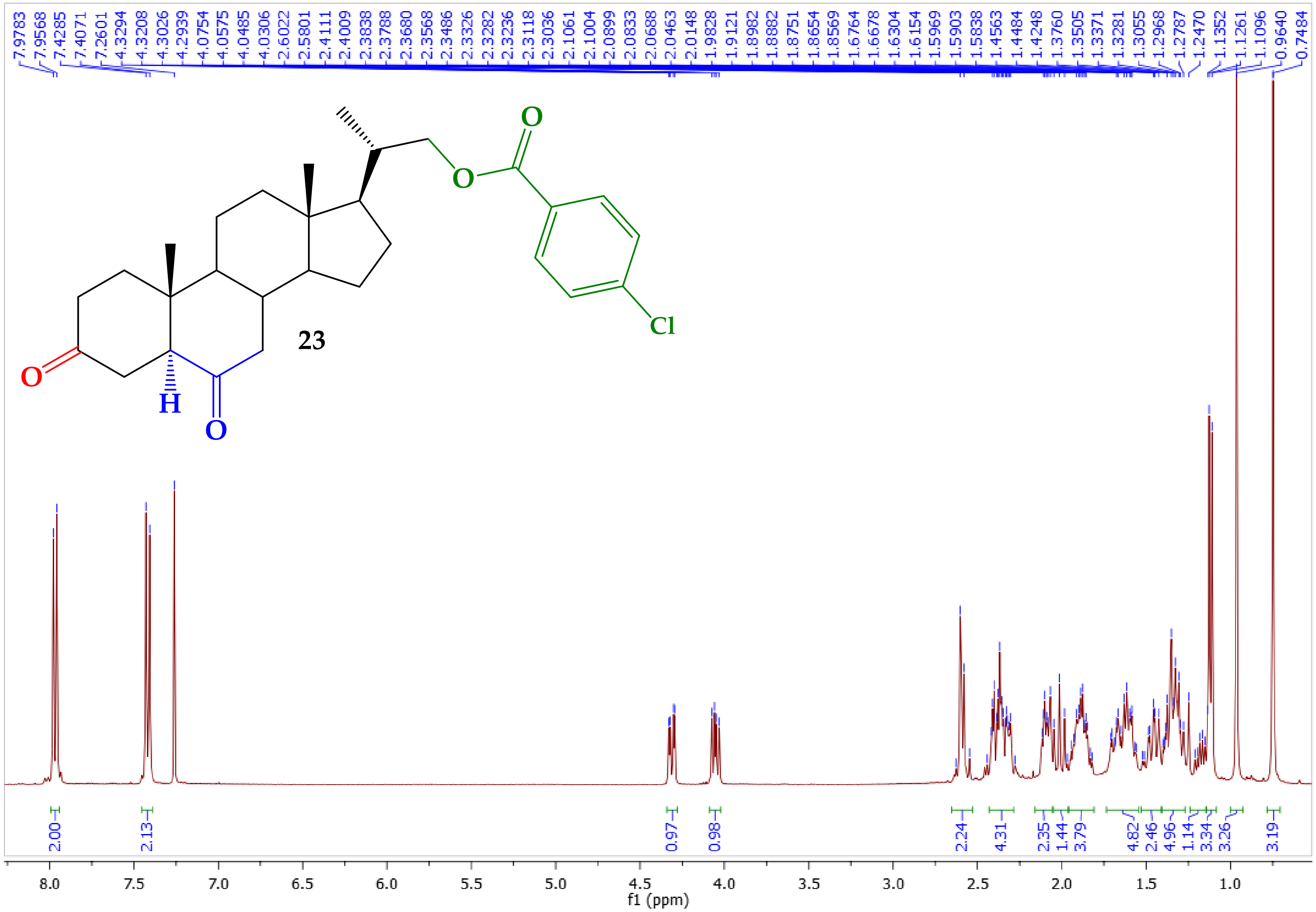

S51. ^{1}H NMR spectrum of *3,6-dioxo-23,24-dinor-5α-cholan-(4-chloro)-benzoate-22-yl* (**23**).

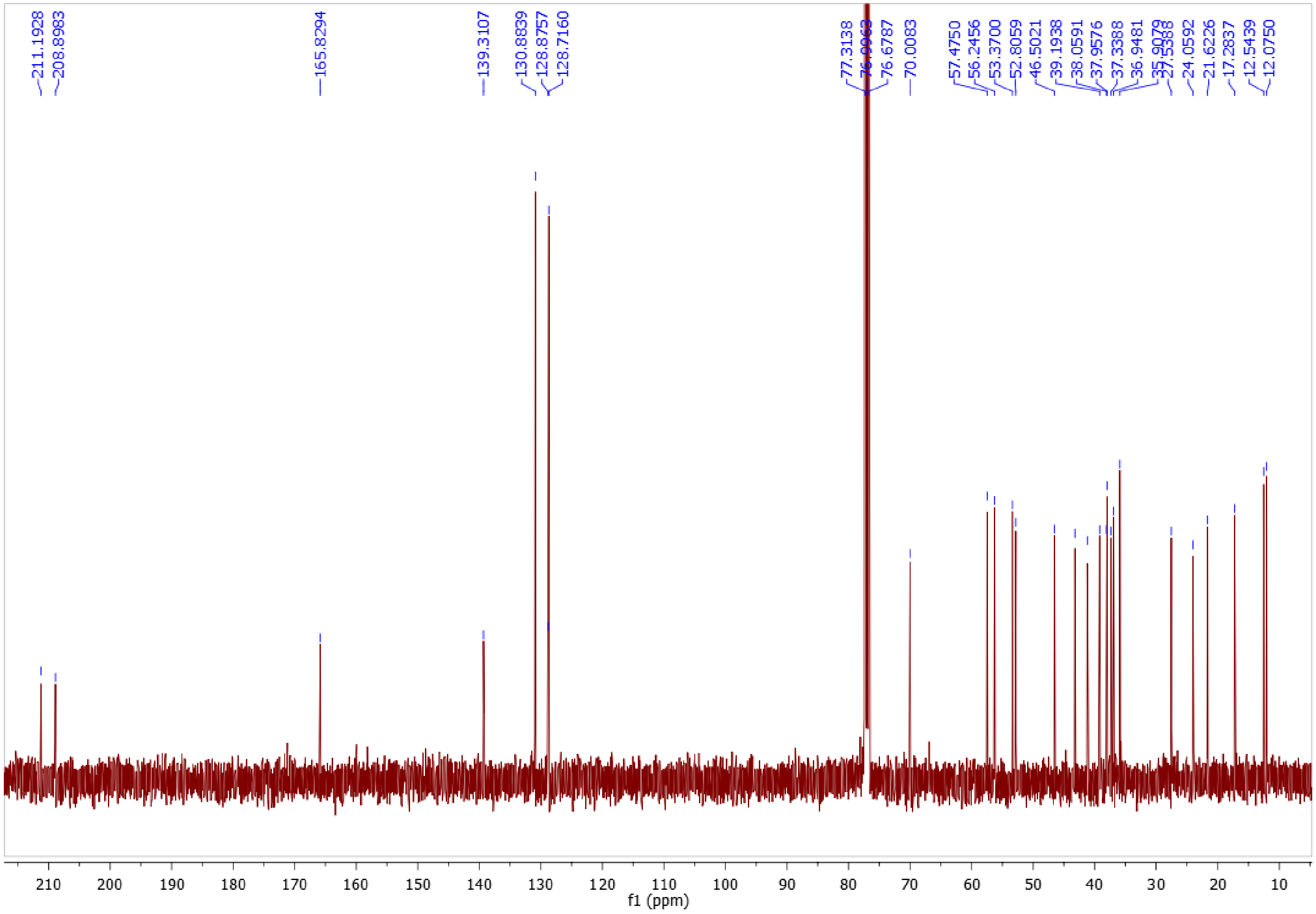

S52. ^{13}C NMR spectrum of *3,6-dioxo-23,24-dinor-5α-cholan-(4-chloro)-benzoate-22-yl* (**23**).

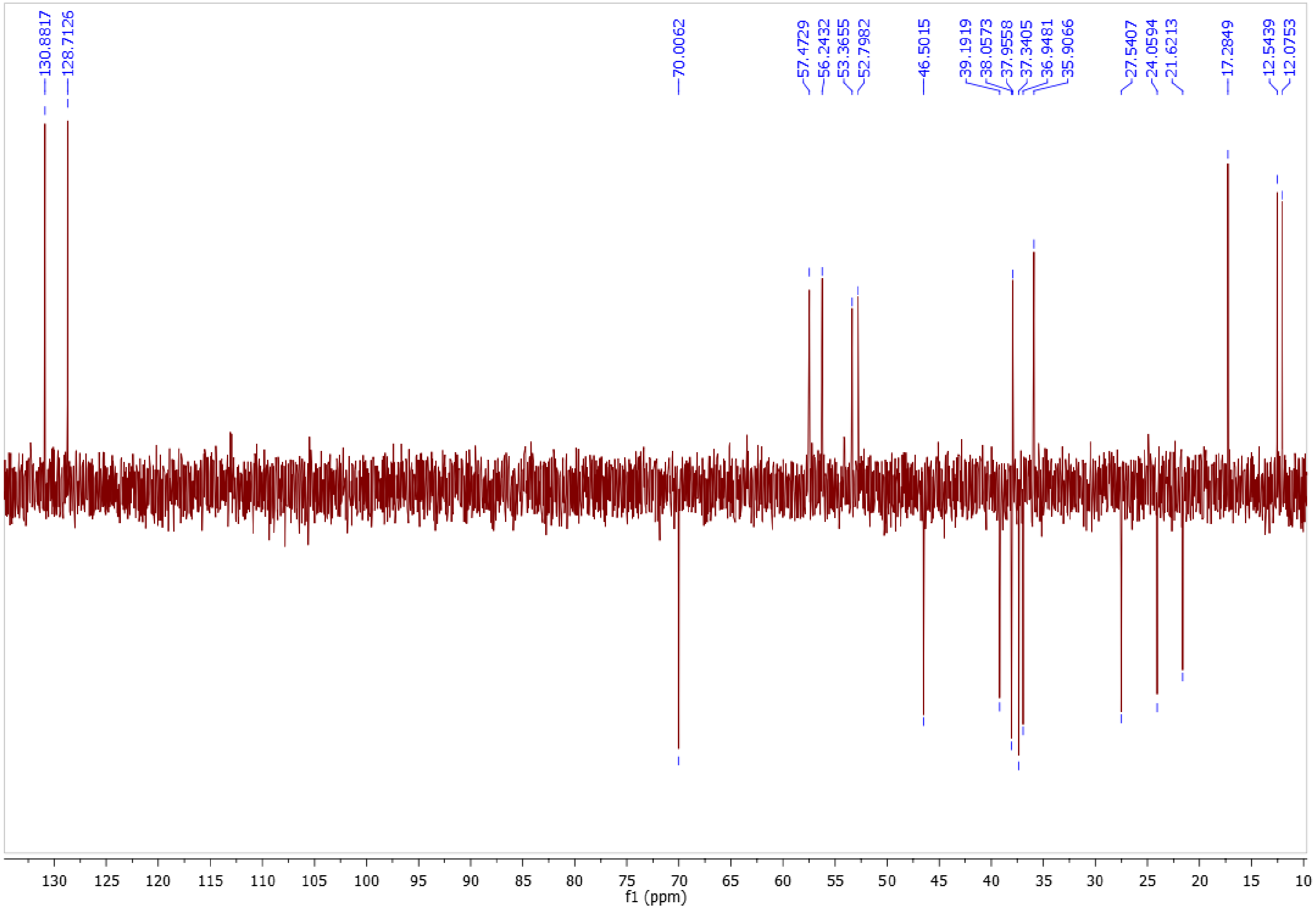

S53. ^{13}C DEPT-135 NMR spectrum of *3,6-dioxo-23,24-dinor-5α-cholan-(4-chloro)-benzoate-22-yl* (**23**).

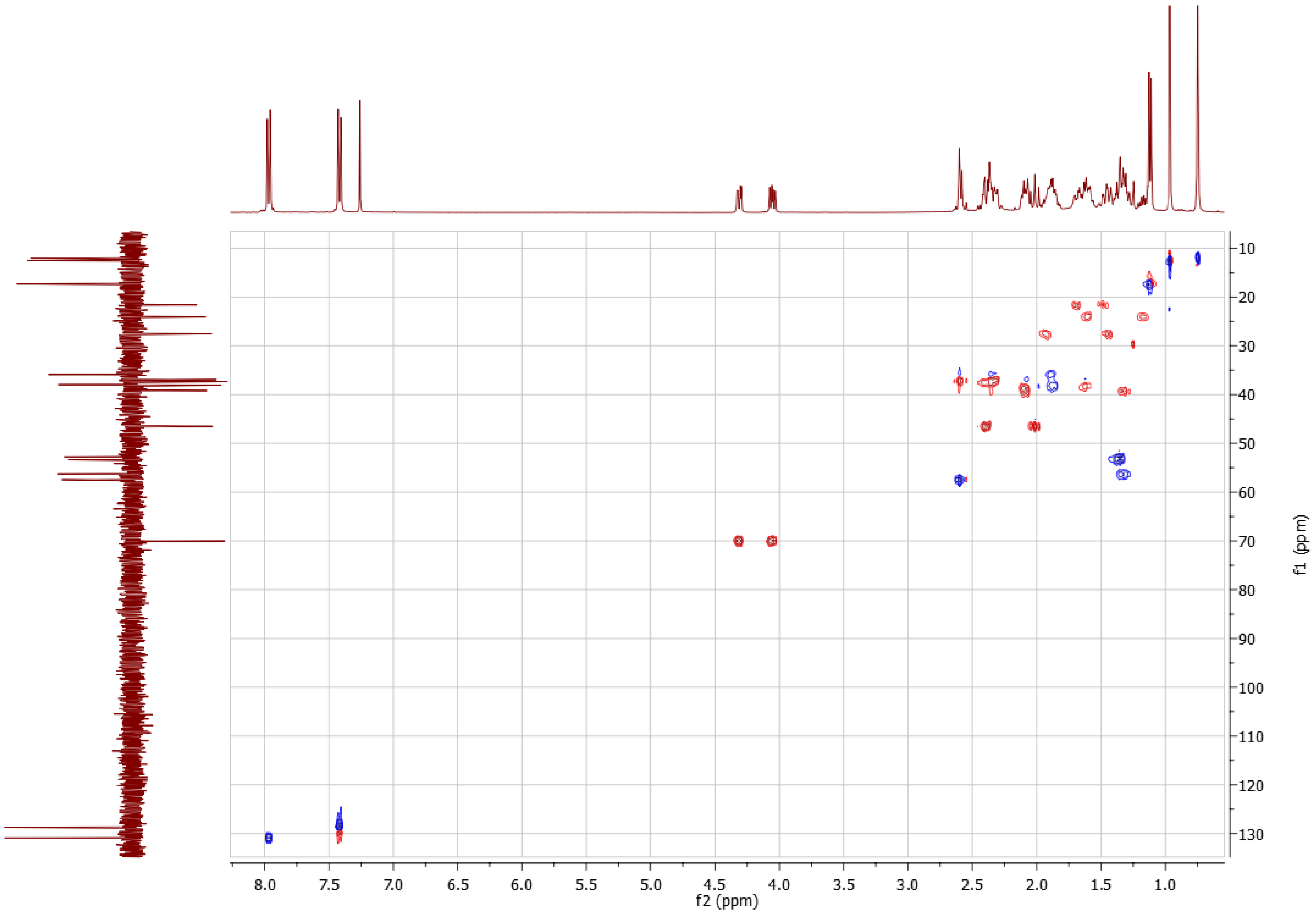

S54. 2D HSQC NMR spectrum of *3,6-dioxo-23,24-dinor-5α-cholan-(4-chloro)-benzoate-22-yl* (**23**).

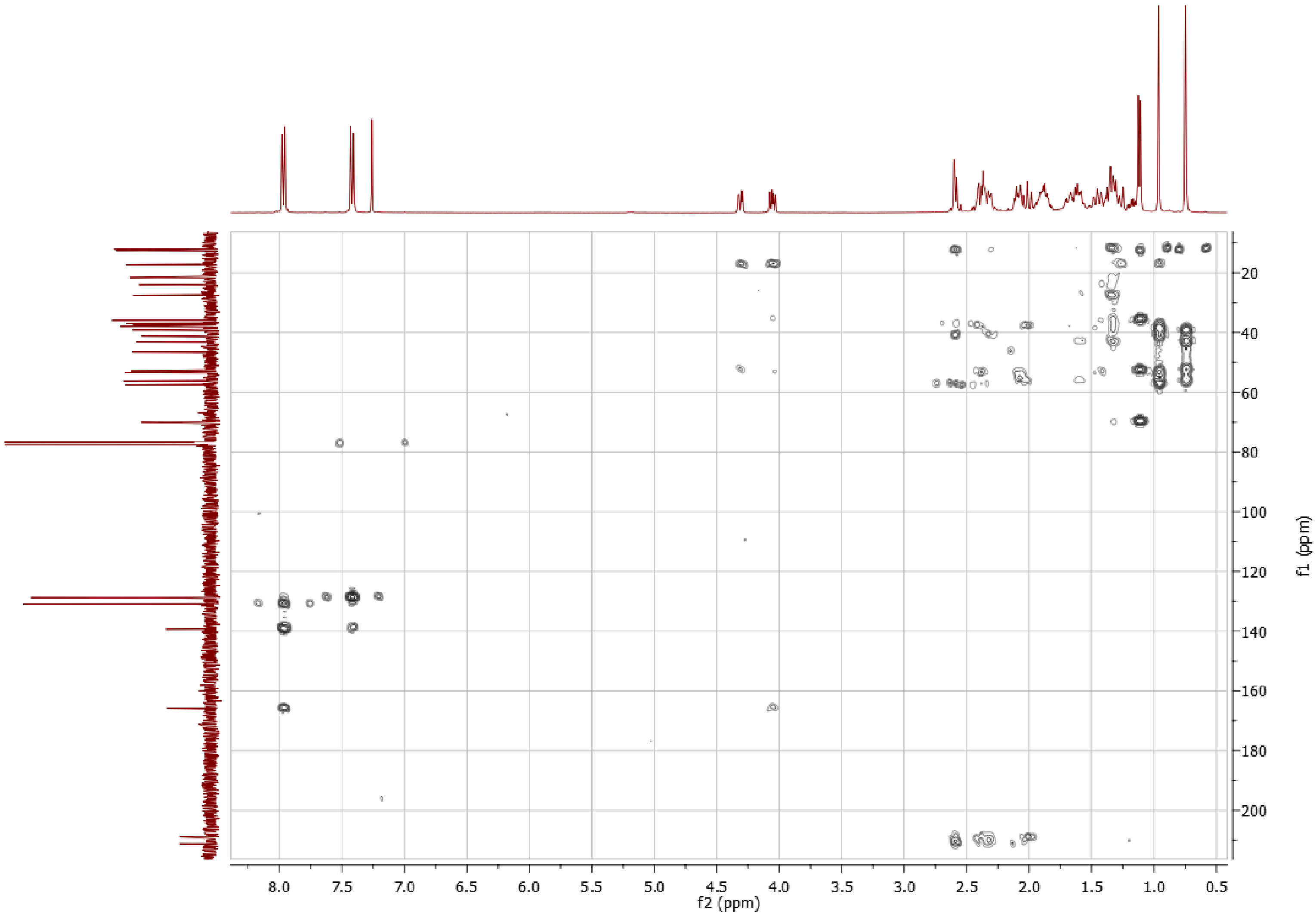

S55. 2D HMBC NMR spectrum of *3,6-dioxo-23,24-dinor-5α-cholan-(4-chloro)-benzoate-22-yl* (**23**).

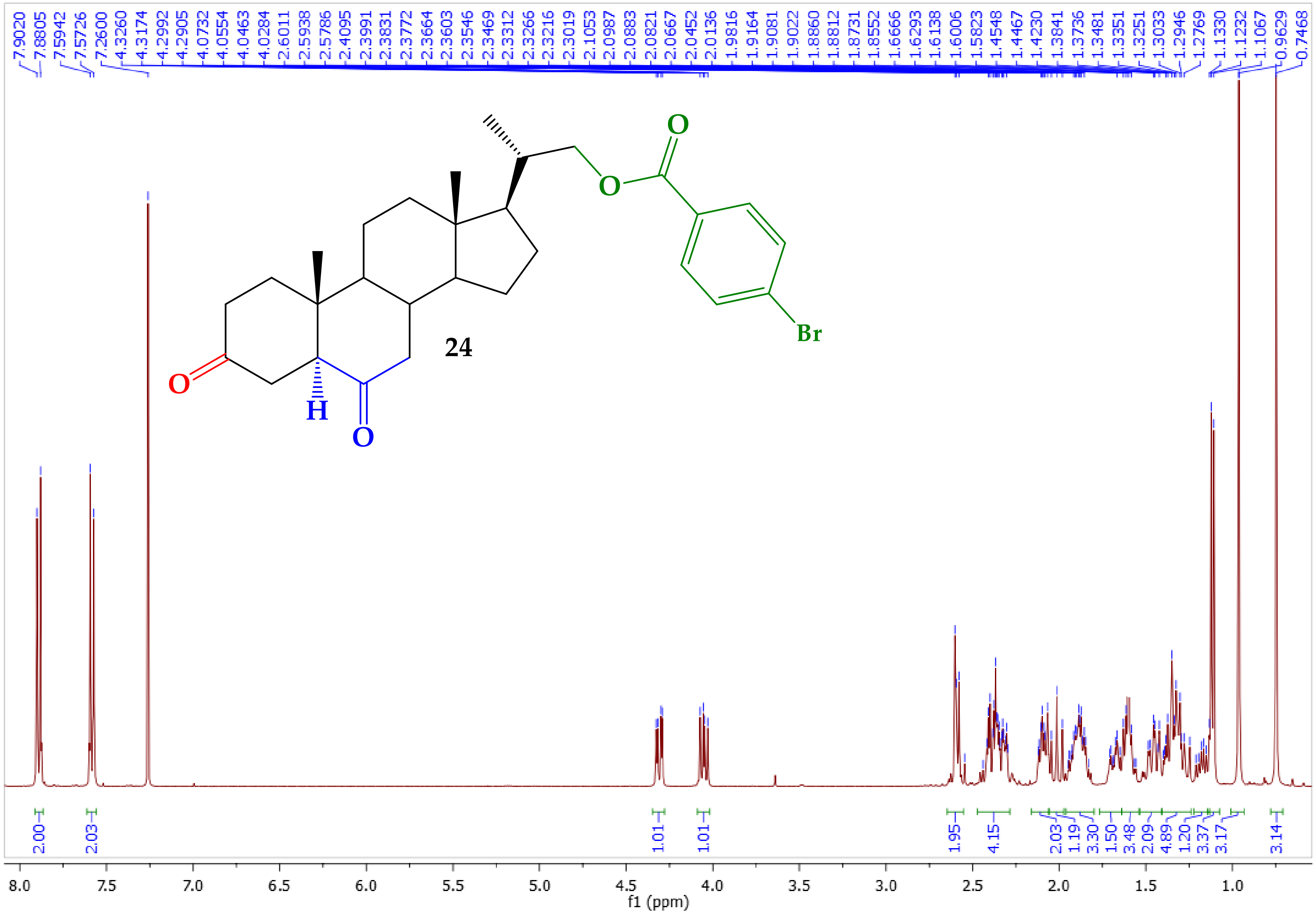

S56. ^{1}H NMR spectrum of *3,6-dioxo-23,24-dinor-5α-cholan-(4-bromo)-benzoate-22-yl* (**24**).

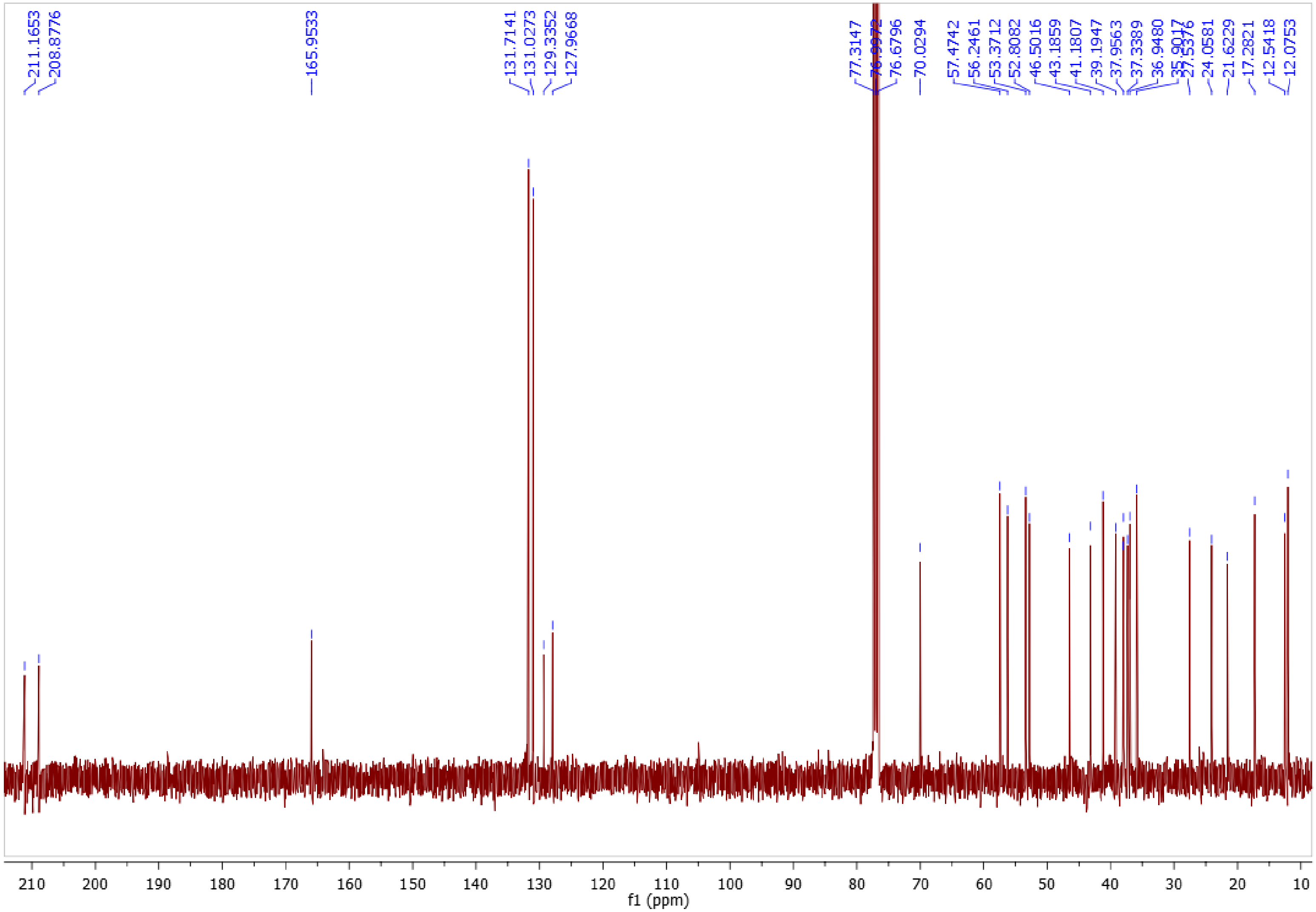

S57. ^{13}C NMR spectrum of *3,6-dioxo-23,24-dinor-5α-cholan-(4-bromo)-benzoate-22-yl* (***24***).

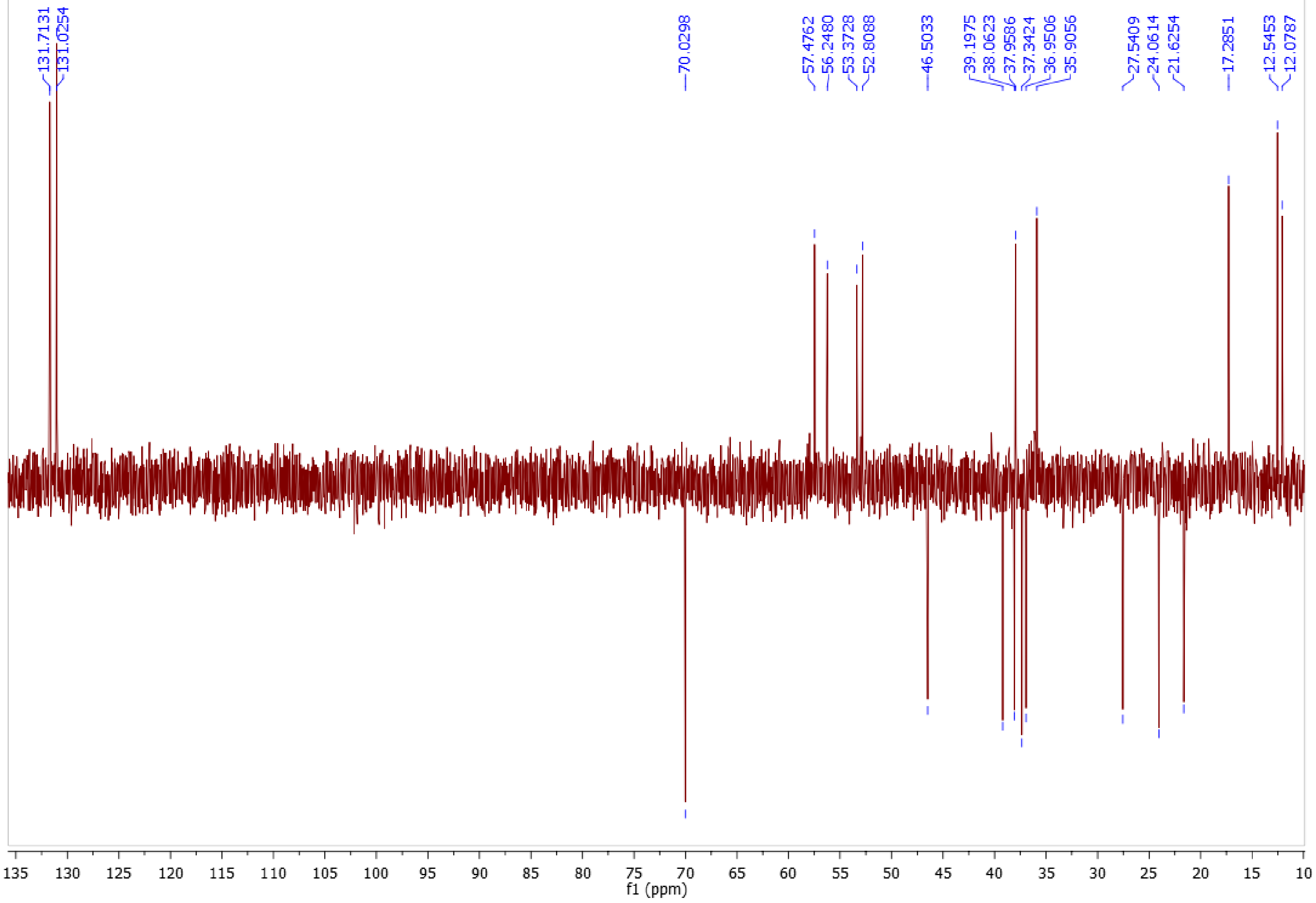

S58. ^{13}C DEPT-135 NMR spectrum of *3,6-dioxo-23,24-dinor-5α-cholan-(4-bromo)-benzoate-22-yl* (**24**).

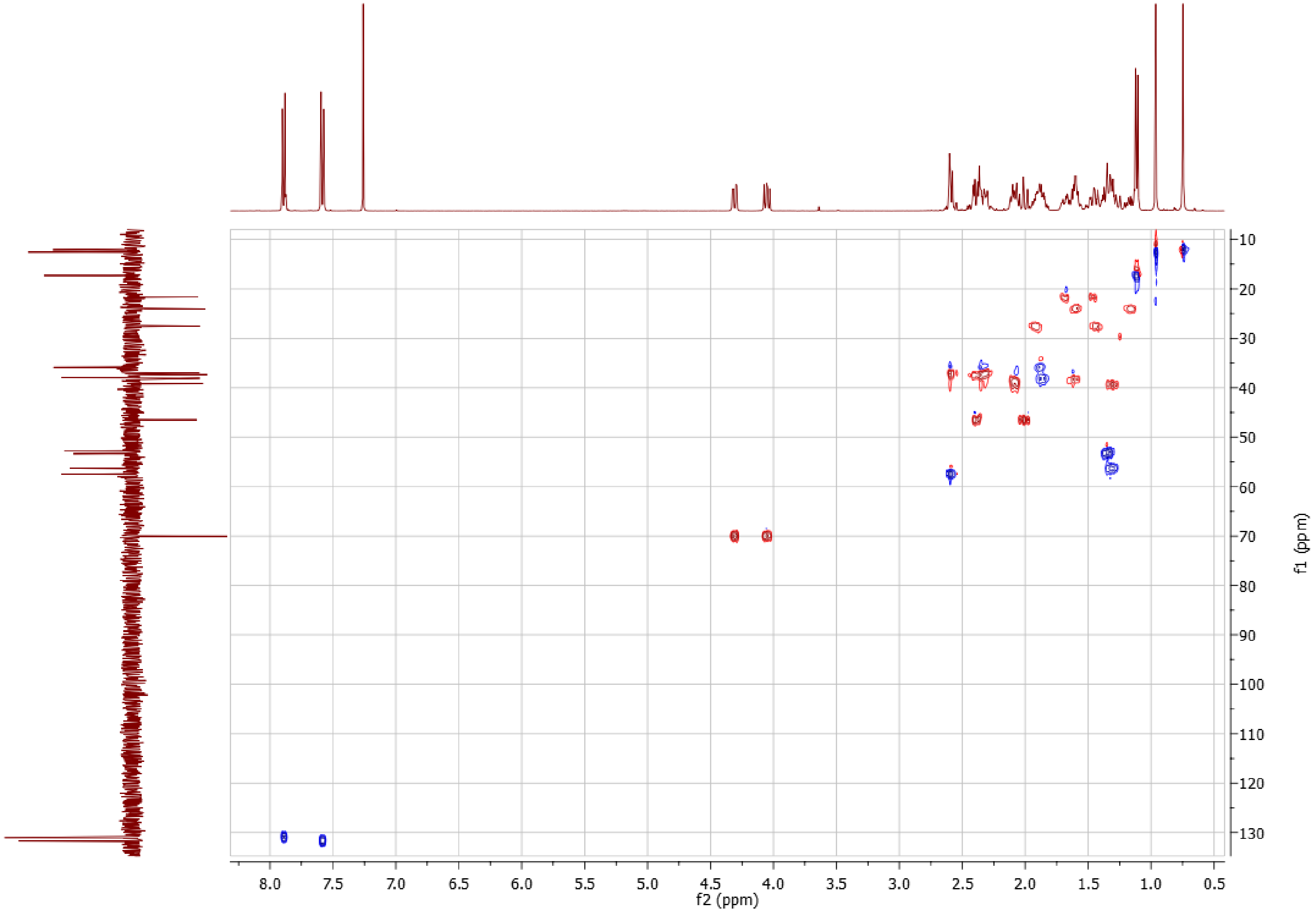

S59. 2D HSQC NMR spectrum of *3,6-dioxo-23,24-dinor-5α-cholan-(4-bromo)-benzoate-22-yl* (**24**).

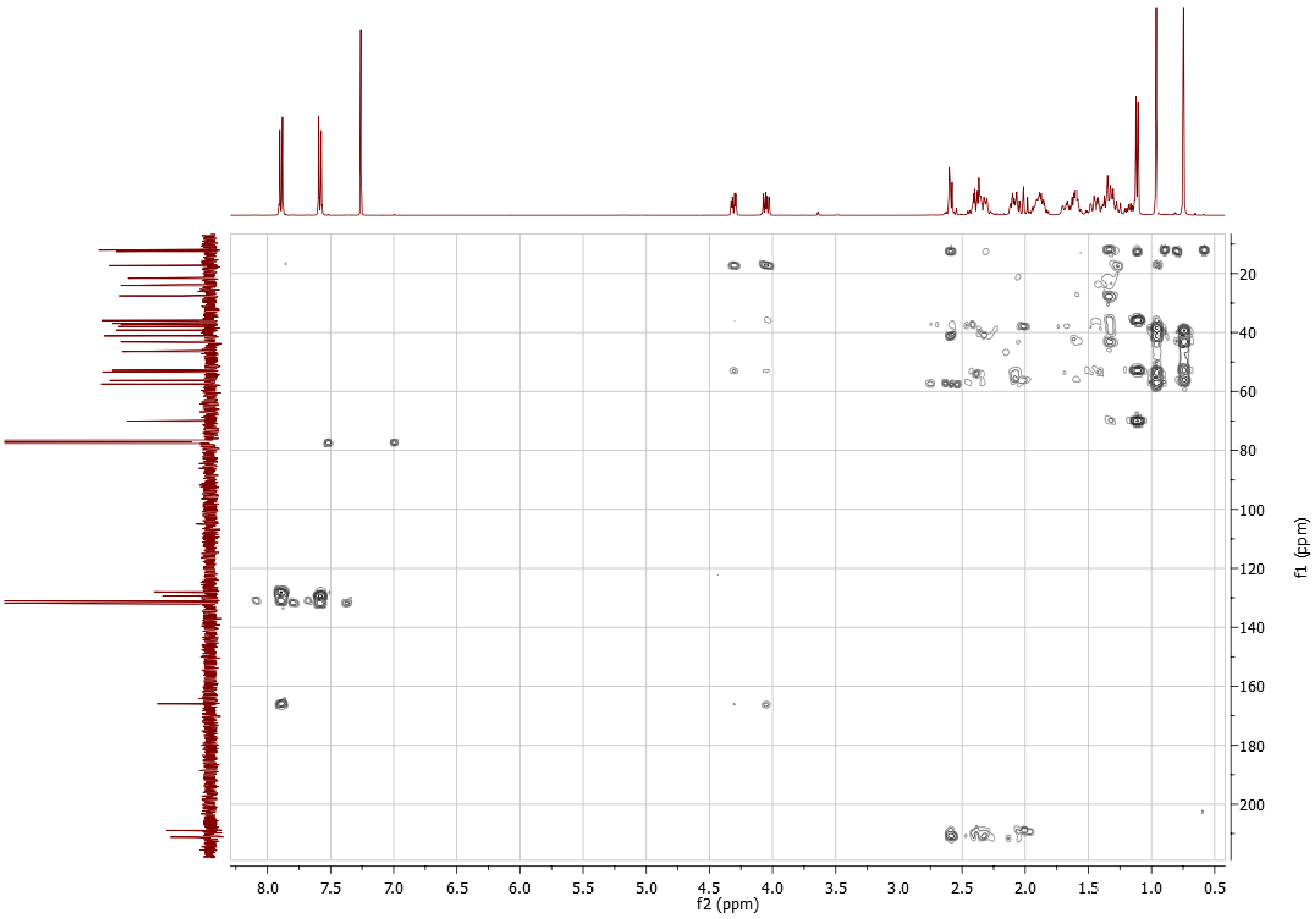

S60. 2D HMBC NMR spectrum of *3,6-dioxo-23,24-dinor-5α-cholan-(4-bromo)-benzoate-22-yl* (**24**).

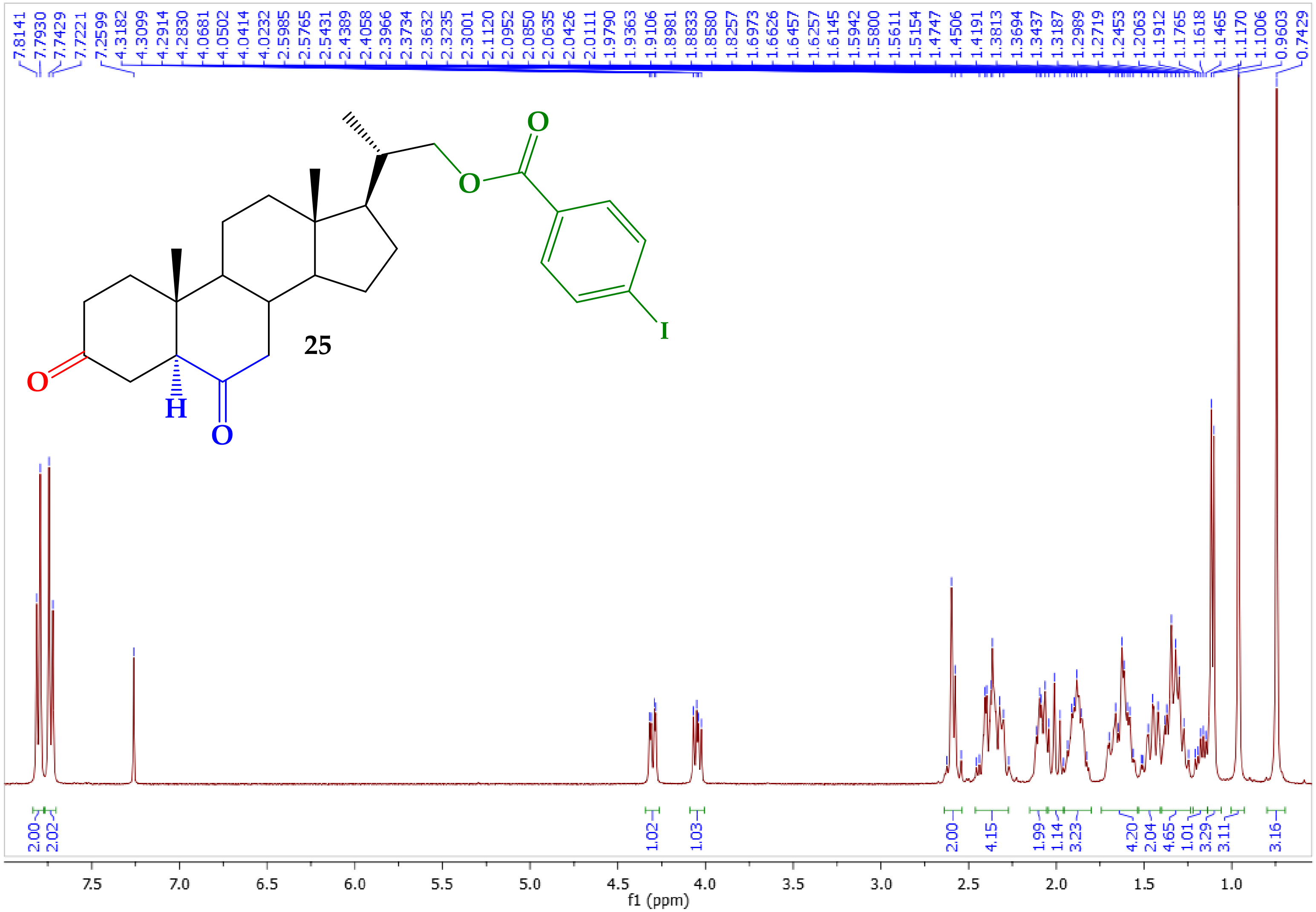

S61. ¹H NMR spectrum of *3,6-dioxo-23,24-dinor-5α-cholan-(4-iodo)-benzoate-22-yl* (**25**).

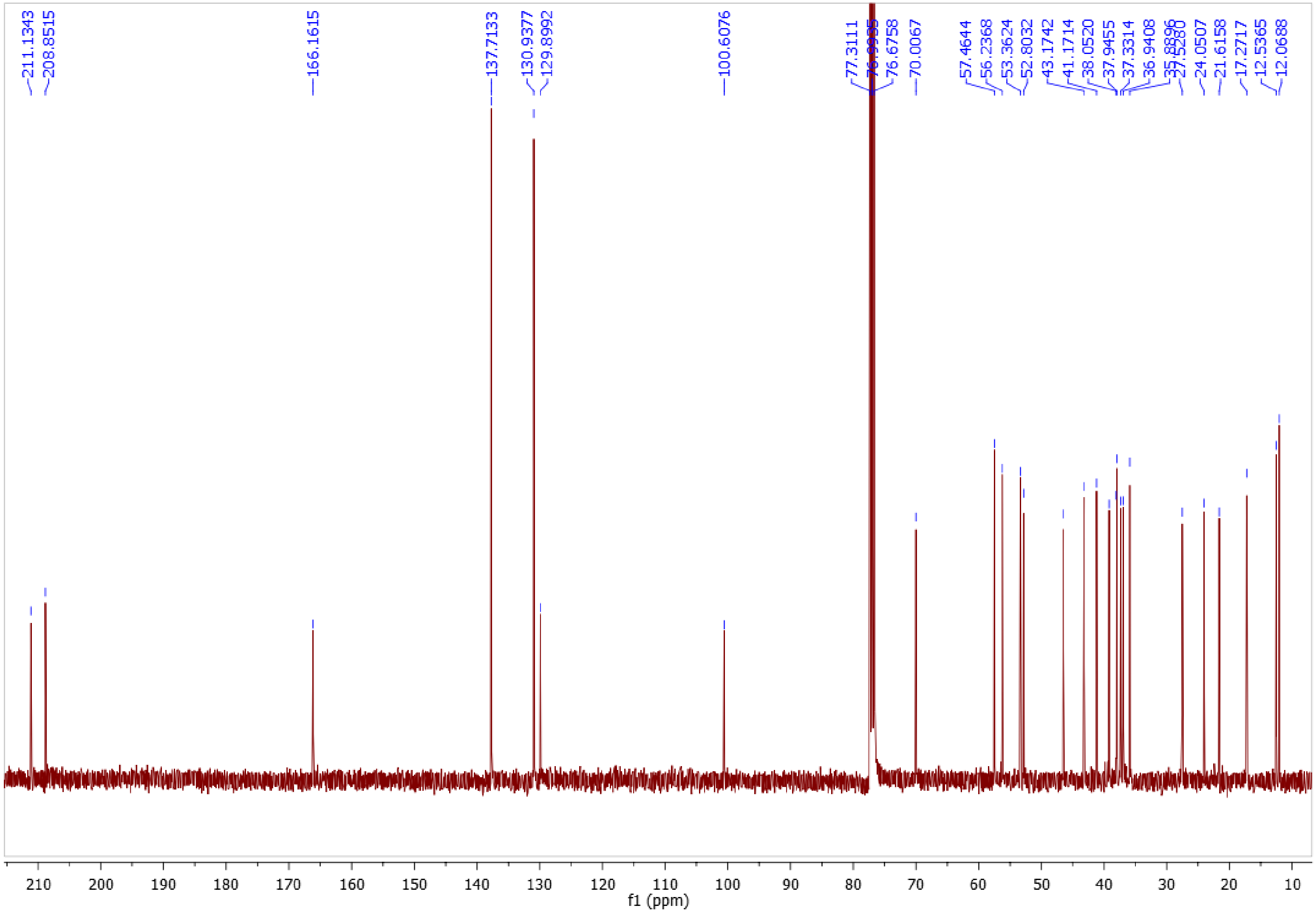

S62. ^{13}C NMR spectrum of *3,6-dioxo-23,24-dinor-5α-cholan-(4-iodo)-benzoate-22-yl* (**25**).

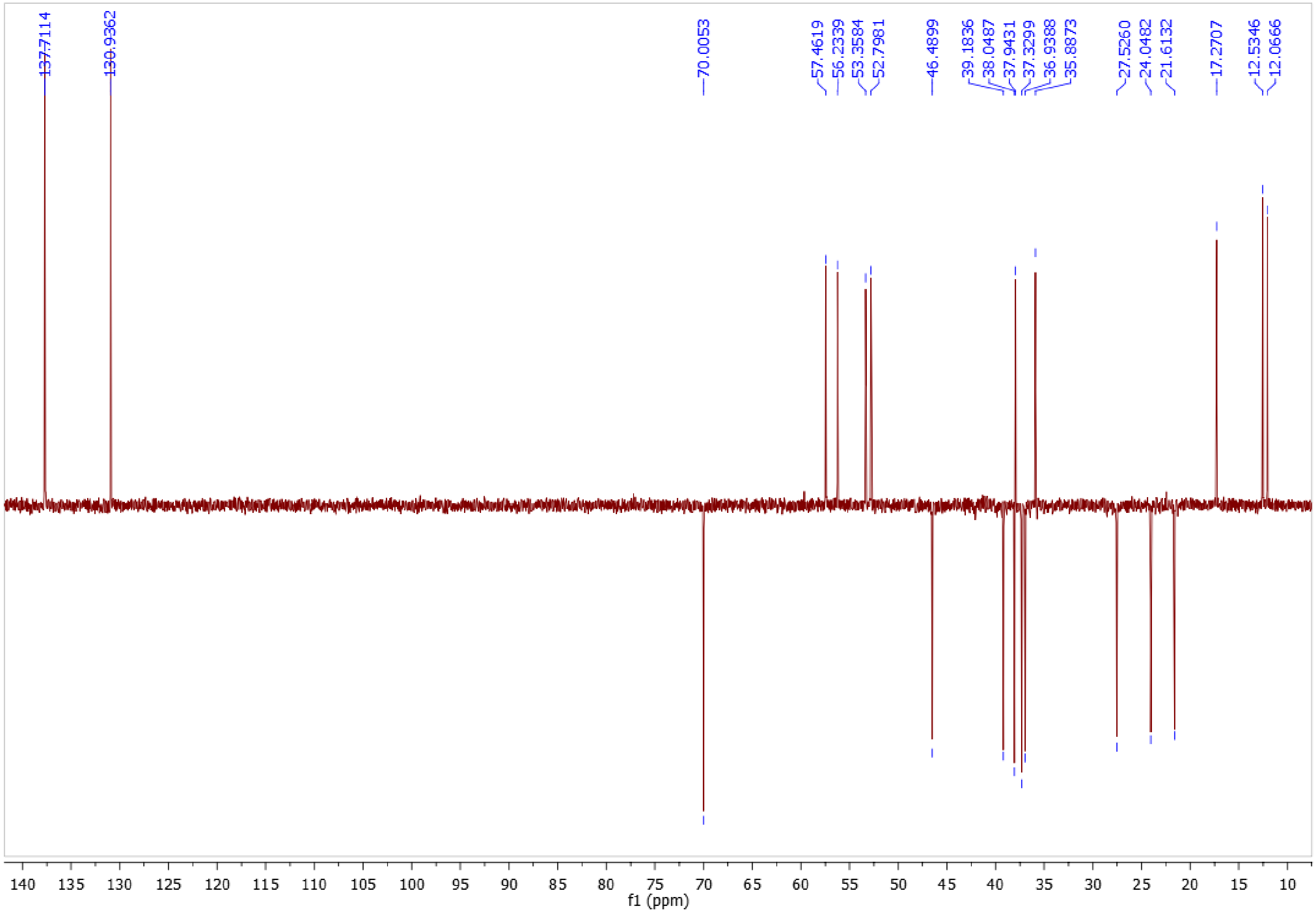

S63. ^{13}C DEPT-135 NMR spectrum of *3,6-dioxo-23,24-dinor-5α-cholan-(4-iodo)-benzoate-22-yl* (**25**).

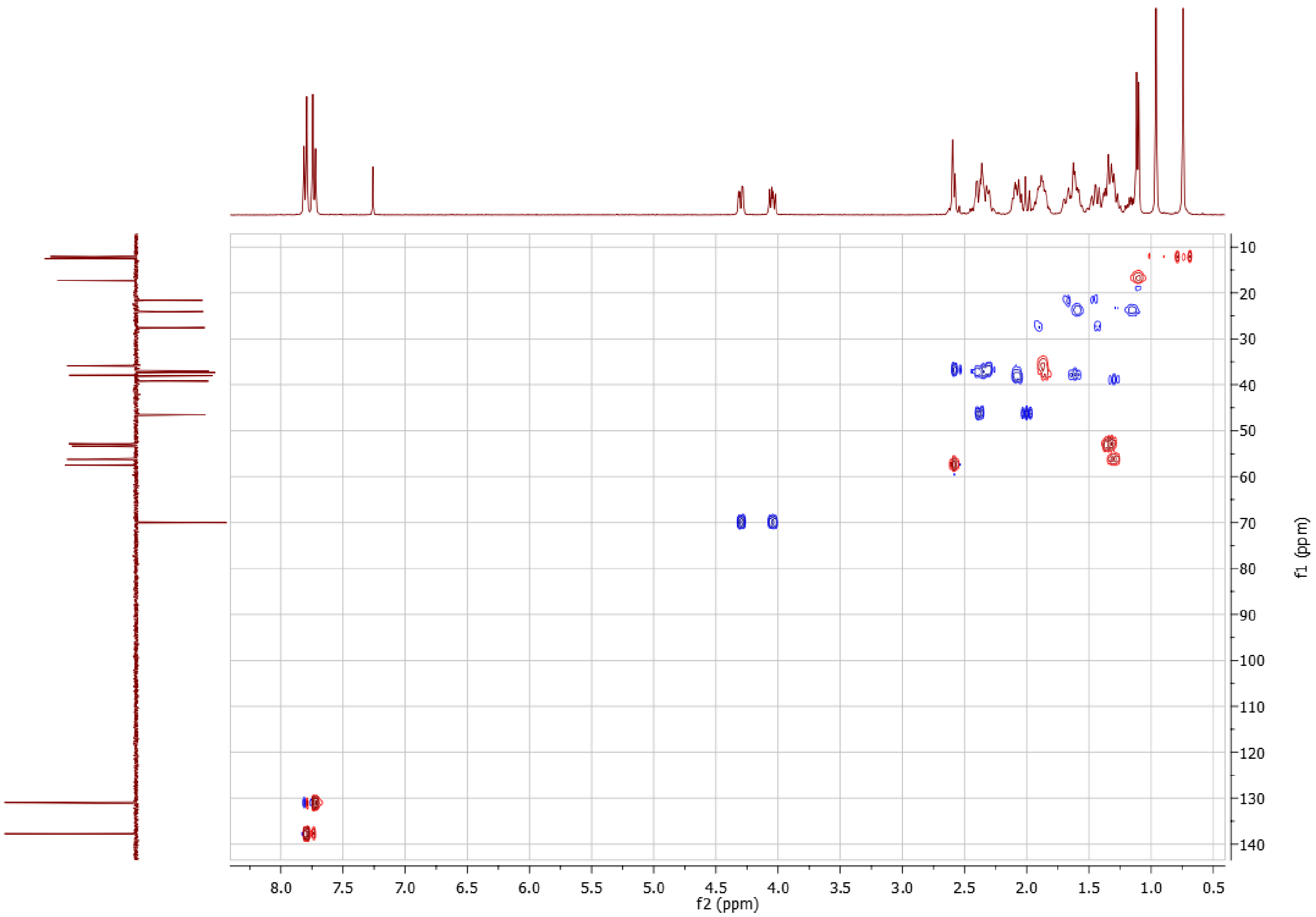

S64. 2D HSQC NMR spectrum of *3,6-dioxo-23,24-dinor-5α-cholan-(4-iodo)-benzoate-22-yl* (**25**).

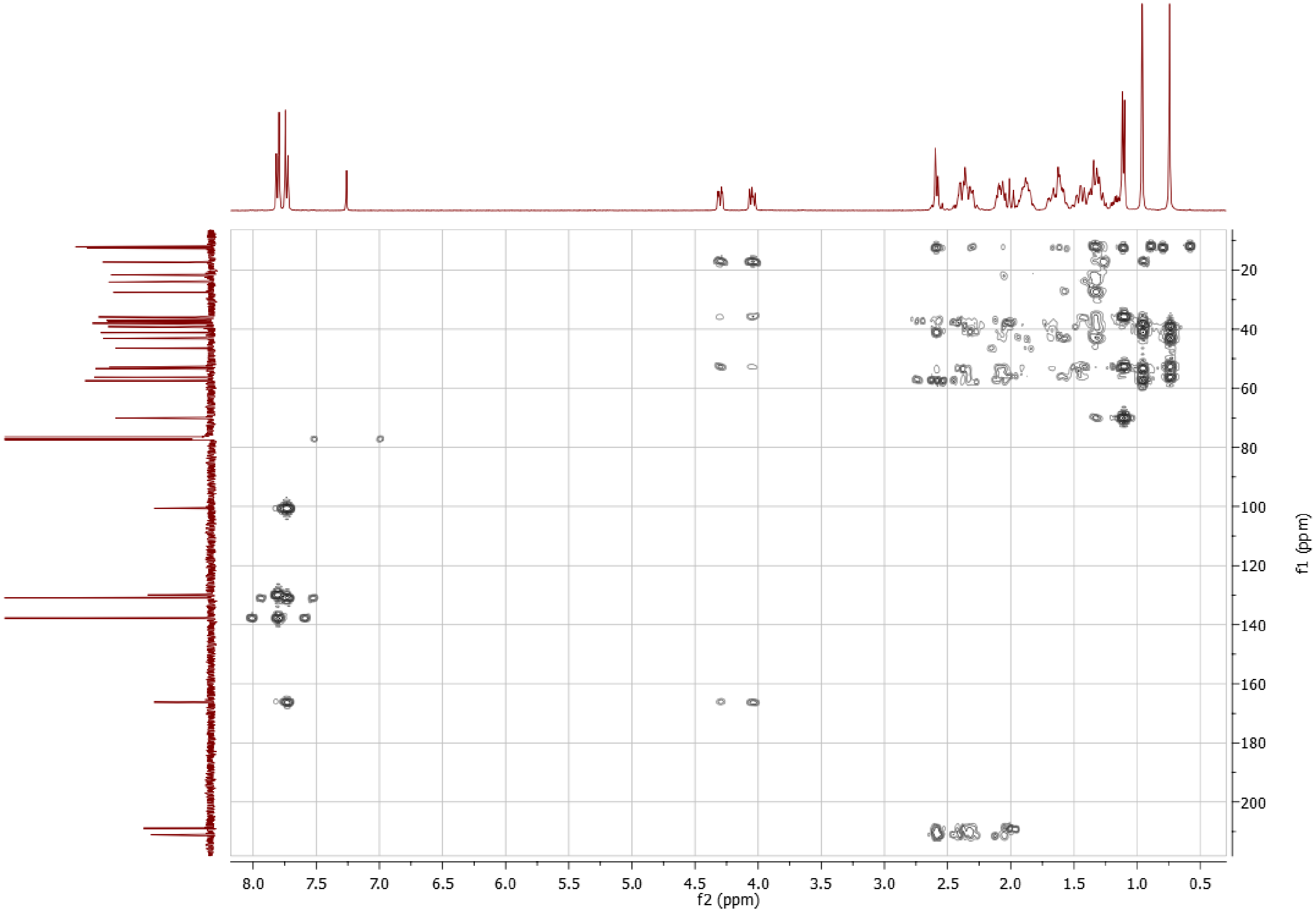

S65. 2D HMBC NMR spectrum of *3,6-dioxo-23,24-dinor-5α-cholan-(4-iodo)-benzoate-22-yl* (**25**).

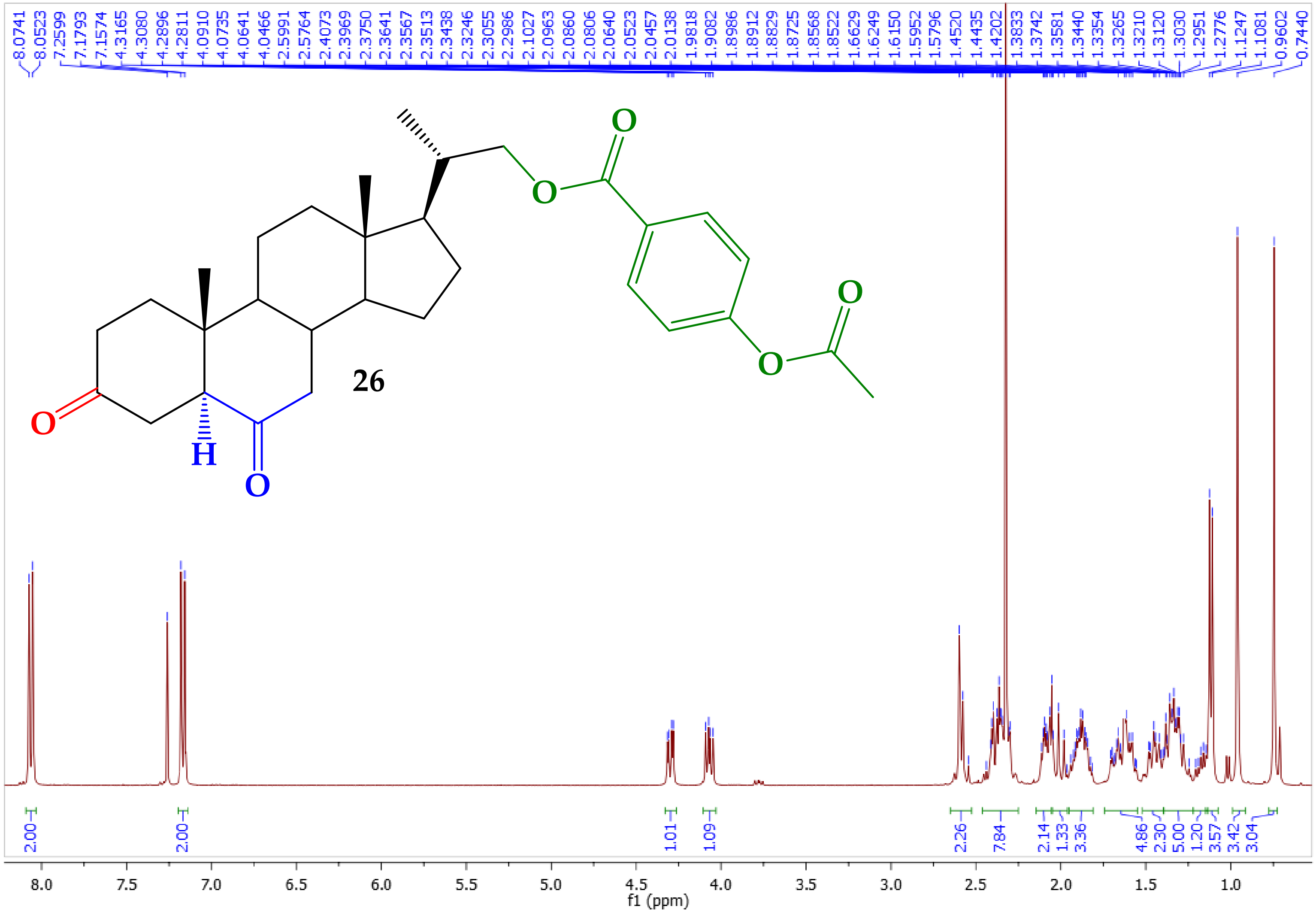

S66. ^{1}H NMR spectrum of *3,6-dioxo-23,24-dinor-5α-cholan-(4-acetoxy)-benzoate-22-yl* (**26**).

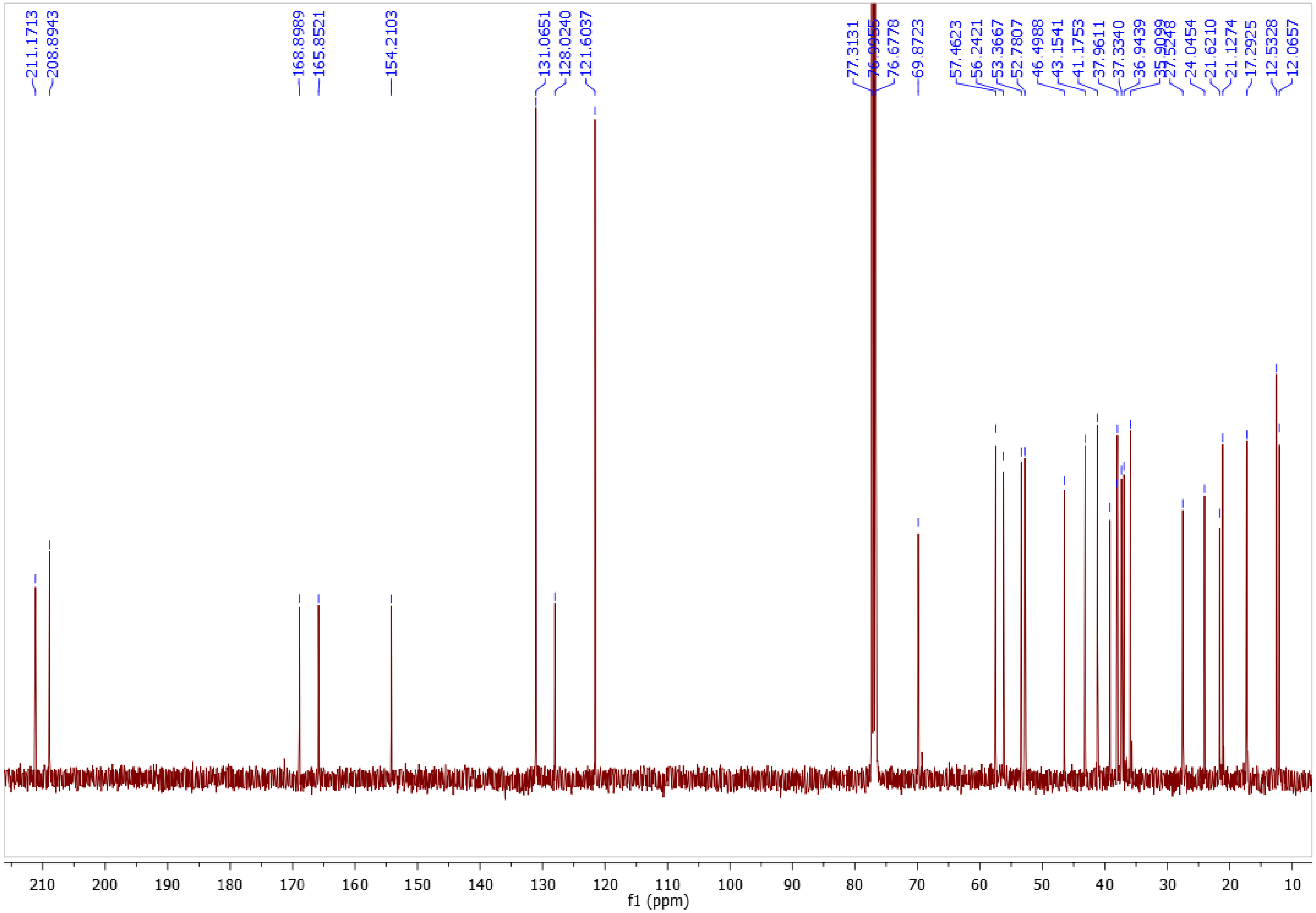

S67. ^{13}C NMR spectrum of *3,6-dioxo-23,24-dinor-5α-cholan-(4-acetoxy)-benzoate-22-yl* (**26**).

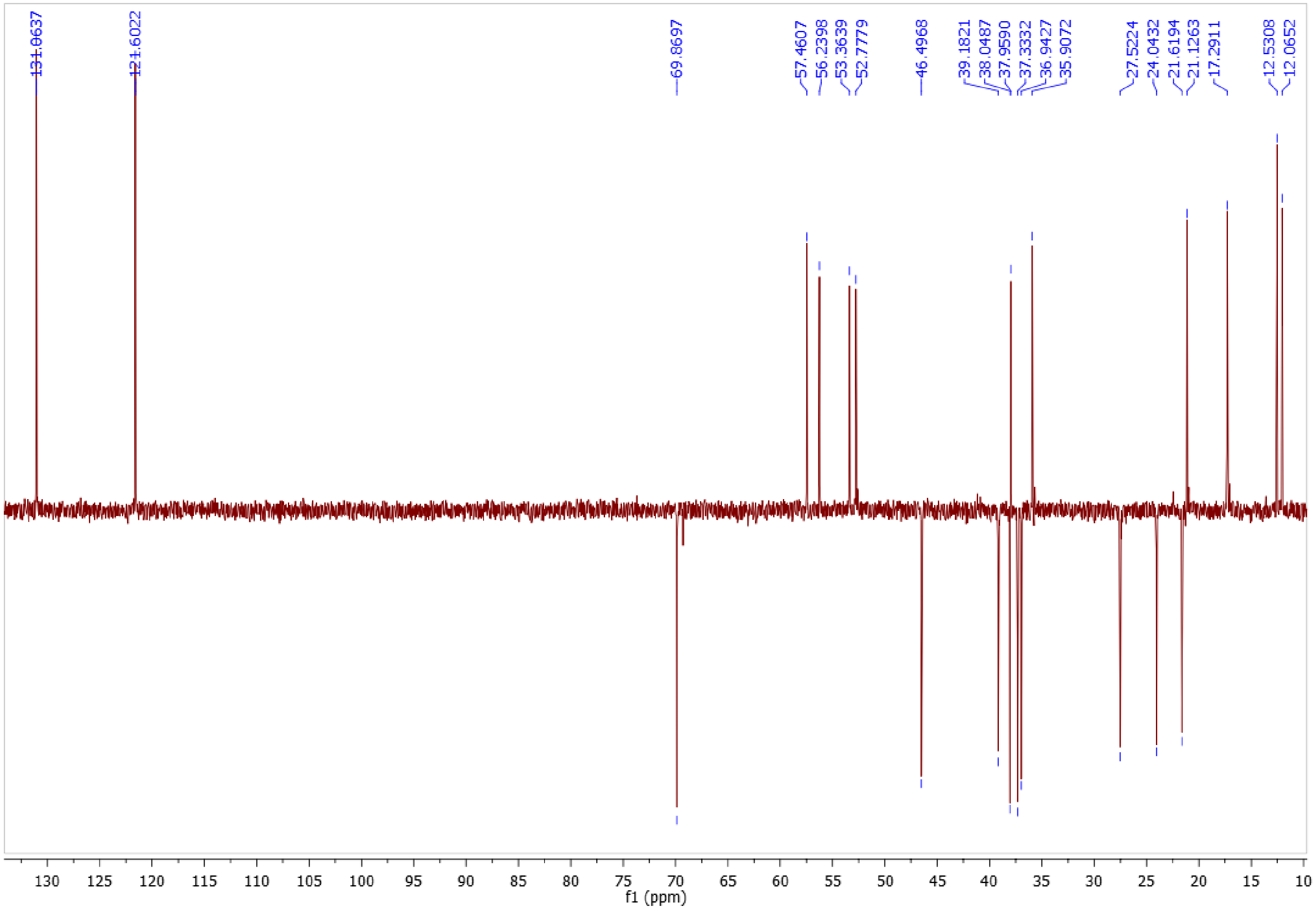

S68. ^{13}C DEPT-135 NMR spectrum of *3,6-dioxo-23,24-dinor-5α-cholan-(4-acetoxy)-benzoate-22-yl* (**26**).

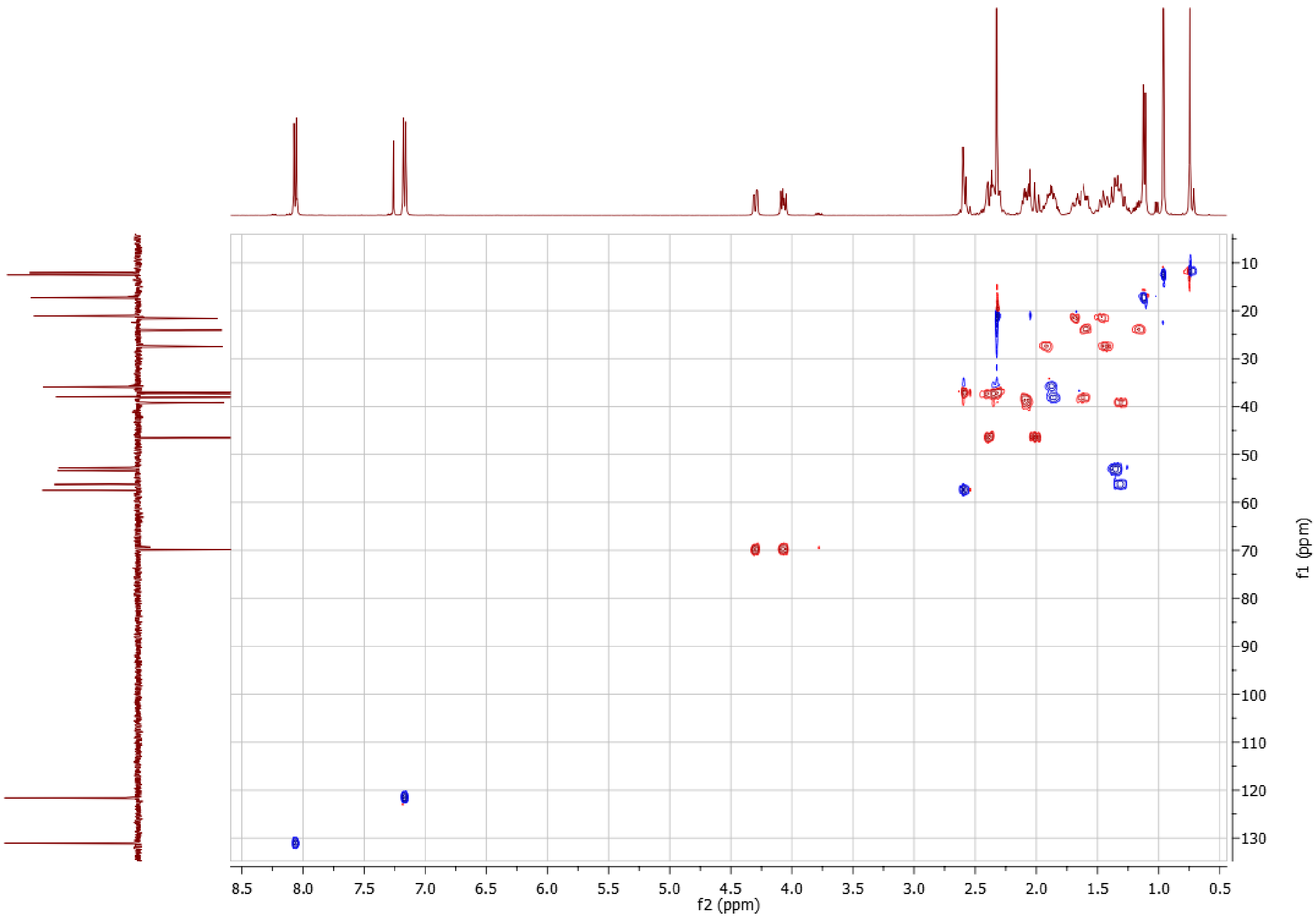

S69. 2D HSQC NMR spectrum of *3,6-dioxo-23,24-dinor-5α-cholan-(4-acetoxy)-benzoate-22-yl* (**26**).

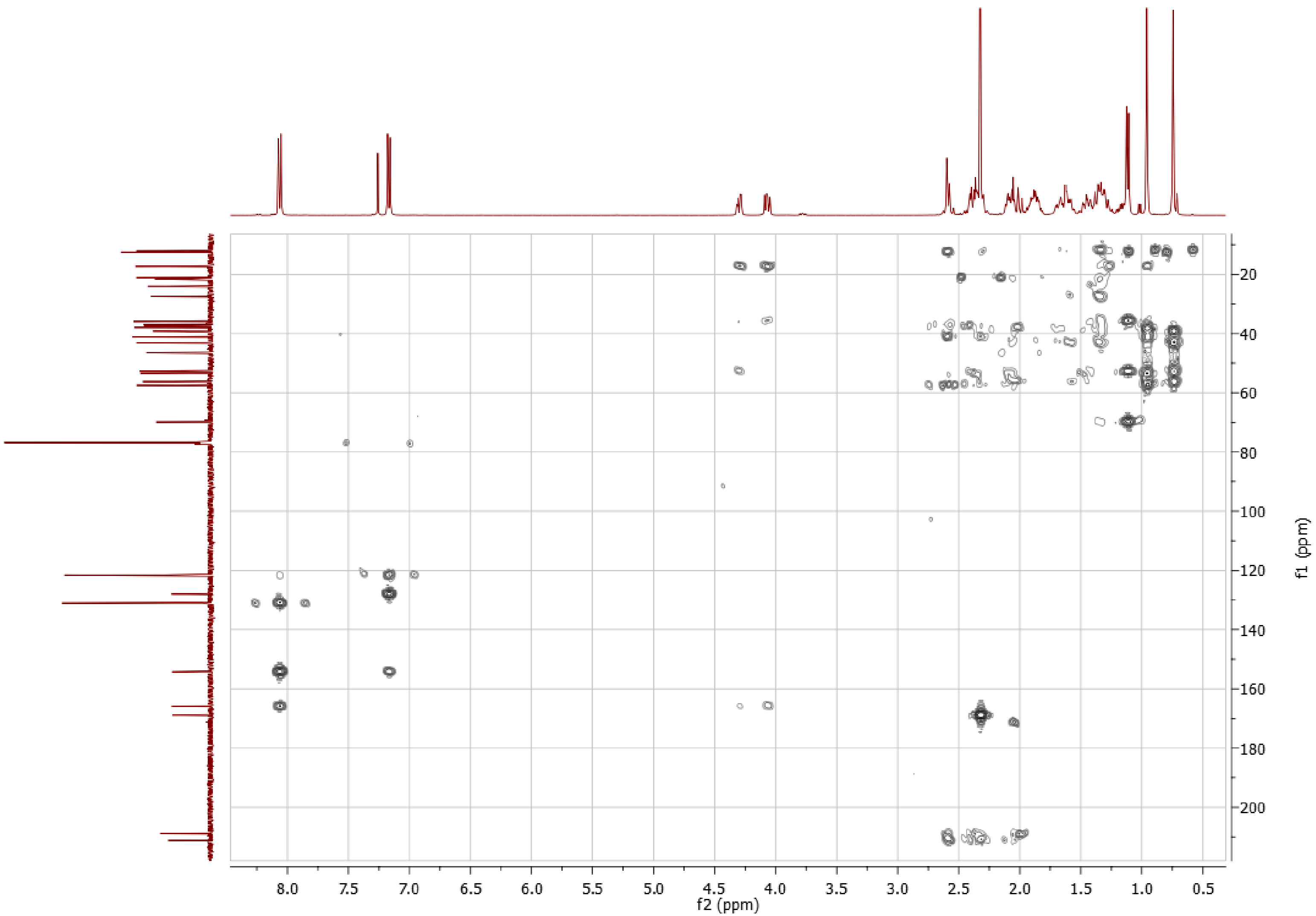

S70. 2D HMBC NMR spectrum of *3,6-dioxo-23,24-dinor-5α-cholan-(4-acetoxy)-benzoate-22-yl* (**26**).

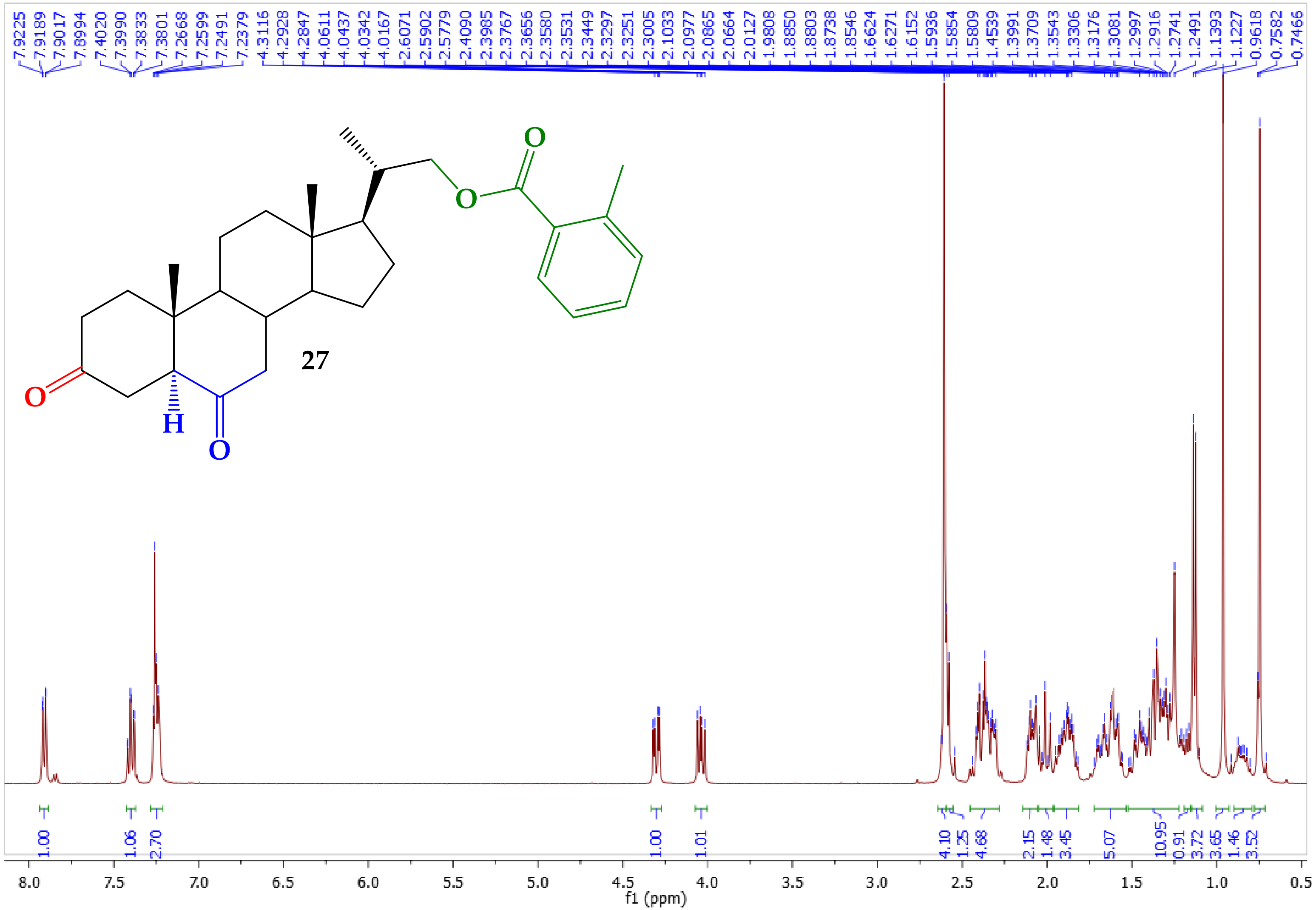

S71. ^{1}H NMR spectrum of *3,6-dioxo-23,24-dinor-5α-cholan-(2-methyl)-benzoate-22-yl* (**27**).

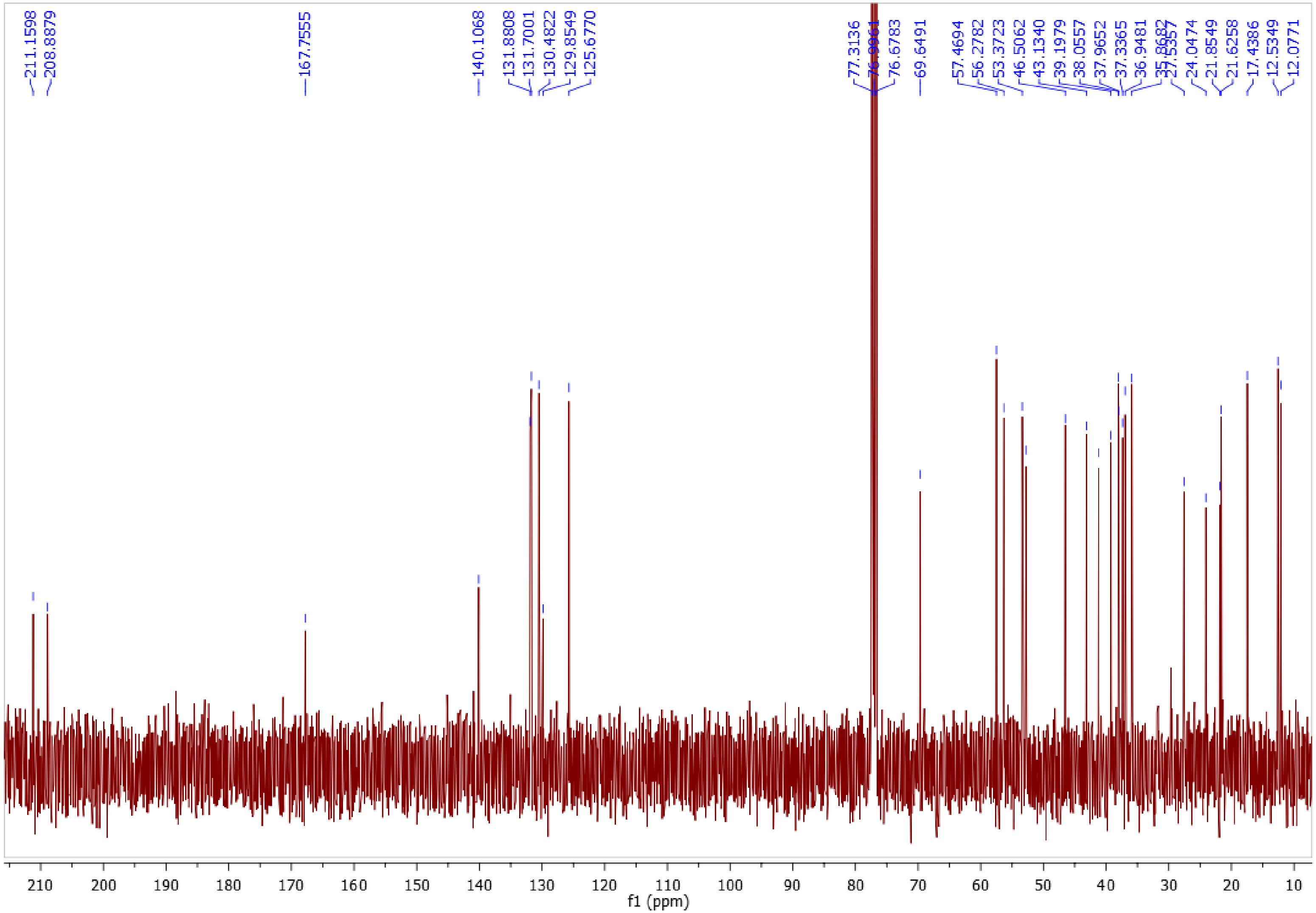

S72. ^{13}C NMR spectrum of *3,6-dioxo-23,24-dinor-5α-cholan-(2-methyl)-benzoate-22-yl* (**27**).

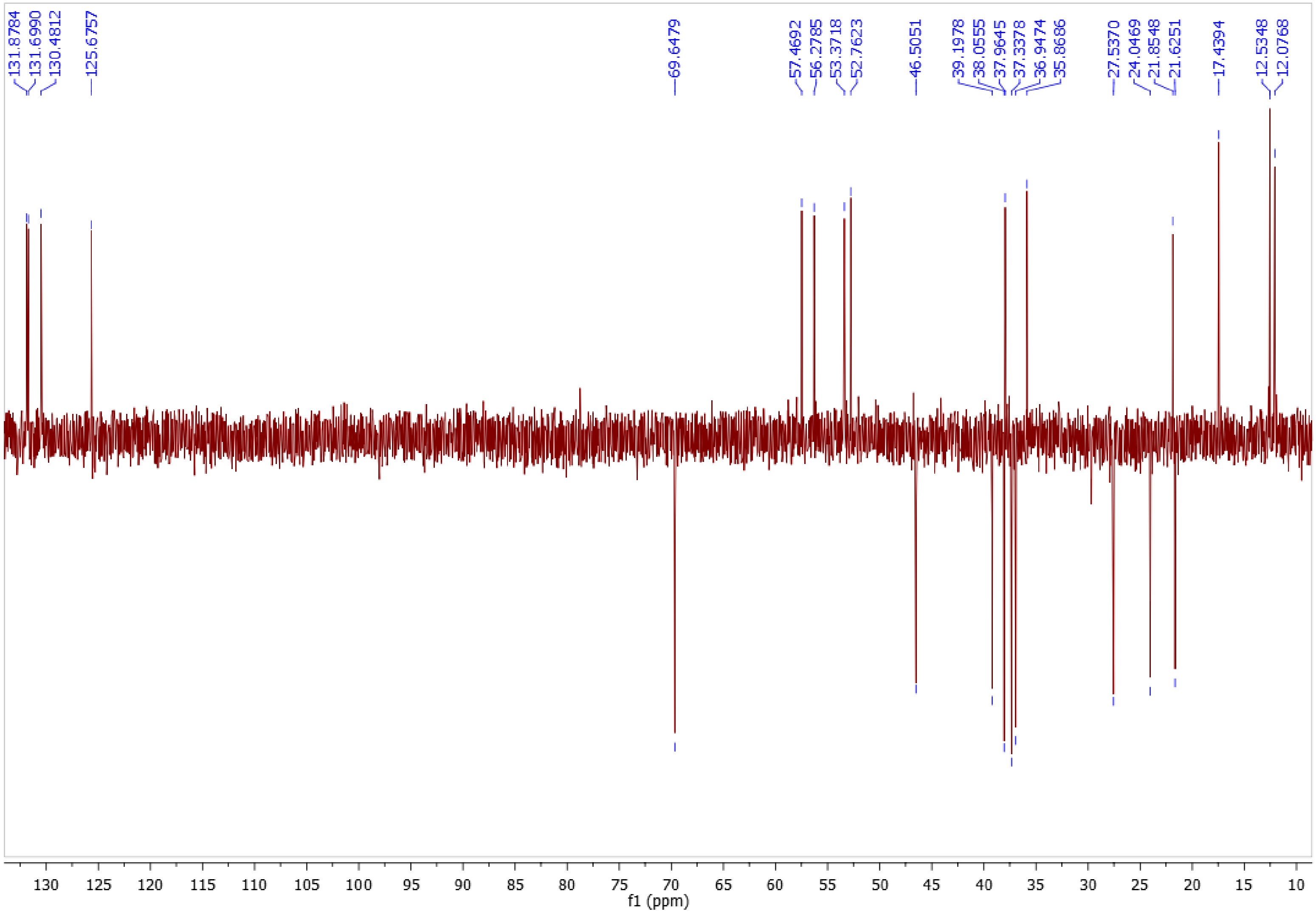

S73. ^{13}C DEPT-135 NMR spectrum of *3,6-dioxo-23,24-dinor-5α-cholan-(2-methyl)-benzoate-22-yl* (**27**).

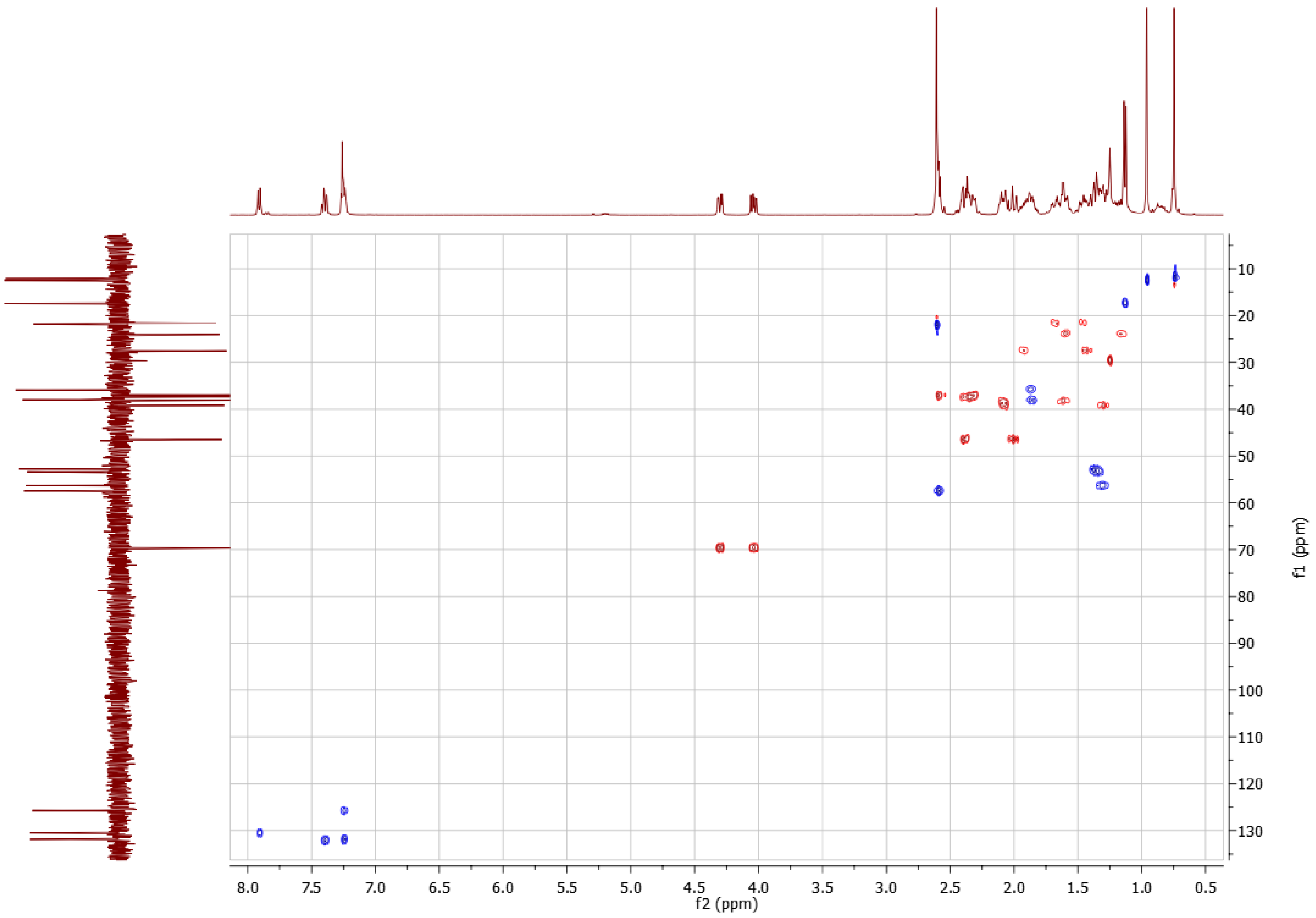

S74. 2D HSQC NMR spectrum of *3,6-dioxo-23,24-dinor-5α-cholan-(2-methyl)-benzoate-22-yl* (**27**).

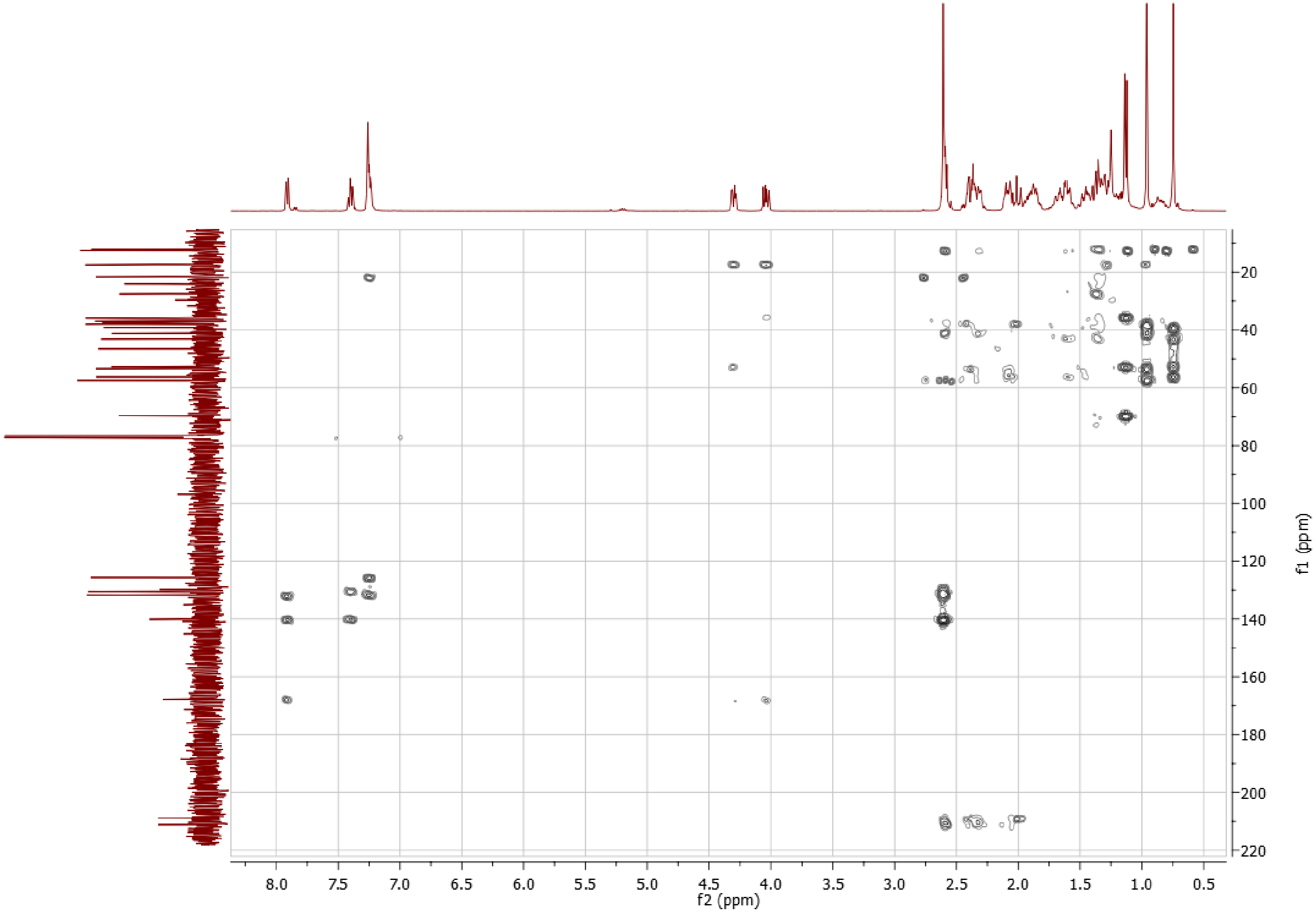

S75. 2D HMBC NMR spectrum of *3,6-dioxo-23,24-dinor-5α-cholan-(2-methyl)-benzoate-22-yl* (**27**).

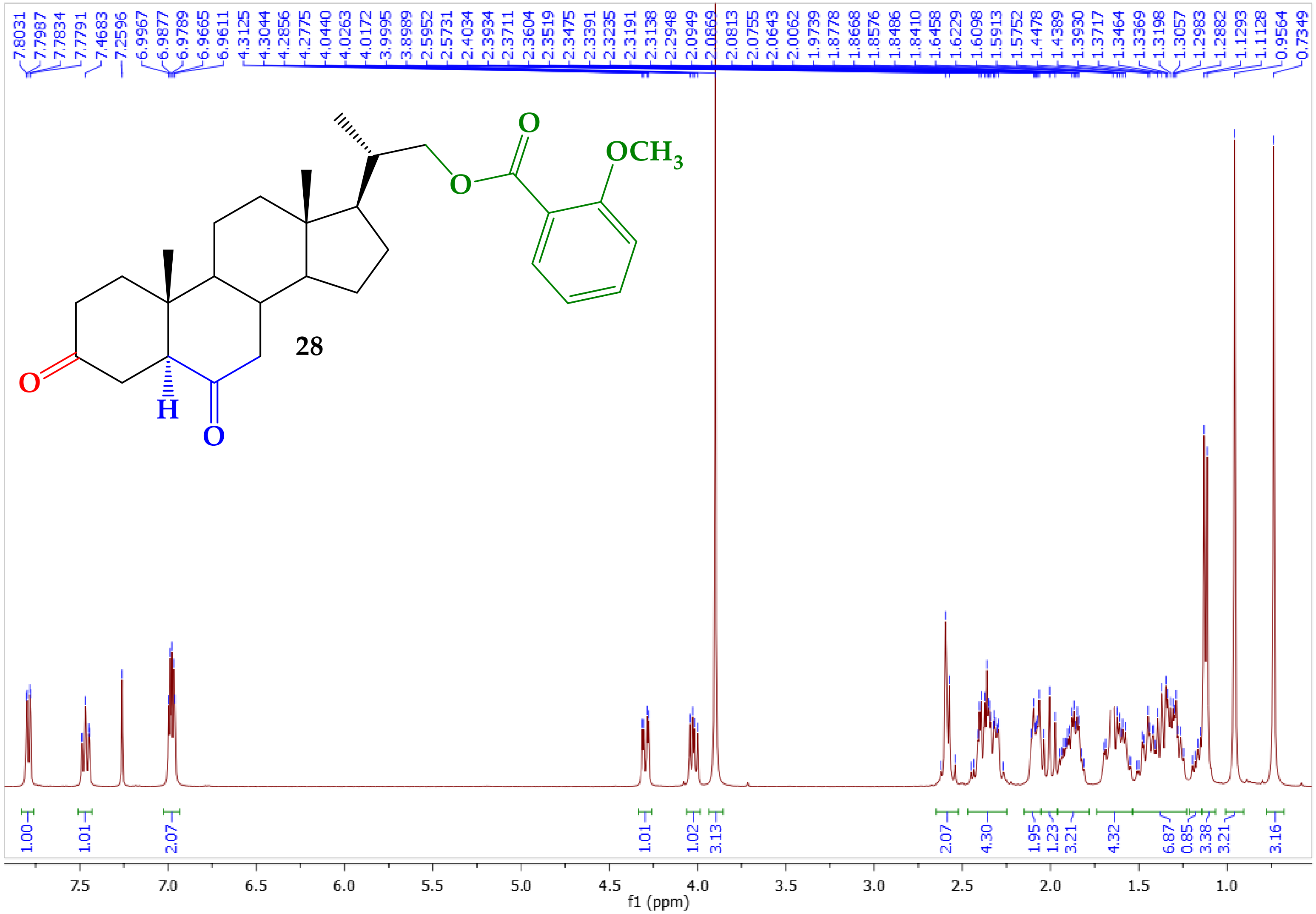

S76. ^{1}H NMR spectrum of *3,6-dioxo-23,24-dinor-5α-cholan-(2-methoxy)-benzoate-22-yl* (**28**).

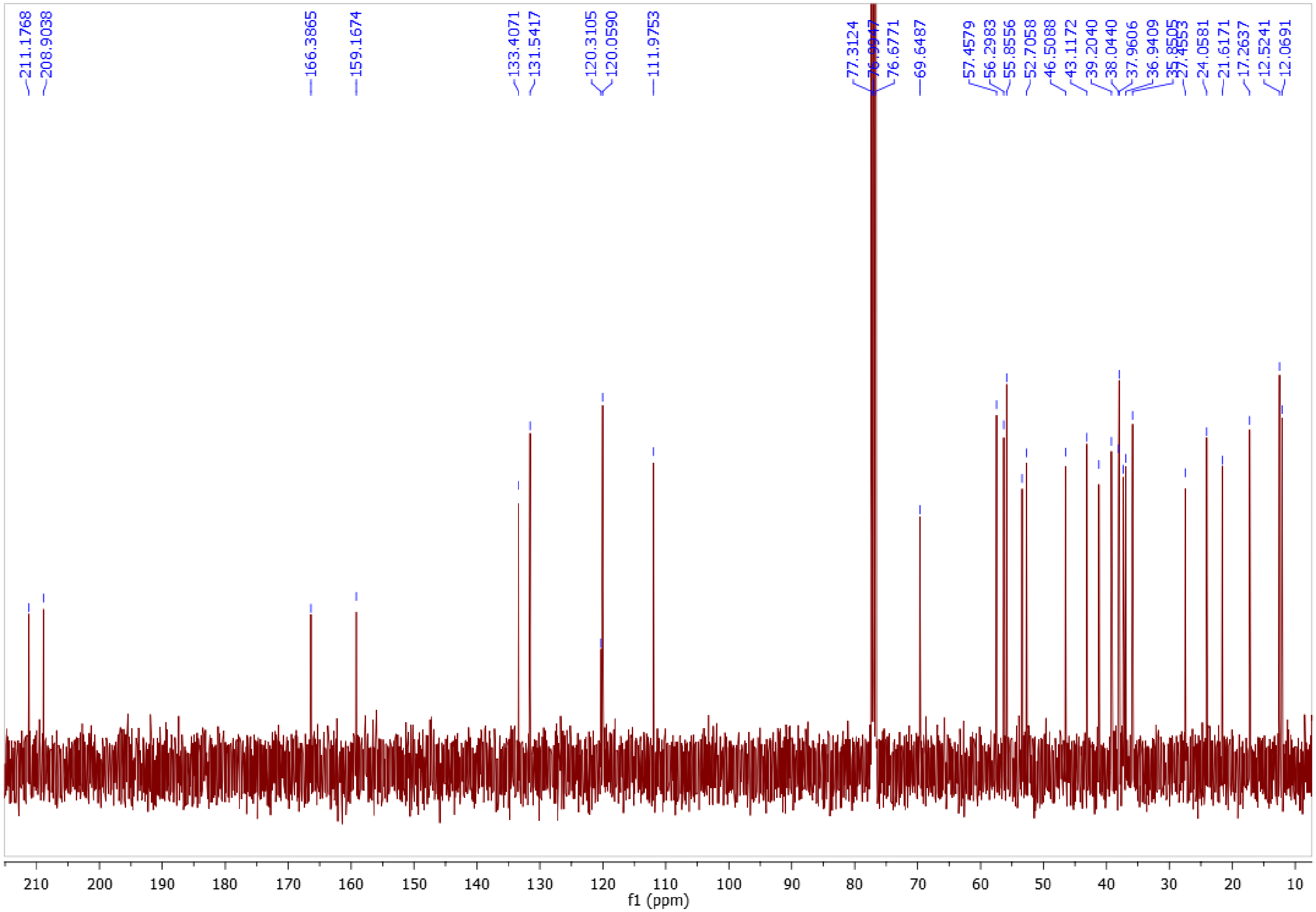

S77. ^{13}C NMR spectrum of *3,6-dioxo-23,24-dinor-5α-cholan-(2-methoxy)-benzoate-22-yl* (**28**).

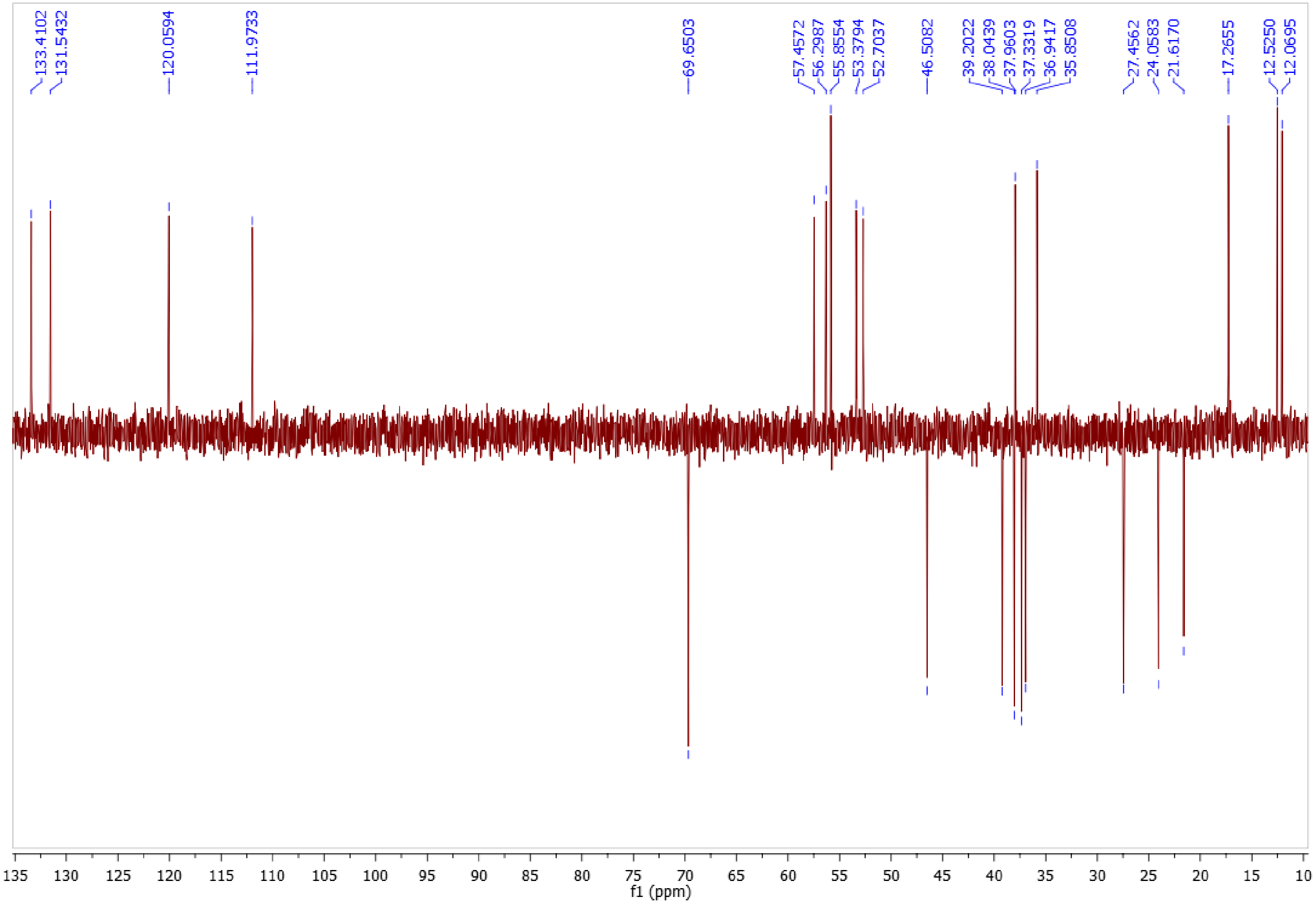

S78. ^{13}C DEPT-135 NMR spectrum of *3,6-dioxo-23,24-dinor-5α-cholan-(2-methoxy)-benzoate-22-yl* (**28**).

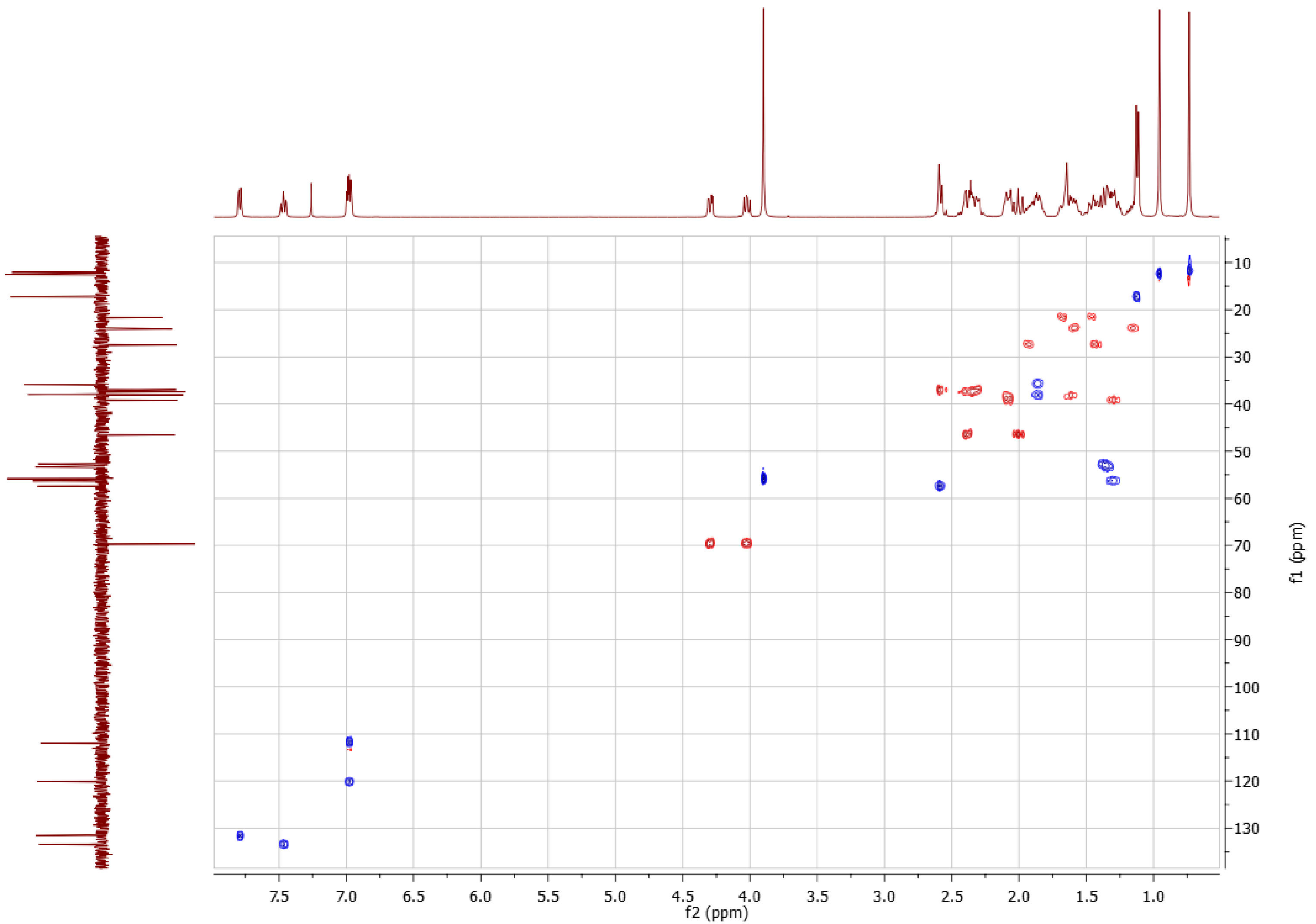

S79. 2D HSQC NMR spectrum of *3,6-dioxo-23,24-dinor-5α-cholan-(2-methoxy)-benzoate-22-yl* (**28**).

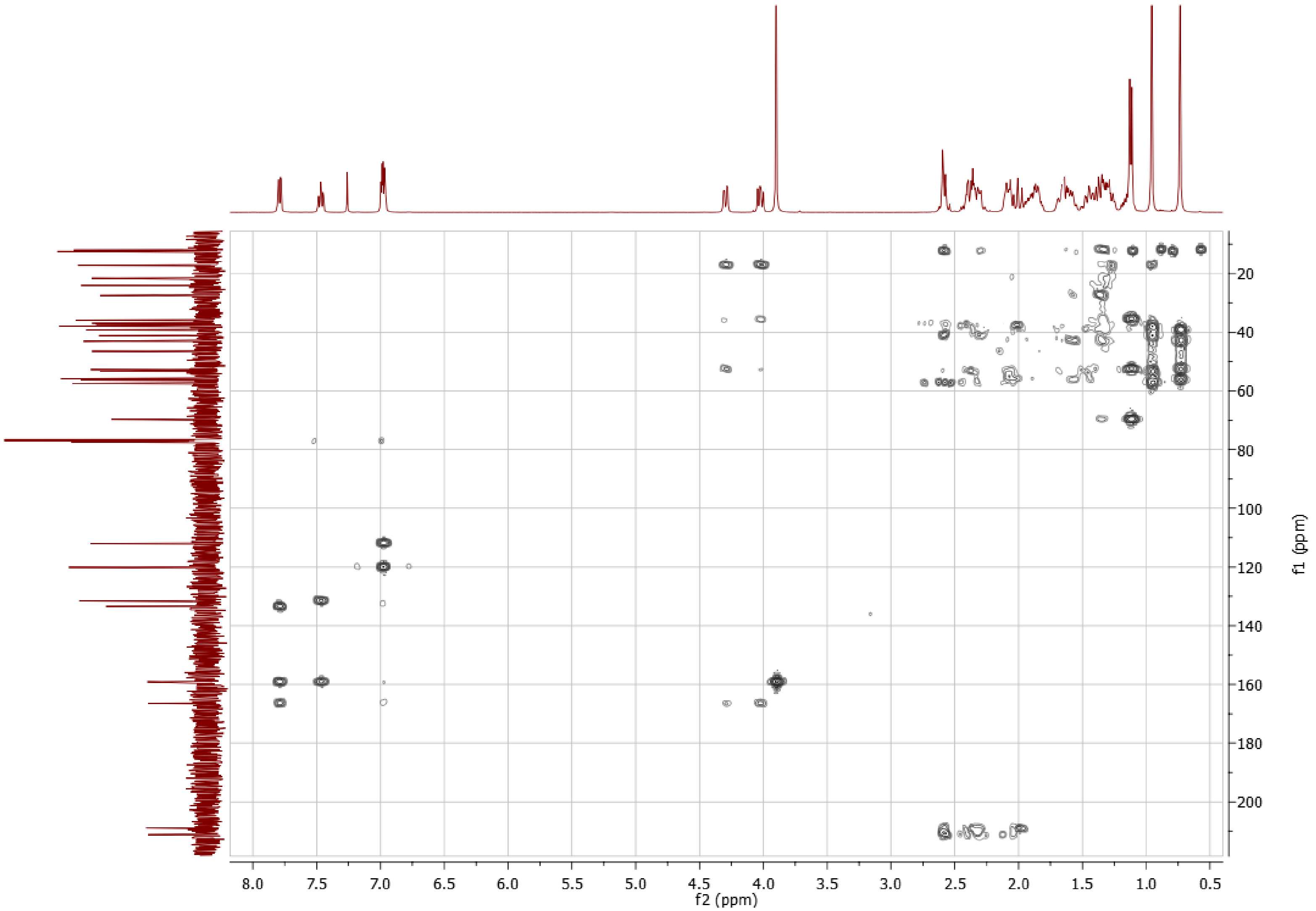

S80. 2D HMBC NMR spectrum of *3,6-dioxo-23,24-dinor-5α-cholan-(2-methoxy)-benzoate-22-yl* (**28**).

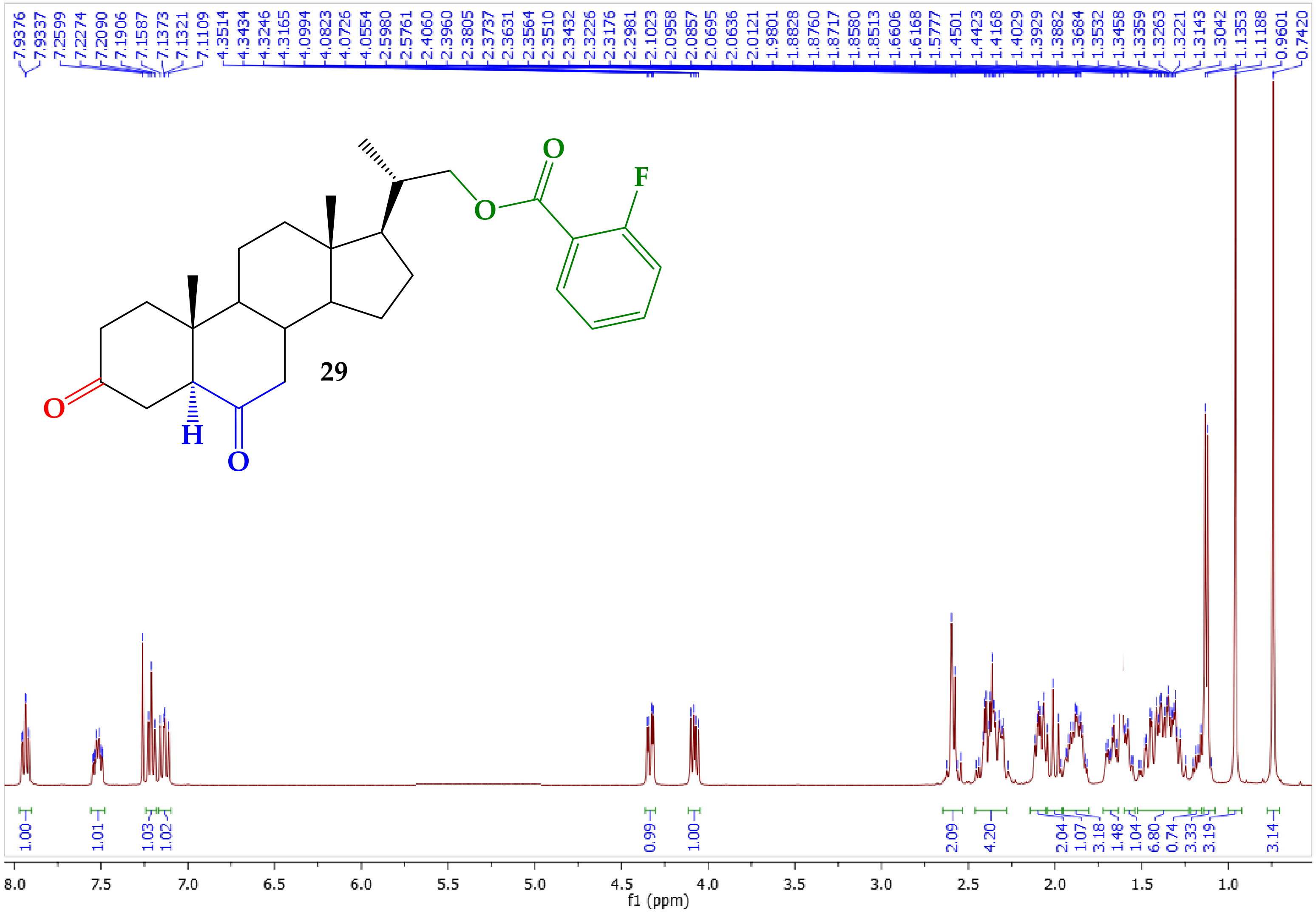

S81. ^{1}H NMR spectrum of *3,6-dioxo-23,24-dinor-5α-cholan-(2-fluoro)-benzoate-22-yl* (**29**).

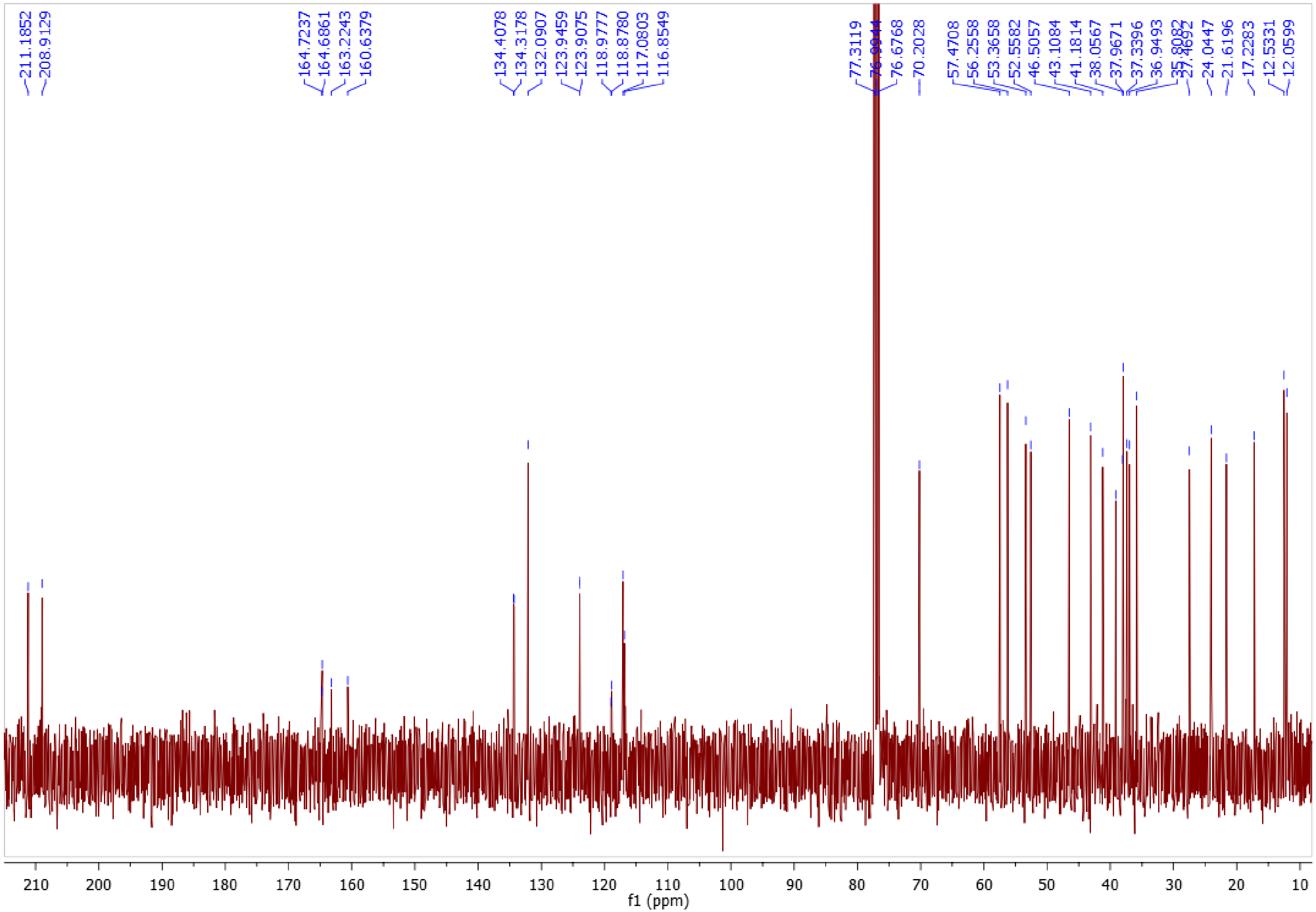

S82. ^{13}C NMR spectrum of *3,6-dioxo-23,24-dinor-5α-cholan-(2-fluoro)-benzoate-22-yl* (**29**).

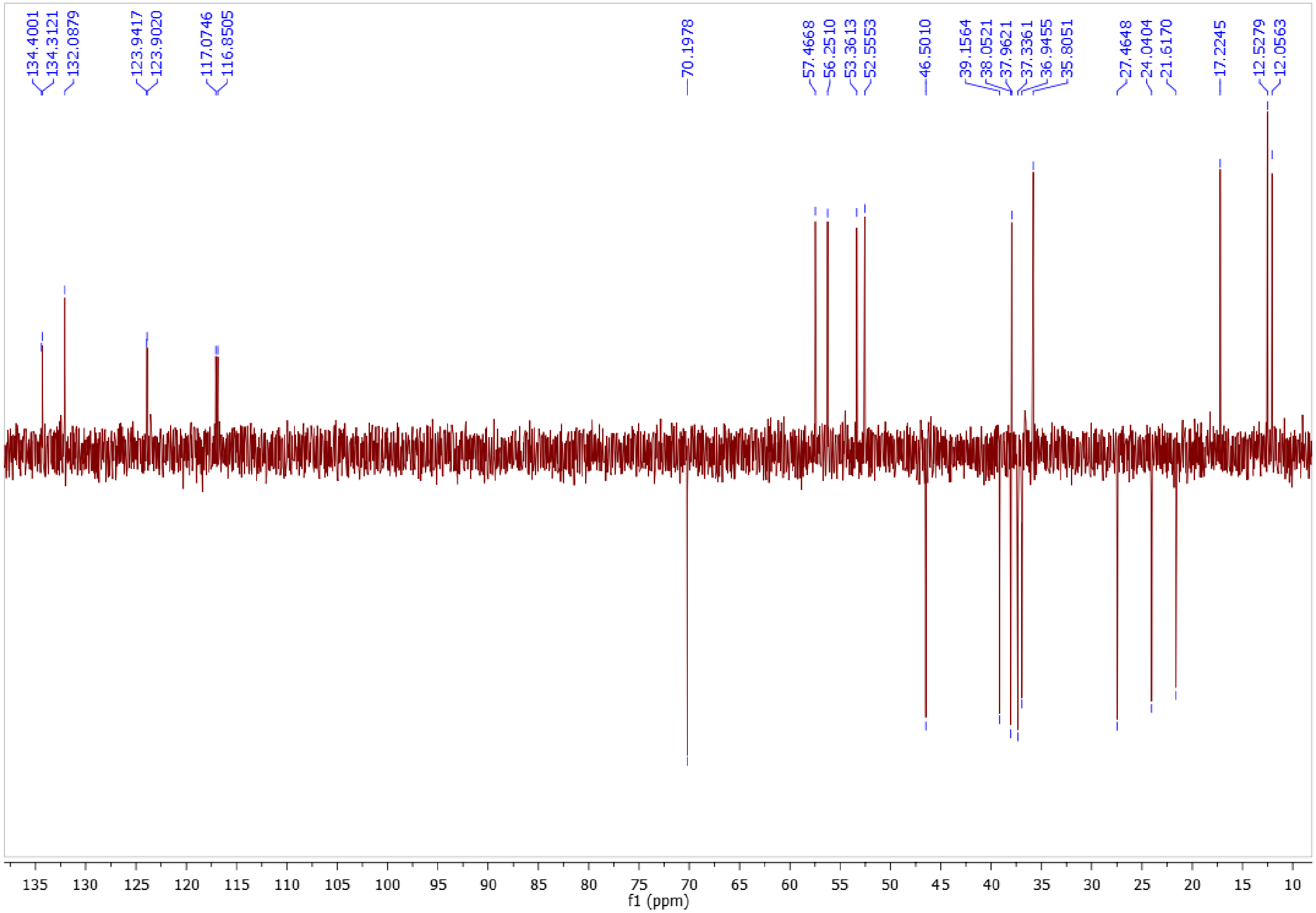

S83. ^{13}C DEPT-135 NMR spectrum of *3,6-dioxo-23,24-dinor-5α-cholan-(2-fluoro)-benzoate-22-yl* (**29**).

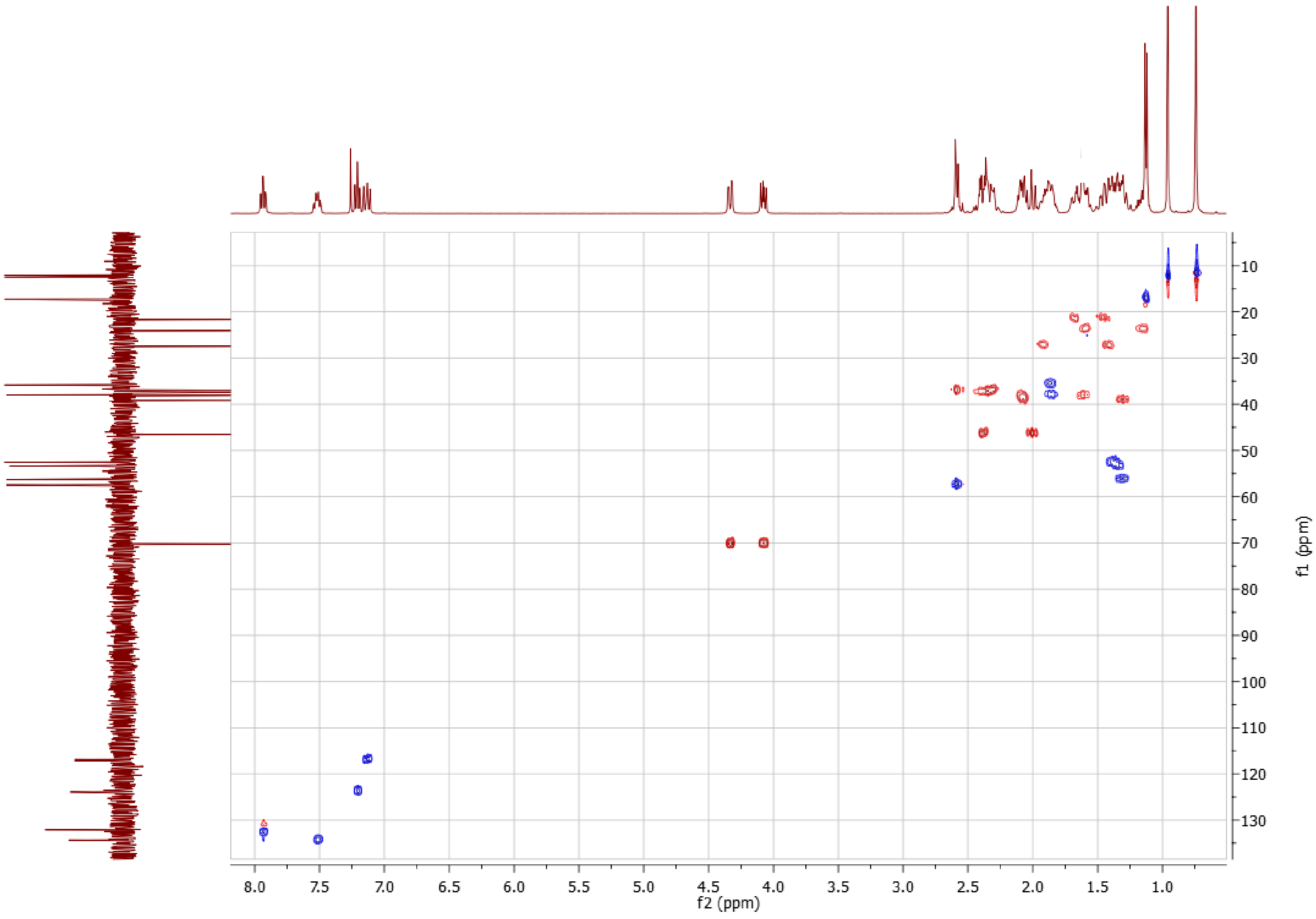

S84. 2D HSQC NMR spectrum of *3,6-dioxo-23,24-dinor-5α-cholan-(2-fluoro)-benzoate-22-yl* (**29**).

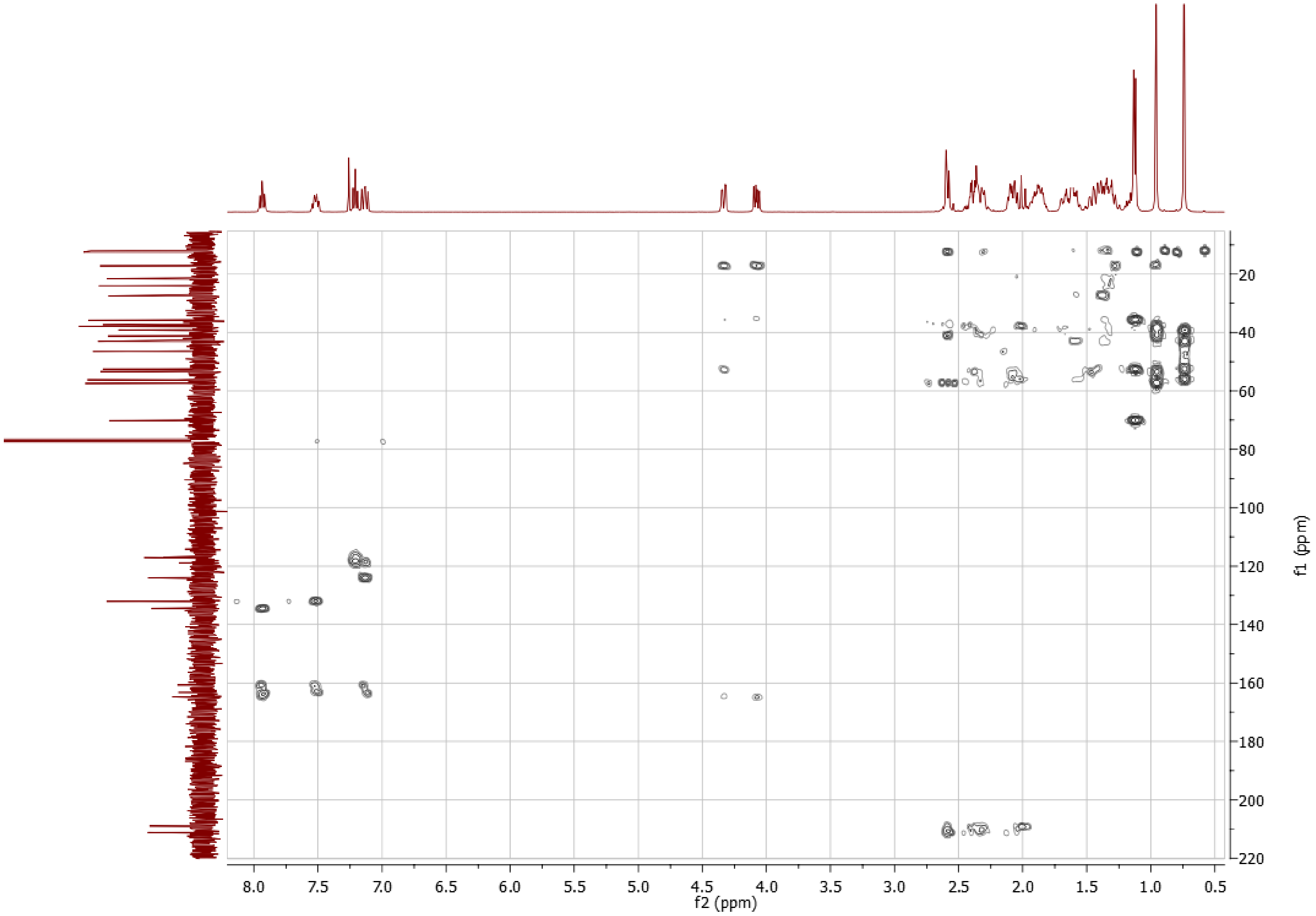

S85. 2D HMBC NMR spectrum of *3,6-dioxo-23,24-dinor-5α-cholan-(2-fluoro)-benzoate-22-yl* (**29**).

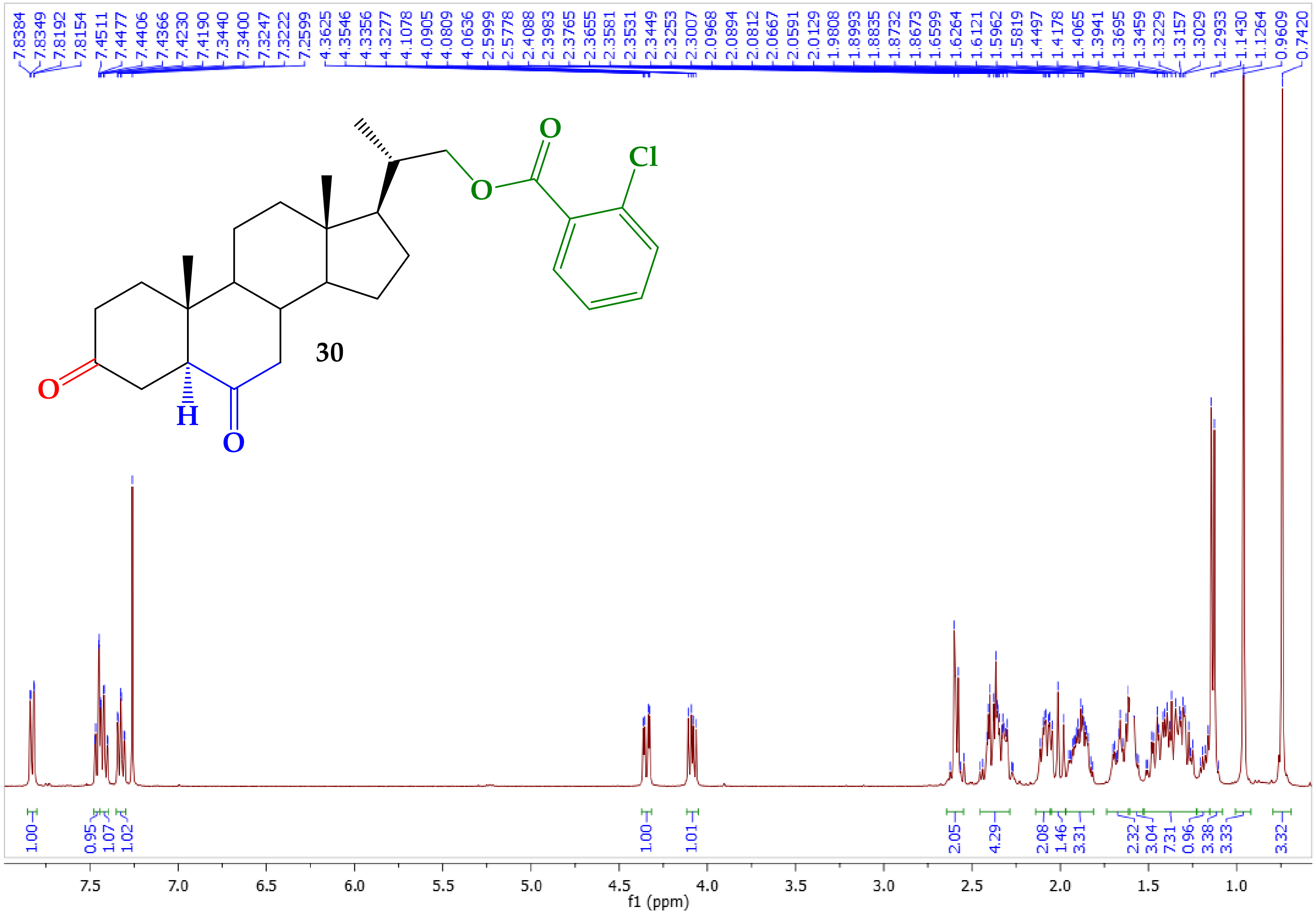

S86. ^{1}H NMR spectrum of *3,6-dioxo-23,24-dinor-5α-cholan-(2-chloro)-benzoate-22-yl* (***30***).

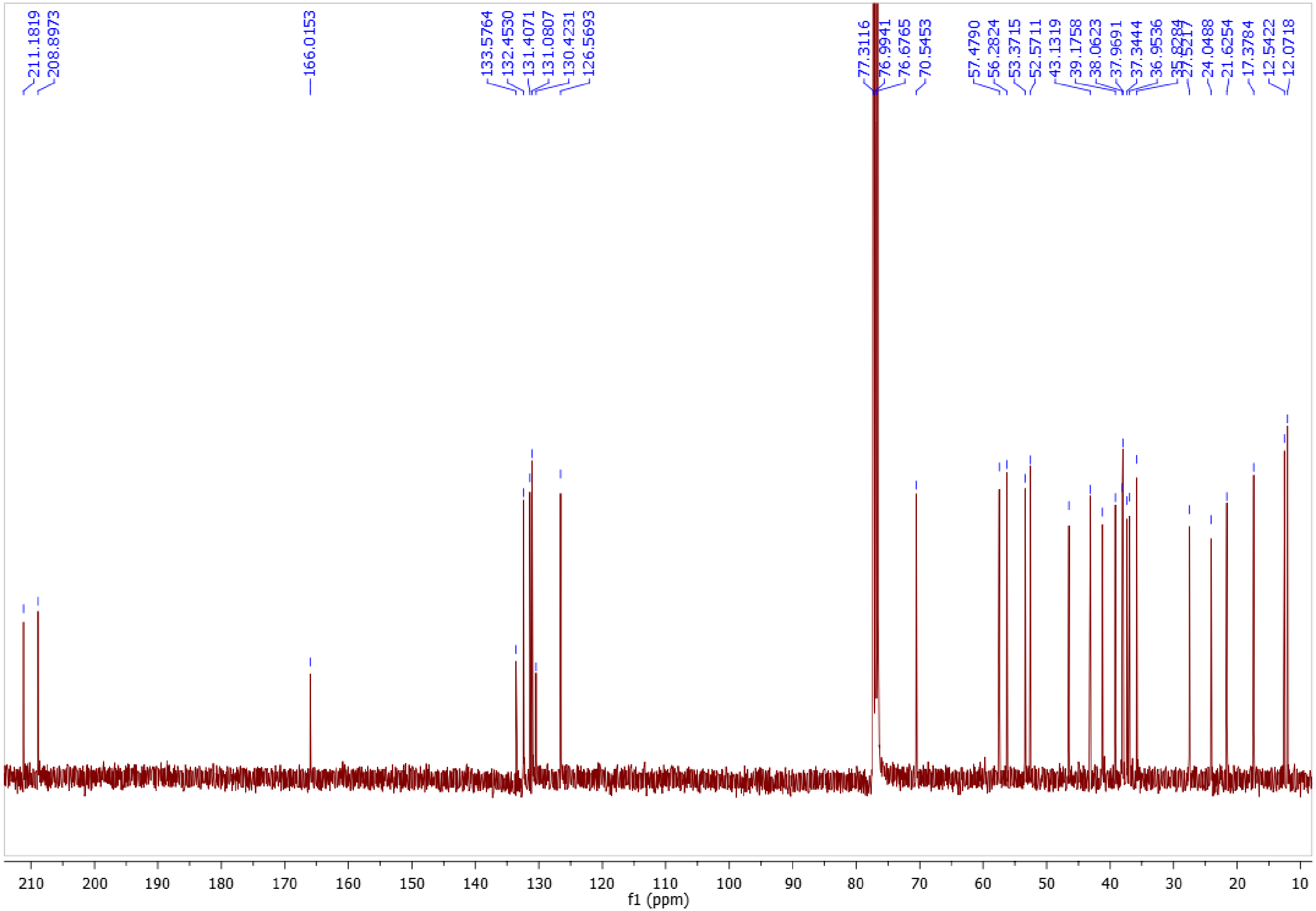

S87. ^{13}C NMR spectrum of *3,6-dioxo-23,24-dinor-5α-cholan-(2-chloro)-benzoate-22-yl* (***30***).

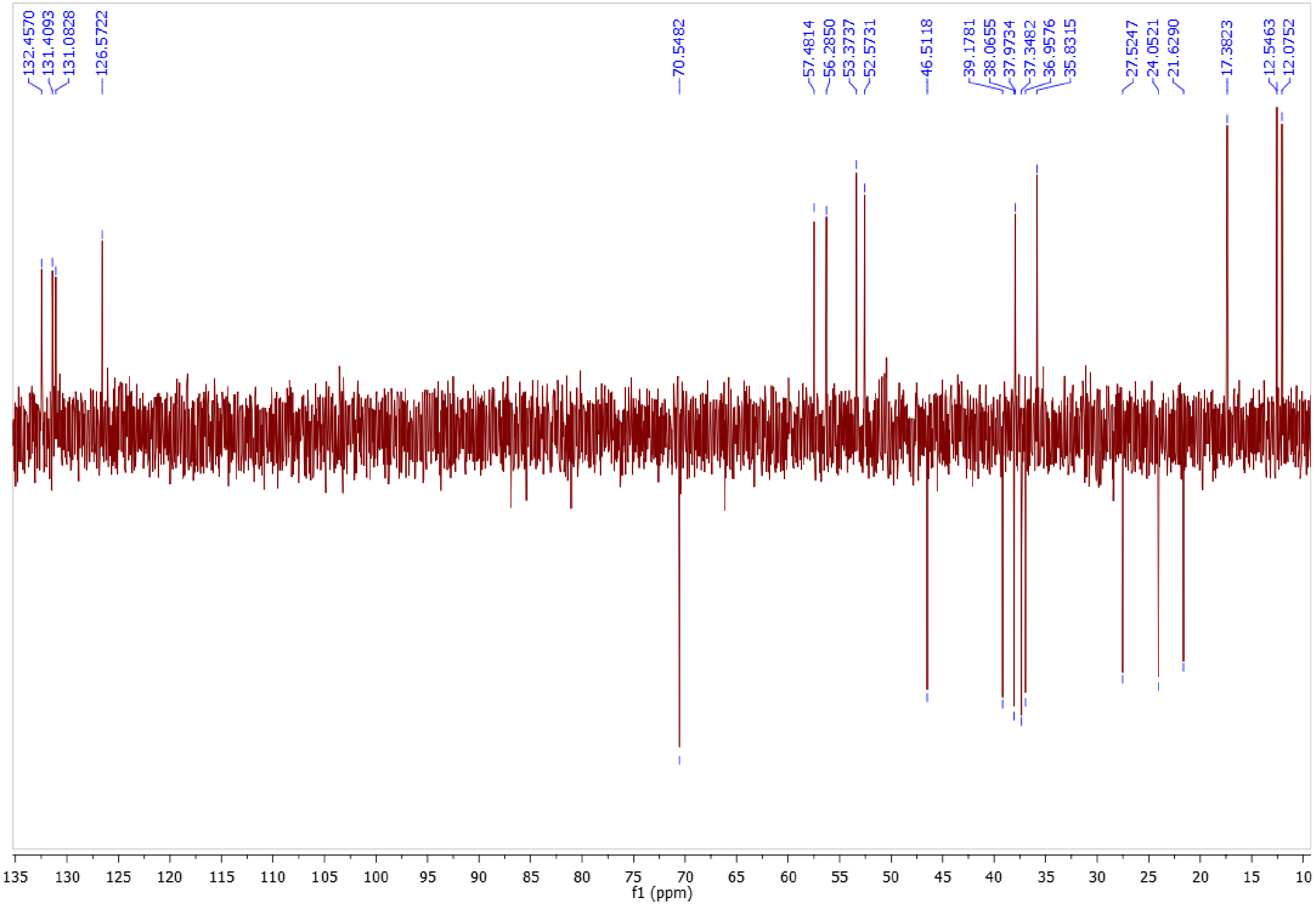

S88. ^{13}C DEPT-135 NMR spectrum of *3,6-dioxo-23,24-dinor-5α-cholan-(2-chloro)-benzoate-22-yl* (***30***).

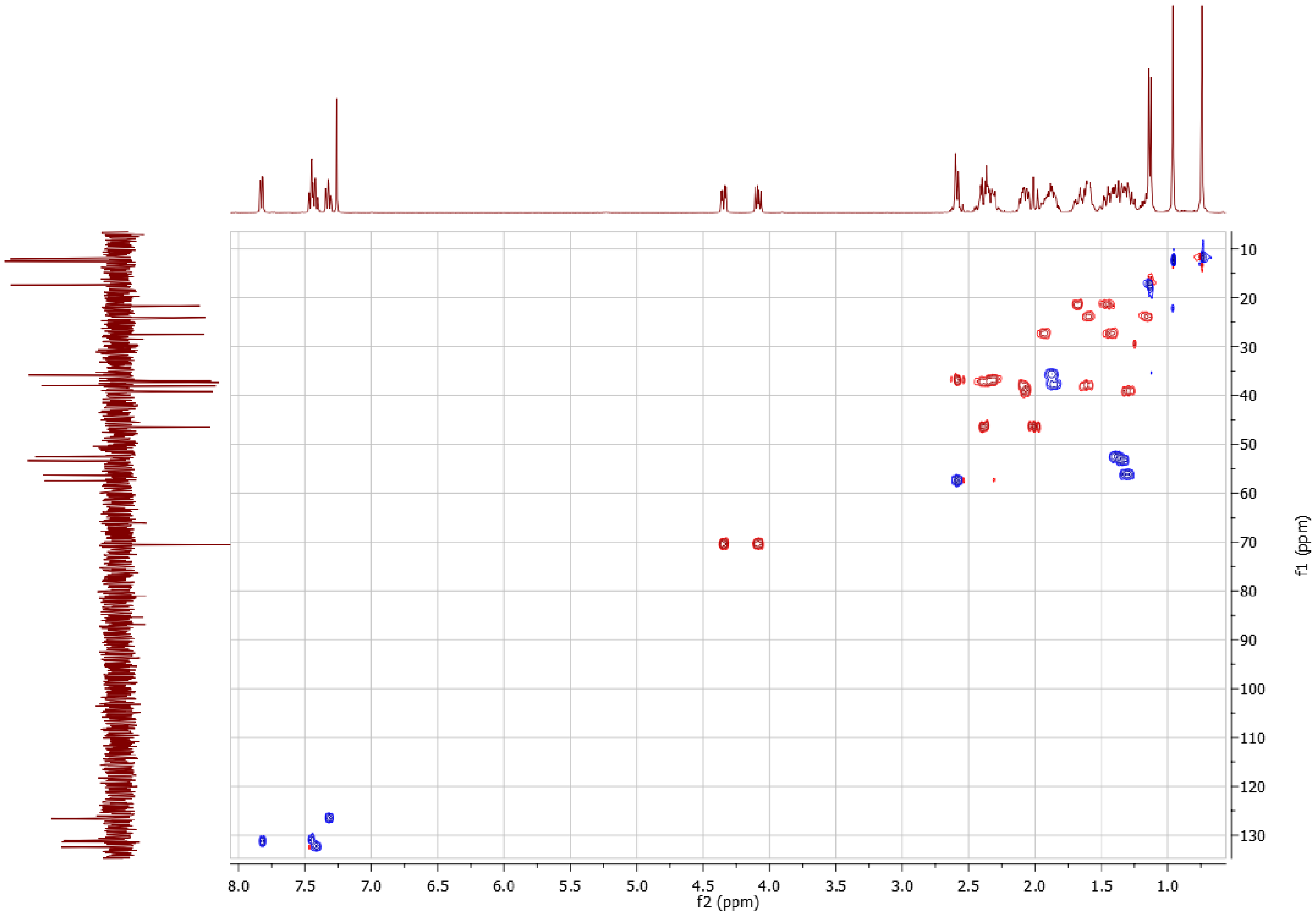

S89. 2D HSQC NMR spectrum of *3,6-dioxo-23,24-dinor-5α-cholan-(2-chloro)-benzoate-22-yl* (***30***).

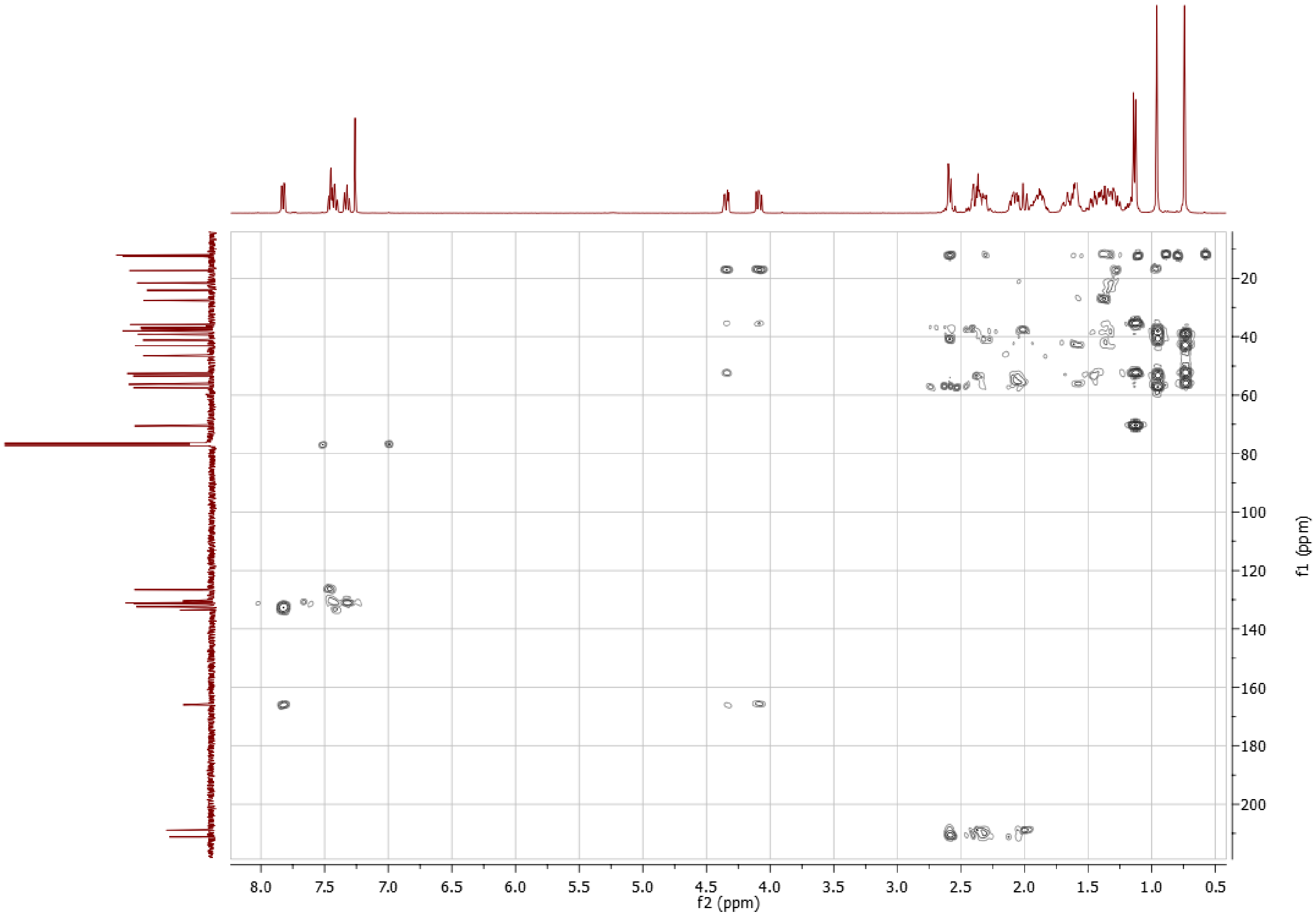

S90. 2D HMBC NMR spectrum of *3,6-dioxo-23,24-dinor-5α-cholan-(2-chloro)-benzoate-22-yl* (***30***).

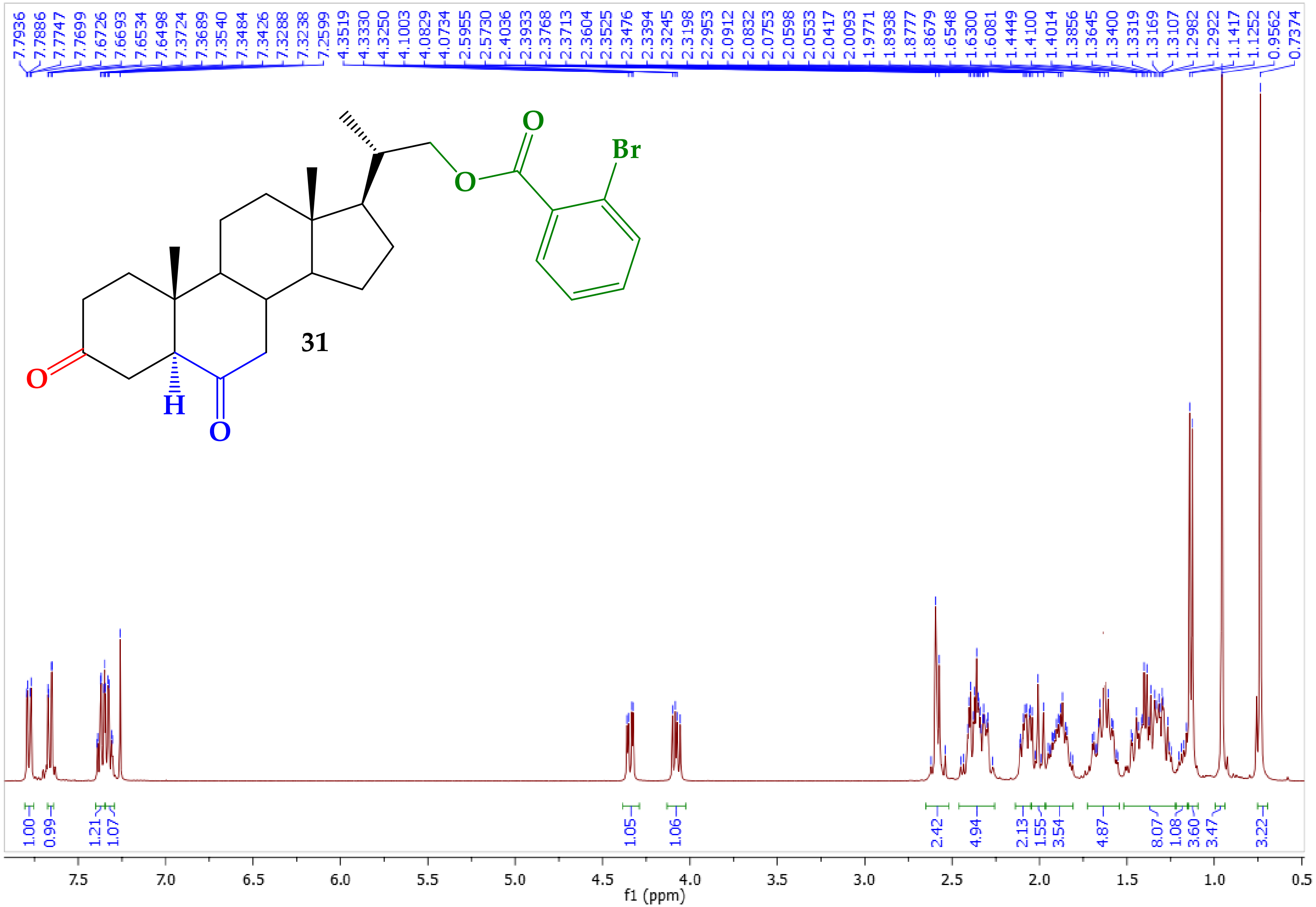

S91. ^{1}H NMR spectrum of *3,6-dioxo-23,24-dinor-5α-cholan-(2-bromo)-benzoate-22-yl* (**31**).

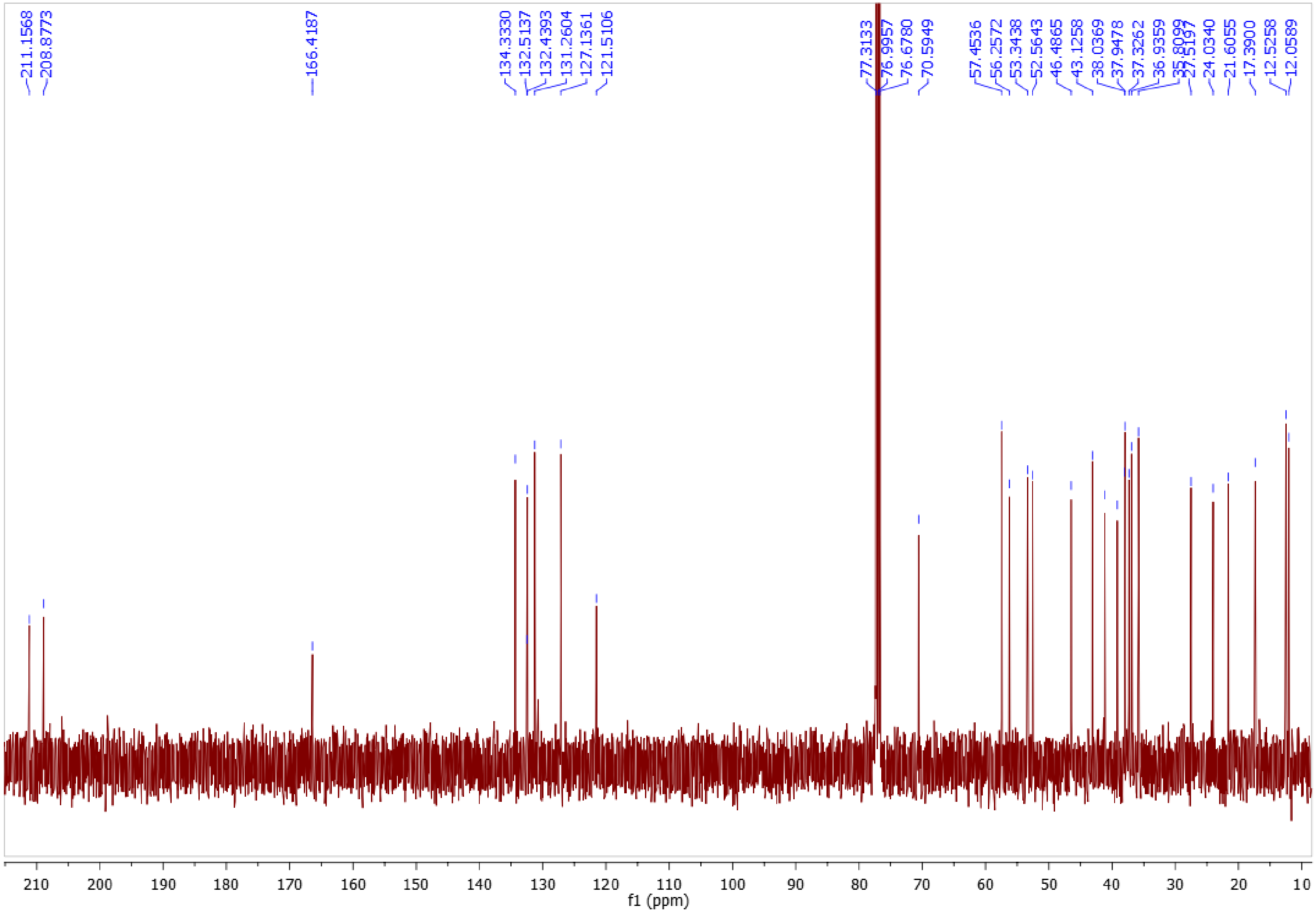

S92. ^{13}C NMR spectrum of *3,6-dioxo-23,24-dinor-5α-cholan-(2-bromo)-benzoate-22-yl* (***31***).

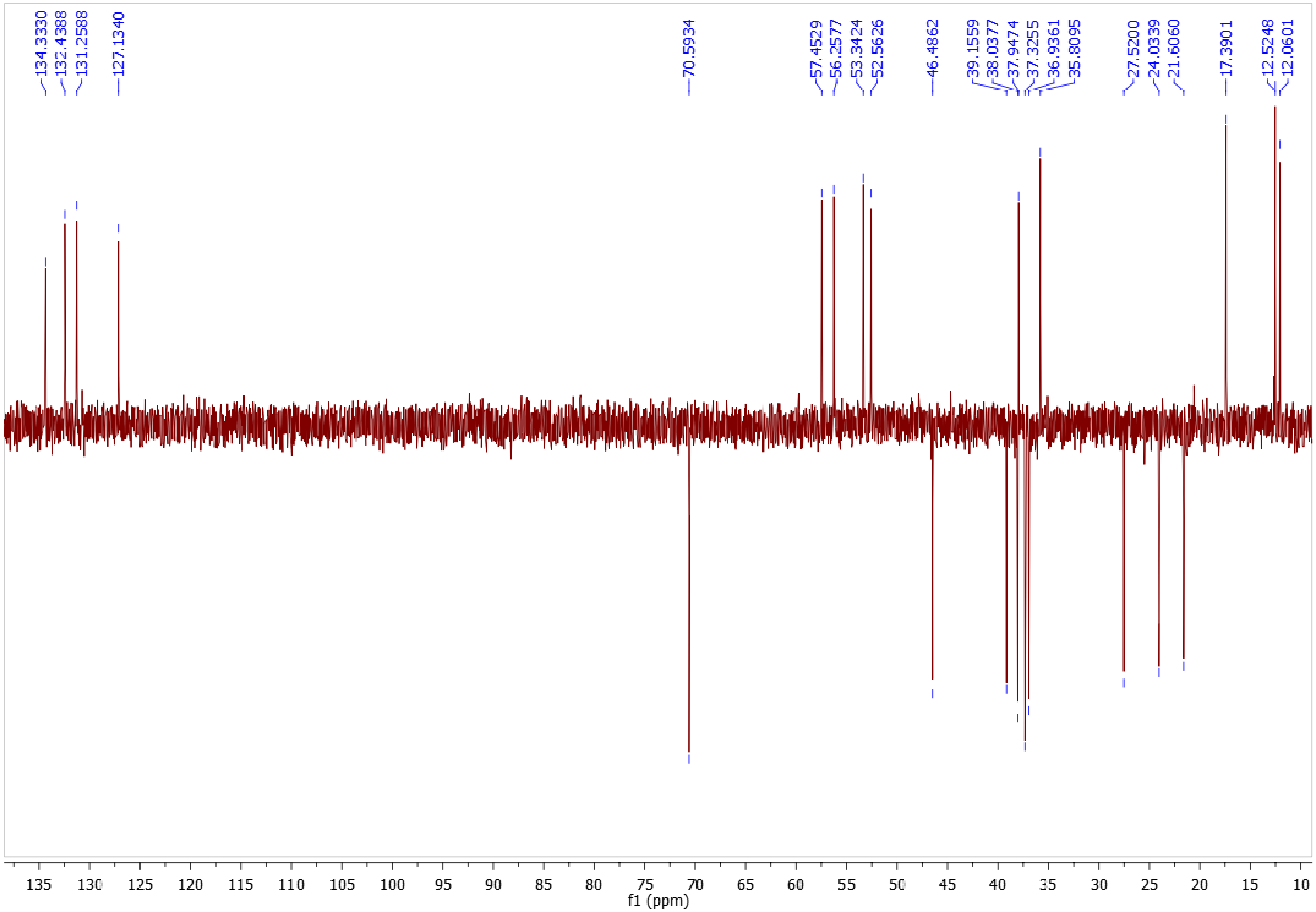

S93. ^{13}C DEPT-135 NMR spectrum of *3,6-dioxo-23,24-dinor-5α-cholan-(2-bromo)-benzoate-22-yl* (***31***).

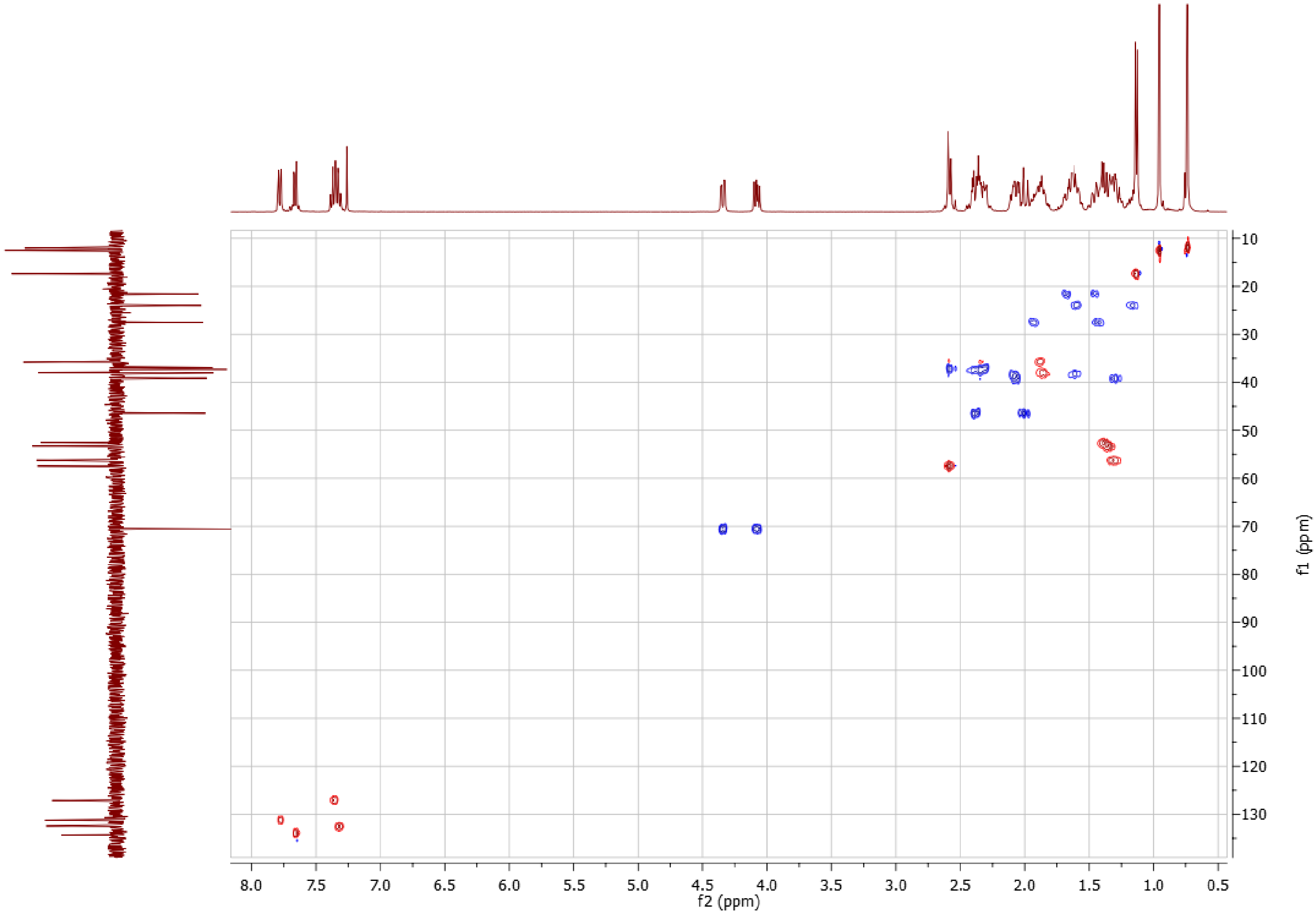

S94. 2D HSQC NMR spectrum of *3,6-dioxo-23,24-dinor-5α-cholan-(2-bromo)-benzoate-22-yl* (***31***).

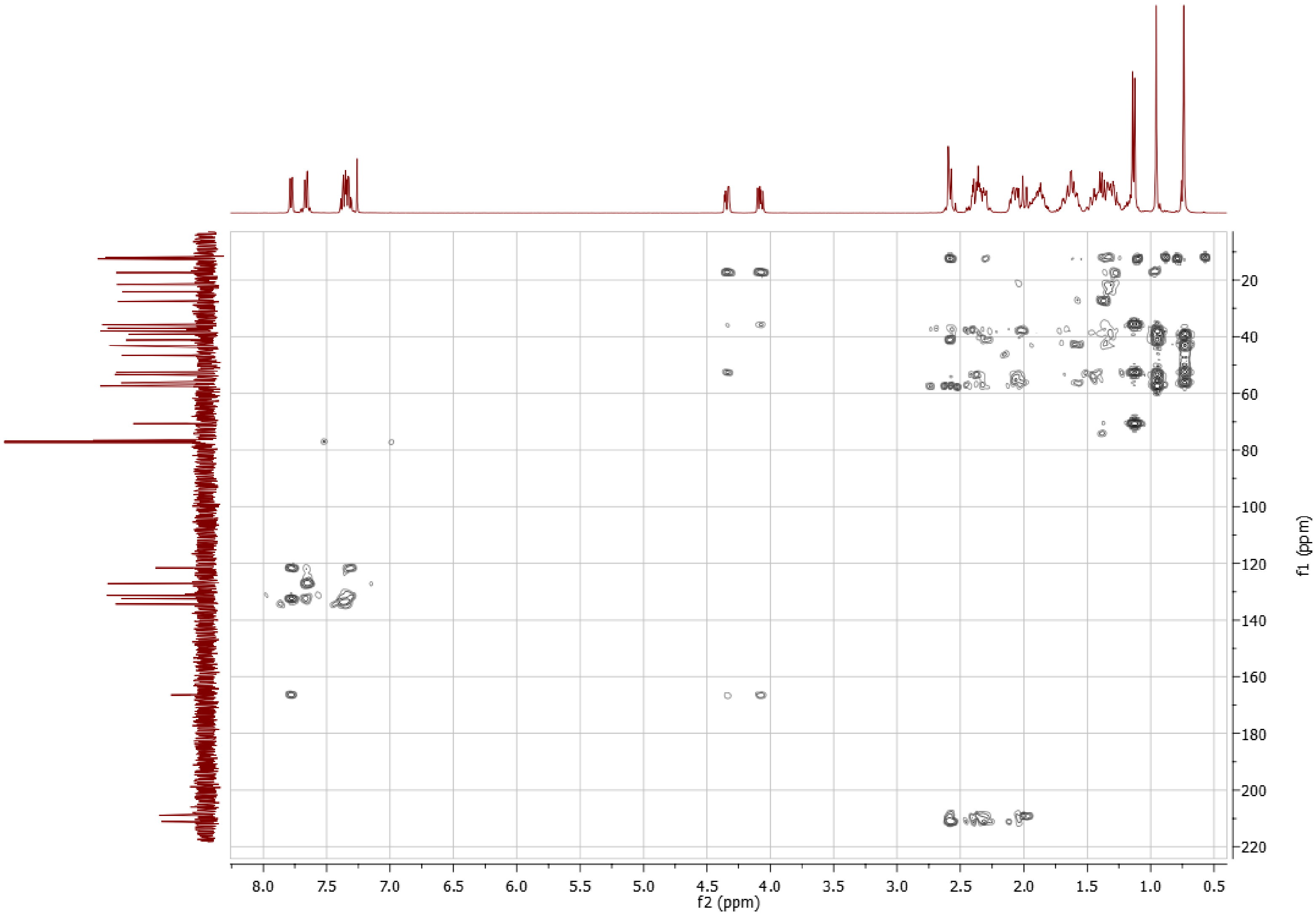

S95. 2D HMBC NMR spectrum of *3,6-dioxo-23,24-dinor-5α-cholan-(2-bromo)-benzoate-22-yl* (***31***).

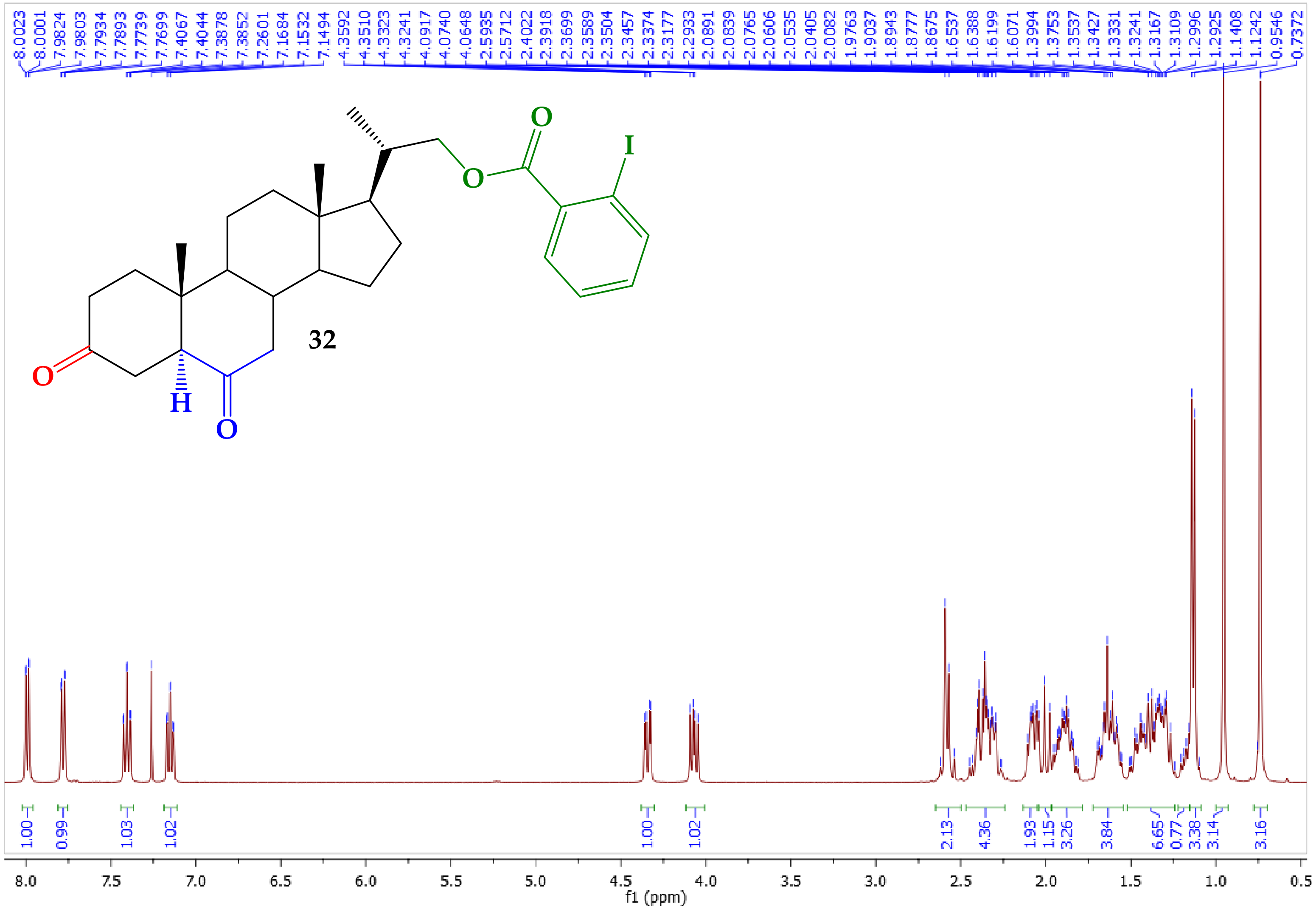

S96. ^{1}H NMR spectrum of *3,6-dioxo-23,24-dinor-5α-cholan-(2-iodo)-benzoate-22-yl* (**32**).

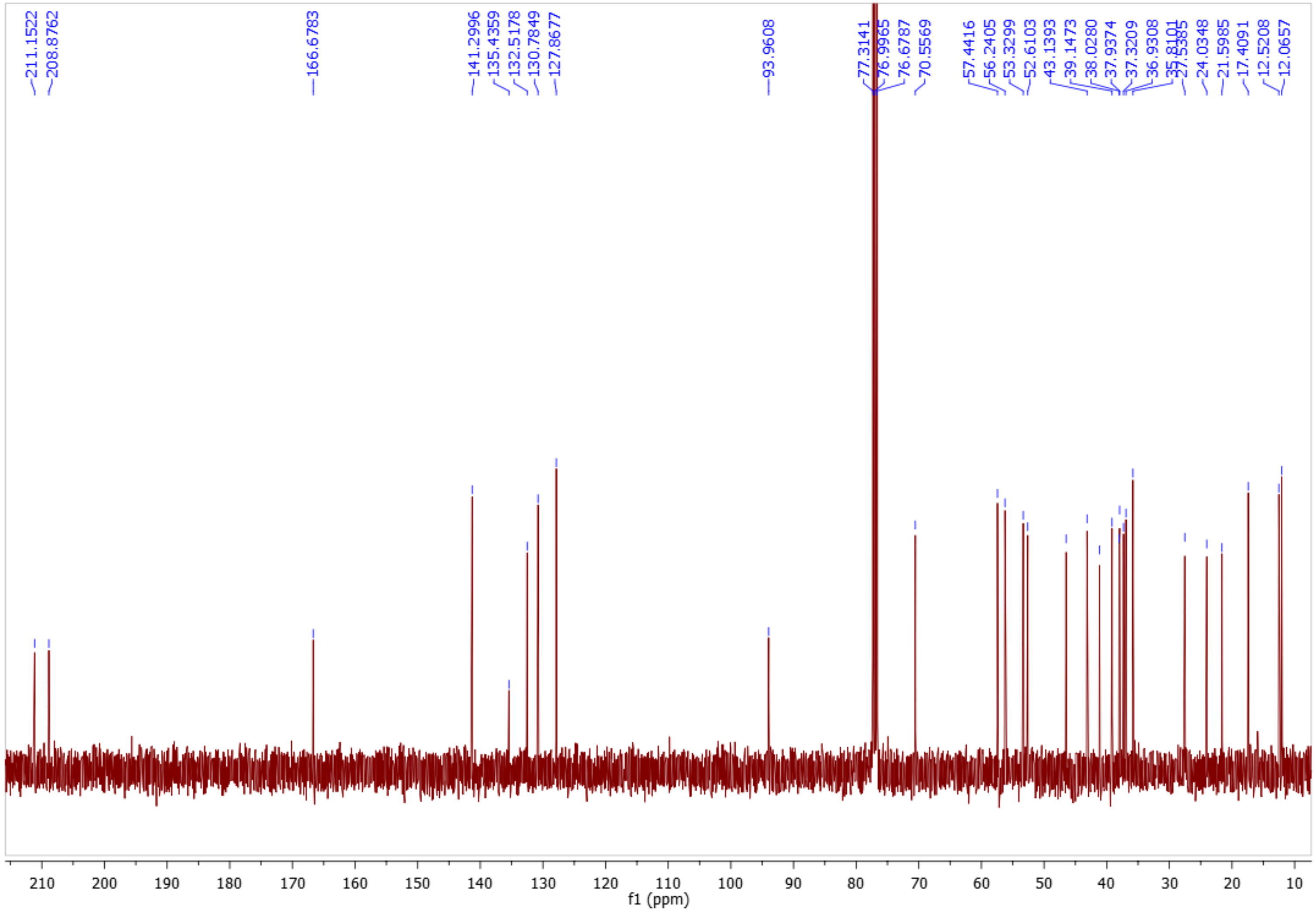

S97. ^{13}C NMR spectrum of *3,6-dioxo-23,24-dinor-5α-cholan-(2-iodo)-benzoate-22-yl* (**32**).

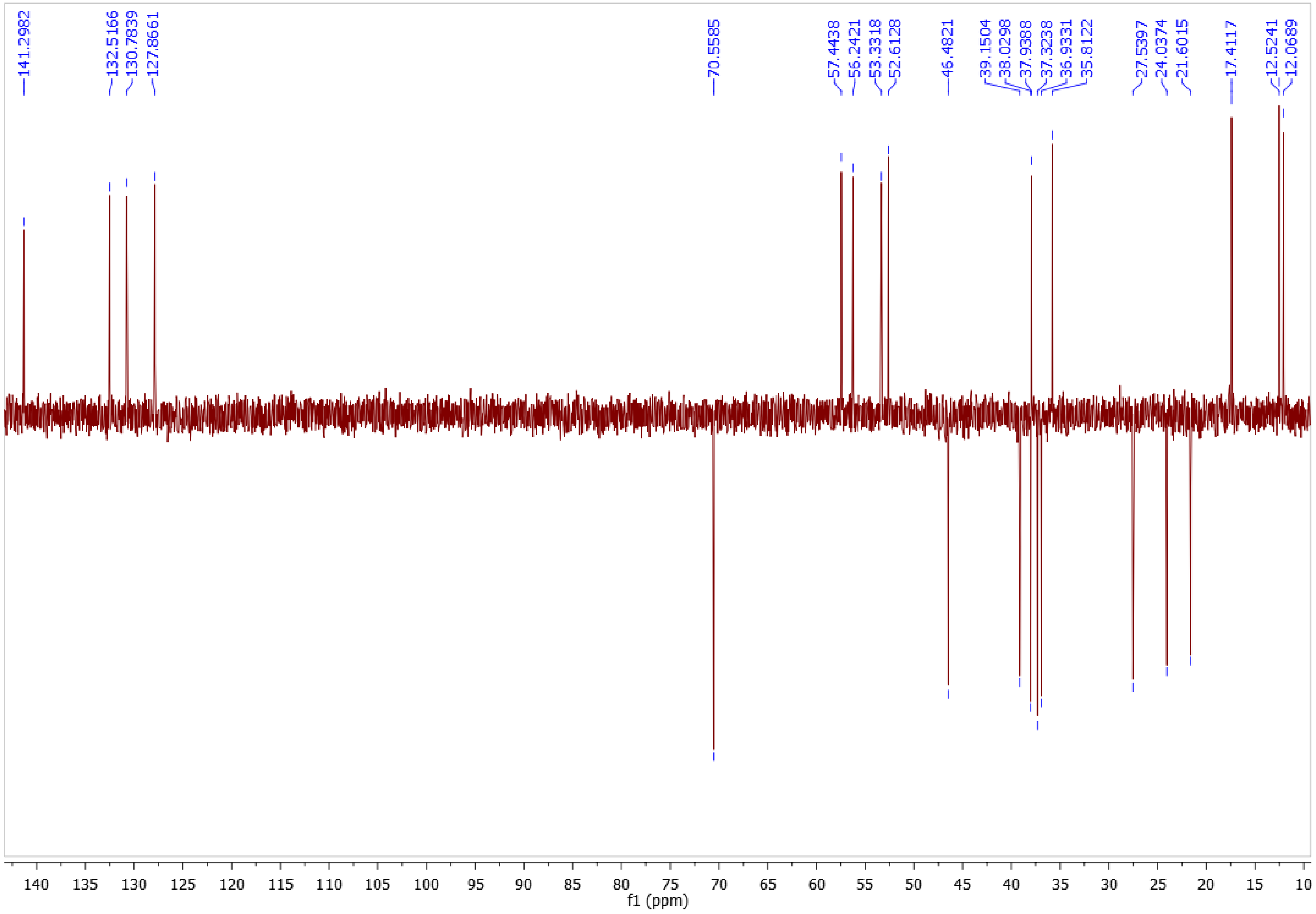

S98. ^{13}C DEPT-135 NMR spectrum of *3,6-dioxo-23,24-dinor-5α-cholan-(2-iodo)-benzoate-22-yl* (**32**).

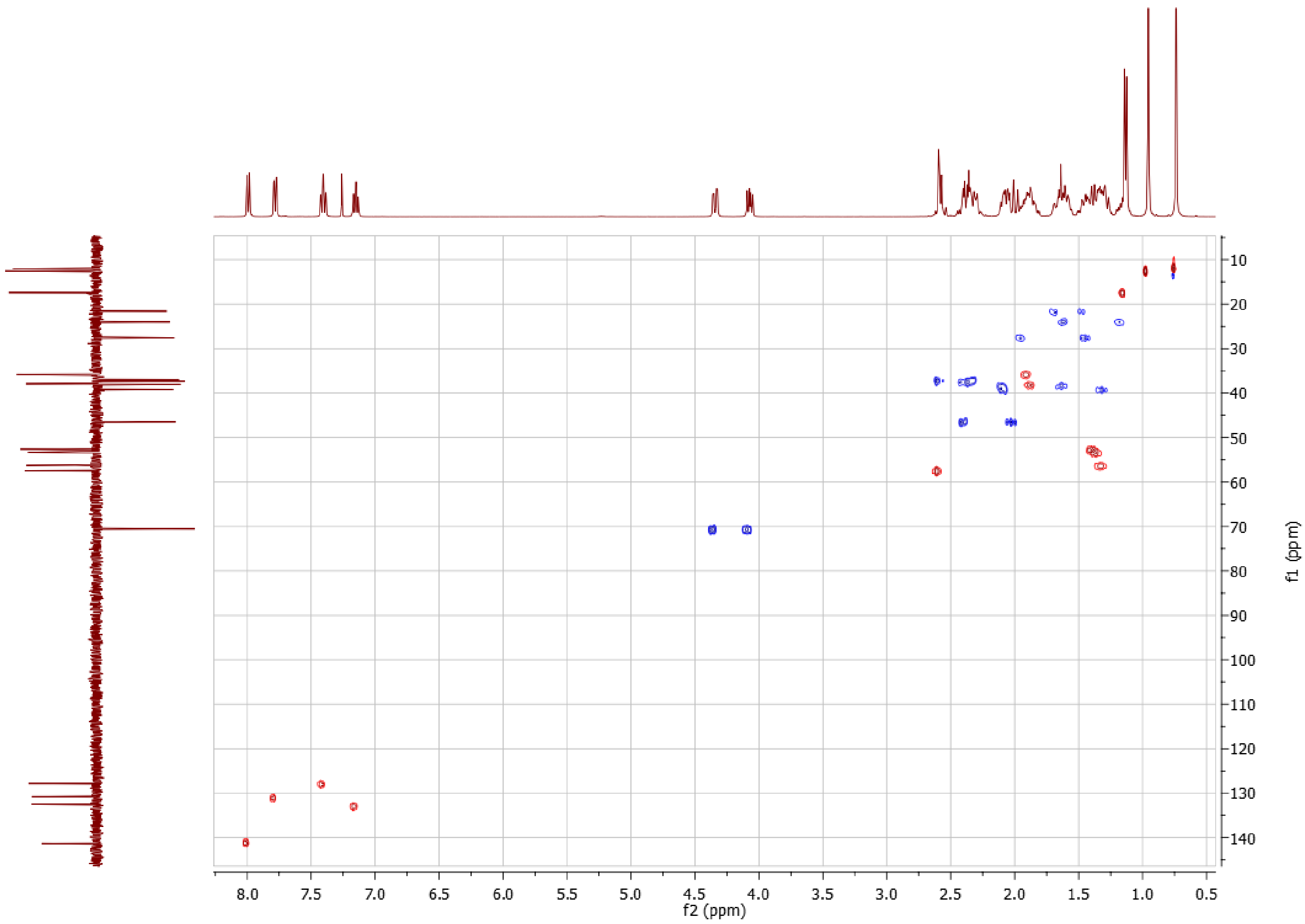

S99. 2D HSQC NMR spectrum of *3,6-dioxo-23,24-dinor-5α-cholan-(2-iodo)-benzoate-22-yl* (**32**).

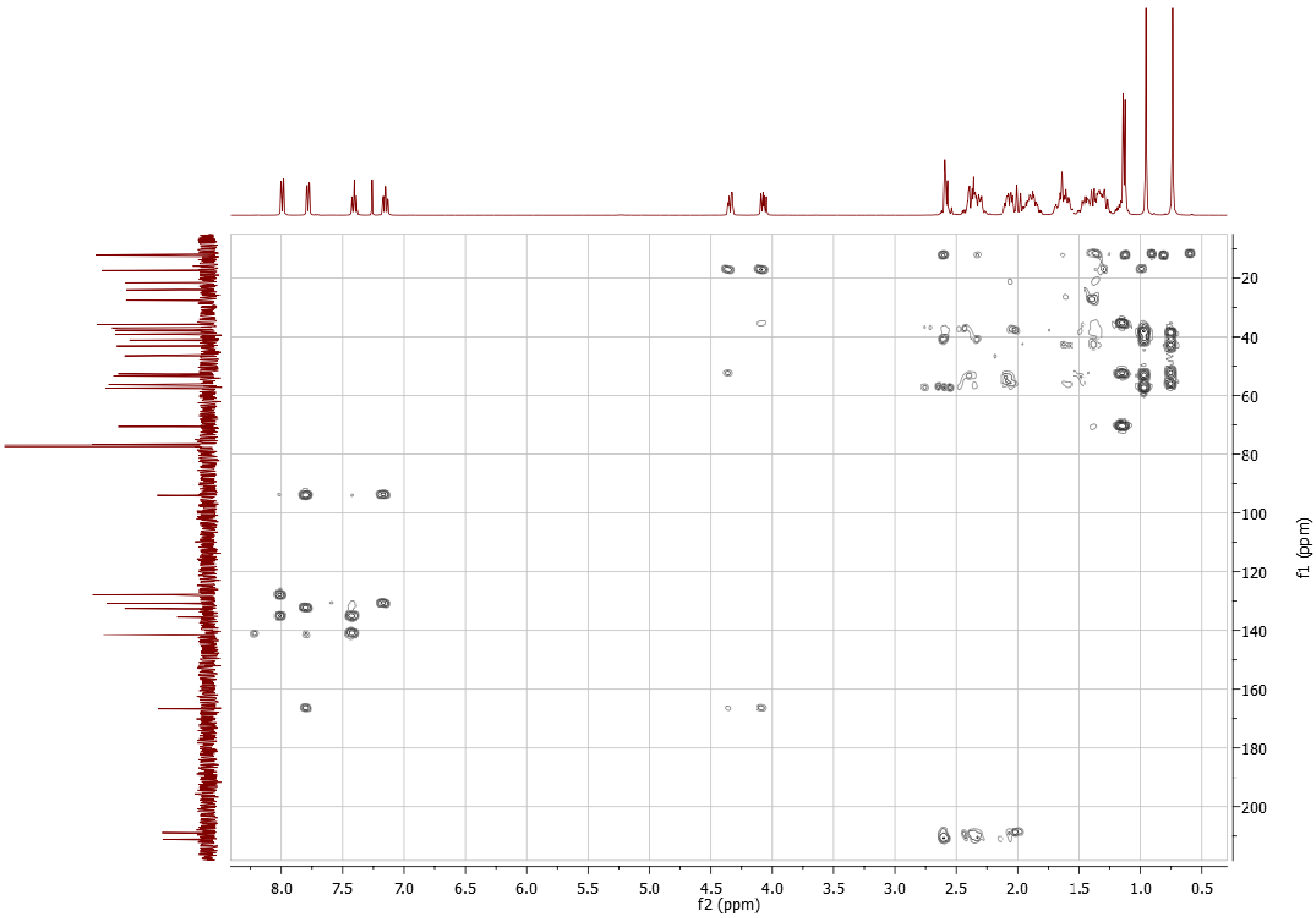

S100. 2D HMBC NMR spectrum of *3,6-dioxo-23,24-dinor-5α-cholan-(2-iodo)-benzoate-22-yl* (**32**).

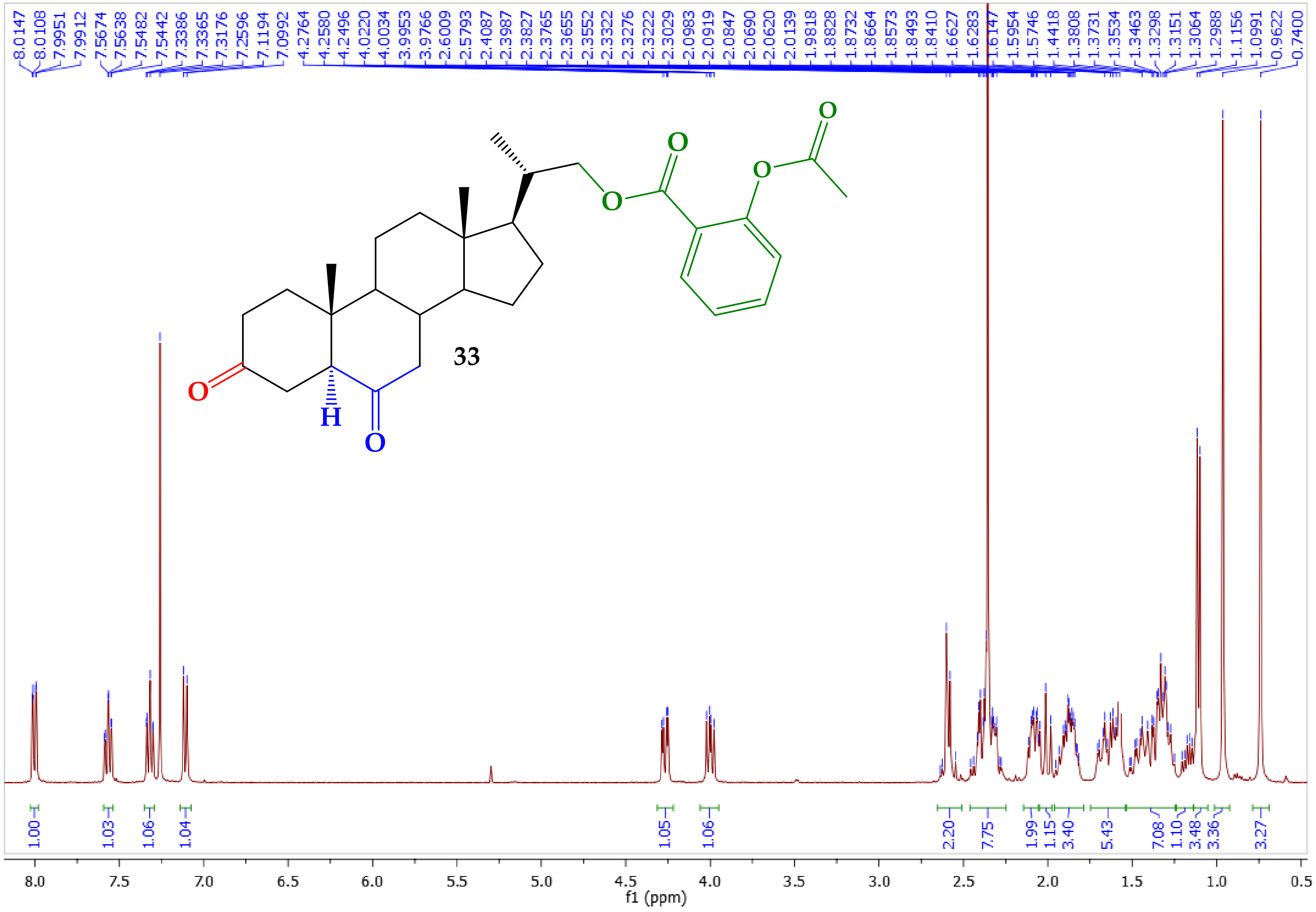

S101. ^{1}H NMR spectrum of *3,6-dioxo-23,24-dinor-5α-cholan-(2-acetoxy)-benzoate-22-yl* (**33**).

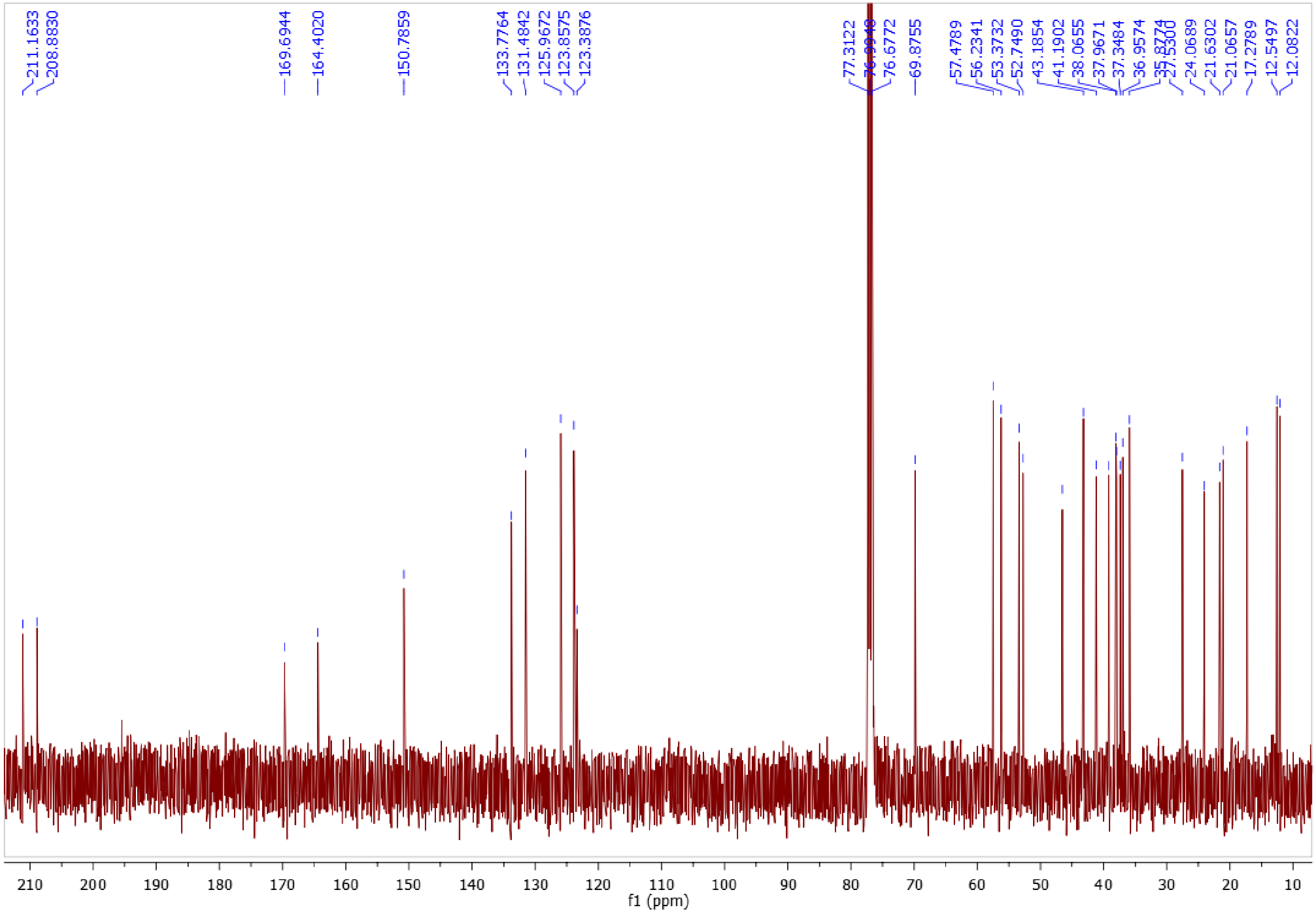

S102. ^{13}C NMR spectrum of *3,6-dioxo-23,24-dinor-5α-cholan-(2-acetoxy)-benzoate-22-yl* (***33***).

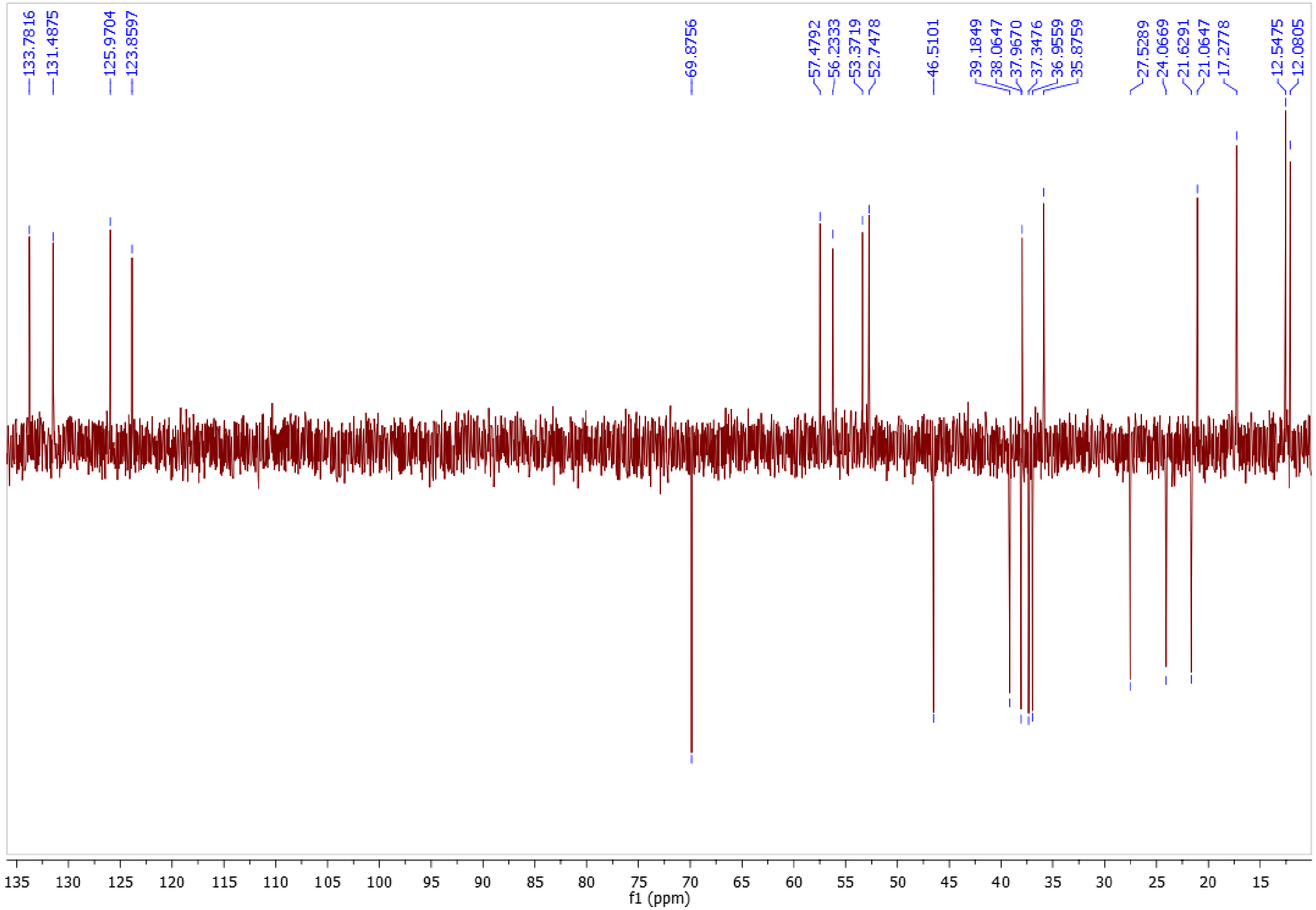

S103. ^{13}C DEPT-135 NMR spectrum of *3,6-dioxo-23,24-dinor-5α-cholan-(2-acetoxy)-benzoate-22-yl* (**33**).

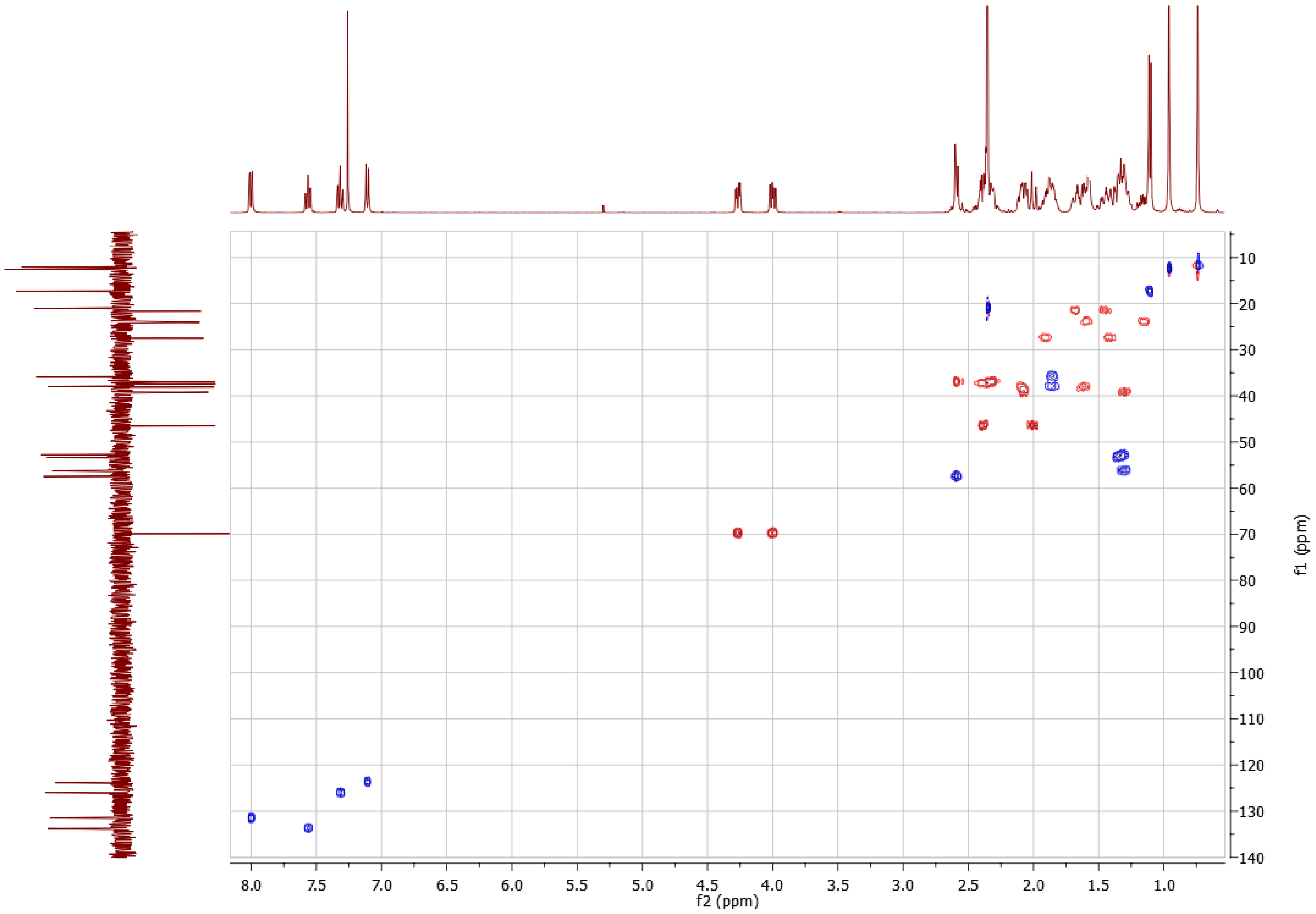

S104. 2D HSQC NMR spectrum of *3,6-dioxo-23,24-dinor-5α-cholan-(2-acetoxy)-benzoate-22-yl* (**33**).

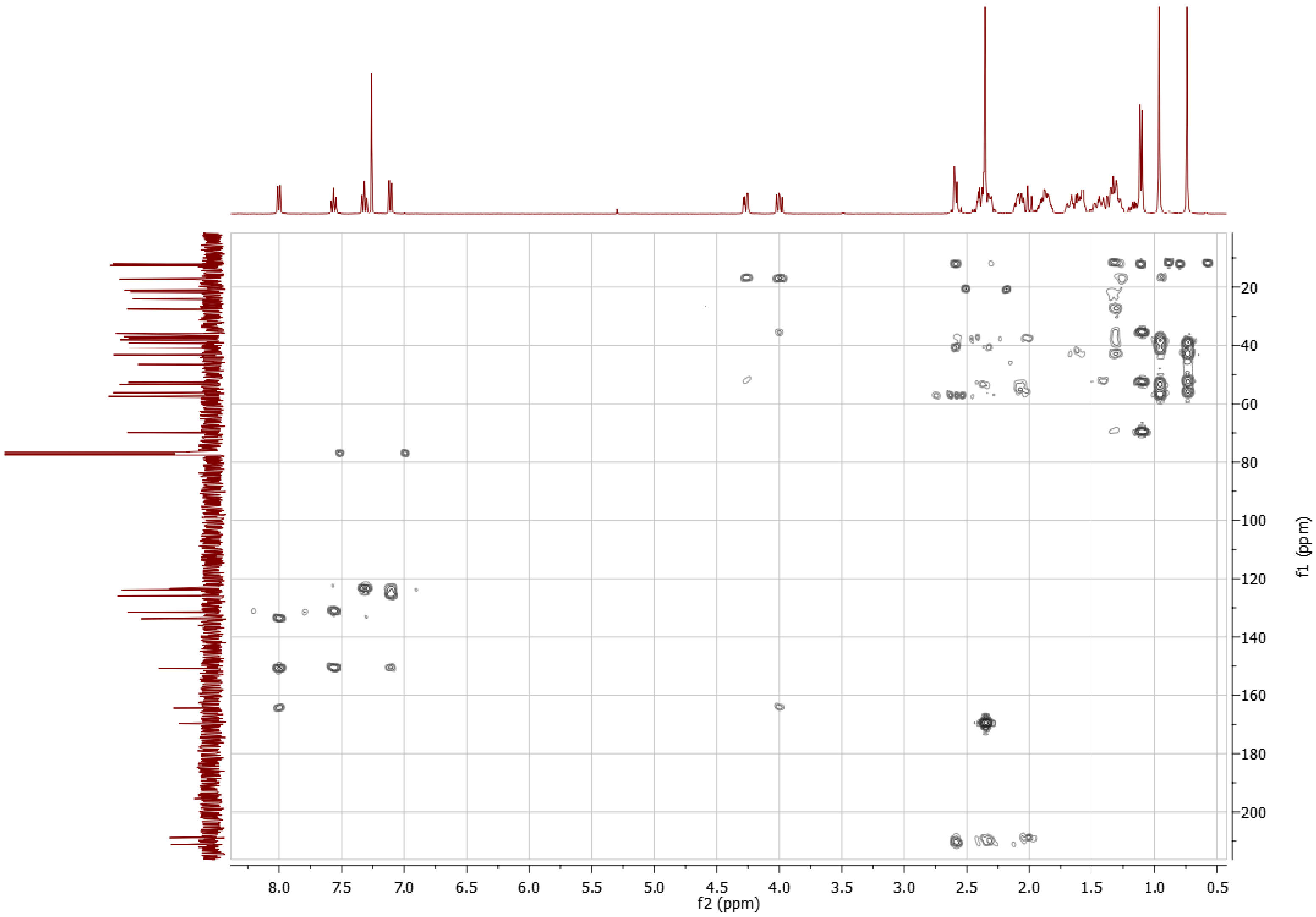

S105. 2D HMBC NMR spectrum of *3,6-dioxo-23,24-dinor-5α-cholan-(2-acetoxy)-benzoate-22-yl* (**33**).

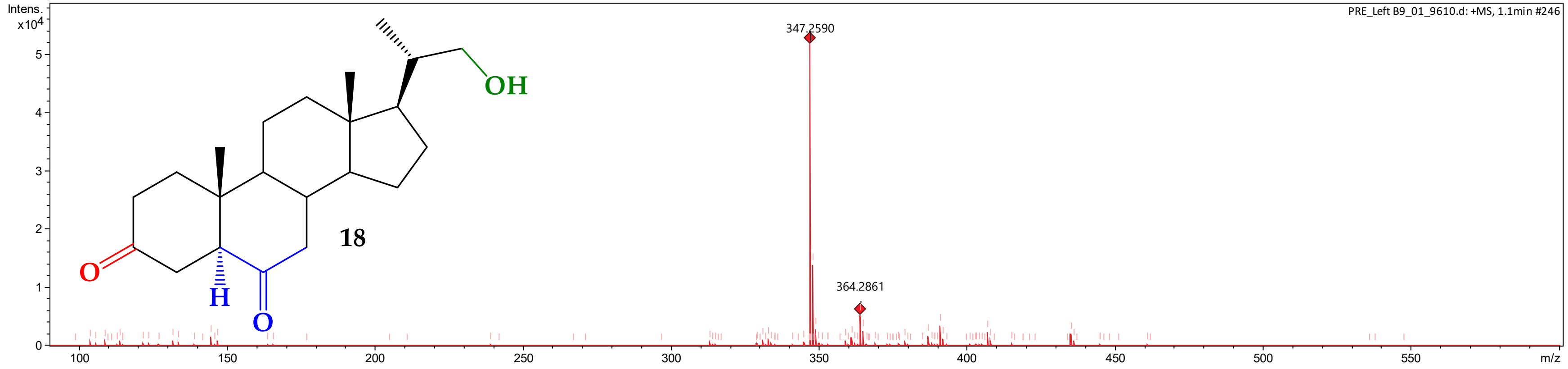

S106. HRSM-ESI spectrum of *22-hydroxy-23,24-dinor-5α-cholan-3,6-dione* (**18**).

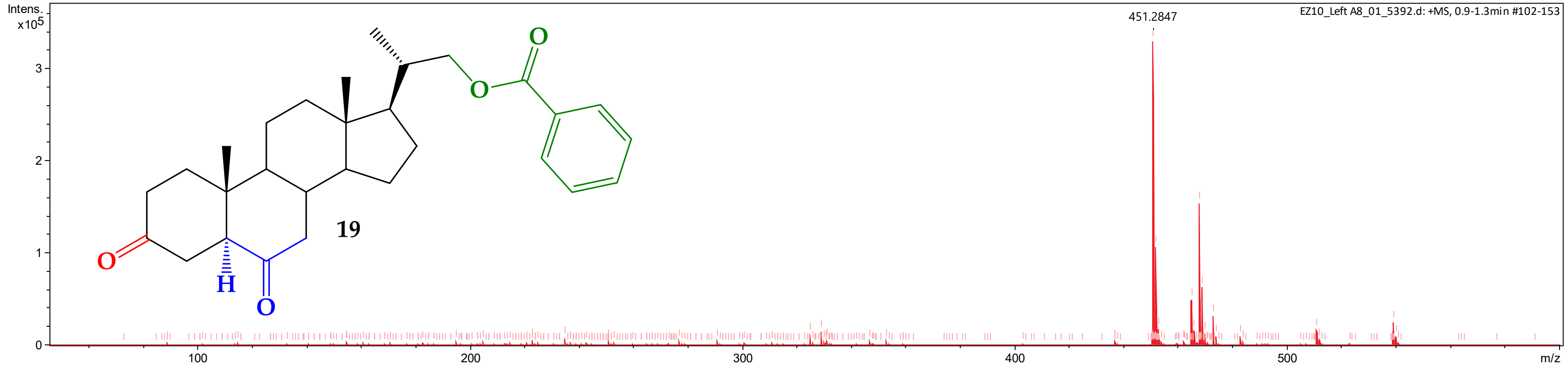

S107. HRSM-ESI spectrum of *3,6-dioxo-23,24-dinor-5α-cholan-benzoate-22-yl* (**19**).

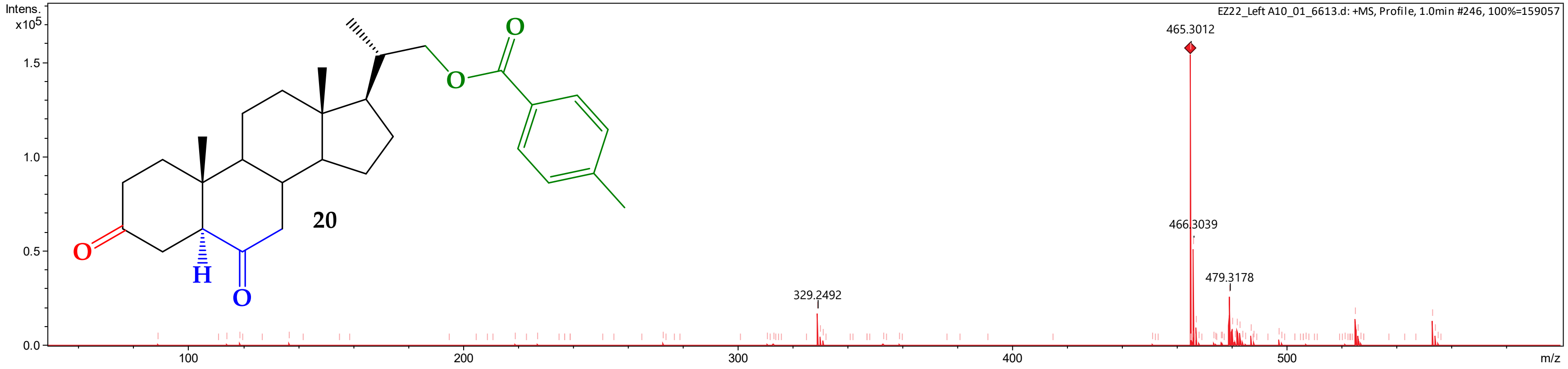

S108. HRSM-ESI spectrum of *3,6-dioxo-23,24-dinor-5α-cholan-(4-methyl)-benzoate-22-yl* (***20***).

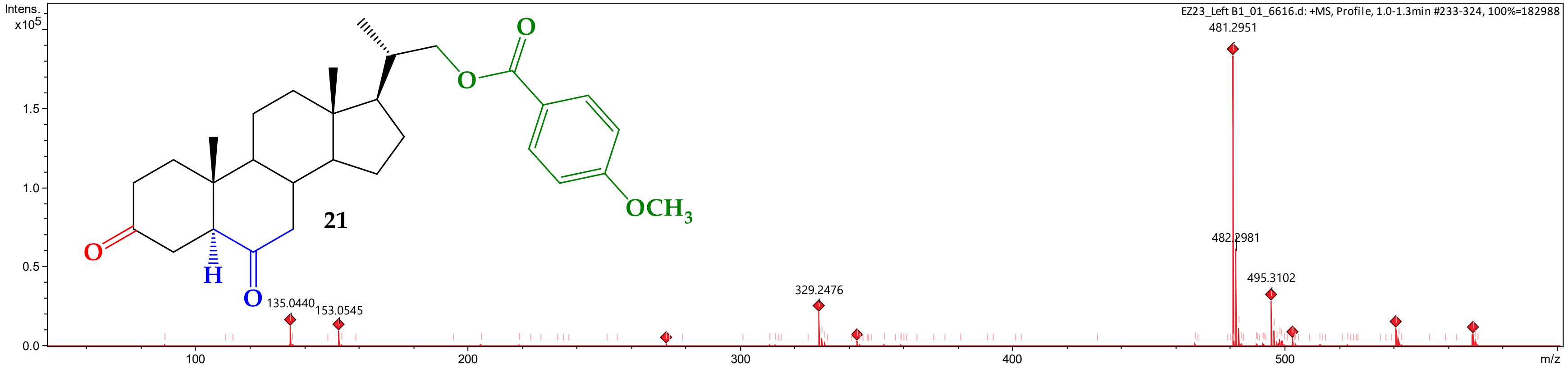

S109. HRSM-ESI spectrum of *3,6-dioxo-23,24-dinor-5α-cholan-(4-methoxy)-benzoate-22-yl* (***21***).

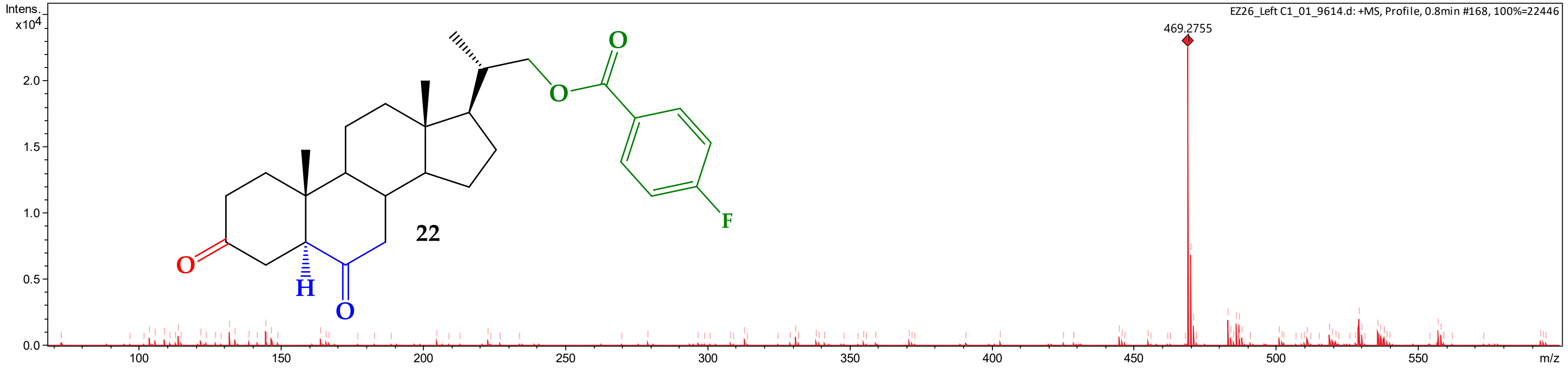

S110. HRSM-ESI spectrum of *3,6-dioxo-23,24-dinor-5α-cholan-(4-fluoro)-benzoate-22-yl* (**22**).

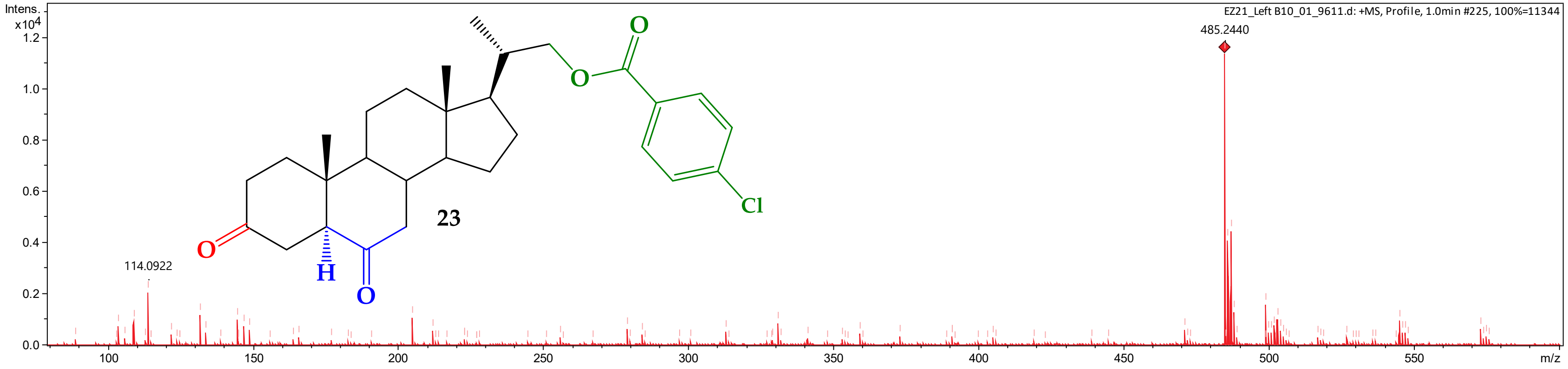

S111. HRSM-ESI spectrum of *3,6-dioxo-23,24-dinor-5α-cholan-(4-chloro)-benzoate-22-yl* (**23**).

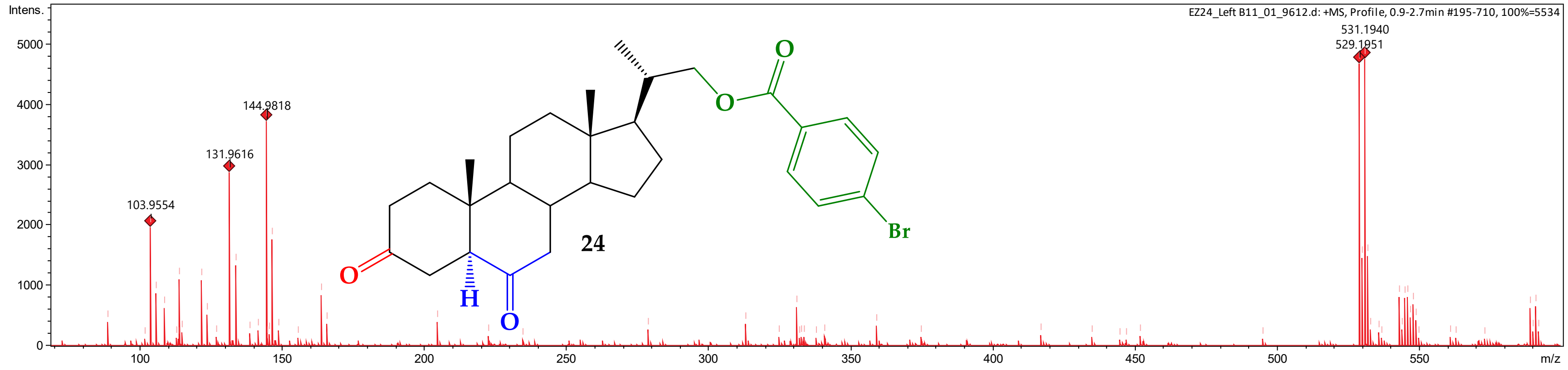

S112. HRSM-ESI spectrum of *3,6-dioxo-23,24-dinor-5α-cholan-(4-bromo)-benzoate-22-yl* (**24**).

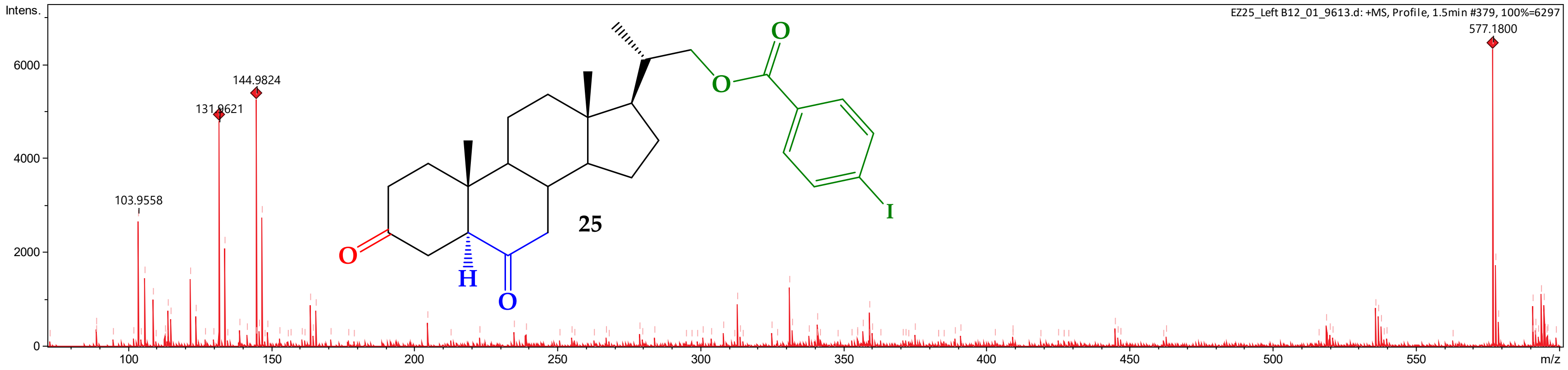

S113. HRSM-ESI spectrum of *3,6-dioxo-23,24-dinor-5α-cholan-(4-iodo)-benzoate-22-yl* (**25**).

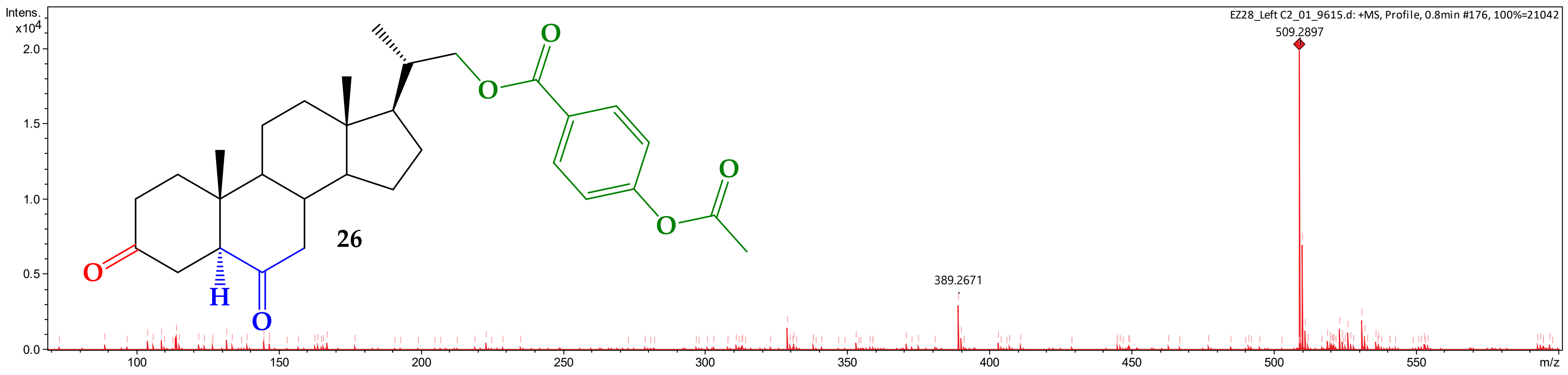

S114. HRSM-ESI spectrum of *3,6-dioxo-23,24-dinor-5α-cholan-(4-acetoxy)-benzoate-22-yl* (**26**).

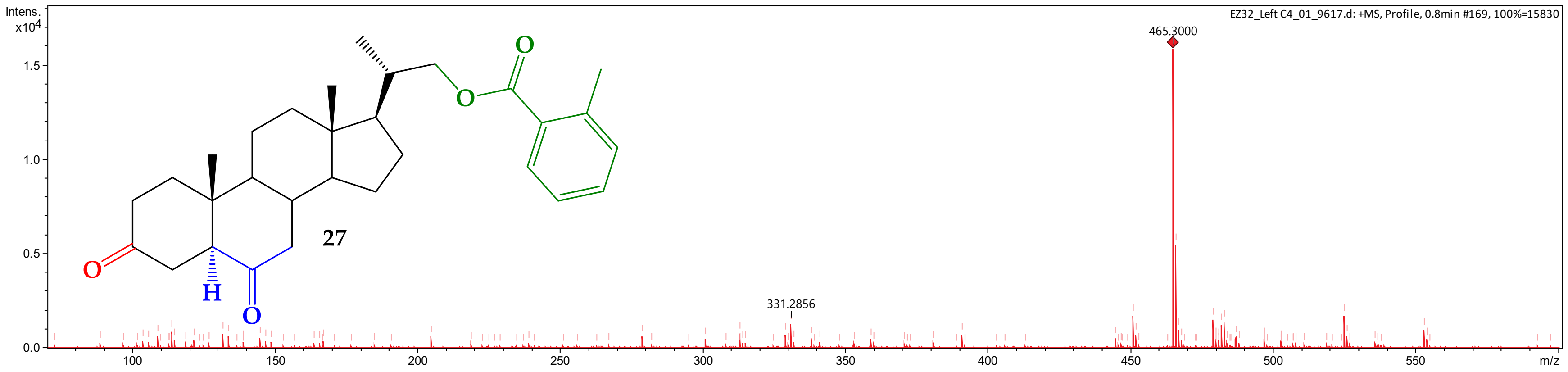

S115. HRSM-ESI spectrum of *3,6-dioxo-23,24-dinor-5α-cholan-(2-methyl)-benzoate-22-yl* (**27**).

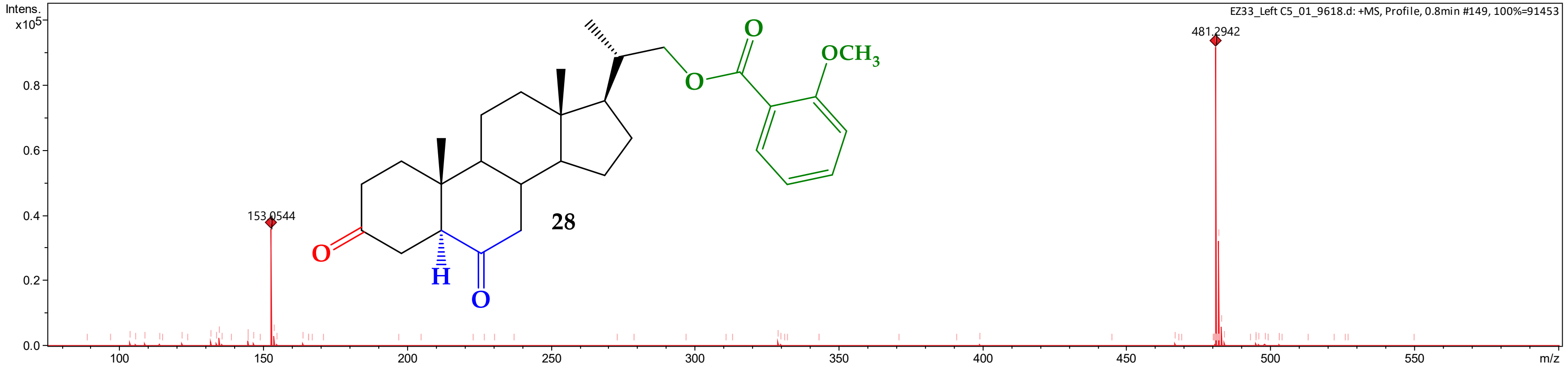

S116. HRSM-ESI spectrum of *3,6-dioxo-23,24-dinor-5α-cholan-(2-methoxy)-benzoate-22-yl* (**28**).

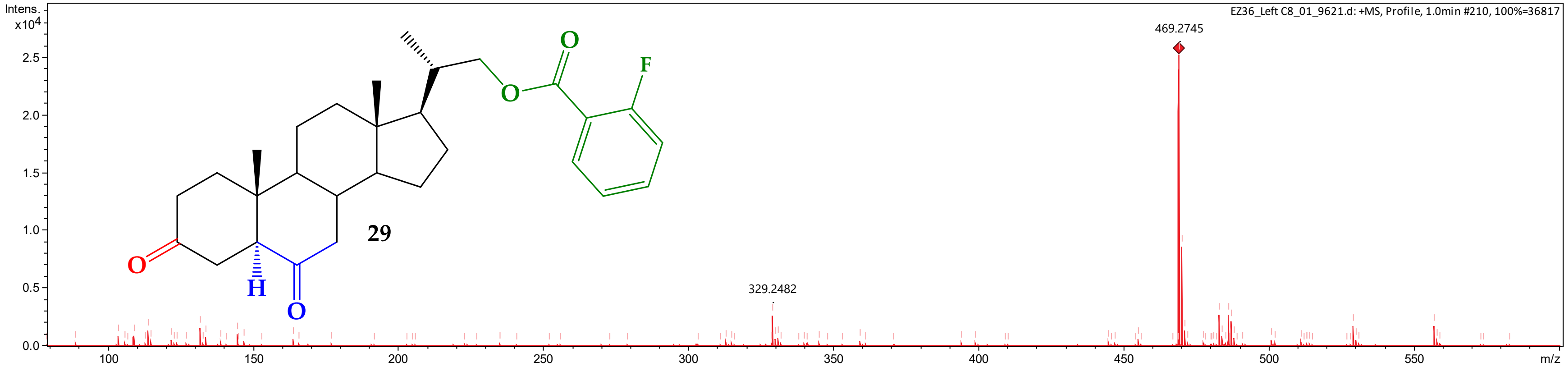

S117. HRSM-ESI spectrum of *3,6-dioxo-23,24-dinor-5α-cholan-(2-fluoro)-benzoate-22-yl* (**29**).

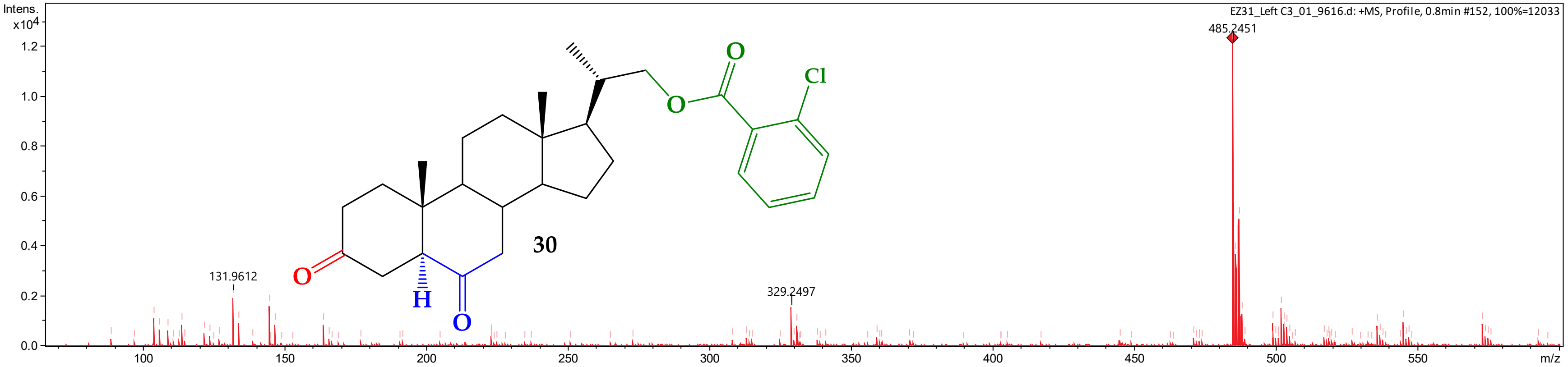

S118. HRSM-ESI spectrum of *3,6-dioxo-23,24-dinor-5α-cholan-(2-chloro)-benzoate-22-yl* (***30***).

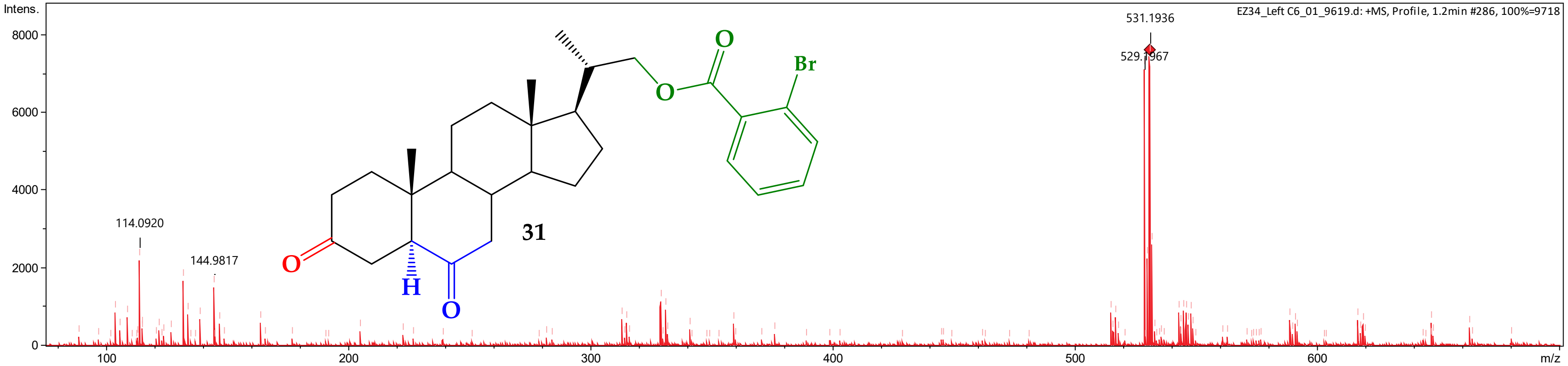

S119. HRSM-ESI spectrum of *3,6-dioxo-23,24-dinor-5α-cholan-(2-bromo)-benzoate-22-yl* (***31***).

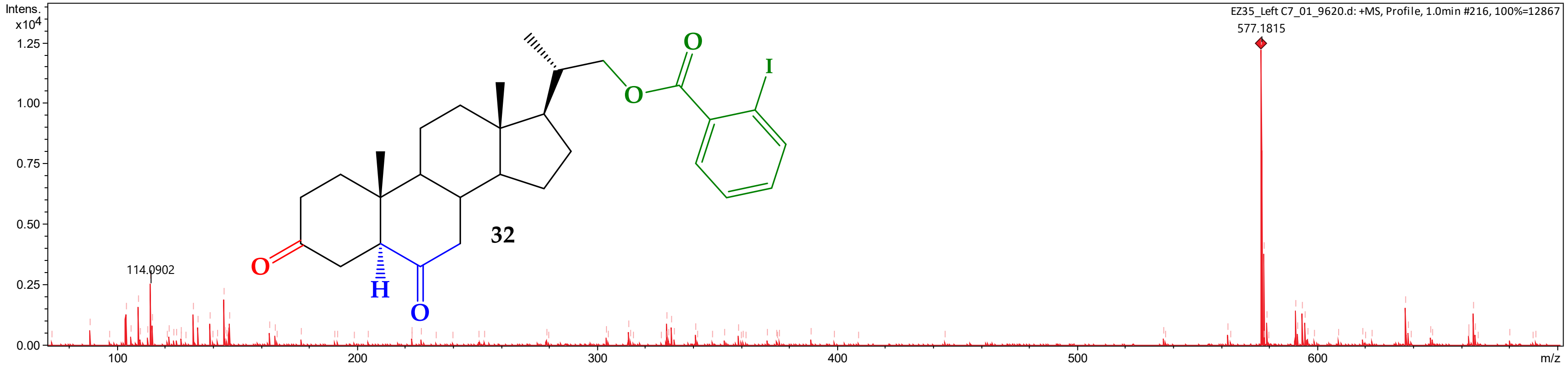

S120. HRSM-ESI spectrum of *3,6-dioxo-23,24-dinor-5α-cholan-(2-iodo)-benzoate-22-yl* (**32**).

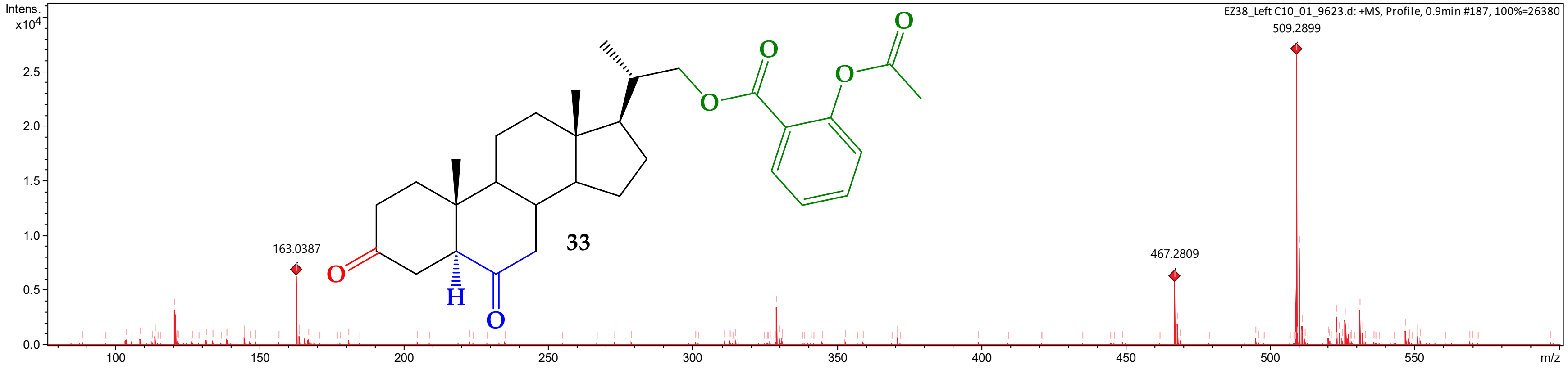

S121. HRSM-ESI spectrum of *3,6-dioxo-23,24-dinor-5α-cholan-(2-acetoxy)-benzoate-22-yl* (**33**).

S122. Rice-lamina assays using the second leaf lamina joints (Angle Opening, Degrees) of excised leaf segments treated with BRs analogs (**2**, **18-33**) at different concentrations: 1 x 10^{-8}, 1 x 10^{-7}, and 1 x 10^{-6} M. Brassinolide (**2**) was used as positive control at the same concentrations.

	**Rice Lamina Inclination (Angle Opening, Degrees)**		
**Compound**	**1x10^{-8} M**	**1x10^{-7} M**	**1x10^{-6} M**
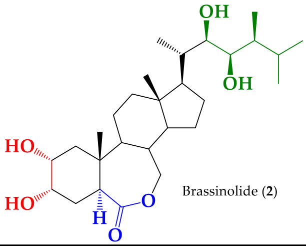 Brassinolide (**2**)	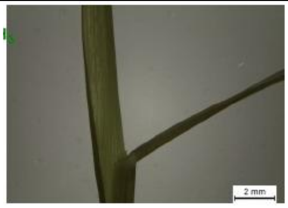 53±7.4[d]	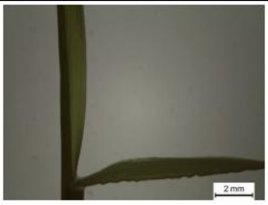 72±3.7[e]	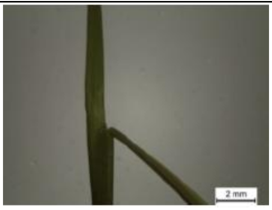 96±7.5[f]
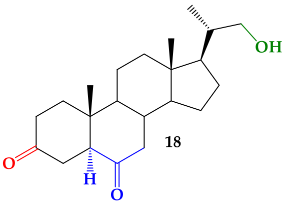 **18**	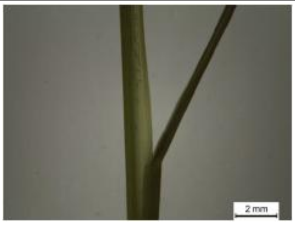	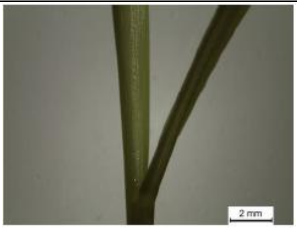 3±3.8[a]	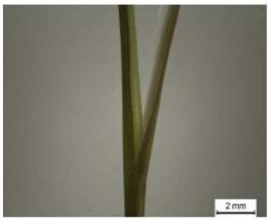 16±4.2[a]
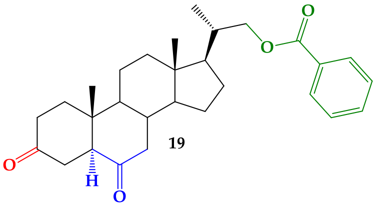 **19**	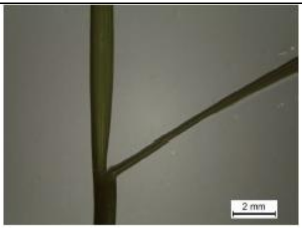 38±5.2[c]	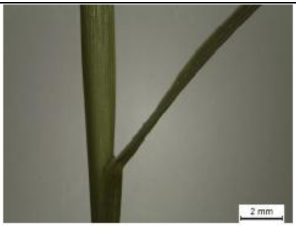 28±4.9[c]	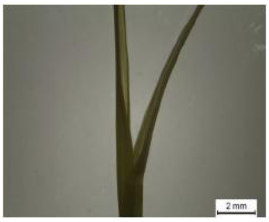 11±4.1[b]
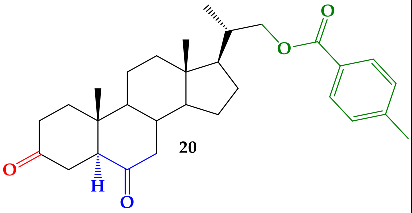 **20**	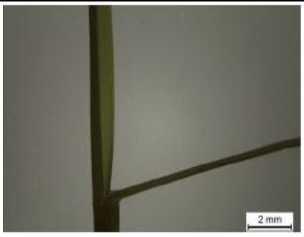 42±8.2[c]	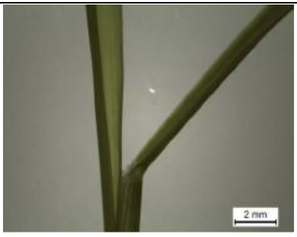 42±4.2[d]	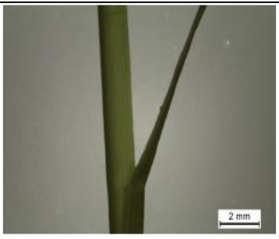 30±4.9[d]
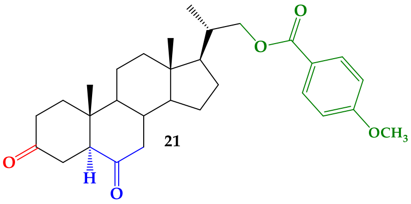 **21**	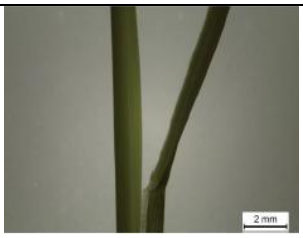 19±5.5[b]	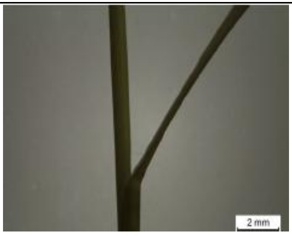 28±4.1[c]	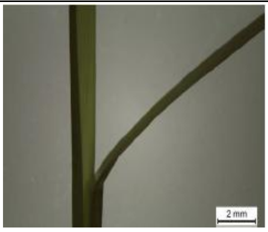 62±8.8[e]

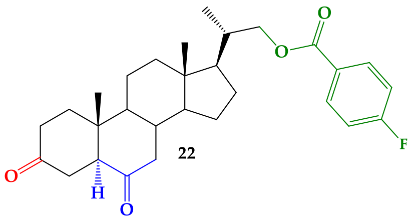 22	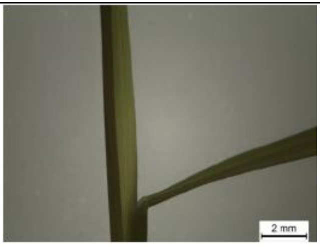 41 ± 5.2^{c}	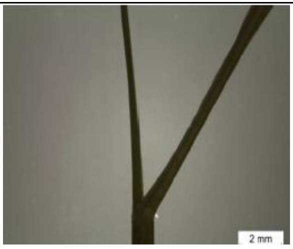 20 ± 2.0^{b}	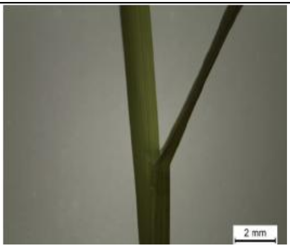 19 ± 4.5^{c}
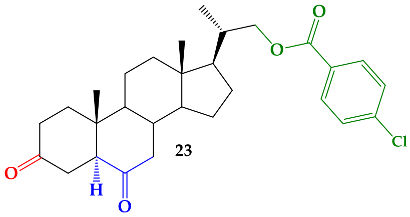 23	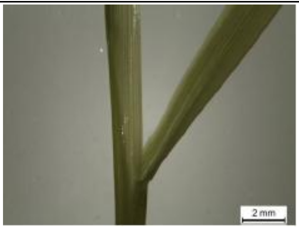 24 ± 3.3^{b}	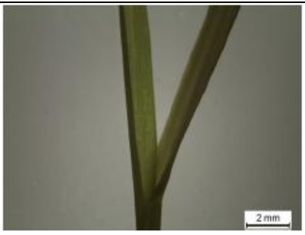 17 ± 2.7^{b}	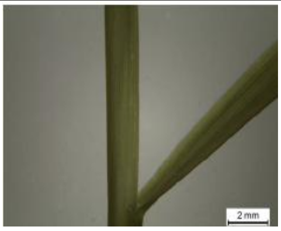 28 ± 4.1^{d}
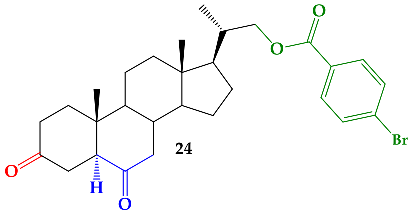 24	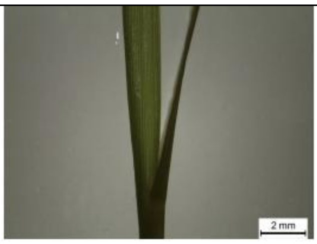 -	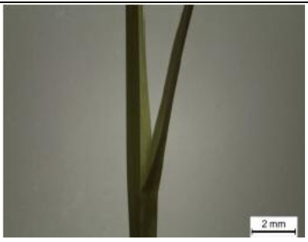 3 ± 1.1^{a}	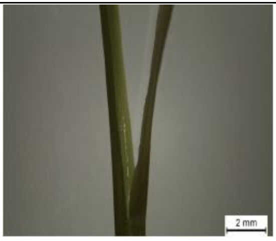 3 ± 1.0^{a}
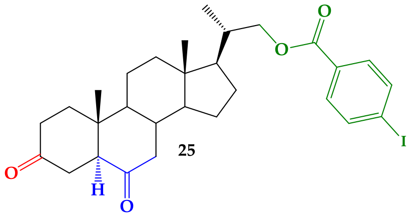 25	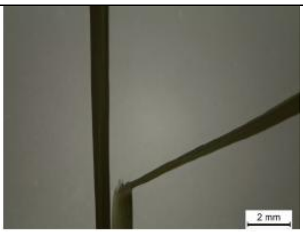 41 ± 8.2^{c}	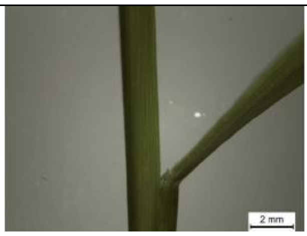 40 ± 2.0^{d}	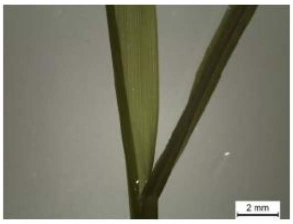 18 ± 2.6^{c}
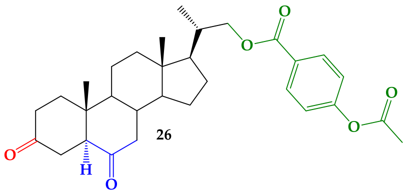 26	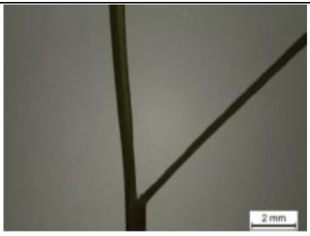 40 ± 3.8^{c}	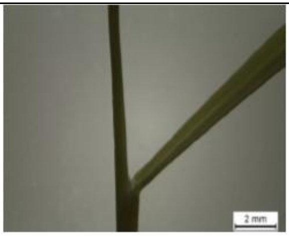 42 ± 2.7^{d}	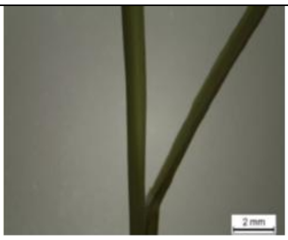 19 ± 5.0^{c}
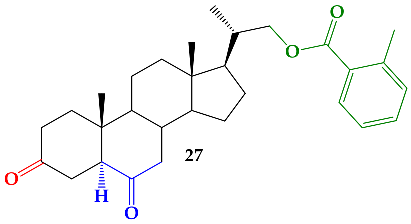 27	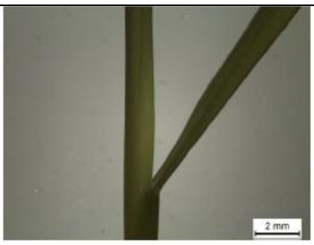 12 ± 5.8^{a}	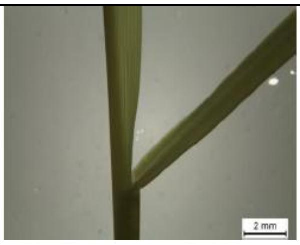 26 ± 5.2^{c}	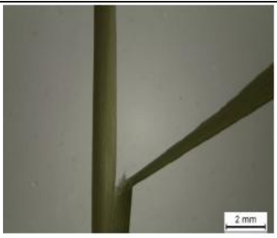 34 ± 4.1^{d}

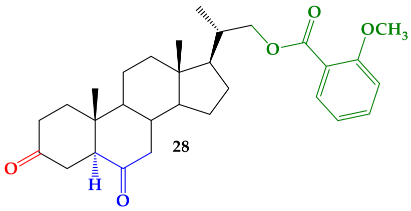 28	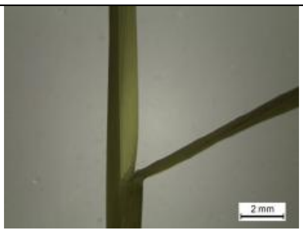 43±8.2^{c}	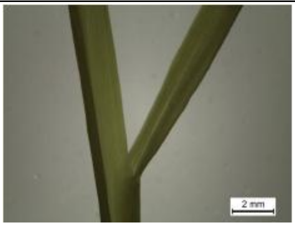 21±4.1^{b}	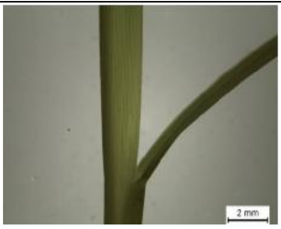 6±2.0^{a}
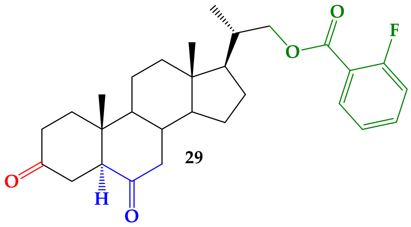 29	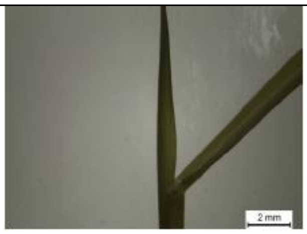 13±4.5^{a}	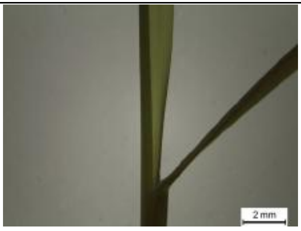 19±26^{b}	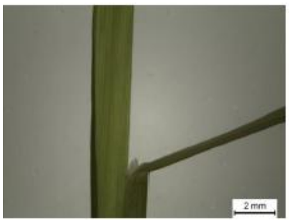 58±3.2^{e}
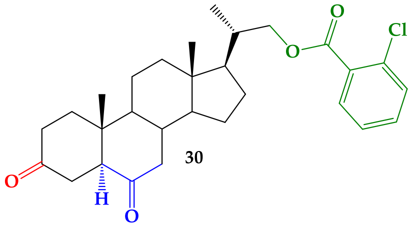 30	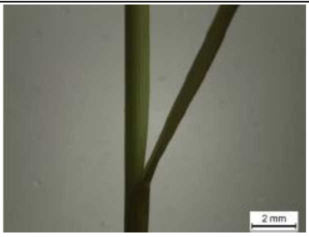 -	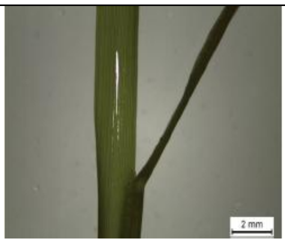 13±4.1^{a}	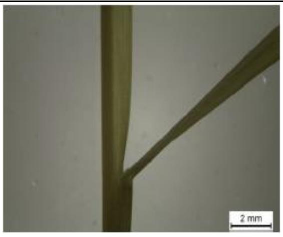 33±4.5^{d}
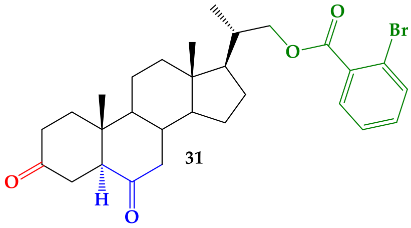 31	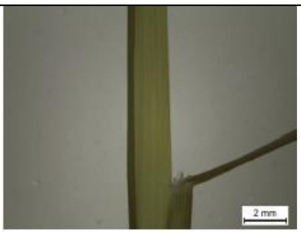 41±5.2^{c}	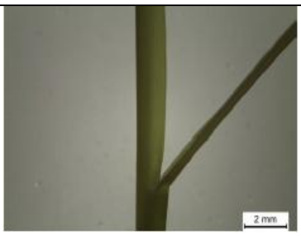 31±2.6^{d}	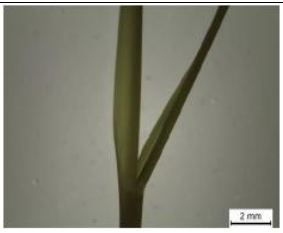 16±7.5^{d}
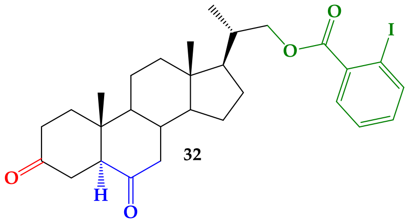 32	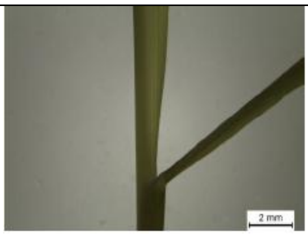 45±2.3^{c}	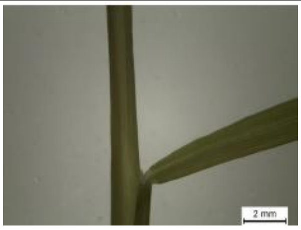 32±4.9^{d}	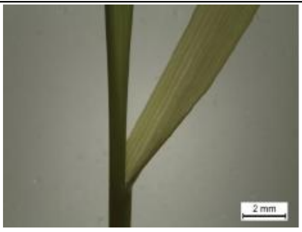 22±3.8^{c}
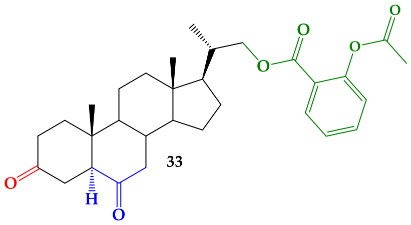 33	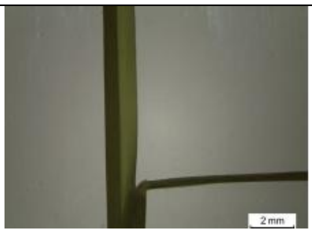 63±6.6^{d}	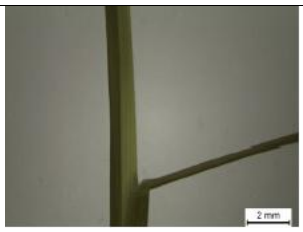 43±4.5^{e}	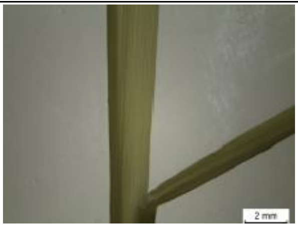 24±2.6^{c}
**Negative control (H_2O)**			

		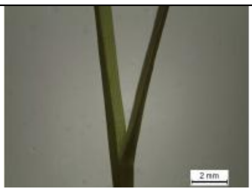 23 ± 2.7^{b}	

S123. Effect of 3-DT analogs **18-33** and positive control (brassinolide (**2**)) on the elongation bean second internode bioassay at a concentration of 1×10^{-8} M.

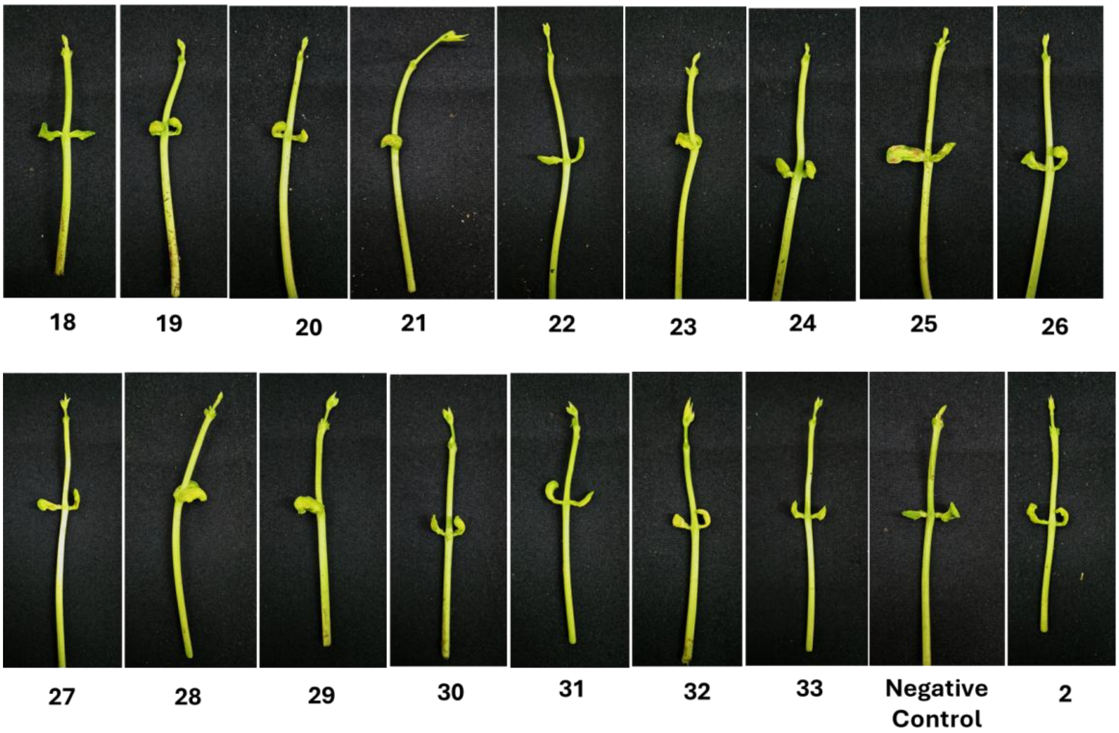

Table S1. Effect of different concentrations of brassinolide (**2**) and **3-DT** derivatives on lamina inclination of rice seedlings: precursor **18**; 3,6-dioxo-22-(4-substituted)-benzoates (**19**-**26**) and 3,6-dioxo-22-(2-substituted)-benzoates (**27**-**33**).

	**Bending Angles Between Laminae and Sheaths** (Degrees ± Standard Error)[1]		
**Compounds**	**1 x 10^{-8} M**	**1x10^{-7} M**	**1 x 10^{-6} M**
**Brassinolide (2)**	53 ± 7.4	72 ± 3.7	96 ± 7.5
**18**	-	3 ± 3.8	16 ± 4.2
**19**	38 ± 5.2	28 ± 4.9	11 ± 4.1
**20**	42 ± 8.2	42 ± 4.2	30 ± 4.9
**27**	12 ± 5.8	26 ± 5.2	34 ± 4.1
**21**	19 ± 5.5	28 ± 4.1	62 ± 8.8
**28**	43 ± 5.8	21 ± 4.1	6 ± 2.0
**22**	41 ± 5.2	20 ± 2.0	19 ± 4.5
**29**	13 ± 4.5	19 ± 2.6	58 ± 3.2
**23**	24 ± 3.3	17 ± 2.7	28 ± 4.1
**30**	-	13 ± 4.1	33 ± 4.5
**24**	-	3 ± 1.1	3 ± 1.0
**31**	41 ± 5.2	31 ± 2.6	16 ± 7.5
**25**	41 ± 8.2	40 ± 2.0	18 ± 2.6
**32**	45 ± 2.3	32 ± 4.9	22 ± 3.8
**26**	40 ± 3.8	42 ± 2.7[d}]	19 ± 5.0
**33**	63 ± 6.6	43 ± 4.5	24 ± 2.6

[1] Values represent the mean ± standard deviation of two independent experiments with at least eight replicates each. Average angle of negative control: 23 ± 2.7. (-): values lower than negative control. Brassinolide (**2**) was used as positive control.
